# Influence of Ion Substitution on the Properties of Apatite-Based Materials: Computational Predictions Using Density Functional Theory

**DOI:** 10.1021/acsomega.4c09997

**Published:** 2025-05-24

**Authors:** Henrique S. Marques, Albert F. B. Bittencourt, Juarez L. F. Da Silva

**Affiliations:** † Institute of Mathematics and Computer Sciences, 153988 University of São Paulo, 13566-590 São Carlos, SP, Brazil; ‡ Institute of Science and Technology, 676681 Federal University of Jequitinhonha and Mucuri Valleys, 39100-000 Diamantina, MG, Brazil; § São Carlos Institute of Chemistry, University of São Paulo, P.O. Box 780, 13560-970, São Carlos, SP, Brazil

## Abstract

Apatite-based materials have attracted recognition as promising candidates for catalytic applications because of their tunable properties that can be achieved through ionic substitutions and their compatibility with sustainability goals for environmentally friendly catalysts. However, a thorough understanding of their physicochemical properties at the atomic level remains insufficient. In this study, calculations based on density functional theory combined with Spearman’s correlation are used to investigate the effects of cationic and anionic substitutions on the structural, energetic, and electronic properties of materials similar to apatite with Ca/P ratios ranging from 0.50 to 2.00. Our results reveal that substitutions with d-block elements, such as Zn and Cd, reduce the energy gap at the Γ-point and decrease the ionic character of the materials, leading to reduced stability. Additionally, d-p orbital hybridization within the PO_4_^3–^, AsO_4_^3–^, and VO_4_^3–^ groups significantly influences structural stability. Using Spearman’s correlation analysis, we identified significant trends, specifically indicating a strong correlation between net atomic charges, energy gaps, and cohesive energy. These results offer critical insights into how ionic substitutions influence the tunable characteristics of materials resembling apatite.

## Graphical abstract

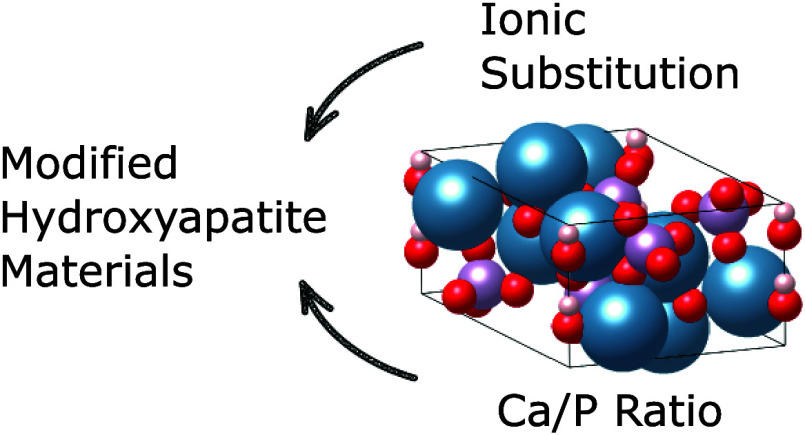

## 1 Introduction

Apatite-based materials, which comprise a class of compounds based on hydroxyapatite (HAP), have been identified as promising candidates within the domain of heterogeneous catalysis. HAP has a specific chemical formula C_10_(PO_4_)_6_(OH)_2_, which contains cationic (Ca^2+^) and anionic (PO_4_^3–^, OH^1–^) species within the crystal structure. Thus, it leads to the presence of both acidic and basic active sites on the surface of HAP particles, which helps to explain its success in catalysis.^1–3^ This unique set of features also opens the possibility for its utilization in a broad range of catalytic processes, including carbon–carbon cross-coupling,^4^ condensation,^5^ oxidation reactions,^6^ and more.^7^ Furthermore, HAP is consistent with the principles of green chemistry^8^ owing to its nontoxicity, high stability, reusability, and abundance.^9^ These attributes establish HAP as an alternative to traditional catalysts, especially in the context of sustainable chemistry applications.^10^

An outstanding characteristic of HAP is its remarkable ion-exchange capacity,^11^ allowing the substitution of calcium ions (Ca^2+^) and phosphate groups (PO_4_^3–^) with various cations (e.g., Mg^2+^,^12^ Sr^2+^,^13^ Ba^2+^,^14^ Fe^2+^,^14^ Zn^2+^ ^15^) and anions (e.g., CO_2_^3–^,^16^ AsO_4_^3–^,^17^ VO_4_^3–^ ^18^), respectively. Thus, the ion exchange capability allows for fine-tuning of surface reactivity, optimizing acidic, basic, and redox properties for targeted catalytic applications.^19^ For example, Ho et al.^20^ demonstrated that the activity of HAP catalysts can be modulated by substituting Ca^2+^ with other alkaline earth metals, which affects the strength of the acidic and basic sites, enhancing the stabilization of intermediate species during condensation reactions. Similarly, Ramesh et al.^21^ showed that replacing PO_4_^3–^ with anions such as WO_4_^2–^ or SO_4_^2–^ can regulate dehydrogenation and dehydration activities in ethanol conversion processes.

As recognized by Yook et al.,^19^ the adaptability of HAP as a catalytic material can be significantly increased through the concurrent substitution of multiple elements. Given that various interchangeable sites are available in the HAP structure (i.e., Ca^2+^, PO_4_^3–^, and OH^–^), multiple substitutions could maximize catalytic efficiency and expand the potential applications of HAP-based catalysts. Although only a few studies have explored this direction, it has been shown that substitutions with elements such as Ni and Ce in HAP have the potential to enhance catalytic performance.^22^ In recent studies, Gadipelly et al.^23^ investigated the efficacy of doping HAP in facilitating bond formation reactions, specifically focusing on the C–C and C–N bonds.

In its stoichiometric composition, HAP is characterized by a Ca/P molar ratio of 1.67. However, this ratio is subject to variation based on the synthesis techniques applied, which leads to diverse crystallographic structures exhibiting distinct morphologies and distributions of surface sites.^19^ For instance, varying the Ca/P ratio permits the acquisition of different phases, such as monocalcium phosphate anhydrous (Ca­(H_2_PO_4_)_2_, Ca/P = 0.50),^24^ dicalcium phosphate anhydrous (CaHPO_4_, Ca/P = 1.00),^25^ tricalcium phosphate (γ-Ca_3_(PO_4_)_2_, Ca/P = 1.50),^26^ and tetracalcium phosphate (Ca_4_(PO_4_)_2_O, Ca/P = 2.00).^27^ Consequently, the unique morphologies and distributions of surface active sites intrinsic to these phases may be investigated to enhance the performance of HAP-based materials, in particular catalytic reactions.

In this study, we investigate the variations in the Ca/P molar ratio along with ionic substitutions to determine whether the effects of substitutions are contingent on the Ca/P ratio or remain uniform across different crystallographic phases. To achieve this objective, we performed density functional theory (DFT) calculations to explore the energetic, electronic, and structural implications of ionic substitution within various HAP phases. Using Spearman’s correlation analysis, we investigated the most important factors that affect the physicochemical stability of apatite-based materials. Our results provide valuable information on the tunable properties of HAP and its potential aptitude for tailored catalytic applications, thus contributing to the formulation of sustainable and economically viable catalysts.

## 2 Theoretical Approach and Computational Details

### 2.1 Total Energy Calculations

Our first-principles calculations are based on spin-polarized DFT^28,29^ within the Perdew–Burke–Ernzenhof (PBE) formulation^30^ for the exchange-correlation energy functional, as implemented in the Vienna *ab initio* simulation package (VASP),^31,32^ version 5.4.4. The all-electron projected augmented-wave (PAW) method^33,34^ was used to describe core–valence electron interactions, and the Kohn–Sham (KS) equations were solved with KS states expanded in plane waves.

For relaxation of the lattice parameters and atomic positions, a plane-wave cutoff energy of 869 eV was used to ensure accurate convergence of the stress tensor, which shows slow convergence as a function of the number of plane waves. All remaining calculations used a cutoff energy of 548 eV, which is 12.5% higher than the highest recommended value for the chemical elements in the bulk materials. The integration of the Brillouin zone (BZ) was sampled using a Monkhorst–Pack^35^ **k**-mesh of 2 × 2 × 3, which was increased to 4 × 4 × 6 for the density of states (DOS) calculations to obtain accurate results. For free atom calculations, an orthorhombic box of dimensions 20 × 21 × 22 Å was used, which is necessary to avoid spherical symmetry in the electron density, while the integration of the BZ was performed using only the Γ-point due to the lack of dispersion in the electronic states. The equilibrium structures were obtained using a force convergence threshold of 0.025 eV Å^–1^ on each atom and a global energy convergence criterion of 10^–5^ eV. Additional details on the selected PAW projectors, computational parameters, and convergence tests are provided in the Supporting Information file.

### 2.2 Selecting Functional Ionic Substituents

We selected ionic species for chemical substitutions that preserved the stoichiometric charge of the unit cells, i.e., the electron counting rule was based on the oxidation state of the chemical species. In addition, we limited our selection of tetroxides to species that maintained the same number of atoms when replacing the PO_4_^3–^ groups, preventing significant structural modifications. Based on these criteria, we selected the following species: *X* = Mg^2+^, Sr^2+^, Ba^2+^, Zn^2+^, and Cd^2+^ to replace Ca^2+^ sites; *Y* = As and V to replace phosphorus at PO_4_^3–^ sites; and *Z* = F^–^, Cl^–^, and Br^–^ to replace OH^–^ sites. This selection resulted in 72 structures corresponding to the Ca/P ratio of 1.67, together with 18 structures for each phase that exhibit varying Ca/P molar ratios. In total, we considered a set of 144 structures for the purpose of conducting equilibrium optimizations through stress-tensor calculations.

### 2.3 Structure Configurations

The initial configuration of the hexagonal HAP bulk unit cell, with a Ca/P molar ratio of 1.67, was obtained from experimental measurements.^37^ To maintain the exact stoichiometry, we reduced the space group from *P*6_3_/*m* to *P*6_3_. This approach has been successfully applied in previous computational studies.^38,39^ Substituted HAP-based structures were created by replacing all atoms at the specific sites of Ca^2+^, PO_4_^3–^, and OH^–^ in the pure *P*6_3_ HAP structure, resulting in mono-, di-, and trisubstituted materials.

For the remaining apatite phases with different ratios of Ca/P, Figure 1, we maintained the space group consistent with the experimental characterization. Initial configurations for Ca/P ratios of 0.50, 1.00, 1.50, and 2.00 were based on the structures of Ca­(H_2_PO_4_)_2_,^24^ CaHPO_4_,^25^ γ-Ca_3_(PO_4_)_2_,^26^ and Ca_4_(PO_4_)_2_O,^27^ respectively. Since these phases lack hydroxyl sites, our evaluation of apatite-based materials only considered substitutions at the Ca^2+^ and PO_4_^3–^ sites.

**Figure 1:**
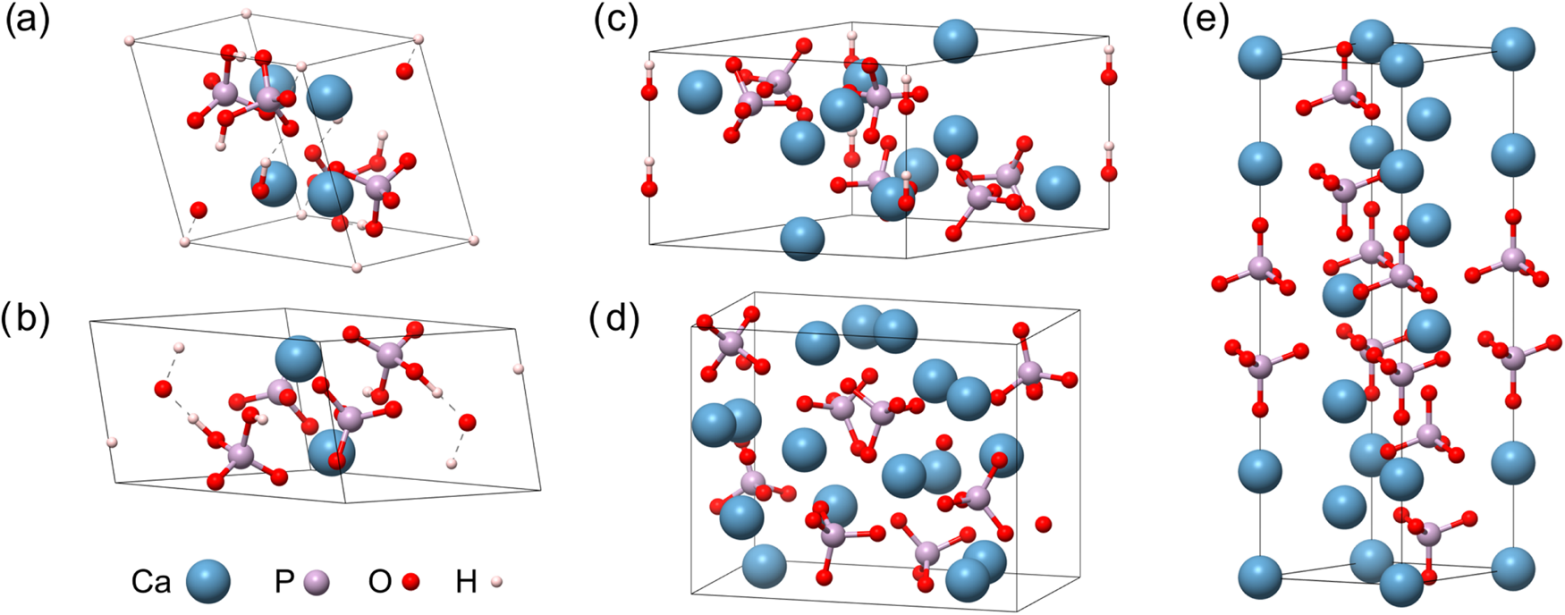
Unit cell representation of pristine (nonsubstituted) phases for the apatite-based materials: (a) CaHPO_4_, (b) Ca­(H_2_PO_4_)_2_, (c) Ca_10_(PO_4_)_6_(OH)_2_, (d) Ca_4_(PO_4_)_2_O, and (e) γ-Ca_3_(PO_4_)_2_. Light blue, pink, red, and white spheres represent Ca, P, O, and H atoms, respectively.

## 3 Results and Discussion

### 3.1 Pristine Apatite-Based Materials

In this section, we will summarize the most important findings related to the pristine apatite phases and compare our results with experimental data.

#### 3.1.1 Structure Features and Parameters

The structural properties of the pristine (nonsubstituted) phases are summarized in Table 1, where we also present the structural characteristics of the calcium phosphate compounds examined in this study. HAP and γ-tricalcium phosphate both crystallize in hexagonal unit cells, although with different space groups and the number of formula units per cell. HAP belongs to the *P*6_3_ space group with two formula units per cell, while γ-tricalcium phosphate adopts the *R*3̅*m* space group with three formula units per unit cell. Monocalcium phosphate and dicalcium phosphate anhydrous crystallize in triclinic unit cells within the *P*1̅ space group. However, they differ in the number of formula units per cell: Ca­(H_2_PO_4_)_2_ contains two, while CaHPO_4_ accommodates four. Tetracalcium diphosphate monoxide stands out as the only compound in this study with a monoclinic unit cell, crystallizing in the *P*2_1_ space group with four formula units per cell. Based on our analysis, the primary structural characteristics of these phases can be summarized as follows:

1. **Hydroxyapatite:** Contains two distinct Ca cation sites. Ca­(I) comprises four atoms arranged in two columns on opposite sides of the unit cell, with two cations in each column. Ca­(II) consists of six atoms arranged in two parallel equilateral triangles. The PO_4_^3–^ groups form two equilateral triangles coplanar with the Ca­(II) triangles but with larger edges. Hydroxyl groups align vertically in the center of both Ca­(II) and PO_4_^3–^ triangles, slightly offset from their planes.^40^
2. **Monocalcium phosphate:** The PO_4_ sites are aligned parallel to the [010] direction. All calcium atoms are crystallographically equivalent, each coordinated to eight oxygen atoms and organized in layers parallel to the direction [100], bonded by five different hydrogen bonds.
3. **Dicalcium phosphate anhydrous:** Features two types of Ca cations. Ca­(I) is coordinated with seven oxygen atoms in a pentagonal bipyramidal arrangement, while Ca­(II) is coordinated with eight oxygen atoms. Distorted Ca-PO_4_ chains align in planes parallel to the [010] direction, a feature shared with other calcium phosphates such as Ca­(H_2_PO_4_)_2_·H_2_O.^36^
4. γ**-Tricalcium phosphate:** Contains two types of Ca atoms arranged in planes parallel to the [001] direction. Ca­(I) occupies 3̅*m* symmetry sites and coordinates with 12 oxygen atoms, while Ca­(II) coordinates with ten. The structure can be described as a linear sequence of parallel PO_4_ – Ca­(II) – Ca­(I) – Ca­(II) – PO_4_ chains repeating along the *b*_0_ direction.^26^
5. **Tetracalcium diphosphate monoxide:** Contains eight crystallographically distinct Ca atoms. Ca­(I) through Ca­(V), along with Ca­(VII) and Ca­(VIII), each coordinates with seven oxygen atoms, while Ca­(VI) coordinates with eight. The phosphate groups are also crystallographically distinct, with each oxygen in all phosphate groups coordinating to three Ca cations. This phase uniquely contains oxide anions strongly coordinated to Ca atoms, which were not substituted in this study due to their absence in the other phases examined.

Table 1 presents a comparison between our calculated equilibrium lattice parameters and experimental results for various Ca/P ratios. The analysis reveals distinct patterns across different phases: (i) For Ca/P = 1.67, 1.50, and 2.00, these phases exhibited slight expansions in all lattice parameters compared to experimental values. The most significant deviation was observed in the HAP (Ca/P = 1.67) structure, where the *a*_0_ = *b*_0_ vectors expanded by 1.38%. (ii) For the Ca/P = 0.50 phase, the lattice parameter *a*_0_ exhibited the most significant deviation, expanding by 18.92% compared to experimental results. This pronounced discrepancy is consistent with findings from the Materials Project database,^41^ which also references the same initial apatite structure and reports a similar deviation from the experimental data. However, when comparing our calculated value *a*_0_ with the theoretical results from the Materials Project database, the deviation decreases to 7.28%. This smaller difference highlights the impact of methodological variations between the two studies. In contrast to *a*_0_, the lattice parameter *b*_0_ expanded by only 1.21%, and the parameter *c*_0_ remained unchanged. (iii) For Ca/P = 1.00, the relaxed structure for this phase demonstrated excellent agreement with the experimental values, with minimal deviations observed.

**Table 1:** DFT Results Obtained for the Structural, Energetic, and Electronic Properties of the Pristine (Nonsubstituted) Phases for the Apatite-Based Materials: Ca/P Molar Ratios, Lattice Parameters (*a*_0_, *b*_0_, *c*_0_), Cohesive Energy (*E*_coh_), and Fundamental Energy Bandgap at the *Γ*-Point (*E*_g_)­^a^ ^24,26,27,36,37^

			*a*_0_	*b*_0_	*c*_0_	*E*_coh_	*E*_g_^Γ^
apatite systems	space group	Ca/P	(Å)	(Å)	(Å)	(eV)	(eV)
Ca(H_2_PO_4_)_2_	*P*1̅	0.50	8.99 (7.56)	8.15 (8.25)	5.55 (5.55)	–4.74	5.16
CaHPO_4_	*P*1̅	1.00	6.71 (6.72)	6.98 (6.98)	7.09 (7.10)	–5.24	5.44
γ-Ca_3_(PO_4_)_2_	*R*3̅*m*	1.50	5.31 (5.25)	5.31 (5.25)	18.77 (18.67)	–5.71	5.53
Ca_10_(PO_4_)_6_(OH)_2_	*P*6_3_	1.67	9.55 (9.42)	9.55 (9.42)	6.89 (6.87)	–5.63	5.28
Ca_4_(PO_4_)_2_O	*P*2_1_	2.00	7.06 (7.02)	12.07 (11.99)	9.54 (9.47)	–5.72	4.54

a Experimental results^24,26,27,36,37^ are presented within parentheses.

These variations in the lattice parameters for different ratios Ca/P suggest that the computational approach may have varying degrees of effectiveness in capturing the structural nuances of each phase. In general, with the exception of the phase Ca/P = 0.50, our calculations provided a reasonably accurate representation of the experimental lattice parameters, lending confidence to the structural basis of our subsequent analyzes.

#### 3.1.2 Stability and Electronic Properties

The cohesive energy (*E*_coh_), which is calculated as the energy difference between the total energy (*E*_tot_^bulk^) and the cumulative sum of the total energies of the free atoms (*E*_tot_^*i*^). Consequently, it can be expressed by the following equation,

1 $$E_{\mathrm{coh}}=\frac{\left(E_{\mathrm{tot}}^{\mathrm{bulk}}-\sum_{i}N^{i}E_{\mathrm{tot}}^{i}\right)}{N_{\mathrm{tot}}}$$

where *N* ^*i*^ is the number of atoms of chemical element *i* and *N*_tot_ is the total number of atoms in the bulk unit cell. Thus, based on its definition, *E*_coh_ is a key stability indicator, measuring the energy required to split a solid into free atoms. Its magnitude reflects the bonding strength, and higher values indicate strong atomic interactions and improved stability, durability, and decomposition resistance.

As shown in Table 1, Ca_4_(PO_4_)_2_O and γ-Ca_3_(PO_4_)_2_ are the most stable pure apatite materials, with *E*_coh_ values of −5.72 and −5.71 eV, respectively. In contrast, the Ca/P = 0.50 phase (Ca­(H_2_PO_4_)_2_) exhibits the lowest stability, with *E*_coh_ = −4.74 *eV*, which is 20.67% higher than that of Ca_4_(PO_4_)_2_O. These results suggest that the higher Ca/P ratios generally correspond to more stable apatite structures.

The fundamental energy bandgap at the Γ-point (*E*_g_, in eV), was evaluated using the following equation,

2 $$E_{g}=E_{\mathrm{CBM}}^{\Gamma }-E_{\mathrm{VBM}}^{\Gamma }$$

where *E*_CBM_^Γ^ and *E*_VBM_^Γ^ are the energies of the conduction band minimum and valence band maximum at the Γ-point, respectively. Therefore, this analysis facilitates the determination of whether the material exhibits conductive, semiconductive, or insulating behavior, which is important to drive its application.

According to the results summarized in Table 1, all pristine apatites exhibit insulating characteristics, as evidenced by their high values of *E*_g_. The γ-tricalcium phosphate phase (Ca/P = 1.50) has the largest band gap, which is 21.81% higher than the lowest value *E*_g_ observed in the structure Ca/P = 2.00. Our calculated *E*_g_ values are generally consistent with other theoretical investigations available in the Materials Project library.^42^ The largest observed deviation was approximately 8.67% for the Ca/P = 1.00 phase. In general, the high band gap energies confirm the insulating nature of the studied apatite materials, with the γ-tricalcium phosphate phase exhibiting the most pronounced insulating character.

### 3.2 Effects of Ionic Substitution in Apatite-Based Materials

In this section, we present a comprehensive analysis of the impacts resulting from ionic substitution on the energetic, electronic, and structural properties.

#### 3.2.1 Equilibrium Lattice Parameters

The lattice parameter variations (Δ*a*_0_, Δ*b*_0_, and Δ*c*_0_) were calculated as percentage changes relative to the pristine apatite structures using the following equation

3 $$\Delta x_{0}=100\frac{\left(x_{0}^{i}-x_{0}^{\mathrm{ref}}\right)}{x_{0}^{\mathrm{ref}}}$$

where *x* = *a*, *b*, *c*, *x*_0_^*i*^ represents the equilibrium lattice constant of the substituted apatite-like material, and *x*_0_^ref^ is the equilibrium lattice of the pristine apatite.

As shown in Figure 2, the variation of the lattice parameters and the corresponding cell volume (*V*_cell_) fluctuate in response to variations in the radii of the cationic and anionic substitution species, which is consistent with size effects. Larger cations (e.g., Sr^2+^, Ba^2+^) and anions (e.g., AsO_4_^3–^, VO_4_^3–^) lead to an expansion of *V*_cell_, while smaller species (e.g., Mg^2+^, Zn^2+^, PO_4_^3–^) generally result in volume contraction.

**Figure 2:**
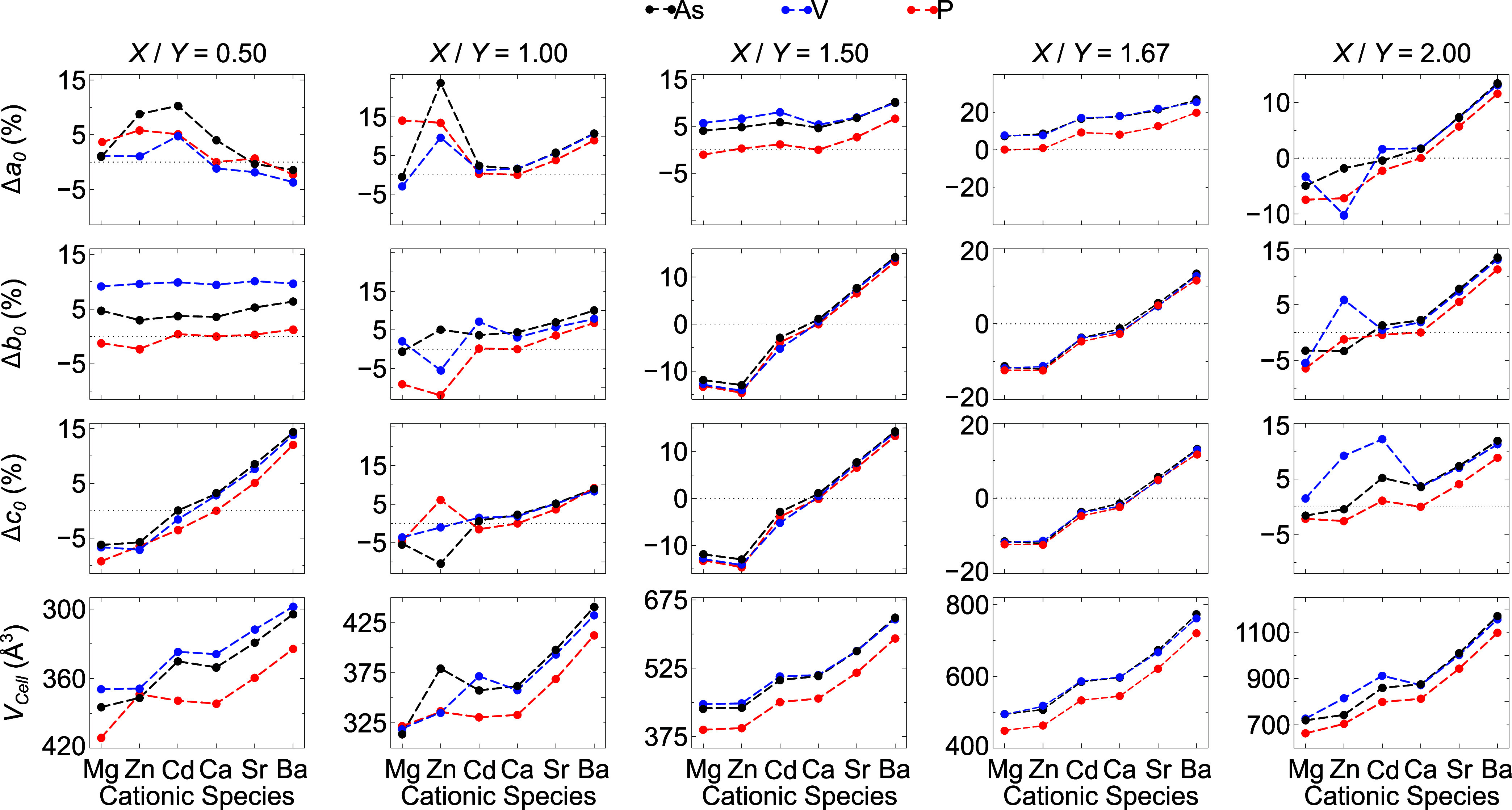
Structural properties of substituted apatite-like materials: variation of lattice parameters (Δ*a*_0_, Δ*b*_0_, and Δ*c*_0_) calculated as percentage changes relative to the pure apatite structure and volume of the unit cell (*V*_cell_).

Interestingly, the hexagonal phases (Ca/P = 1.50 and 1.67) exhibit unique behavior: the *a*_0_ parameter does not contract with any substitutional species, even when the smallest cations are introduced, while *b*_0_ = *c*_0_ prove to be more flexible. For the Ca/P = 0.50 phase, the smaller cations (e.g., Mg^2+^, Zn^2+^, Cd^2+^) increase the *a*_0_ lattice parameter, whereas the larger cations have the opposite effect. Furthermore, phosphate species have a more pronounced influence on the *b*_0_ lattice parameter, while the *c*_0_ parameter behaves as expected for this phase.

#### 3.2.2 Effects of Ionic Substitution on the Structural Stability

Two notable trends are observed for all structures: the stability (cohesive energy analyzes) of apatite-based materials (i) decreased with substitutions by d-block elements such as Zn and Cd, and (ii) increased in order of AsO_4_ < PO_4_ < VO_4_ tetroxide substituents. Figure 3 shows that the presence of d-block elements in the apatite structure leads to a decrease in the fundamental energy band gap, which can be partially explained by the principle of maximum hardness.^43^ This principle suggests that materials with smaller *E*_g_ tend to exhibit fewer ionic interactions and higher reactivity.

**Figure 3:**
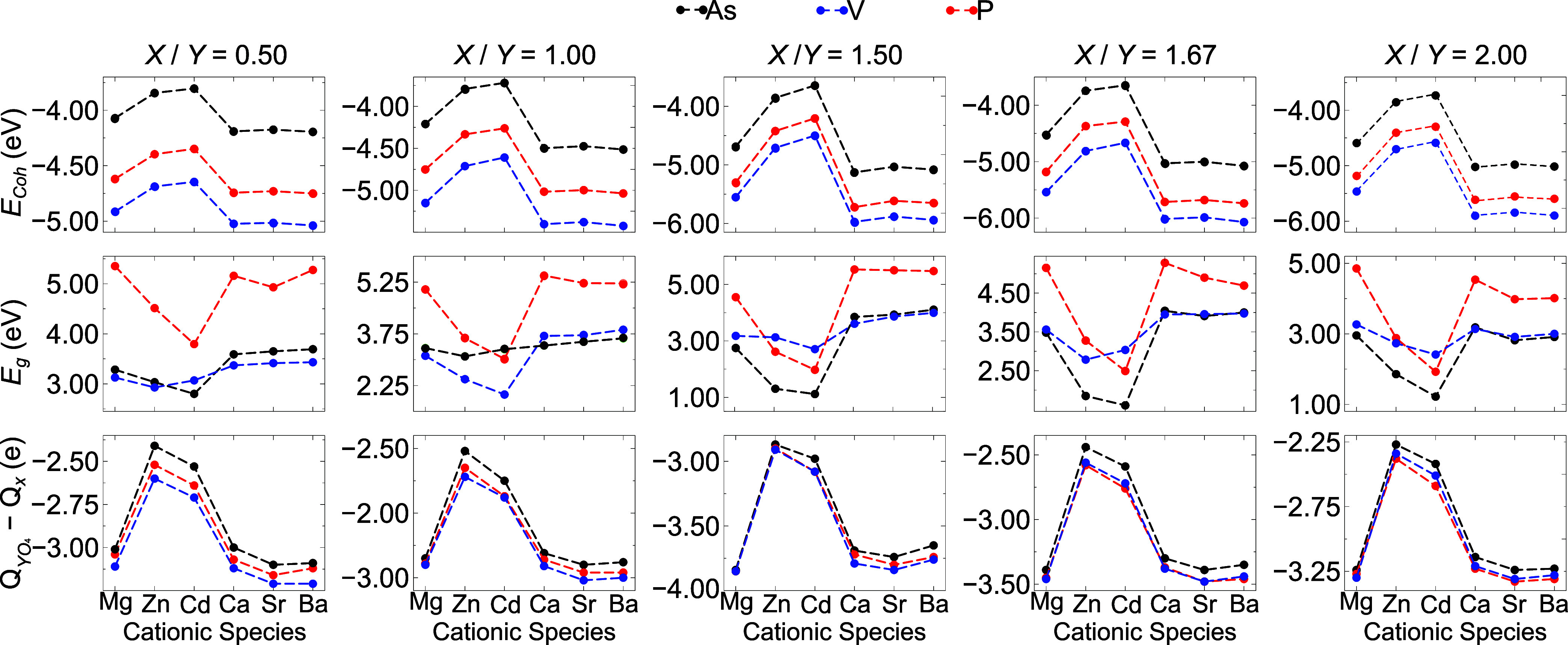
Energetic and electronic properties of substituted apatite-like materials: cohesive energy per atom (*E*_coh_), fundamental energy bandgap at the Γ-point (*E*_g_), and net atomic charge difference between anionic and cationic species within the bulk structure (*Q*_*Y*O_4__ – *Q*_*X*_).

Consequently, substitution with Zn and Cd reduced the ionic nature of the bulk structures. This effect can be observed by the difference between the average charge of *Y*O_4_ groups and the average charge of *X* cations (*Q*_*Y*O_4__ – *Q*_*X*_). The weaker ionic interactions induced by these d-block elements lead to reduced charge differences and weaker atomic bonds, resulting in decreased structural stability.

In contrast, substitutions with alkaline earth cations, such as Sr^2+^ and Ba^2+^ tend to restore stronger ionic interactions, thus increasing charge differences and improving structural stability. This is due to the higher ionic character of alkaline earth cations compared to d-block elements, which promotes a more polarized bonding with oxygen atoms. The correlation between *E*_coh_ and *Q*_YO_4__ – *Q*_X_ indicates a strong relationship between the type of cationic substituent, the charge difference, and the structural stability of these materials. This trend has been further confirmed through a Spearman’s correlation analysis, as discussed in Section 4.

Furthermore, tetroxide substituents (VO_4_, PO_4_, AsO_4_) can influence structural stability in different ways. The stability order of AsO_4_ < PO_4_ < VO_4_ cannot be solely explained by the *E*_g_ values, as VO_4_-substituted bulks exhibit the highest stability despite not having the largest band gaps. To understand this behavior, we performed local DOS calculations. These calculations reveal significant d-p hybridization between the d-orbitals of the *Y* atoms (e.g., V, P, As) and the *p*-orbitals of the oxygen atoms. This hybridization, particularly pronounced for VO_4_, leads to a stronger covalent character in the bonds and contributes to the enhanced stability of these phases.^44,45^ Not surprisingly, the intensity of the d-p hybridization correlates well with the cohesive energy (*E*_coh_), highlighting the strong relationship between the bulk electronic structure and the stability of substituted apatite materials.

It is important to note that, in addition to thermodynamic stability, other factors play a crucial role in practical ion exchange for real-world applications. In particular, ion conductivity, ion mobility, and the kinetics of ion exchange can significantly influence the feasibility of synthesizing substituted apatites experimentally. These properties depend on diffusion rates within the apatite lattice, which can be impacted by ion migration barriers and structural distortions induced by substitutions. While our study primarily focuses on thermodynamic aspects, experimental research has demonstrated that the substituted apatites investigated here not only exhibit thermodynamic stability but also exhibit promising properties for practical applications across different fields.^46–50^

## 4 Insights into Energetic Stability from Spearman’s Correlation

To improve our understanding of the key factors that contribute to the stability of apatite-like materials, we performed a Spearman rank correlation analysis. Our analysis was carried out on all optimized bulk structures, both collectively and grouped by their composition *X/Y* ratios (e.g., 0.50, 1.00, 1.50, etc.). The primary objective was to identify the correlations among the structural, energetic, and electronic properties selected in this study. Figure 4 presents the results as a correlation matrix, where each cell represents the correlation coefficient (ρ_s_) between the cohesive energy (*E*_coh_) of a specific phase (rows) and the studied properties (columns). The strength of the correlation increases as ρ_s_ approaches 1 (indicating a direct correlation) or −1 (indicating an inverse correlation). The analysis yielded several critical insights into the determinants of structural stability:

- Charges: *Q*_*X*_, *Q*_O_, and *Q*_*Y*O_4__ – *Q*_*X*_ exhibited strong correlations with *E*_coh_, confirming the discussions in Section 3.2.1. The negative ρ_s_ for *Q*_*X*_ indicate that stability increases with more highly charged cations, while the opposite holds for *Q*_*O*_. Conversely, |ρ_s_|≤ 0.30 for *Q*_*Y*_ confirms that *Y* had minimal impact on the ionic nature.
- *E*_g_: This property can be used as a stability descriptor for *X/Y* ratios of 1.50, 1.67, and 2.00, as discussed in Section 3.2.1. However, its effect is less pronounced for the triclinic phases with *X/Y* ratios of 0.50 and 1.00.
- Structural properties: ECN*_Y_* (average effective number of *Y* substituents) and ECN_H_ (average effective number of H) showed the highest ρ_s_ among structural properties, particularly for *X/Y* ratios of 0.50 and 1.00. However, the standard deviations were lower than 0.1 for both properties and phases, indicating that substitutions by the chosen ions did not significantly affect these parameters. Therefore, the selected structural properties are poor descriptors of the cohesive energy of the selected apatite-like compounds.

**Figure 4:**
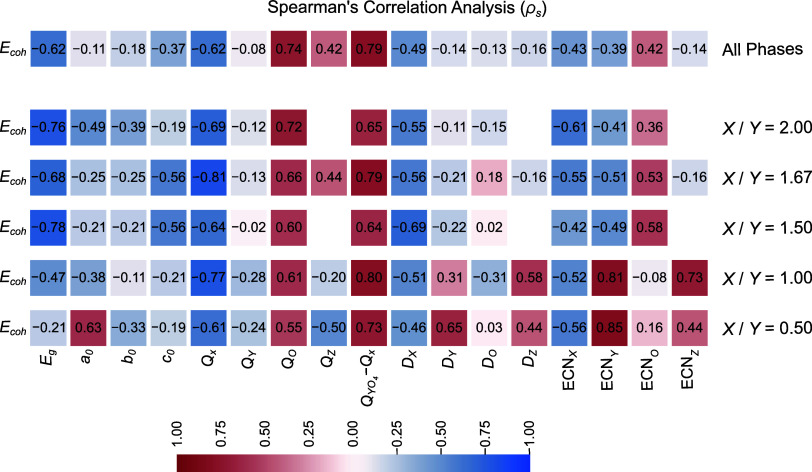
Spearman’s correlation analysis (ρ_s_) of the cohesive energy per atom (*E*_coh_) for the of substituted apatite-like materials in relation to the following properties: energy gap at the Γ-point (*E*_g_ in eV), length of lattice parameters (*a*_0_, *b*_0_, and *c*_0_ in Å), average net atomic charges (*Q*_*X*_, *Q*_*Y*_, *Q*_*O*_, and *Q*_*Z*_ in *e*), net atomic charge difference between anionic and cationic species (*Q*_*Y*O_4__ – *Q*_*X*_ in *e*), average distance for nearest neighbors (*D*_*X*_, *D*_*Y*_, *D*_*O*_, and *D*_*Z*_ in Å), and effective coordination number (ECN_*X*_, ECN_*Y*_, ECN_*O*_, and ECN_*Z*_ in NNN). Subscripts are defined as X = Ca, Mg, Sr, Ba, Zn, and Cd; *Y* = P, As, and V; and *Z* = H, F, Cl, and Br. Direct and inverse monotonic correlations are observed when ρ_s_ is closer to 1 and −1, respectively.

Thus, only 9.4% (42.7%) of the explored properties demonstrated strong correlations with |ρ_s_|> 0.75 (moderate correlations with |ρ_s_|> 0.50). The predominance of weak correlations, indicated by the fact that most absolute coefficients fall below 0.50, underscores the complex interplay of factors that affect the stability of the selected apatites. This complexity highlights the challenge of isolating definitive determinants of stability, suggesting that energetic stability results from a multifaceted combination of structural, chemical, and electronic contributions rather than being governed by a singular property.

## 5 Conclusions

In this investigation, we performed DFT-PBE calculations to characterize the physicochemical properties of apatite-like materials as a function of the Ca/P ratios, ranging from 0.50 to 2.00. Apatite-like materials show variations in space group, composition, size, and stability, and hence their physicochemical properties spread over a wide range of values. Our aim was to explore the impact of ionic substitutions at the cationic and anionic sites on the geometric, electronic, and energetic characteristics of these materials. Furthermore, we used Spearman correlation analysis to discern the primary factors (descriptors) that influence the stability and material properties.

Structurally, we identified a strong correlation between the ionic radii of the substituents and the unit cell volume. Substitutions with larger cations, such as Ba^2+^ and Sr^2+^, resulted in expansions of the volume of the unit cells, while smaller cations, such as Mg^2+^ and Cd^2+^, led to volume contractions. Stability analysis revealed that substitutions with Zn and Cd significantly reduced the magnitude of the cohesive energies, indicating decreased material stability. This decline in stability correlated with a narrowing of the fundamental band gap at the Γ-point and a reduction in ionic character, quantified by the charge difference between the net atomic charges of *Y*O_4_ groups and *X* cations (*Q*_*Y*O_4__ – *Q*_*X*_). Among the tetroxide substituents, VO_4_ was the most stable, followed by PO_4_ and AsO_4_. This trend is attributed to the extent of d-p hybridization between the d-orbitals of *Y* atoms and the p-orbitals of oxygen, which closely matched the cohesive energy order.

Finally, Spearman’s correlation analysis further highlighted that net atomic charges on the *X* and O species, along with the energy gap, have a significant influence on the cohesive energy. This analysis underscores the importance of electronic properties in determining material stability. Overall, our findings provide valuable insight into the impact of ionic substitutions on the stability of apatite-like materials, contributing to a deeper understanding of their properties.

## Supplementary Material



## Notes

Data used for the figures, as well as additional analyses and additional computational parameters used in the calculations (PDF)
