# Supplementary material for: Influence of Ion Substitution on the Properties of Apatite-Based Materials: Computational Predictions Using Density Functional Theory
Source: ACS Omega. 2025 May 24;10(22):22521–8. doi: 10.1021/acsomega.4c09997 (PMC12163767; doi:10.1021/acsomega.4c09997)
Supplement: Supplementary file 1 [file ao4c09997_si_001.pdf]

Supporting Information: Influence of Ion Substitution on the Properties of Apatite-Based Materials: Computational Predictions Using Density Functional Theory

Henrique S. Marques,*,† Albert F. B. Bittencourt,*,‡,¶ and Juarez L. F. Da Silva*,¶

†*Institute of Mathematics and Computer Sciences, University of São Paulo,* 13566-590*, São Carlos, SP, Brazil*

‡*Institute of Science and Technology, Federal University of Jequitinhonha and Mucuri Valleys,* 39100-000*, Diamantina, MG, Brazil*

¶*São Carlos Institute of Chemistry, University of São Paulo, P.O. Box* 780*,* 13560-970*, São Carlos, SP, Brazil*

E-mail: hesomarques@usp.br; albertbittenc@ict.ufvjm.edu.br; juarez_dasilva@iqsc.usp.br

Contents

S-1 Introduction **S-3**

S-2 Additional Details on the Selected PAW Projectors **S-3**

S-3 Computational Convergence Tests **S-4**

S-4 Additional Details on the Definition of the Structural, Energetic, and Electronic Properties **S-6**

S-5 Equilibrium Lattice Parameters, Cohesive Energy, and Energy Gap **S-8**

S-5.1 $Ca(H_2PO_4)_2$-based Materials S-8

S-5.2 $CaHPO_4$-based Materials S-10

S-5.3 γ-$Ca_3(PO_4)_2$-based Materials S-12

S-5.4 $Ca_{10}(PO_4)_6(OH)_2$-based Materials S-13

S-5.5 $Ca_4(PO_4)_2O$-based Materials S-16

S-6 Density of States, Effective Coordination Number and Effective Charge . **S-18**

S-6.1 $Ca(H_2PO_4)_2$-based Materials S-18

S-6.2 $CaHPO_4$-based Materials S-54

S-6.3 γ-$Ca_3(PO_4)_2$-based Materials S-90

S-6.4 $Ca_{10}(PO_4)_6(OH)_2$-based Materials S-126

S-6.5 $Ca_4(PO_4)_2O$-based Materials S-270

References **S-305**

S-1 Introduction

The supplementary information document provides detailed technical data to substantiate the findings and discussions articulated in the primary manuscript. It specifically comprises (*i*) technical particulars concerning the selection of PAW projectors and the execution of convergence tests, alongside (*ii*) supplementary analysis of the structural, energetic, and electronic characteristics of substituted apatite-like compounds.

S-2 Additional Details on the Selected PAW Projectors

As elucidated in the primary article, all computational analyzes were executed using the Vienna *Ab initio* Simulation Package (VASP), version 5.4.4.[1,2] Within this computational framework, the core-valence electron interactions are represented via the frozen core projector augmented wave (PAW) method,[3,4] while the Kohn–Sham (KS) states are delineated using plane wave basis sets. Table S-1 presents additional specifications regarding PAW projectors pertinent to each chemical species examined in this study.

Table S-1: Technical specifications for the PAW-PBE projectors used in this study. Recommended cutoff energy for the plane-wave basis set, `ENMAX` (eV); number of valence electrons, `ZVAL`; and valence electron configuration for each chemical element.

Element	PAW projector	`ENMAX`	`ZVAL`	Valence
H	H_GW 21Apr2008	300.000	1	$1s^1$
O	O_GW_new 19Mar2012	434.431	6	$2s^22p^4$
F	F_GW_new 19Mar2012	487.698	7	$2s^22p^5$
Cl	Cl_GW_new 19Mar2012	196.854	7	$3s^23p^5$
Br	Br_GW_new 21Nov2013	475.692	7	$4s^24p^5$
Mg	Mg_pv_GW 20Apr2010	403.929	8	$2p^63s^2$
P	P_GW 19Mar2012	255.040	5	$3s^23p^3$
Ca	Ca_sv_GW 31Mar2010	281.430	10	$3s^23p^63d^2$
V	V_sv_GW 05Dec2013	286.741	13	$3s^23p^63d^5$
Zn	Zn_GW 09Oct2010	328.191	12	$3d^{10}4s^2$
As	As_GW 20Mar2012	208.702	5	$4s^24p^3$
Sr	Sr_sv_GW 23Mar2010	224.817	10	$4s^24p^64d^2$
Cd	Cd_sv_GW 16Apr2014	361.806	20	$4s^25s^24p^64d^{10}$
Ba	Ba_sv_GW 23Mar2010	237.515	10	$5s^25p^65d^2$

S-3 Computational Convergence Tests

The convergence of the **k**-point density and the plane-wave cutoff energy (ENCUT) was evaluated for the $Ca_{10}(PO_4)_6(OH)_2$ bulk phase. Tables S-2 and S-3, along with Figure S-1, summarize these results. The optimized computational parameters derived from these tests were then employed in all calculations throughout this study.

The relative errors in the lattice parameters, expressed as percentages for Δa_0 and Δc_0, were determined by means of the subsequent equation:

$$\Delta a_0 = \frac{(a_0^i - a_0^{ref})}{a_0^{ref}} \times 100 \,, \tag{1}$$

in which a_0^i represents the equilibrium lattice constant determined for a specified set of computational parameters, and a_0^{ref} denotes a reference value (largest computational parameter) as specified in each table.

The graphs clearly show that increasing both ENCUT and **k**-point density reduces the relative error in the lattice parameters to nearly zero. However, we concluded that employing excessively high ENCUT and **k**-point density values is unnecessary, as it significantly increases computational cost without proportional gains in accuracy. Based on these results, we selected a plane-wave cutoff energy 50 % higher than the largest recommended value among all chemical elements in the unit cell, and a **k**-point density of 20 for bulk relaxation. To ensure consistency in the energetic analyses, we performed an additional calculation that allowed only changes in atomic positions, using a cutoff energy of 548 eV and the same **k**-point density.

Table S-2: Computational convergence tests as a function of the **k**-density using a cutoff energy of 868.862 eV (2.0×$\mathtt{ENMAX}_{\mathtt{max}}$). Number of **k**-points, $N_{\mathbf{k}}$; lattice parameters, a_0 and c_0; relative errors with respect to the highest **k**-density calculation, Δa_0 and Δc_0; energy gap at the Γ-point, E_g^Γ; total energy per unit cell, E_{tot}; and difference in total energy per unit cell with respect to the highest **k**-density calculation, ΔE_{tot}.

**k**-density	**k**-mesh	$N_{\mathbf{k}}$	a_0 (Å)	Δa_0 (%)	c_0 (Å)	Δc_0 (%)	E_g^Γ (eV)	E_{tot} (eV)	ΔE_{tot} (eV)
5	1 × 1 × 1	1	9.56	0.01	6.91	−0.19	5.29	−359.837 126 26	0.160 229 20
10	1 × 1 × 1	1	9.56	0.01	6.91	−0.19	5.29	−359.837 126 26	0.160 229 20
15	2 × 2 × 2	4	9.56	0.00	6.90	−0.03	5.34	−360.410 358 66	0.001 181 15
20	2 × 2 × 3	4	9.56	0.02	6.90	−0.02	5.33	−360.414 109 10	0.000 140 56
25	3 × 3 × 4	9	9.56	0.04	6.90	−0.05	5.33	−360.413 295 17	−0.000 366 39
30	4 × 4 × 4	12	9.56	0.00	6.90	0.04	5.33	−360.414 127 83	0.000 135 36
35	4 × 4 × 5	12	9.56	0.00	6.90	0.00	5.34	−360.414 615 70	0.000 000 00

Table S-3: Computational convergence tests as a function of the cutoff energy using a **k**-density of 20. Lattice parameters, a_0 and c_0; relative errors with respect to the highest `ENCUT` calculation, Δa_0 and Δc_0; energy gap at the Γ-point, E_g^Γ; total energy per unit cell, E_{tot}; and difference in total energy per unit cell with respect to the highest `ENCUT` calculation, ΔE_{tot}.

`ENCUT` (eV)	a_0 (Å)	Δa_0 (%)	c_0 (Å)	Δc_0 (%)	E_g^Γ (eV)	E_{tot} (eV)	ΔE_{tot} (eV)
434.43	9.32	2.50	6.82	1.11	5.54	−358.873 448 32	0.427 249 67
488.73	9.50	0.65	6.87	0.39	5.38	−360.040 383 37	0.103 472 77
543.04	9.55	0.09	6.90	0.04	5.35	−360.224 327 35	0.052 435 82
651.65	9.55	0.14	6.90	0.03	5.34	−360.240 426 07	0.047 969 08
760.25	9.54	0.17	6.90	0.06	5.36	−360.324 289 65	0.024 700 36
868.86	9.56	0.00	6.90	0.00	5.34	−360.413 313 02	0.000 000 00

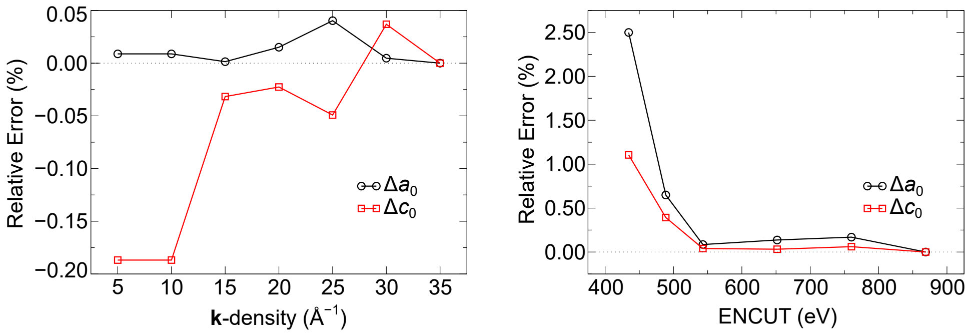

Figure S-1: Trends in convergence of lattice parameters for the $Ca_{10}(PO_4)_6(OH)_2$ bulk phase with respect to the **k**-density and the cutoff energy.

S-4 Additional Details on the Definition of the Structural, Energetic, and Electronic Properties

Lattice parameter variations (Δa_0, Δb_0, and Δc_0) are calculated as percentage changes relative to the pure apatite structure:

$$\Delta a_0(\%) = \frac{({a_0}^{i} - {a_0}^{ref})}{{a_0}^{ref}} \times 100 \,, \tag{2}$$

where ${a_0}^{i}$ is the equilibrium lattice constant of the substituted apatite-like material and ${a_0}^{ref}$ is the equilibrium lattice of the pure apatite.

The cohesive energy per atom (E_{coh}) is calculated to assess structural stability:

$$E_{coh} = \frac{E_{tot}^{bulk} - \sum_{1}^{N_{tot}} N^{i} E_{tot}^{i}}{N_{tot}} \,, \tag{3}$$

where E_{tot}^{bulk} is the total energy of the bulk unit cell (Tables S-5 to S-8), E_{tot}^{i} is the total energy of the free atom i (Table S-4), N^{i} is the number of atoms of chemical element i, and N_{tot} is the total number of atoms in the bulk unit cell. All energies for E_{coh} calculations employed a plane-wave cutoff energy of 548.660 eV.

The fundamental energy gap at the Γ-point (E_{g}^{Γ}, in eV) is evaluated to understand electronic properties:

$$E_{g}^{\Gamma} = E_{CBM}^{\Gamma} - E_{VBM}^{\Gamma} \,, \tag{4}$$

where E_{CBM}^{Γ} and E_{VBM}^{Γ} are the energies of the conduction band minimum and valence band maximum at the Γ-point, respectively.

Effective coordination number (ECN, in NNN) is calculated to characterize the local coordination of each atom within the structure:

$$\mathrm{ECN} = \sum_{i} \exp\left[1 - \left(\frac{d_{ij}}{d_{av}}\right)^{6}\right] \,, \tag{5}$$

where d_{ij} is the distance between atoms i and j, and d_{av} is the weighted average distance,

calculated by solving the non-linear equation:

$$d_{av} = \frac{\sum_j d_{ij} \exp\left[1 - \left(\frac{d_{ij}}{d_{av}}\right)^6\right]}{\sum_j \exp\left[1 - \left(\frac{d_{ij}}{d_{av}}\right)^6\right]}, \tag{6}$$

which is done using a self-consistent iterative procedure.[5,6]

Table S-4: Total energy and valence occupancy of all free atoms selected as constituents of the apatite-like materials.

Element	E_{tot} (eV)	Valence occupancy
H	−1.116 411 79	$1s^1$
O	−1.903 814 99	$2s^2 2p^4$
F	−0.689 838 46	$2s^2 2p^5$
Cl	−0.238 000 22	$3s^2 3p^5$
Br	−0.206 261 30	$4s^2 4p^5$
Mg	−0.006 771 57	$2p^6 3s^2$
P	−1.880 297 39	$3s^2 3p^3$
Ca	−4.935 926 86	$3s^2 3p^6 3d^2$
V	−5.602 357 64	$3s^2 3p^6 3d^5$
Zn	−0.007 756 04	$3d^{10} 4s^2$
As	−1.699 861 13	$4s^2 4p^3$
Sr	−4.747 600 50	$4s^2 4p^6 4d^2$
Cd	−0.054 345 97	$4s^2 5s^2 4p^6 4d^{10}$
Ba	−2.861 133 94	$5s^2 5p^6 5d^2$

S-5 Equilibrium Lattice Parameters, Cohesive Energy, and Energy Gap

S-5.1 $Ca(H_2PO_4)_2$-based Materials

Table S-5: Structural and energetic properties of $X(H_2YO_4)_2$ apatite-like materials with X/Y=0.50. Lattice parameters, a_0, b_0, and c_0; lattice parameter variations with respect to the $Ca(H_2PO_4)_2$ bulk phase, Δa_0, Δb_0, and Δc_0; unit cell volume, V_{cell}; total energy, E_{tot}; cohesive energy, E_{coh}; and energy gap at the Γ-point, E_g^{Γ}.

X	Y	a_0 (Å)	Δa_0 (%)	b_0 (Å)	Δb_0 (%)	c_0 (Å)	Δc_0 (%)	V_{cell} (Å^3)	E_{tot} (eV)	E_{coh} (eV)	E_g^{Γ} (eV)
Ba	V	8.66	−3.66	8.94	9.67	6.32	13.83	422.13	−218.697 883 19	−5.04	3.43
Ca	V	8.88	−1.16	8.92	9.46	5.71	2.83	381.09	−222.386 173 19	−5.02	3.37
Sr	V	8.82	−1.85	8.97	10.10	5.98	7.62	402.36	−221.754 829 44	−5.02	3.42
Mg	V	9.09	1.13	8.89	9.15	5.18	−6.68	350.82	−209.240 287 93	−4.91	3.13
Ba	P	8.78	−2.29	8.25	1.26	6.22	12.06	385.62	−195.140 453 30	−4.75	5.28
Ca	P	8.99	0.00	8.15	0.00	5.55	0.00	338.36	−199.090 703 21	−4.74	5.16
Sr	P	9.05	0.68	8.17	0.32	5.84	5.09	360.58	−198.310 129 62	−4.73	4.93
Zn	V	9.08	1.05	8.93	9.61	5.16	−7.11	351.34	−202.441 216 34	−4.69	2.93
Cd	V	9.41	4.75	8.96	9.90	5.46	−1.58	383.07	−201.282 411 99	−4.65	3.07
Mg	P	9.31	3.59	8.05	−1.23	5.04	−9.23	308.81	−185.501 009 13	−4.62	5.35
Zn	P	9.51	5.82	7.96	−2.28	5.19	−6.48	346.43	−178.823 925 35	−4.40	4.51
Cd	P	9.44	5.08	8.18	0.42	5.36	−3.50	340.82	−177.451 893 12	−4.35	3.80
Ba	As	8.86	−1.45	8.67	6.38	6.35	14.30	415.59	−177.743 412 98	−4.19	3.69
Ca	As	9.35	4.01	8.44	3.57	5.72	3.10	369.70	−181.770 830 37	−4.19	3.59
Sr	As	8.96	−0.34	8.58	5.28	6.02	8.50	390.96	−180.923 994 61	−4.17	3.65
Mg	As	9.07	0.94	8.53	4.71	5.21	−6.26	335.31	−168.325 527 26	−4.07	3.28
Zn	As	9.78	8.79	8.39	2.98	5.23	−5.77	343.35	−161.515 303 57	−3.84	3.03
Cd	As	9.91	10.29	8.45	3.73	5.55	0.04	374.84	−160.402 453 10	−3.80	2.80

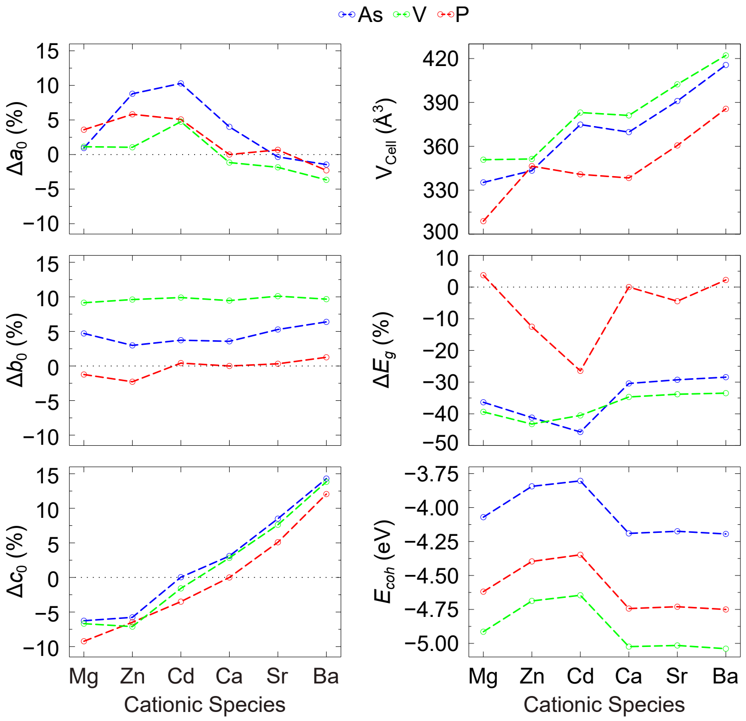

Figure S-2: Trends in structural and energetic properties of $X(H_2YO_4)_2$ apatite-like materials with X/Y=0.50. Table S-5 presents the detailed values for the data points shown in the plots.

S-5.2 $CaHPO_4$-based Materials

Table S-6: Structural and energetic properties of $XHYO_4$ apatite-like materials with X/Y=1.00. Lattice parameters, a_0, b_0, and c_0; lattice parameter variations with respect to the $CaHPO_4$ bulk phase, Δa_0, Δb_0, and Δc_0; unit cell volume, V_{cell}; total energy, E_{tot}; cohesive energy, E_{coh}; and energy gap at the Γ-point, E_g^Γ.

X	Y	a_0 (Å)	Δa_0 (%)	b_0 (Å)	Δb_0 (%)	c_0 (Å)	Δc_0 (%)	V_{cell} (Å^3)	E_{tot} (eV)	E_{coh} (eV)	E_g^Γ (eV)
Ba	V	7.28	8.55	7.83	12.24	7.82	10.20	429.63	−224.600 814 80	−5.57	3.62
Ca	V	6.96	3.79	7.17	2.80	7.33	3.39	353.30	−232.235 463 73	−5.54	3.41
Sr	V	7.15	6.63	7.47	7.08	7.58	6.83	390.17	−230.816 314 56	−5.52	3.52
Mg	V	6.89	2.76	6.90	−1.09	6.95	−2.08	318.44	−205.162 554 83	−5.28	3.34
Ba	P	7.13	6.24	7.64	9.49	7.61	7.32	396.51	−201.017 465 88	−5.25	5.21
Ca	P	6.71	0.00	6.98	0.00	7.09	0.00	319.76	−209.019 642 32	−5.24	5.44
Sr	P	6.93	3.22	7.27	4.19	7.34	3.46	355.26	−207.503 821 24	−5.22	5.22
Mg	P	6.41	−4.42	7.11	1.95	6.61	−6.76	291.84	−181.058 872 18	−4.95	5.04
Zn	V	6.45	−3.86	7.73	10.85	7.04	−0.75	334.26	−192.065 687 09	−4.81	3.10
Cd	V	7.22	7.59	7.18	2.95	7.31	3.01	368.10	−189.416 447 15	−4.71	3.30
Ba	As	7.41	10.41	7.80	11.76	7.84	10.49	433.75	−183.259 012 59	−4.65	3.87
Ca	As	6.99	4.24	7.15	2.52	7.31	3.09	351.95	−191.132 847 09	−4.63	3.69
Sr	As	7.21	7.42	7.45	6.85	7.57	6.74	390.83	−189.654 095 31	−4.60	3.71
Zn	P	5.85	−12.84	7.80	11.78	7.38	4.07	319.87	−167.562 383 78	−4.47	3.62
Cd	P	6.65	−0.89	6.93	−0.73	7.05	−0.70	312.40	−165.115 299 95	−4.37	3.02
Mg	As	6.85	2.10	6.86	−1.66	6.86	−3.27	312.14	−163.642 459 64	−4.35	3.11
Zn	As	6.05	−9.89	7.99	14.46	7.56	6.58	347.51	−150.171 772 54	−3.87	2.43
Cd	As	6.92	3.12	7.10	1.83	7.24	2.04	343.23	−147.723 733 92	−3.78	1.99

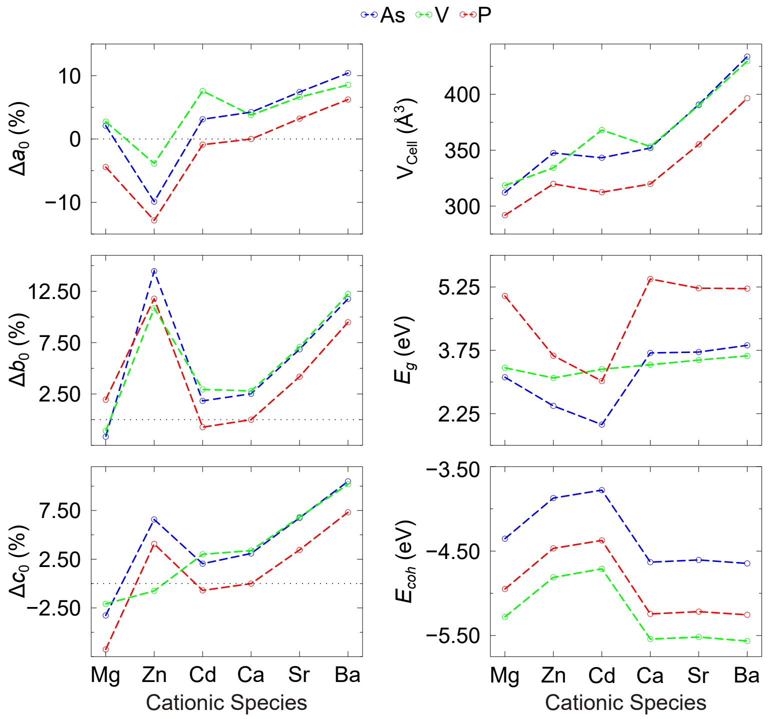

Figure S-3: Trends in structural and energetic properties of $XHYO_4$ apatite-like materials with X/Y=1.00. Table S-6 presents the detailed values for the data points shown in the plots.

S-5.3 γ-$Ca_3(PO_4)_2$-based Materials

Table S-7: Structural and energetic properties of γ-$X_3(YO_4)_2$ apatite-like materials with X/Y=1.50. Lattice parameters, $a_0 = b_0$, and c_0; lattice parameter variations with respect to the γ-$Ca_3(PO_4)_2$ bulk phase, Δa_0, Δb_0, and Δc_0; unit cell volume, V_{cell}; total energy, E_{tot}; cohesive energy, E_{coh}; and energy gap at the Γ-point, E_g^Γ.

X	Y	a_0 (Å)	Δa_0 (%)	c_0 (Å)	Δc_0 (%)	V_{cell} (Å^3)	E_{tot} (eV)	E_{coh} (eV)	E_g^Γ (eV)
Ba	V	5.84	10.01	21.40	14.00	632.43	−341.798 878 82	−6.07	3.99
Ca	V	5.59	5.30	18.83	0.32	509.94	−358.302 784 78	−6.01	3.61
Sr	V	5.68	6.89	20.17	7.46	562.88	−355.491 314 27	−5.99	3.86
Ba	P	5.66	6.63	21.26	13.26	590.32	−306.503 689 81	−5.74	5.48
Ca	P	5.31	0.00	18.77	0.00	458.43	−324.234 924 68	−5.71	5.53
Sr	P	5.45	2.70	20.00	6.54	515.11	−321.171 250 90	−5.68	5.50
Mg	V	5.61	5.71	16.35	−12.90	446.20	−295.486 707 42	−5.54	3.18
Mg	P	5.26	−1.03	16.30	−13.16	389.95	−259.064 293 23	−5.18	4.55
Ba	As	5.85	10.18	21.45	14.27	635.93	−279.537 760 67	−5.07	4.11
Ca	As	5.56	4.70	18.95	0.97	507.45	−296.457 555 21	−5.03	3.85
Sr	As	5.67	6.76	20.22	7.70	562.76	−293.671 718 26	−5.00	3.93
Zn	V	5.66	6.66	16.11	−14.16	447.71	−267.007 329 65	−4.81	3.13
Cd	V	5.74	8.01	17.79	−5.21	506.93	−261.733 468 44	−4.67	2.71
Mg	As	5.53	4.05	16.54	−11.90	437.24	−232.404 991 58	−4.52	2.75
Zn	P	5.32	0.27	16.03	−14.62	393.54	−227.432 623 84	−4.37	2.62
Cd	P	5.37	1.15	18.04	−3.88	450.80	−224.754 260 92	−4.29	1.97
Zn	As	5.57	4.80	16.34	−12.97	438.23	−201.919 649 19	−3.74	1.30
Cd	As	5.62	5.89	18.23	−2.89	499.18	−198.591 667 84	−3.65	1.12

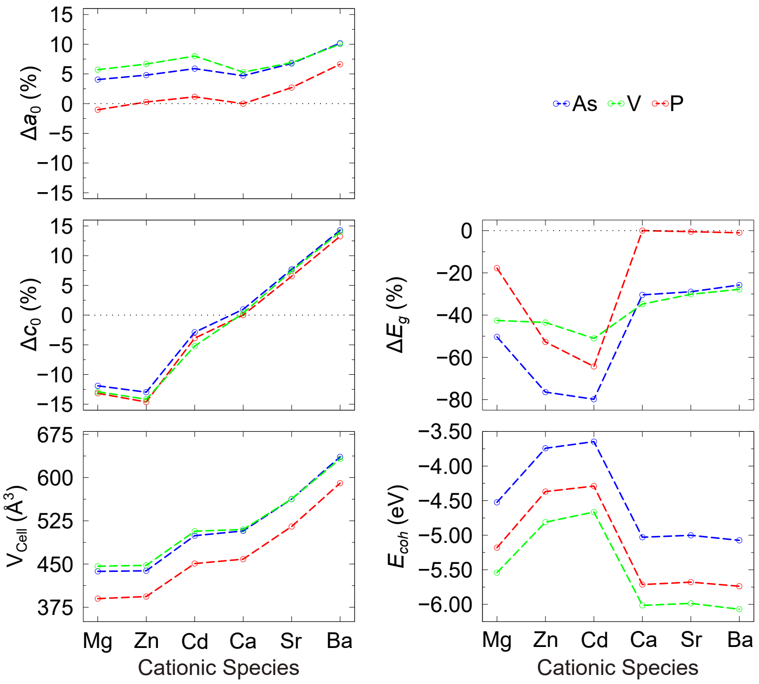

Figure S-4: Trends in structural and energetic properties of γ-$X_3(YO_4)_2$ apatite-like materials with X/Y=1.50. Table S-7 presents the detailed values for the data points shown in the plots.

S-5.4 $Ca_{10}(PO_4)_6(OH)_2$-based Materials

Table S-8: Structural and energetic properties of $X_{10}(YO_4)_6Z_2$ apatite-like materials with X/Y=1.67. Lattice parameters, $a_0 = b_0$, and c_0; lattice parameter variations with respect to the $Ca_{10}(PO_4)_6(OH)_2$ bulk phase, Δa_0, and Δc_0; unit cell volume, V_{cell}; total energy, E_{tot}; cohesive energy, E_{coh}; and energy gap at the Γ-point, E_g^Γ.

X	Y	Z	a_0 (Å)	Δa_0 (%)	c_0 (Å)	Δc_0 (%)	V_{cell} (Å^3)	E_{tot} (eV)	E_{coh} (eV)	E_g^Γ (eV)
Ba	V	F	10.53	10.26	7.90	14.54	758.14	−362.959 773 93	−6.04	4.00
Ca	V	F	9.93	3.94	6.91	0.19	589.35	−383.130 847 59	−6.03	3.97
Ba	V	Cl	10.68	11.82	7.77	12.69	767.22	−359.279 216 16	−5.97	3.94
Sr	V	F	10.11	5.89	7.46	8.24	660.79	−379.040 577 68	−5.97	3.97
Ca	V	Cl	10.34	8.29	6.76	−1.95	626.01	−379.155 115 03	−5.95	3.20
Ba	V	Br	10.76	12.68	7.78	12.86	780.26	−357.818 527 14	−5.94	3.90
Ca	V	Br	10.40	8.85	6.75	−2.13	631.35	−377.387 531 69	−5.91	2.41
Ba	V	OH	10.57	10.64	7.88	14.38	762.43	−373.439 314 57	−5.90	3.97
Sr	V	Cl	10.43	9.20	7.25	5.22	683.25	−374.870 201 99	−5.90	3.96
Ca	V	OH	10.02	4.92	6.87	−0.30	597.61	−394.100 497 09	−5.90	3.95
Sr	V	Br	10.58	10.80	7.26	4.82	700.63	−373.382 423 26	−5.86	3.70
Sr	V	OH	10.17	6.49	7.45	8.06	667.25	−389.687 037 14	−5.84	3.96
Ca	P	F	9.49	−0.68	6.90	0.15	537.91	−349.495 374 52	−5.76	5.60

Continued on next page

X	Y	Z	a_0 (Å)	Δa_0 (%)	c_0 (Å)	Δc_0 (%)	V_{cell} ($Å^3$)	E_{tot} (eV)	E_{coh} (eV)	E_g^Γ (eV)
Ba	P	F	10.28	7.63	7.79	13.01	712.91	−327.791 608 48	−5.73	5.21
Sr	P	F	9.83	2.96	7.35	6.68	615.77	−344.901 606 08	−5.69	5.26
Ba	P	Cl	10.40	8.85	7.70	11.75	720.95	−324.008 518 82	−5.67	5.17
Ca	P	Cl	9.86	3.29	6.75	−2.04	569.04	−344.641 843 06	−5.66	5.39
Ca	P	OH	9.55	0.00	6.89	0.00	544.51	−360.242 824 96	−5.63	5.28
Ba	P	Br	10.50	9.91	7.71	11.80	735.34	−322.368 868 45	−5.63	5.10
Ca	P	Br	9.95	4.15	6.72	−2.50	575.88	−342.775 391 59	−5.62	4.68
Sr	P	Cl	10.03	4.98	7.23	4.95	629.81	−340.458 268 80	−5.61	5.17
Ba	P	OH	10.33	8.14	7.79	12.96	719.34	−338.168 266 10	−5.60	4.69
Sr	P	Br	10.13	6.12	7.24	5.00	643.84	−338.687 388 50	−5.57	5.05
Sr	P	OH	9.89	3.56	7.34	6.53	622.14	−355.391 688 14	−5.57	4.90
Mg	V	F	9.96	4.24	5.86	−15.04	502.71	−314.155 726 59	−5.56	3.51
Mg	V	Cl	9.85	3.19	6.03	−12.50	507.29	−310.010 858 67	−5.48	1.58
Mg	V	OH	9.80	2.62	5.93	−13.92	493.64	−325.544 346 70	−5.46	3.56
Mg	V	Br	9.91	3.80	6.09	−11.72	517.96	−307.962 228 81	−5.43	0.22
Mg	P	F	9.36	−2.05	5.90	−14.45	446.98	−280.392 338 27	−5.29	5.18
Mg	P	Cl	9.48	−0.73	5.98	−13.21	465.74	−275.926 071 17	−5.20	3.46
Mg	P	OH	9.36	−1.95	5.91	−14.20	449.17	−291.823 728 92	−5.20	5.15
Mg	P	Br	9.55	−0.06	6.04	−12.31	476.92	−273.561 548 80	−5.15	1.92
Ca	As	F	9.91	3.78	6.94	0.64	590.21	−321.487 509 71	−5.12	4.10
Ba	As	F	10.57	10.67	7.92	14.85	765.88	−300.733 328 14	−5.12	4.15
Sr	As	F	10.14	6.13	7.48	8.49	665.32	−317.271 181 35	−5.06	3.99
Ba	As	Cl	10.73	12.40	7.78	12.91	776.69	−297.014 974 63	−5.05	4.11
Ca	As	Cl	10.32	8.02	6.81	−1.28	627.21	−317.453 201 02	−5.04	3.32
Ca	As	OH	9.98	4.48	6.93	0.47	597.17	−332.459 676 78	−5.03	4.04
Ba	As	OH	10.62	11.16	7.91	14.78	772.24	−311.224 489 92	−5.02	4.00
Ba	As	Br	10.82	13.28	7.79	13.04	789.87	−295.548 496 58	−5.02	4.01
Ca	As	Br	10.39	8.82	6.80	−1.34	636.12	−315.626 050 64	−5.00	2.37
Sr	As	Cl	10.42	9.10	7.31	6.10	687.61	−313.055 513 40	−4.98	3.95
Sr	As	OH	10.20	6.76	7.47	8.41	672.84	−327.945 379 87	−4.97	3.91
Sr	As	Br	10.55	10.42	7.28	5.60	701.11	−311.555 956 98	−4.95	3.68
Zn	V	F	10.14	6.17	5.82	−15.63	517.79	−280.515 028 53	−4.76	2.76
Zn	V	OH	10.11	5.84	5.83	−15.38	516.19	−292.484 853 02	−4.71	2.78
Zn	V	Cl	9.89	3.55	6.03	−12.52	510.73	−276.634 645 69	−4.69	1.56
Mg	As	F	9.86	3.23	5.89	−14.54	495.91	−253.211 882 69	−4.66	3.45
Zn	V	Br	9.92	3.82	6.11	−11.33	520.44	−275.011 716 07	−4.65	0.29
Cd	V	F	10.11	5.88	6.59	−4.38	583.74	−274.748 019 67	−4.61	3.08
Mg	As	OH	9.81	2.76	5.93	−14.03	494.38	−264.569 607 27	−4.60	3.48
Mg	As	Cl	9.85	3.11	6.03	−12.47	506.72	−248.856 749 66	−4.58	1.43
Cd	V	Cl	10.30	7.84	6.60	−4.30	606.08	−272.085 489 59	−4.57	2.63
Cd	V	OH	10.13	6.05	6.61	−4.17	586.88	−286.723 152 53	−4.56	3.03
Cd	V	Br	10.35	8.40	6.63	−3.78	615.72	−270.796 600 24	−4.54	1.64
Mg	As	Br	9.89	3.60	6.10	−11.47	517.43	−246.727 141 31	−4.53	1.77
Zn	P	F	9.57	0.17	5.82	−15.60	461.18	−244.851 872 67	−4.44	3.27
Zn	P	OH	9.56	0.07	5.84	−15.28	461.97	−256.825 645 48	−4.40	3.28
Zn	P	Cl	9.54	−0.08	5.96	−13.48	470.36	−241.095 153 76	−4.37	1.86

Continued on next page

X	Y	Z	a_0 (Å)	Δa_0 (%)	c_0 (Å)	Δc_0 (%)	V_{cell} ($Å^3$)	E_{tot} (eV)	E_{coh} (eV)	E_g^{Γ} (eV)
Zn	P	Br	9.57	0.25	6.05	−12.28	480.05	−239.220 701 89	−4.33	0.43
Cd	P	F	9.64	0.98	6.58	−4.51	530.27	−239.982 765 62	−4.31	2.52
Cd	P	OH	9.65	1.09	6.61	−4.13	533.48	−251.854 963 06	−4.28	2.49
Cd	P	Cl	9.91	3.81	6.55	−5.03	557.34	−236.842 175 18	−4.26	2.18
Cd	P	Br	9.99	4.57	6.57	−4.66	567.68	−235.335 229 31	−4.22	1.19
Zn	As	F	10.00	4.77	5.84	−15.26	506.48	−219.034 715 57	−3.85	1.80
Zn	As	OH	10.00	4.69	5.86	−15.00	507.29	−230.986 345 98	−3.84	1.85
Zn	As	Cl	9.93	3.97	6.00	−13.00	512.11	−215.140 797 61	−3.78	0.55
Zn	As	Br	9.96	4.25	6.07	−12.00	520.79	−213.490 431 32	−3.74	1.77
Cd	As	F	10.10	5.70	6.58	−4.58	580.53	−213.748 497 10	−3.71	1.62
Cd	As	OH	10.12	5.95	6.59	−4.35	584.64	−225.676 382 53	−3.71	1.61
Cd	As	Cl	10.29	7.71	6.59	−4.38	604.04	−210.980 977 52	−3.67	1.11
Cd	As	Br	10.34	8.27	6.64	−3.72	614.56	−209.636 425 33	−3.64	0.20

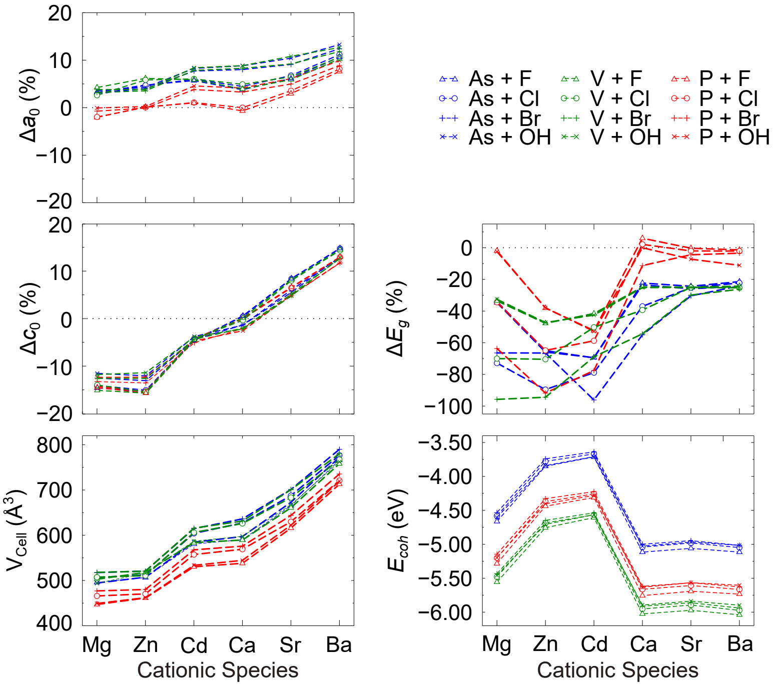

Figure S-5: Trends in structural and energetic properties of $X_{10}(YO_4)_6Z_2$ apatite-like materials with X/Y=1.67. Table S-8 presents the detailed values for the data points shown in the plots.

S-5.5 $Ca_4(PO_4)_2O$-based Materials

Table S-9: Structural and energetic properties of $X_4(YO_4)_2O$ apatite-like materials with X/Y=2.00. Lattice parameters, a_0, b_0, and c_0; lattice parameter variations with respect to the $Ca_4(PO_4)_2O$ bulk phase, Δa_0, Δb_0, and Δc_0; unit cell volume, V_{cell}; total energy, E_{tot}; cohesive energy, E_{coh}; and energy gap at the Γ-point, E_g^Γ.

X	Y	a_0 (Å)	Δa_0 (%)	b_0 (Å)	Δb_0 (%)	c_0 (Å)	Δc_0 (%)	V_{cell} (Å^3)	E_{tot} (eV)	E_{coh} (eV)	E_g^Γ (eV)
Ca	V	7.18	1.76	12.30	1.85	9.88	3.57	871.77	−550.739 589 16	−5.97	3.14
Ba	V	7.98	13.12	13.65	13.08	10.61	11.21	1155.94	−515.629 508 62	−5.94	3.00
Sr	V	7.57	7.26	12.96	7.35	10.21	7.01	1001.18	−542.234 438 38	−5.88	2.91
Ca	P	7.06	0.00	12.07	0.00	9.54	0.00	812.48	−505.855 624 50	−5.72	4.54
Ba	P	7.87	11.56	13.44	11.32	10.38	8.79	1097.80	−468.400 965 29	−5.65	4.01
Sr	P	7.46	5.68	12.74	5.50	9.92	4.04	942.62	−496.384 612 64	−5.61	3.98
Mg	V	6.82	−3.30	11.42	−5.44	9.68	1.52	727.14	−446.565 997 82	−5.55	3.27
Mg	P	6.53	−7.47	11.29	−6.45	9.33	−2.16	663.35	−401.758 625 09	−5.30	4.85
Ca	As	7.18	1.68	12.34	2.18	9.89	3.67	875.07	−468.790 489 08	−5.13	3.18
Ba	As	8.01	13.44	13.70	13.48	10.67	11.83	1169.66	−432.789 063 22	−5.08	2.91
Sr	As	7.58	7.38	13.01	7.73	10.24	7.32	1008.80	−460.038 220 31	−5.03	2.82
Zn	V	6.34	−10.19	12.78	5.87	10.41	9.15	814.95	−396.016 516 05	−4.71	2.73
Mg	As	6.71	−4.97	11.68	−3.25	9.39	−1.58	719.31	−363.556 810 70	−4.69	2.95
Cd	V	7.17	1.65	12.13	0.48	10.69	12.12	912.19	−384.194 279 79	−4.50	2.41
Zn	P	6.55	−7.19	11.93	−1.22	9.29	−2.56	703.89	−348.998 900 42	−4.42	2.88
Cd	P	6.90	−2.26	12.02	−0.41	9.64	1.06	798.93	−336.766 661 00	−4.21	1.93
Zn	As	6.93	−1.80	11.67	−3.35	9.50	−0.44	743.01	−313.585 319 00	−3.86	1.86
Cd	As	7.03	−0.43	12.23	1.29	10.03	5.17	860.22	−301.637 143 46	−3.64	1.22

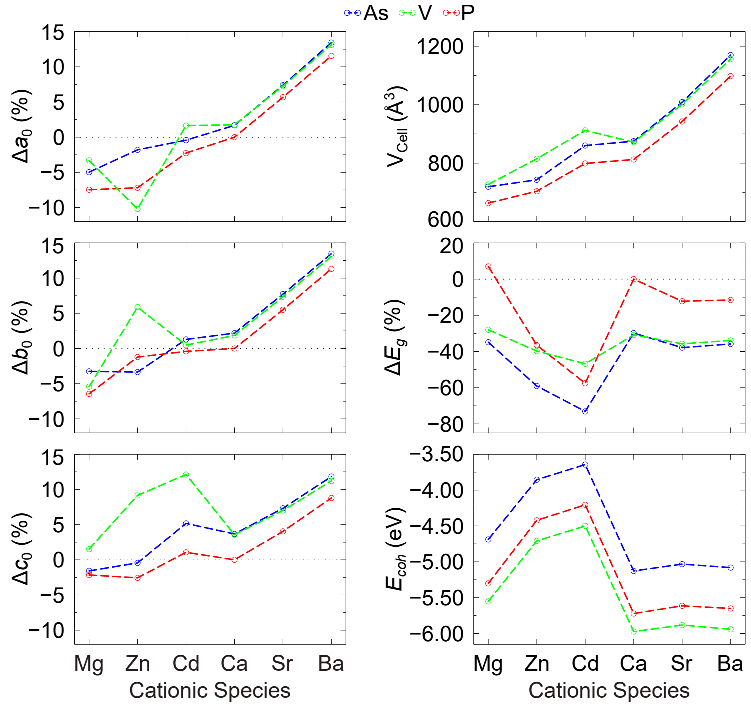

Figure S-6: Trends in structural and energetic properties of $X_4(YO_4)_2O$ apatite-like materials with X/Y=2.00. Table S-9 presents the detailed values for the data points shown in the plots.

S-6 Density of States, Effective Coordination Number and Effective Charge

S-6.1 $Ca(H_2PO_4)_2$-based Materials

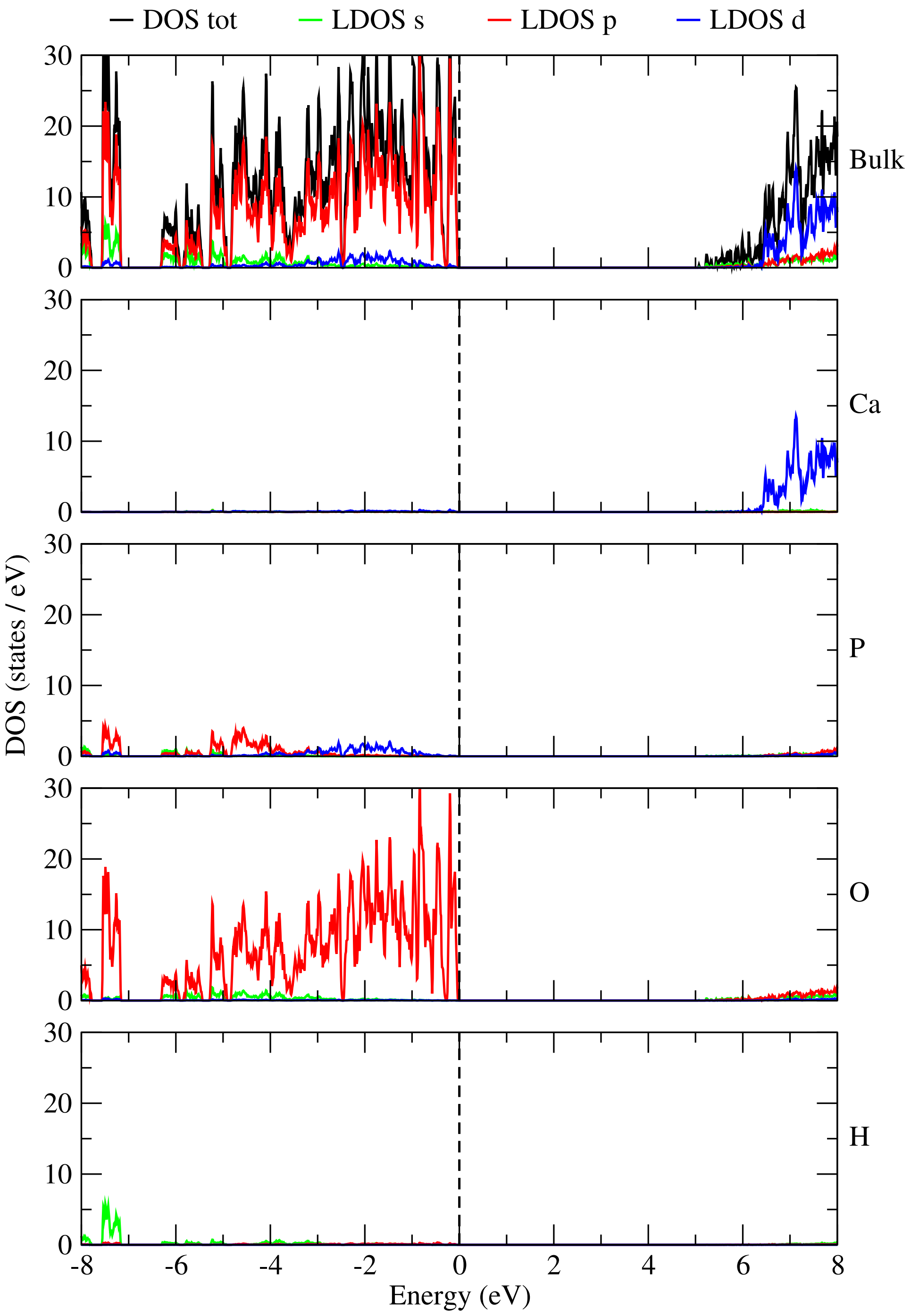

Figure S-7: Local density of states for the $Ca(H_2PO_4)_2$ bulk phase.

Table S-10: Calculated properties for the $Ca(H_2PO_4)_2$ bulk phase. Number of non-equivalent species, N; average distance for nearest neighbors, d_{NN}; effective coordination number, ECN; and net atomic charge, Q.

Non-equivalent species	N	d_{NN} (Å)	ECN (NNN)	Q (e^-)
Ca(I)	2	2.3360	6.6659	1.492 357
P(I)	2	1.5352	3.9344	1.539 132
P(II)	2	1.5203	3.9221	1.583 789
O(I)	2	1.5352	1.0001	−0.921 671
O(II)	2	1.5465	1.9918	−0.864 692
O(III)	2	1.4318	2.8961	−0.752 819
O(IV)	2	1.0264	1.0000	−0.754 192
O(V)	2	1.5203	1.0000	−0.887 324
O(VI)	2	1.0442	1.0001	−0.703 753
O(VII)	2	0.9798	1.0000	−0.714 662
O(VIII)	2	1.0605	1.0008	−0.676 209
H(I)	2	1.0605	1.0212	0.410 312
H(II)	2	1.0264	1.0000	0.412 077
H(III)	2	1.0442	1.0014	0.419 185
H(IV)	2	0.9798	1.0000	0.418 471

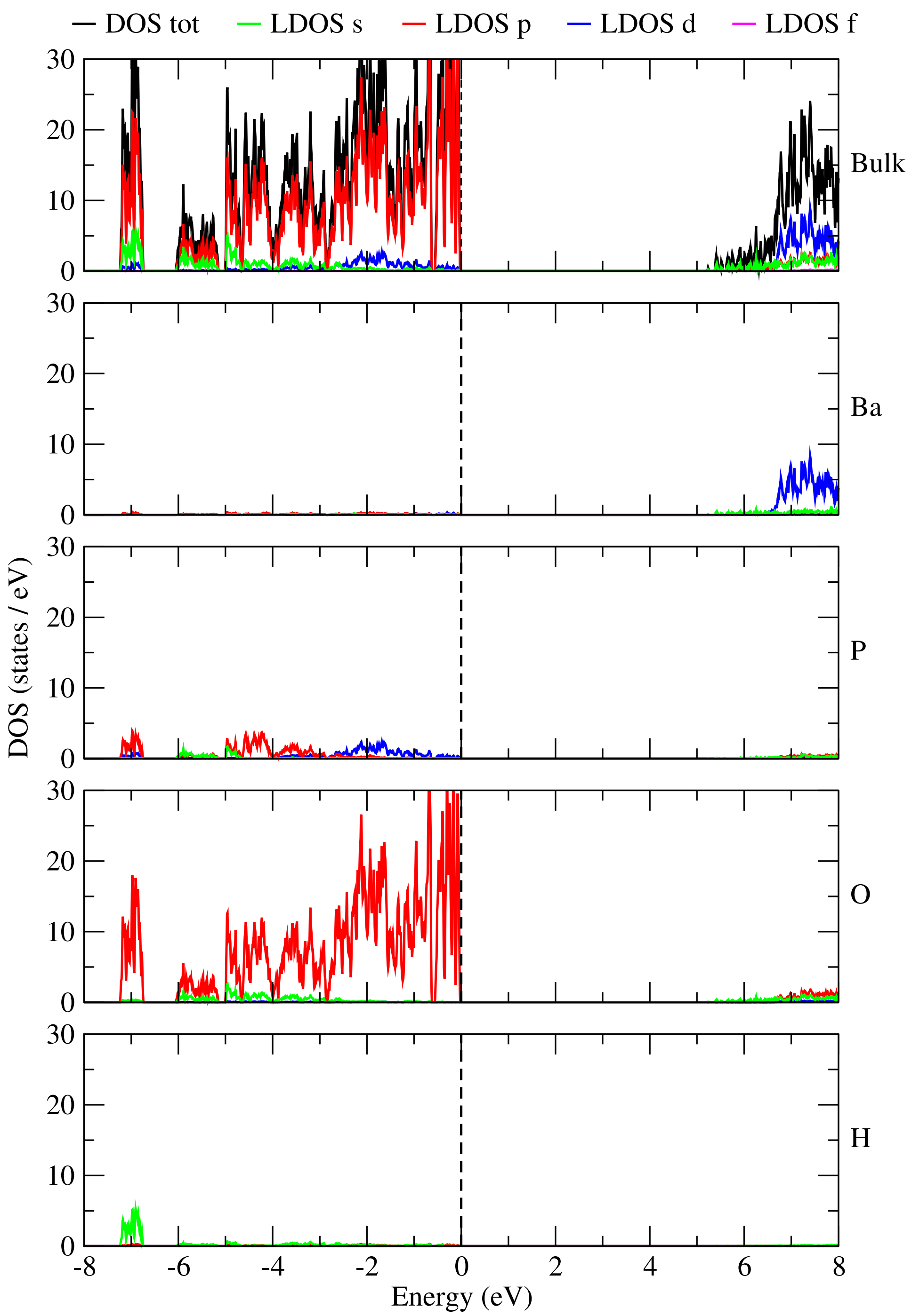

Figure S-8: Local density of states for the $Ba(H_2PO_4)_2$ bulk phase.

Table S-11: Calculated properties for the $Ba(H_2PO_4)_2$ bulk phase. Number of non-equivalent species, N; average distance for nearest neighbors, d_{NN}; effective coordination number, ECN; and net atomic charge, Q.

Non-equivalent species	N	d_{NN} (Å)	ECN (NNN)	Q (e^-)
Ba(I)	2	2.6376	8.6099	1.541 266
P(I)	2	1.5202	3.9328	1.539 046
P(II)	2	1.5031	3.8429	1.538 431
O(I)	2	1.5202	1.0000	−0.880 199
O(II)	2	1.5480	2.2467	−0.846 091
O(III)	2	1.1015	1.0018	−0.705 733
O(IV)	2	1.0182	1.0000	−0.741 102
O(V)	2	1.5254	1.0000	−0.892 289
O(VI)	2	1.0036	1.0000	−0.731 401
O(VII)	2	0.9938	1.0000	−0.707 325
O(VIII)	2	1.3309	1.7455	−0.737 127
H(I)	2	1.1015	1.3813	0.432 491
H(II)	2	1.0182	1.0000	0.406 731
H(III)	2	1.0036	1.0000	0.404 585
H(IV)	2	0.9938	1.0000	0.378 716

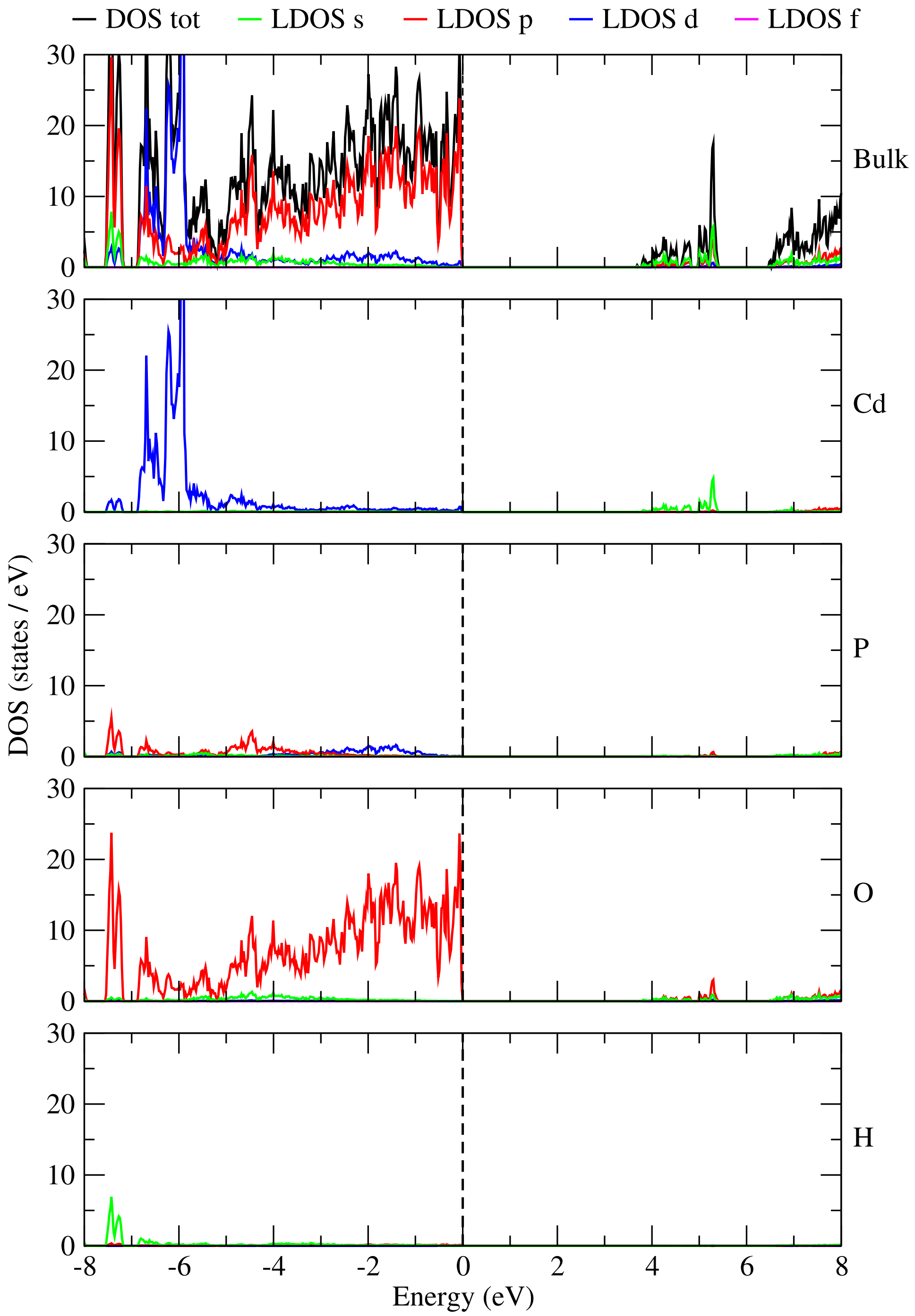

Figure S-9: Local density of states for the $Cd(H_2PO_4)_2$ bulk phase.

Table S-12: Calculated properties for the $Cd(H_2PO_4)_2$ bulk phase. Number of non-equivalent species, N; average distance for nearest neighbors, d_{NN}; effective coordination number, ECN; and net atomic charge, Q.

Non-equivalent species	N	d_{NN} (Å)	ECN (NNN)	Q (e^-)
Cd(I)	2	2.2131	5.7473	1.205 186
P(I)	2	1.5365	3.9497	1.534 325
P(II)	2	1.5269	3.9486	1.577 621
O(I)	2	1.5513	1.0007	−0.841 398
O(II)	2	1.5533	1.9539	−0.811 591
O(III)	2	1.4901	2.9843	−0.744 108
O(IV)	2	1.0166	1.0000	−0.705 030
O(V)	2	1.5269	1.0000	−0.833 263
O(VI)	2	1.0414	1.0001	−0.679 345
O(VII)	2	0.9926	1.0000	−0.697 673
O(VIII)	2	1.0440	1.0002	−0.660 786
H(I)	2	1.0440	1.0021	0.409 026
H(II)	2	1.0166	1.0000	0.409 317
H(III)	2	1.0414	1.0010	0.423 542
H(IV)	2	0.9926	1.0000	0.414 178

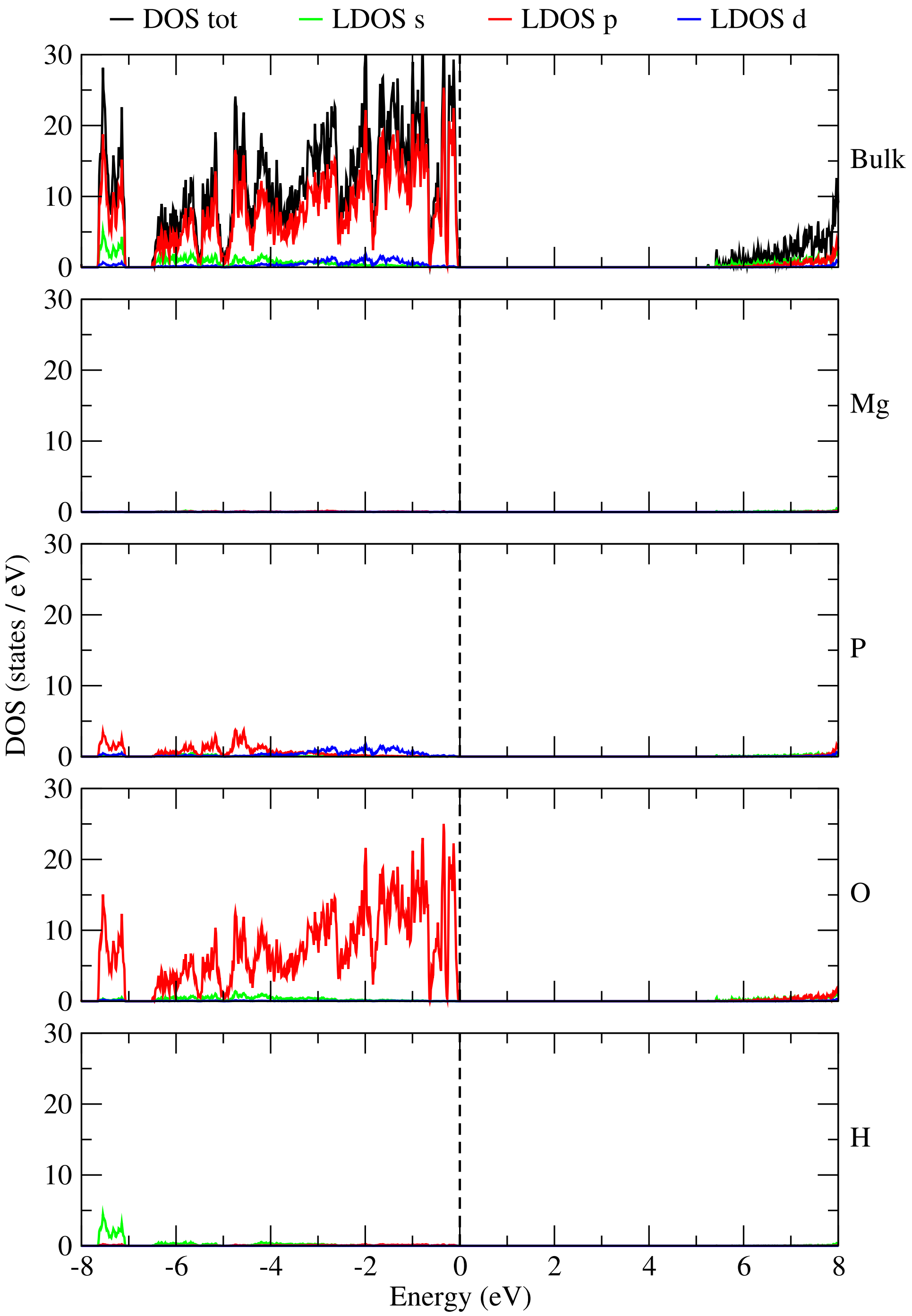

Figure S-10: Local density of states for the $Mg(H_2PO_4)_2$ bulk phase.

Table S-13: Calculated properties for the $Mg(H_2PO_4)_2$ bulk phase. Number of non-equivalent species, N; average distance for nearest neighbors, d_{NN}; effective coordination number, ECN; and net atomic charge, Q.

Non-equivalent species	N	d_{NN} (Å)	ECN (NNN)	Q (e^-)
Mg(I)	2	2.0383	5.7386	1.471 342
P(I)	2	1.5351	3.9453	1.586 387
P(II)	2	1.5313	3.9530	1.607 790
O(I)	2	1.5513	1.0527	−0.975 800
O(II)	2	1.5480	2.0109	−0.887 868
O(III)	2	1.4543	2.9282	−0.752 619
O(IV)	2	1.0147	1.0000	−0.711 467
O(V)	2	1.5313	1.0084	−0.928 998
O(VI)	2	1.0350	1.0001	−0.677 234
O(VII)	2	1.0026	1.0000	−0.727 921
O(VIII)	2	1.0536	1.0005	−0.665 183
H(I)	2	1.0536	1.0092	0.407 130
H(II)	2	1.0147	1.0000	0.410 954
H(III)	2	1.0350	1.0002	0.426 727
H(IV)	2	1.0026	1.0000	0.416 762

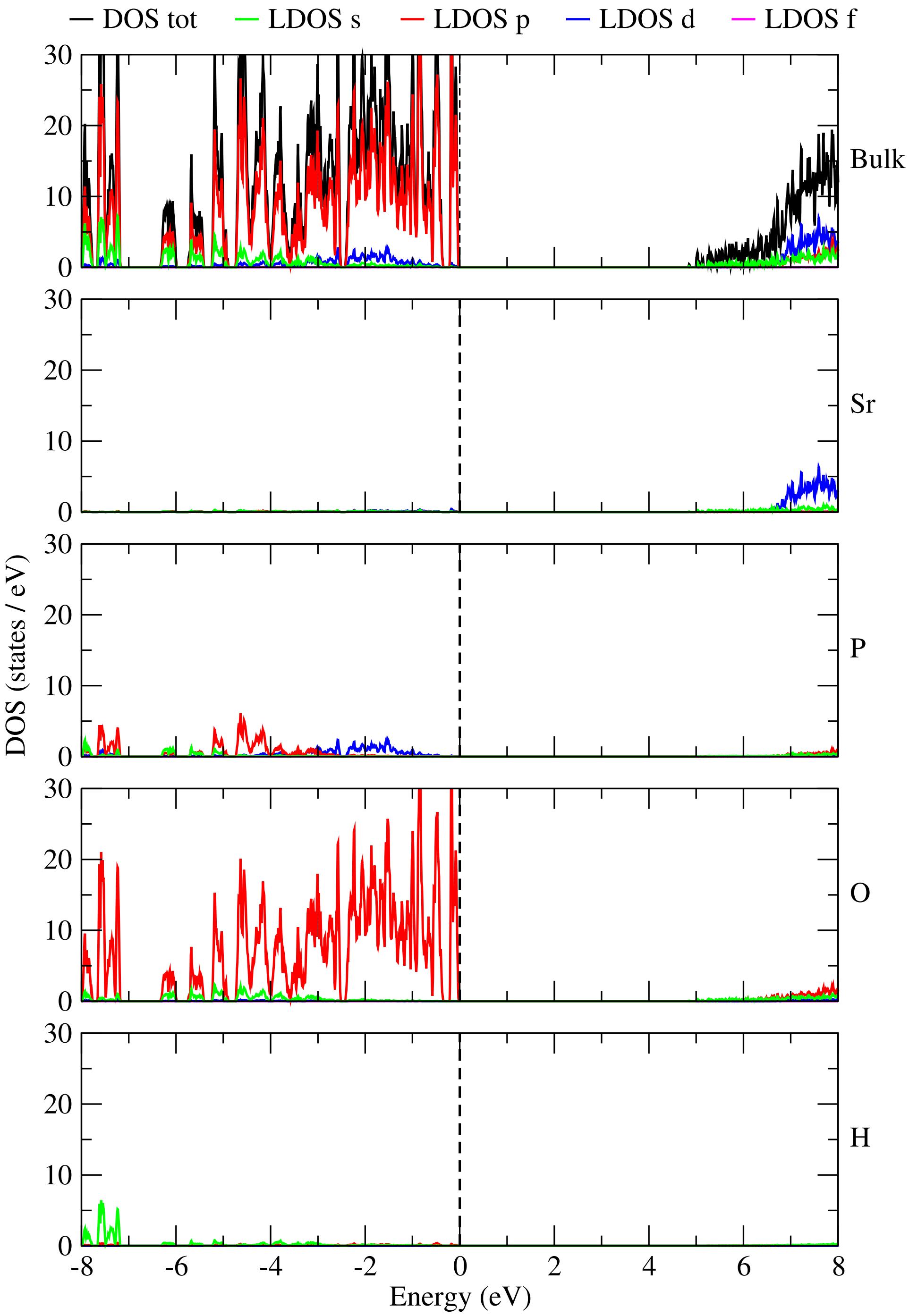

Figure S-11: Local density of states for the $Sr(H_2PO_4)_2$ bulk phase.

Table S-14: Calculated properties for the $Sr(H_2PO_4)_2$ bulk phase. Number of non-equivalent species, N; average distance for nearest neighbors, d_{NN}; effective coordination number, ECN; and net atomic charge, Q.

Non-equivalent species	N	d_{NN} (Å)	ECN (NNN)	Q (e^-)
Sr(I)	2	2.4977	6.9881	1.551 213
P(I)	2	1.5312	3.9317	1.535 637
P(II)	2	1.5160	3.9128	1.577 176
O(I)	2	1.5312	1.0000	−0.923 134
O(II)	2	1.5470	1.9998	−0.868 922
O(III)	2	1.4394	2.9015	−0.754 578
O(IV)	2	1.0307	1.0000	−0.767 530
O(V)	2	1.5160	1.0000	−0.892 713
O(VI)	2	1.0507	1.0002	−0.714 011
O(VII)	2	0.9787	1.0000	−0.723 656
O(VIII)	2	1.0609	1.0007	−0.676 999
H(I)	2	1.0609	1.0178	0.411 443
H(II)	2	1.0307	1.0001	0.411 693
H(III)	2	1.0507	1.0028	0.415 207
H(IV)	2	0.9787	1.0000	0.419 177

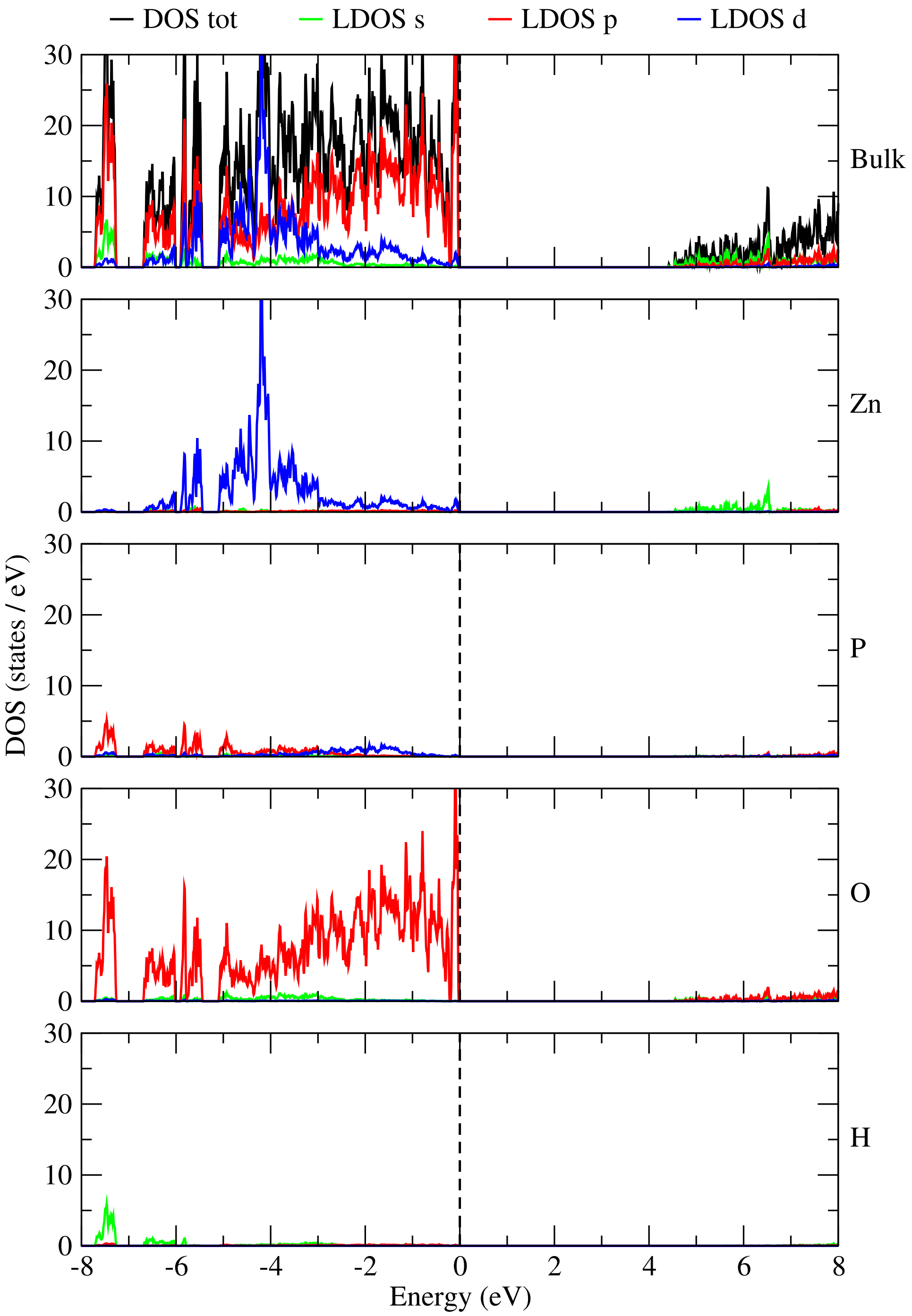

Figure S-12: Local density of states for the $Zn(H_2PO_4)_2$ bulk phase.

Table S-15: Calculated properties for the $Zn(H_2PO_4)_2$ bulk phase. Number of non-equivalent species, N; average distance for nearest neighbors, d_{NN}; effective coordination number, ECN; and net atomic charge, Q.

Non-equivalent species	N	d_{NN} (Å)	ECN (NNN)	Q (e^-)
Zn(I)	2	1.9047	3.8315	1.121 936
P(I)	2	1.5374	3.9553	1.523 547
P(II)	2	1.5481	3.9993	1.587 567
O(I)	2	1.5387	1.2467	−0.790 894
O(II)	2	1.5607	2.1389	−0.822 235
O(III)	2	1.4465	2.9140	−0.756 481
O(IV)	2	0.9997	1.0000	−0.693 767
O(V)	2	1.5553	1.0897	−0.815 904
O(VI)	2	1.0276	1.0001	−0.675 208
O(VII)	2	0.9811	1.0000	−0.667 670
O(VIII)	2	1.0590	1.0006	−0.683 202
H(I)	2	1.0590	1.0138	0.412 265
H(II)	2	0.9997	1.0000	0.404 997
H(III)	2	1.0276	1.0001	0.432 639
H(IV)	2	0.9811	1.0000	0.422 408

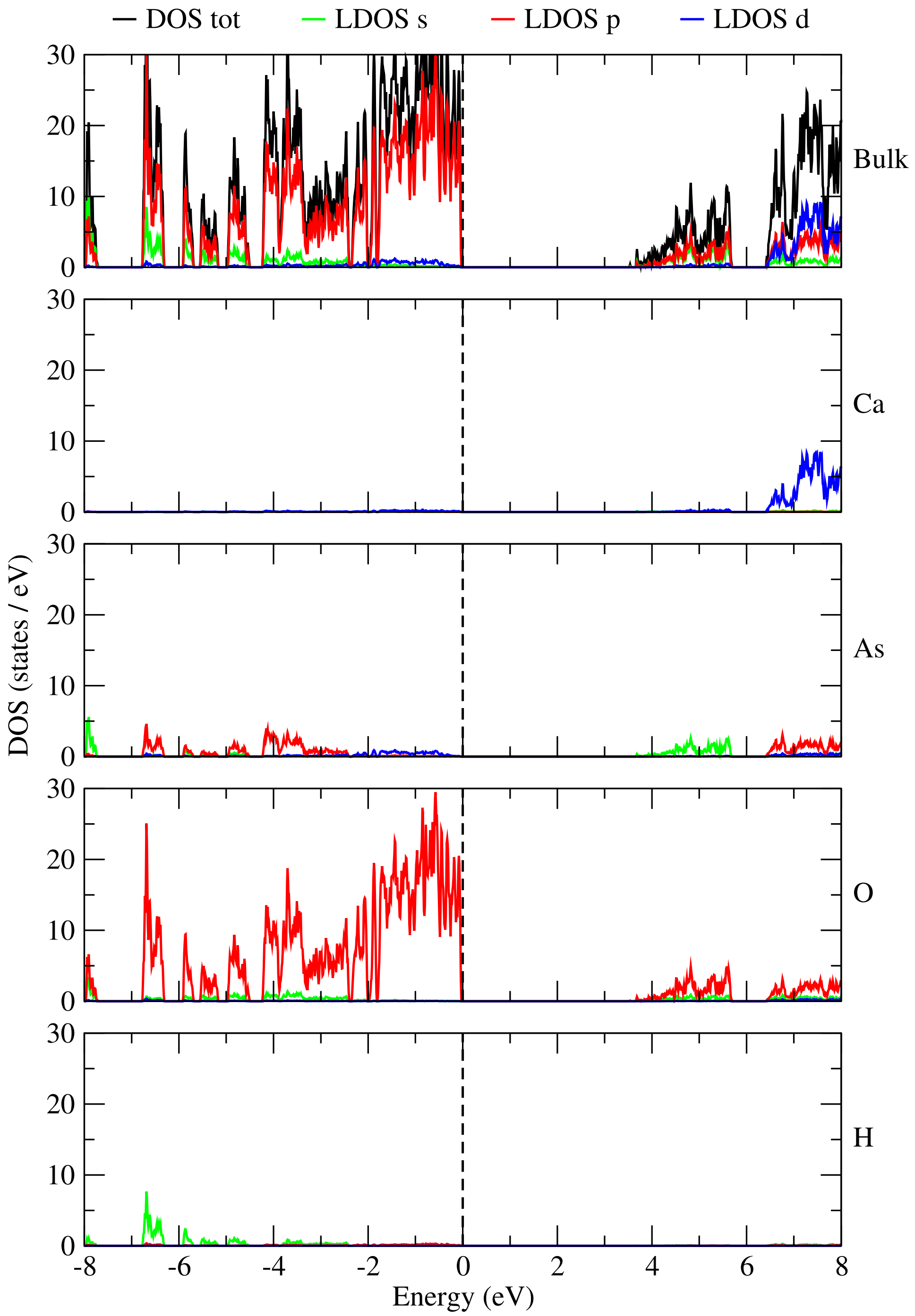

Figure S-13: Local density of states for the $Ca(H_2AsO_4)_2$ bulk phase.

Table S-16: Calculated properties for the $Ca(H_2AsO_4)_2$ bulk phase. Number of non-equivalent species, N; average distance for nearest neighbors, d_{NN}; effective coordination number, ECN; and net atomic charge, Q.

Non-equivalent species	N	d_{NN} (Å)	ECN (NNN)	Q (e^-)
Ca(I)	2	2.3268	6.7198	1.473 039
As(I)	2	1.6971	3.9629	1.688 955
As(II)	2	1.6812	3.9886	1.740 209
O(I)	2	1.6971	1.0161	−0.952 558
O(II)	2	1.5877	1.9182	−0.892 010
O(III)	2	1.4435	2.5206	−0.775 432
O(IV)	2	1.0265	1.0000	−0.774 938
O(V)	2	1.6812	1.0010	−0.912 668
O(VI)	2	1.0410	1.0000	−0.712 225
O(VII)	2	0.9813	1.0000	−0.742 587
O(VIII)	2	1.0650	1.0000	−0.713 277
H(I)	2	1.0650	1.0184	0.382 159
H(II)	2	1.0265	1.0000	0.395 879
H(III)	2	1.0410	1.0004	0.394 901
H(IV)	2	0.9813	1.0000	0.400 556

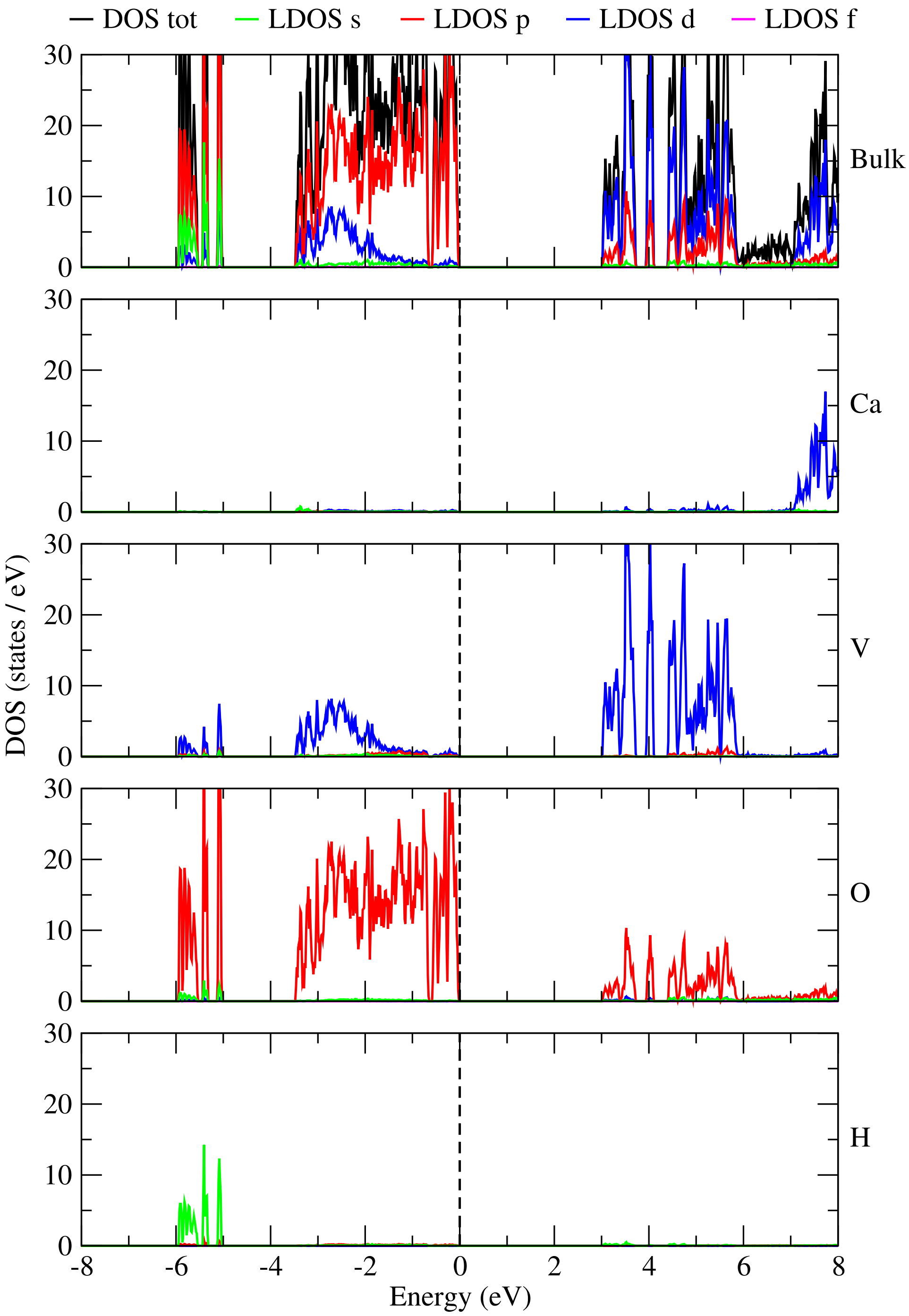

Figure S-14: Local density of states for the $Ca(H_2VO_4)_2$ bulk phase.

Table S-17: Calculated properties for the $Ca(H_2VO_4)_2$ bulk phase. Number of non-equivalent species, N; average distance for nearest neighbors, d_{NN}; effective coordination number, ECN; and net atomic charge, Q.

Non-equivalent species	N	d_{NN} (Å)	ECN (NNN)	Q (e^-)
Ca(I)	2	2.3840	7.0503	1.531 880
V(I)	2	1.6630	3.8345	2.000 364
V(II)	2	1.6254	3.6797	1.997 425
O(I)	2	1.6630	1.0015	−0.941 753
O(II)	2	1.6716	1.9762	−0.912 797
O(III)	2	1.0171	1.0000	−0.861 334
O(IV)	2	1.0025	1.0000	−0.895 029
O(V)	2	1.7018	1.0085	−1.035 508
O(VI)	2	1.0010	1.0000	−0.851 536
O(VII)	2	0.9753	1.0000	−0.934 205
O(VIII)	2	1.5821	1.9880	−0.747 636
H(I)	2	1.0171	1.0000	0.422 233
H(II)	2	1.0025	1.0000	0.413 733
H(III)	2	1.0010	1.0000	0.415 384
H(IV)	2	0.9753	1.0000	0.398 778

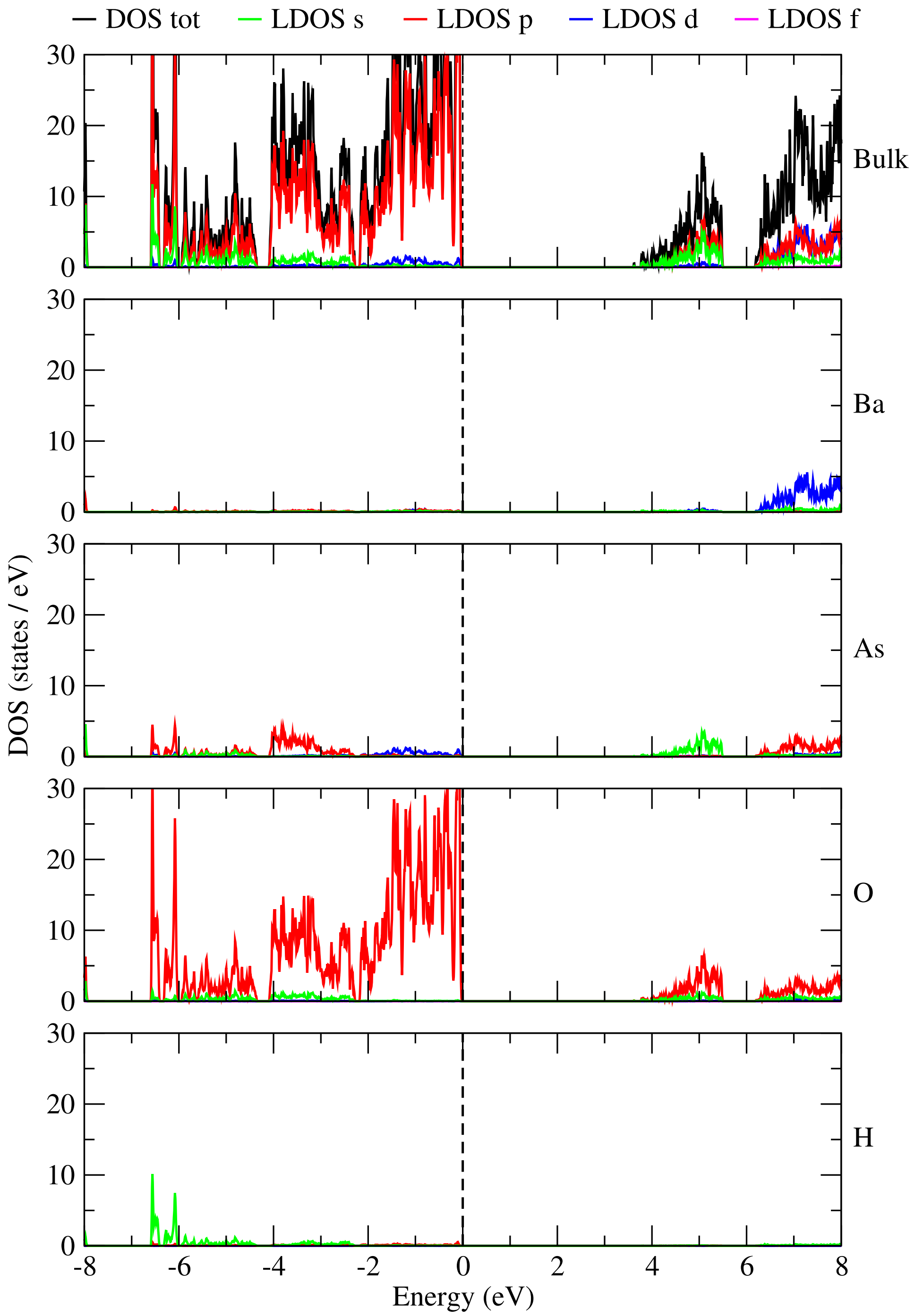

Figure S-15: Local density of states for the $Ba(H_2AsO_4)_2$ bulk phase.

Table S-18: Calculated properties for the $Ba(H_2AsO_4)_2$ bulk phase. Number of non-equivalent species, N; average distance for nearest neighbors, d_{NN}; effective coordination number, ECN; and net atomic charge, Q.

Non-equivalent species	N	d_{NN} (Å)	ECN (NNN)	Q (e^-)
Ba(I)	2	2.6724	7.1558	1.537 561
As(I)	2	1.6816	3.9705	1.694 117
As(II)	2	1.6639	3.8689	1.700 790
O(I)	2	1.6816	1.0000	−0.911 664
O(II)	2	1.5692	1.8987	−0.872 810
O(III)	2	1.0708	1.0000	−0.731 068
O(IV)	2	1.0288	1.0000	−0.778 700
O(V)	2	1.6844	1.0000	−0.917 281
O(VI)	2	1.0033	1.0000	−0.750 139
O(VII)	2	0.9778	1.0000	−0.772 582
O(VIII)	2	1.4227	1.5719	−0.772 366
H(I)	2	1.0708	1.0367	0.393 231
H(II)	2	1.0288	1.0000	0.394 963
H(III)	2	1.0033	1.0000	0.387 983
H(IV)	2	0.9778	1.0000	0.397 964

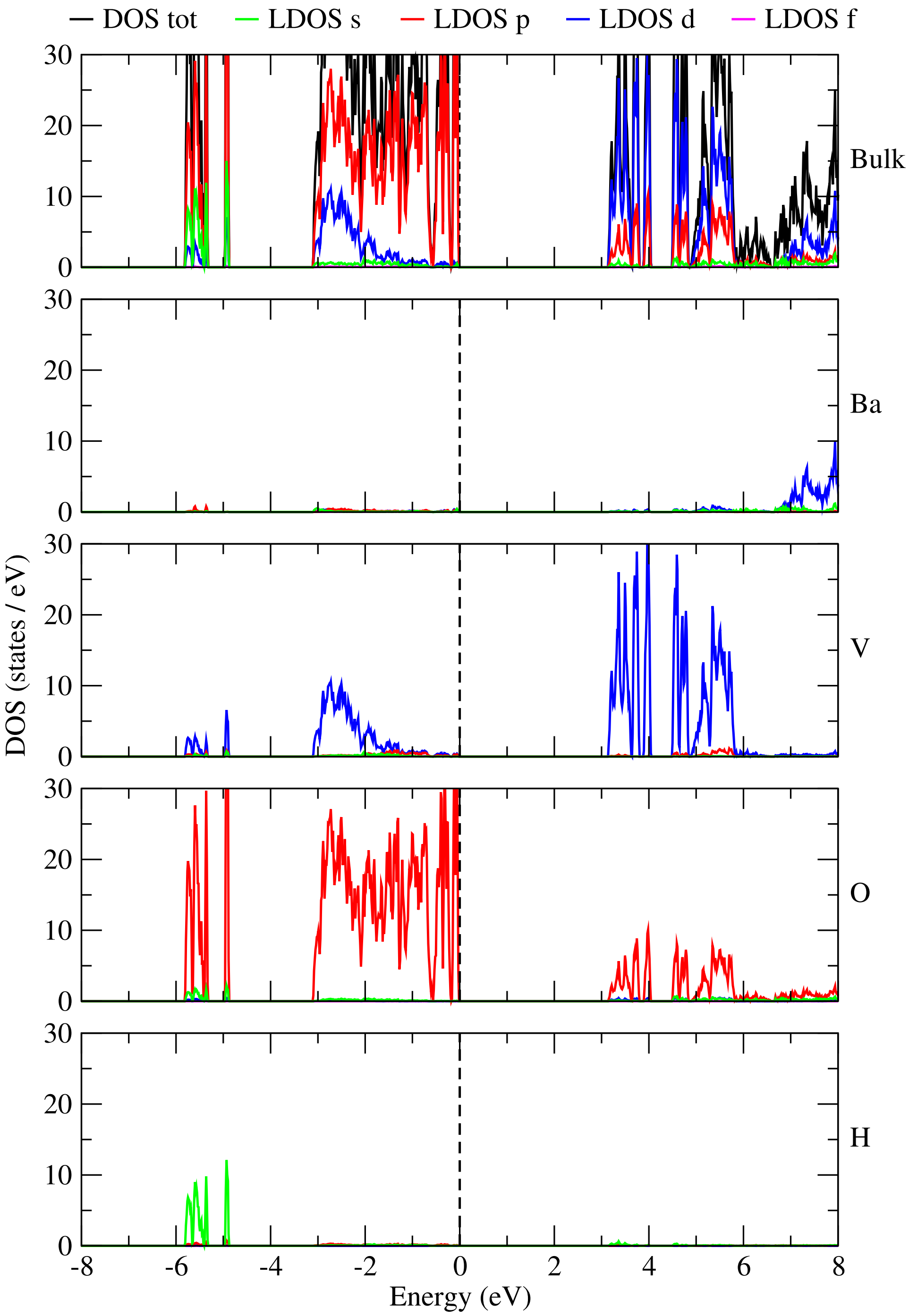

Figure S-16: Local density of states for the $Ba(H_2VO_4)_2$ bulk phase.

Table S-19: Calculated properties for the $Ba(H_2VO_4)_2$ bulk phase. Number of non-equivalent species, N; average distance for nearest neighbors, d_{NN}; effective coordination number, ECN; and net atomic charge, Q.

Non-equivalent species	N	d_{NN} (Å)	ECN (NNN)	Q (e^-)
Ba(I)	2	2.7108	7.7566	1.592 350
V(I)	2	1.6594	3.8031	1.987 599
V(II)	2	1.6249	3.6435	1.982 248
O(I)	2	1.6594	1.0000	−0.918 582
O(II)	2	1.6701	1.9900	−0.913 319
O(III)	2	1.0081	1.0000	−0.863 084
O(IV)	2	1.0042	1.0000	−0.910 286
O(V)	2	1.6809	1.0000	−0.989 367
O(VI)	2	0.9987	1.0000	−0.914 751
O(VII)	2	0.9767	1.0000	−0.934 937
O(VIII)	2	1.6249	1.9973	−0.753 078
H(I)	2	1.0081	1.0000	0.413 169
H(II)	2	1.0042	1.0000	0.413 155
H(III)	2	0.9987	1.0000	0.411 470
H(IV)	2	0.9767	1.0000	0.397 415

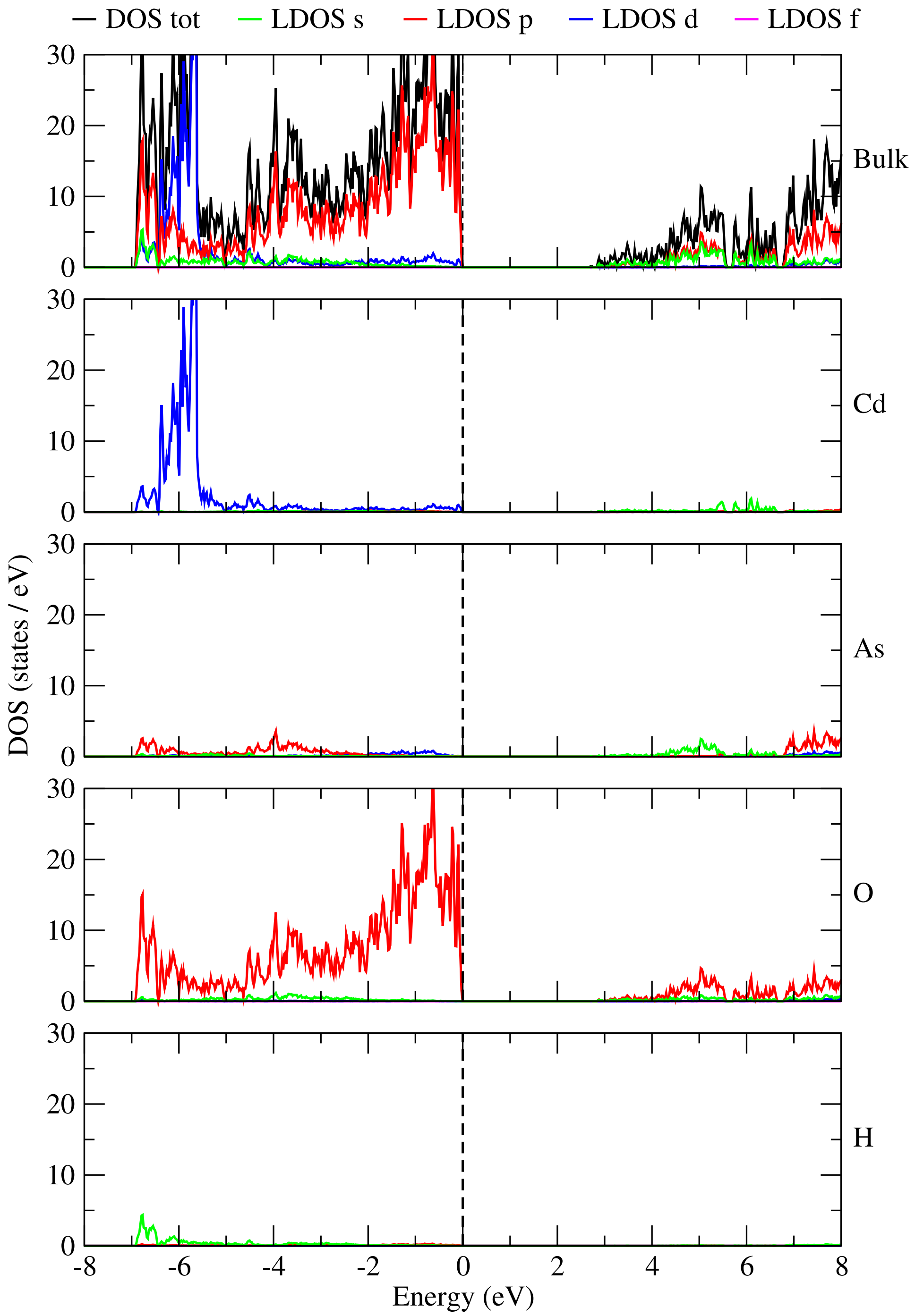

Figure S-17: Local density of states for the $Cd(H_2AsO_4)_2$ bulk phase.

Table S-20: Calculated properties for the $Cd(H_2AsO_4)_2$ bulk phase. Number of non-equivalent species, N; average distance for nearest neighbors, d_{NN}; effective coordination number, ECN; and net atomic charge, Q.

Non-equivalent species	N	d_{NN} (Å)	ECN (NNN)	Q (e^-)
Cd(I)	2	2.2100	5.9728	1.161 580
As(I)	2	1.6959	3.9695	1.682 425
As(II)	2	1.6901	4.0191	1.728 216
O(I)	2	1.7143	1.0613	−0.865 294
O(II)	2	1.6299	1.9931	−0.835 511
O(III)	2	1.4997	2.7340	−0.766 518
O(IV)	2	1.0199	1.0000	−0.722 313
O(V)	2	1.6901	1.0070	−0.859 234
O(VI)	2	1.0355	1.0000	−0.684 513
O(VII)	2	0.9995	1.0000	−0.716 007
O(VIII)	2	1.0467	1.0000	−0.689 150
H(I)	2	1.0467	1.0017	0.380 372
H(II)	2	1.0199	1.0000	0.392 610
H(III)	2	1.0355	1.0002	0.399 627
H(IV)	2	0.9995	1.0000	0.393 711

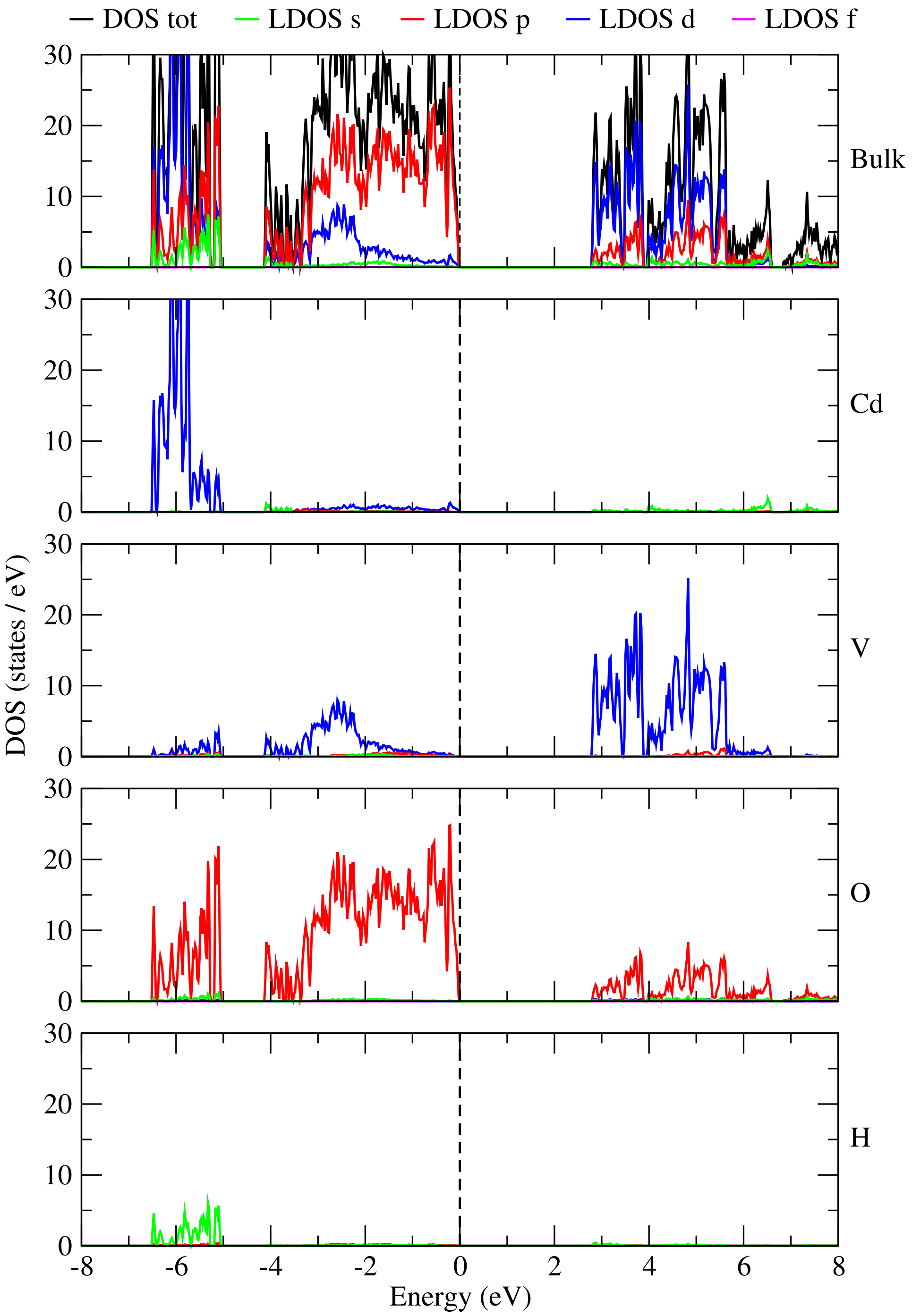

Figure S-18: Local density of states for the $Cd(H_2VO_4)_2$ bulk phase.

Table S-21: Calculated properties for the $Cd(H_2VO_4)_2$ bulk phase. Number of non-equivalent species, N; average distance for nearest neighbors, d_{NN}; effective coordination number, ECN; and net atomic charge, Q.

Non-equivalent species	N	d_{NN} (Å)	ECN (NNN)	Q (e^-)
Cd(I)	2	2.2761	6.0649	1.252 646
V(I)	2	1.6801	3.9156	2.011 312
V(II)	2	1.6174	3.6678	1.992 704
O(I)	2	1.6912	1.0182	−0.925 717
O(II)	2	1.6801	2.0180	−0.868 322
O(III)	2	1.0071	1.0000	−0.854 121
O(IV)	2	1.0037	1.0000	−0.820 704
O(V)	2	1.7043	1.0360	−0.959 671
O(VI)	2	1.0043	1.0000	−0.852 513
O(VII)	2	1.0031	1.0000	−0.928 032
O(VIII)	2	1.6174	1.9889	−0.709 043
H(I)	2	1.0071	1.0000	0.405 296
H(II)	2	1.0037	1.0000	0.405 620
H(III)	2	1.0043	1.0000	0.425 275
H(IV)	2	1.0031	1.0000	0.425 270

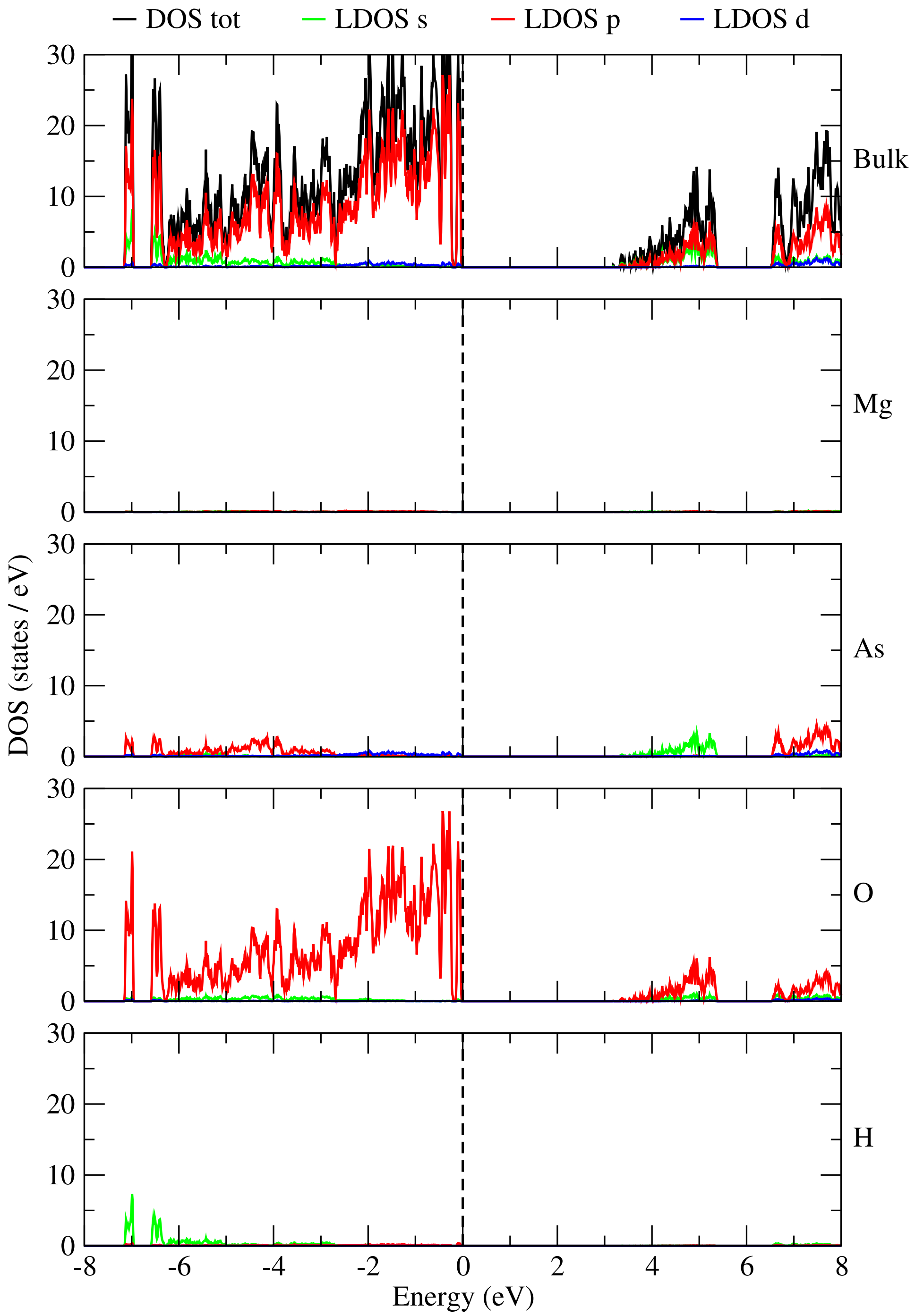

Figure S-19: Local density of states for the $Mg(H_2AsO_4)_2$ bulk phase.

Table S-22: Calculated properties for the $Mg(H_2AsO_4)_2$ bulk phase. Number of non-equivalent species, N; average distance for nearest neighbors, d_{NN}; effective coordination number, ECN; and net atomic charge, Q.

Non-equivalent species	N	d_{NN} (Å)	ECN (NNN)	Q (e^-)
Mg(I)	2	2.0522	5.9246	1.474 584
As(I)	2	1.6981	4.0170	1.768 690
As(II)	2	1.6562	3.8916	1.753 605
O(I)	2	1.7074	1.6558	−0.987 645
O(II)	2	1.6981	2.3354	−0.907 736
O(III)	2	1.0788	1.0000	−0.739 750
O(IV)	2	1.0102	1.0000	−0.720 384
O(V)	2	1.6969	1.4050	−0.977 592
O(VI)	2	1.0063	1.0000	−0.712 595
O(VII)	2	1.0020	1.0000	−0.800 128
O(VIII)	2	1.3877	1.4547	−0.739 220
H(I)	2	1.0788	1.0973	0.387 801
H(II)	2	1.0102	1.0000	0.383 095
H(III)	2	1.0063	1.0000	0.404 102
H(IV)	2	1.0020	1.0000	0.413 177

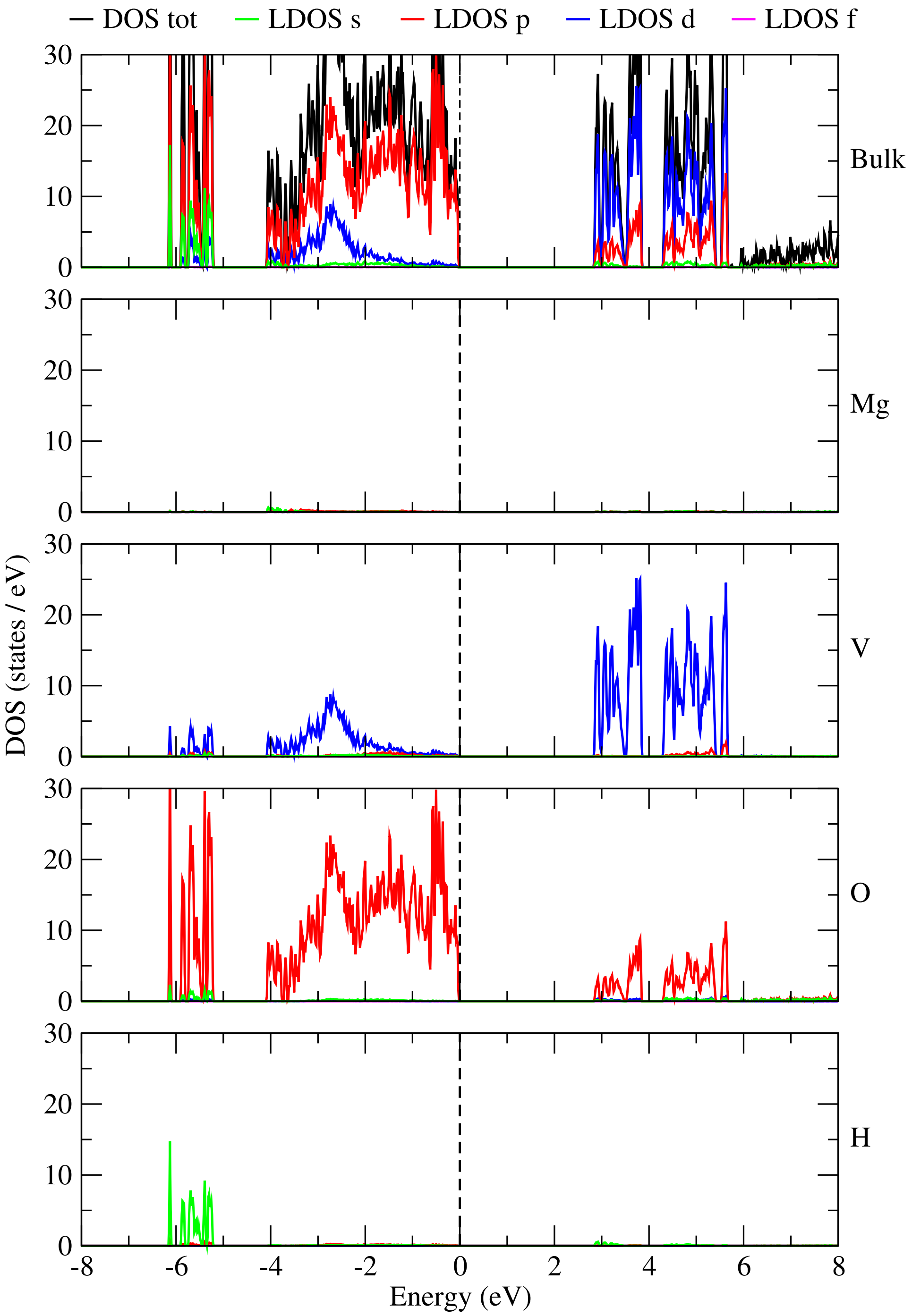

Figure S-20: Local density of states for the $Mg(H_2VO_4)_2$ bulk phase.

Table S-23: Calculated properties for the $Mg(H_2VO_4)_2$ bulk phase. Number of non-equivalent species, N; average distance for nearest neighbors, d_{NN}; effective coordination number, ECN; and net atomic charge, Q.

Non-equivalent species	N	d_{NN} (Å)	ECN (NNN)	Q (e^-)
Mg(I)	2	2.0894	6.0152	1.516 705
V(I)	2	1.6730	3.9245	2.052 744
V(II)	2	1.6140	3.6606	2.013 435
O(I)	2	1.7013	1.5361	−1.047 636
O(II)	2	1.6730	2.2205	−0.915 135
O(III)	2	1.0081	1.0000	−0.851 935
O(IV)	2	0.9969	1.0000	−0.835 254
O(V)	2	1.7071	1.6539	−1.077 068
O(VI)	2	0.9975	1.0000	−0.838 747
O(VII)	2	1.0003	1.0000	−0.978 629
O(VIII)	2	1.6140	1.9938	−0.699 469
H(I)	2	1.0081	1.0000	0.412 357
H(II)	2	0.9969	1.0000	0.402 415
H(III)	2	0.9975	1.0000	0.419 172
H(IV)	2	1.0003	1.0000	0.427 046

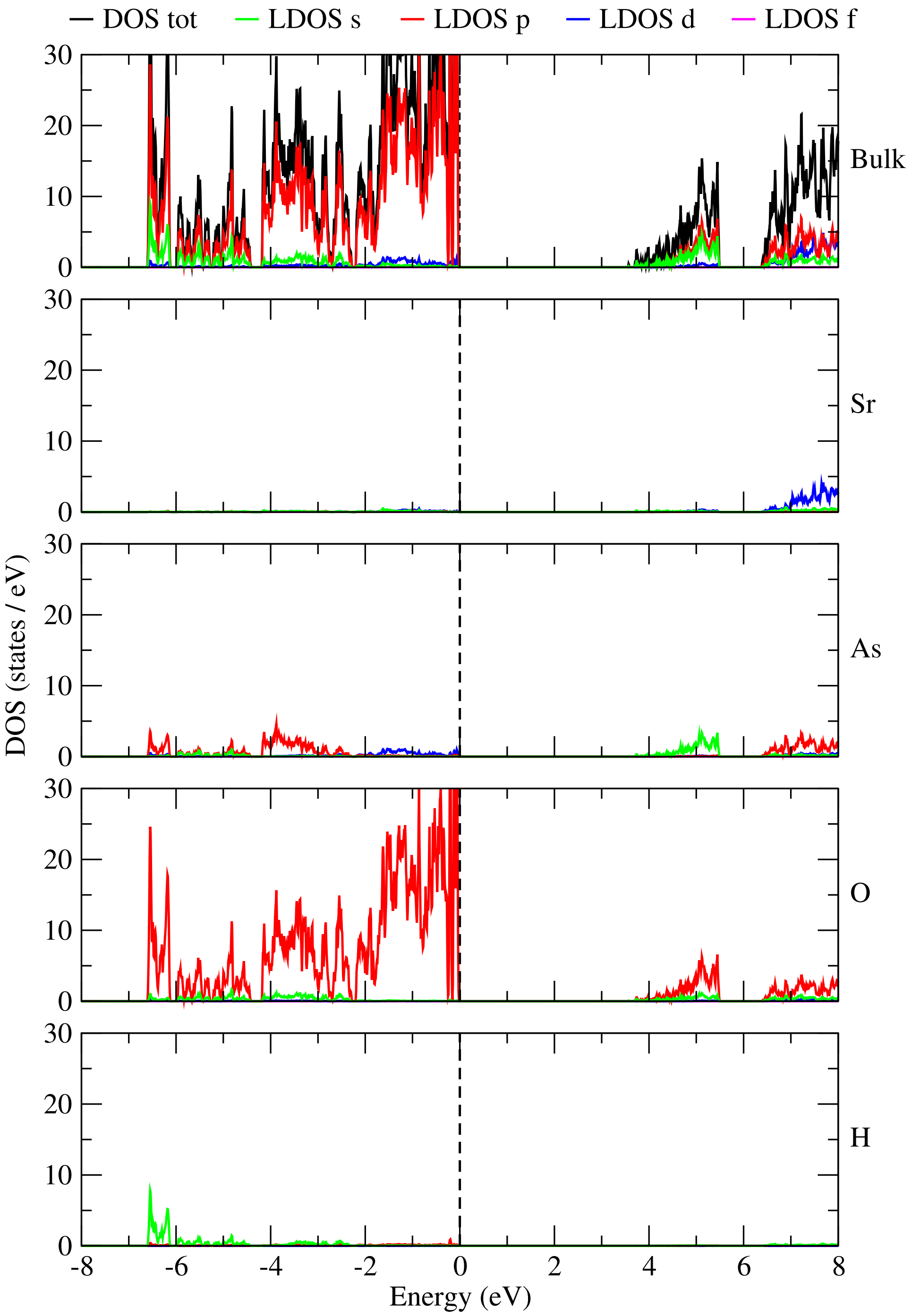

Figure S-21: Local density of states for the $Sr(H_2AsO_4)_2$ bulk phase.

Table S-24: Calculated properties for the $Sr(H_2AsO_4)_2$ bulk phase. Number of non-equivalent species, N; average distance for nearest neighbors, d_{NN}; effective coordination number, ECN; and net atomic charge, Q.

Non-equivalent species	N	d_{NN} (Å)	ECN (NNN)	Q (e^-)
Sr(I)	2	2.4996	6.9410	1.539 498
As(I)	2	1.6828	3.9671	1.700 433
As(II)	2	1.6710	3.8953	1.712 718
O(I)	2	1.6828	1.0003	−0.932 878
O(II)	2	1.5773	1.9103	−0.879 895
O(III)	2	1.1032	1.0000	−0.732 849
O(IV)	2	1.0279	1.0000	−0.780 114
O(V)	2	1.6863	1.0001	−0.935 656
O(VI)	2	1.0075	1.0000	−0.731 693
O(VII)	2	0.9782	1.0000	−0.768 660
O(VIII)	2	1.3535	1.2604	−0.766 659
H(I)	2	1.1032	1.2940	0.389 821
H(II)	2	1.0279	1.0000	0.397 469
H(III)	2	1.0075	1.0000	0.390 221
H(IV)	2	0.9782	1.0000	0.398 242

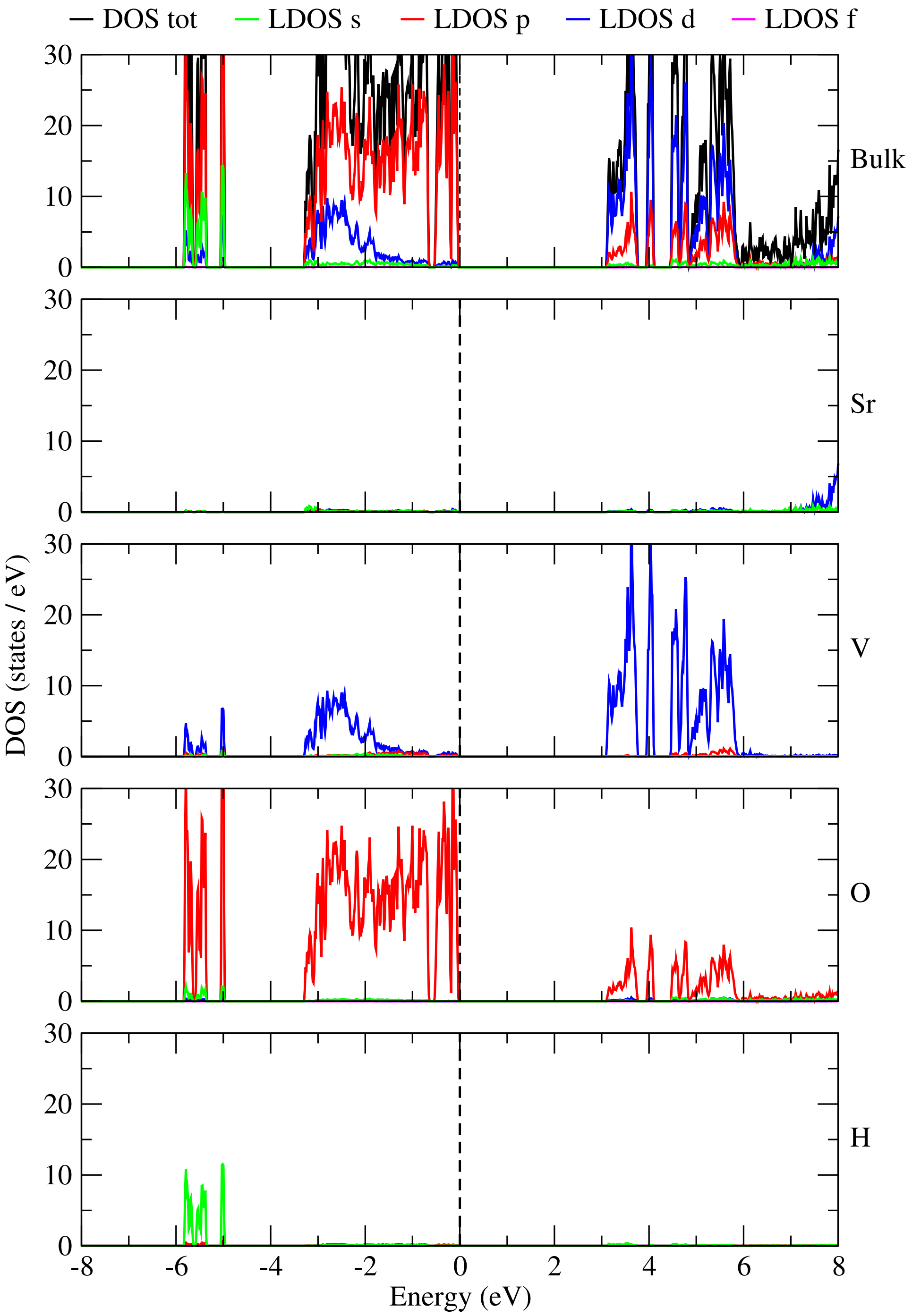

Figure S-22: Local density of states for the $Sr(H_2VO_4)_2$ bulk phase.

Table S-25: Calculated properties for the $Sr(H_2VO_4)_2$ bulk phase. Number of non-equivalent species, N; average distance for nearest neighbors, d_{NN}; effective coordination number, ECN; and net atomic charge, Q.

Non-equivalent species	N	d_{NN} (Å)	ECN (NNN)	Q (e^-)
Sr(I)	2	2.5462	7.2757	1.592 138
V(I)	2	1.6598	3.8176	1.996 965
V(II)	2	1.6260	3.6654	1.991 179
O(I)	2	1.6598	1.0000	−0.936 246
O(II)	2	1.6721	1.9920	−0.921 371
O(III)	2	1.0144	1.0000	−0.862 692
O(IV)	2	1.0049	1.0000	−0.908 963
O(V)	2	1.6903	1.0002	−1.023 423
O(VI)	2	1.0016	1.0000	−0.879 832
O(VII)	2	0.9755	1.0000	−0.944 056
O(VIII)	2	1.5977	1.9949	−0.754 643
H(I)	2	1.0144	1.0000	0.419 749
H(II)	2	1.0049	1.0000	0.415 809
H(III)	2	1.0016	1.0000	0.414 802
H(IV)	2	0.9755	1.0000	0.400 584

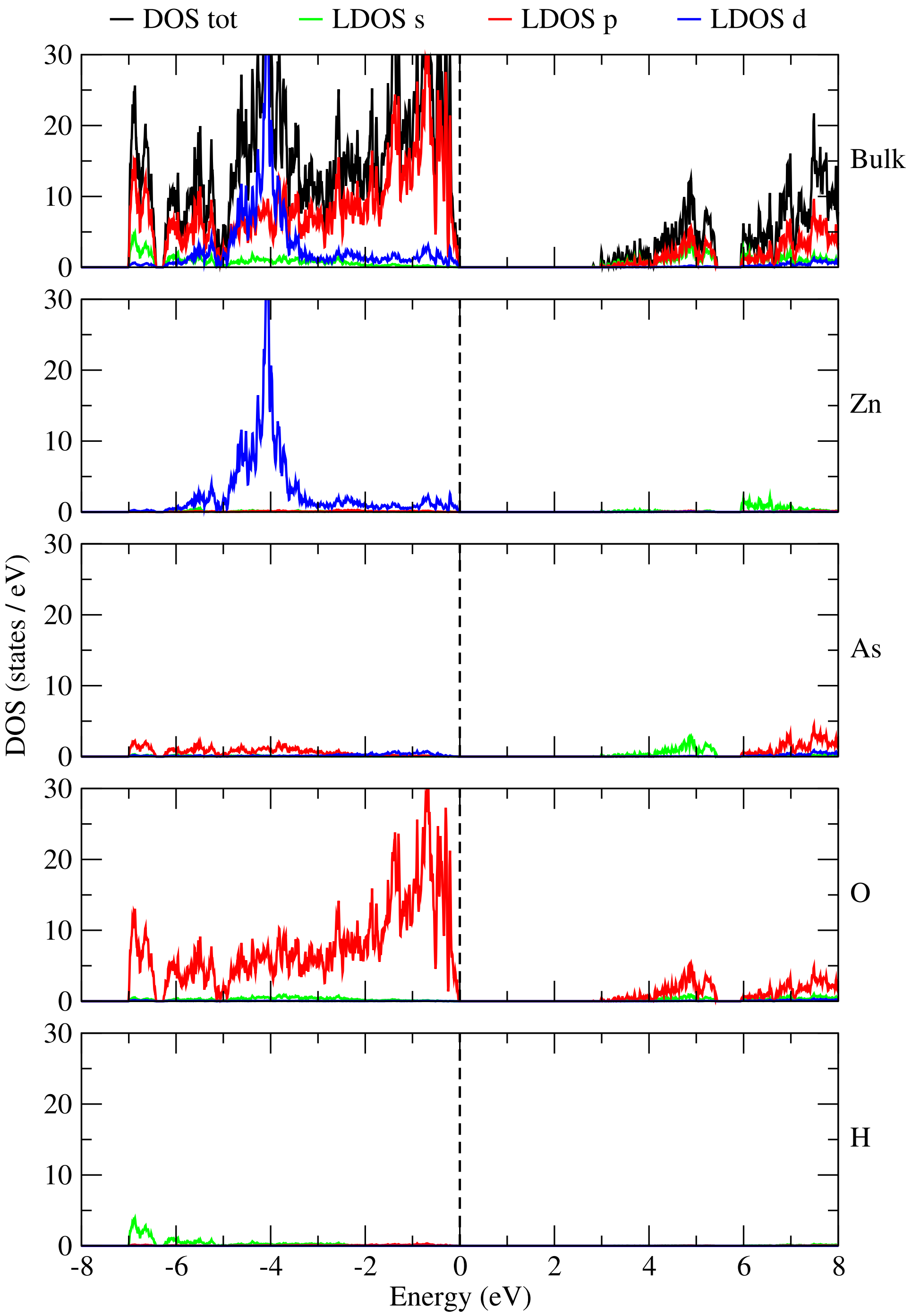

Figure S-23: Local density of states for the $Zn(H_2AsO_4)_2$ bulk phase.

Table S-26: Calculated properties for the $Zn(H_2AsO_4)_2$ bulk phase. Number of non-equivalent species, N; average distance for nearest neighbors, d_{NN}; effective coordination number, ECN; and net atomic charge, Q.

Non-equivalent species	N	d_{NN} (Å)	ECN (NNN)	Q (e^-)
Zn(I)	2	1.9945	5.5795	1.079 289
As(I)	2	1.6908	3.9719	1.710 352
As(II)	2	1.6949	4.0438	1.742 001
O(I)	2	1.7218	1.9825	−0.853 282
O(II)	2	1.6984	2.5022	−0.825 337
O(III)	2	1.4793	2.6978	−0.767 925
O(IV)	2	1.0115	1.0000	−0.727 242
O(V)	2	1.6949	1.1244	−0.851 289
O(VI)	2	1.0271	1.0000	−0.683 786
O(VII)	2	1.0006	1.0000	−0.704 260
O(VIII)	2	1.0513	1.0000	−0.694 747
H(I)	2	1.0513	1.0041	0.377 552
H(II)	2	1.0115	1.0000	0.391 391
H(III)	2	1.0271	1.0000	0.402 979
H(IV)	2	1.0006	1.0000	0.404 303

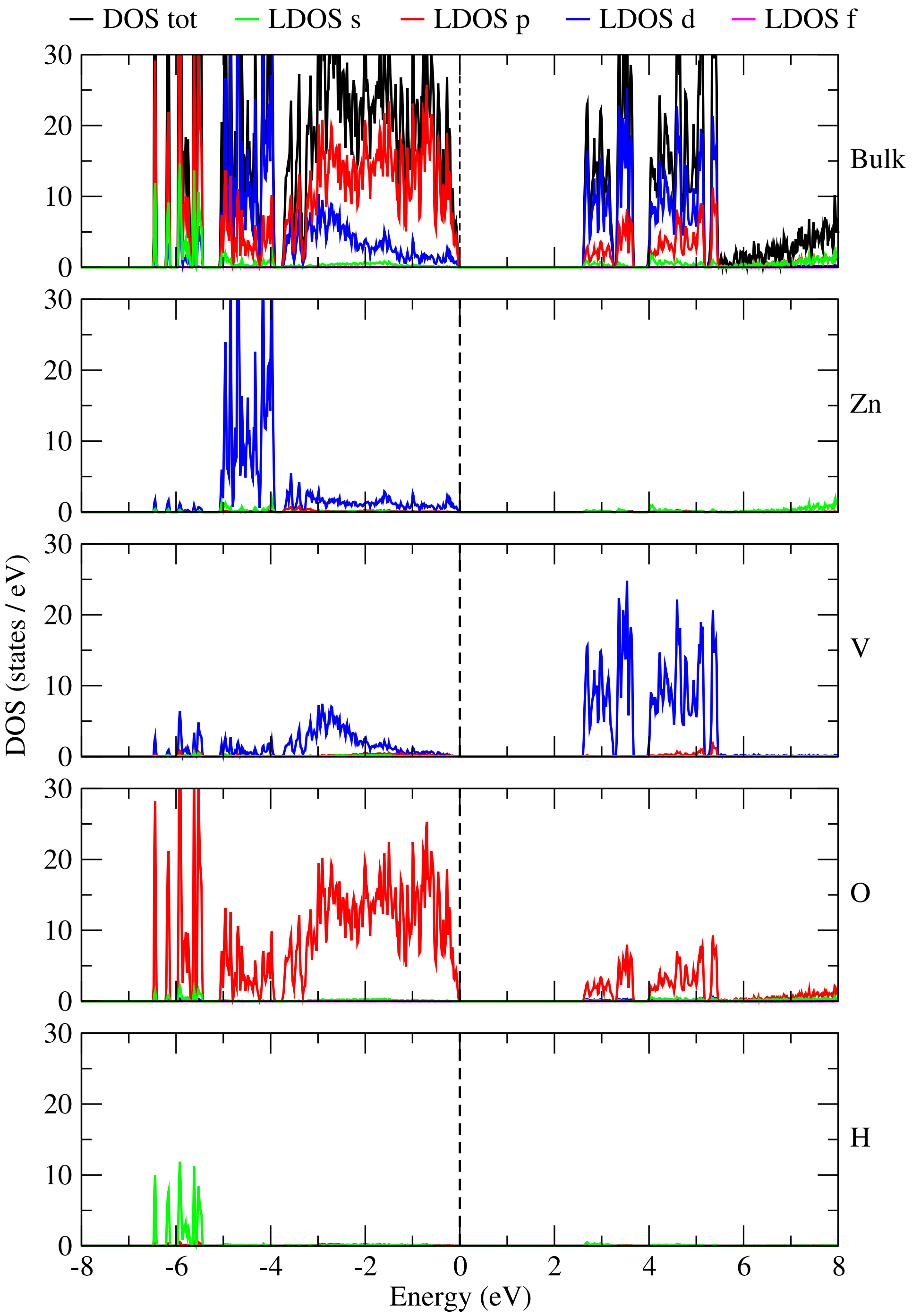

Figure S-24: Local density of states for the $Zn(H_2VO_4)_2$ bulk phase.

Table S-27: Calculated properties for the $Zn(H_2VO_4)_2$ bulk phase. Number of non-equivalent species, N; average distance for nearest neighbors, d_{NN}; effective coordination number, ECN; and net atomic charge, Q.

Non-equivalent species	N	d_{NN} (Å)	ECN (NNN)	Q (e^-)
Zn(I)	2	2.0683	5.9507	1.179 123
V(I)	2	1.6757	3.9286	2.028 299
V(II)	2	1.6139	3.6626	1.992 529
O(I)	2	1.7038	1.5035	−0.922 748
O(II)	2	1.6757	2.2702	−0.851 288
O(III)	2	1.0086	1.0000	−0.851 718
O(IV)	2	0.9953	1.0000	−0.831 506
O(V)	2	1.7108	1.6241	−0.950 921
O(VI)	2	0.9973	1.0000	−0.834 562
O(VII)	2	1.0007	1.0000	−0.919 067
O(VIII)	2	1.6139	1.9952	−0.698 062
H(I)	2	1.0086	1.0000	0.414 917
H(II)	2	0.9953	1.0000	0.400 584
H(III)	2	0.9973	1.0000	0.418 298
H(IV)	2	1.0007	1.0000	0.426 123

S-6.2 $CaHPO_4$-based Materials

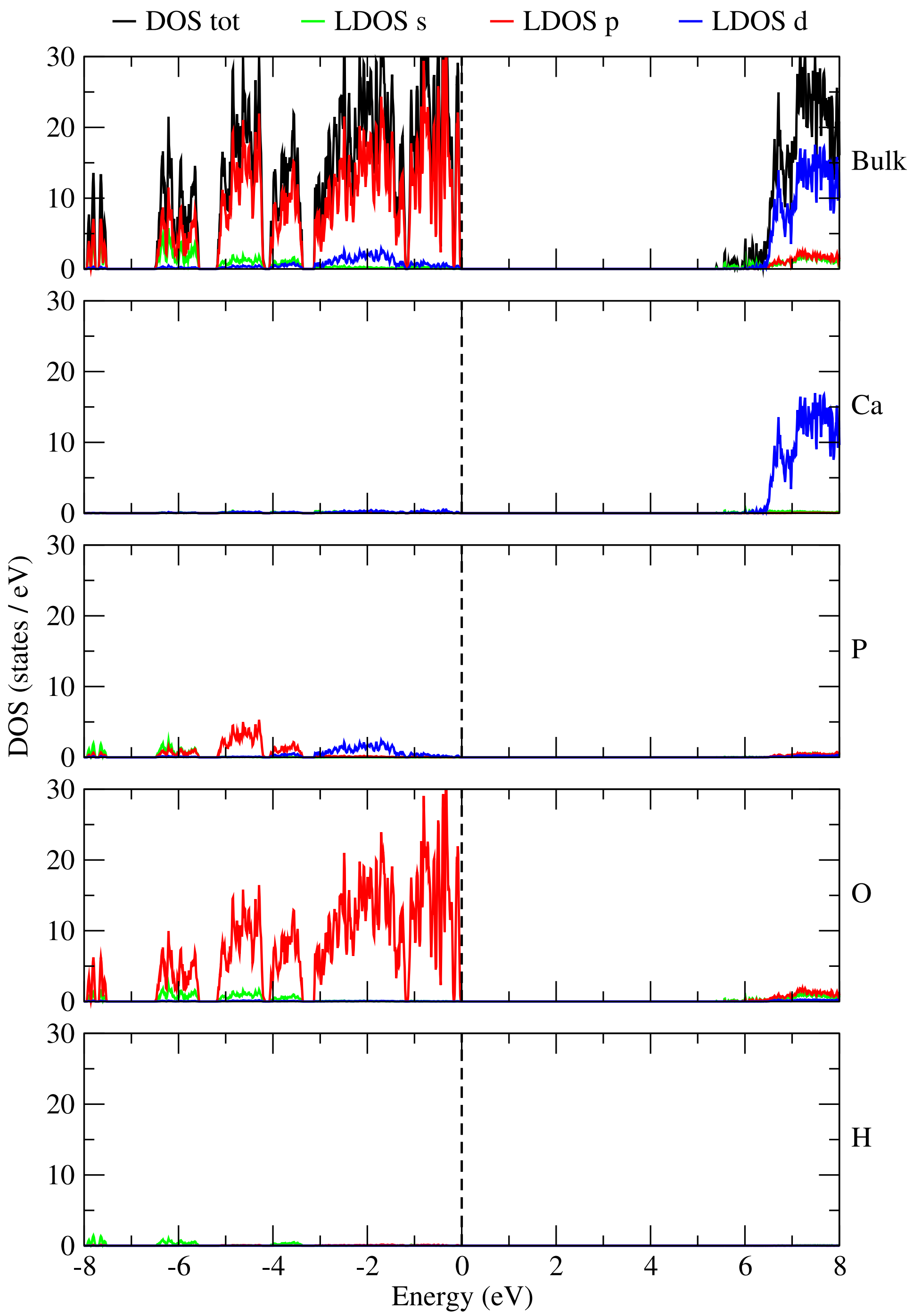

Figure S-25: Local density of states for the $CaHPO_4$ bulk phase.

Table S-28: Calculated properties for the $CaHPO_4$ bulk phase. Number of non-equivalent species, N; average distance for nearest neighbors, d_{NN}; effective coordination number, ECN; and net atomic charge, Q.

Non-equivalent species	N	d_{NN} (Å)	ECN (NNN)	Q (e^-)
Ca(I)	1	2.3227	6.4635	1.497 443
Ca(II)	1	2.2875	7.0426	1.489 763
Ca(III)	1	2.3912	8.9585	1.466 972
Ca(IV)	1	2.3826	8.3186	1.480 044
P(I)	1	1.5354	3.9359	1.565 416
P(II)	1	1.5351	3.9344	1.560 036
P(III)	1	1.5304	3.9596	1.546 308
P(IV)	1	1.5234	3.9097	1.562 454
O(I)	1	1.5234	1.0000	−0.894 207
O(II)	1	1.4997	1.9748	−0.880 829
O(III)	1	1.4409	1.8994	−0.862 906
O(IV)	1	1.0509	1.0000	−0.761 452
O(V)	1	1.0363	1.0000	−0.755 460
O(VI)	1	1.0434	1.0000	−0.753 919
O(VII)	1	1.5618	1.0000	−0.935 859
O(VIII)	1	1.5609	1.0000	−0.938 256
O(IX)	1	1.5354	1.0000	−0.888 829
O(X)	1	1.5370	1.0000	−0.898 798
O(XI)	1	1.5375	1.0002	−0.918 938
O(XII)	1	1.5351	1.0001	−0.911 087
O(XIII)	1	1.5300	1.9982	−0.836 630
O(XIV)	1	1.5369	1.9968	−0.831 514
O(XV)	1	1.5304	1.0000	−0.889 296
O(XVI)	1	1.0393	1.0000	−0.784 775
H(I)	1	1.0393	1.0012	0.394 346
H(II)	1	1.0434	1.0005	0.395 568
H(III)	1	1.0363	1.0001	0.395 280
H(IV)	1	1.0509	1.0119	0.389 125

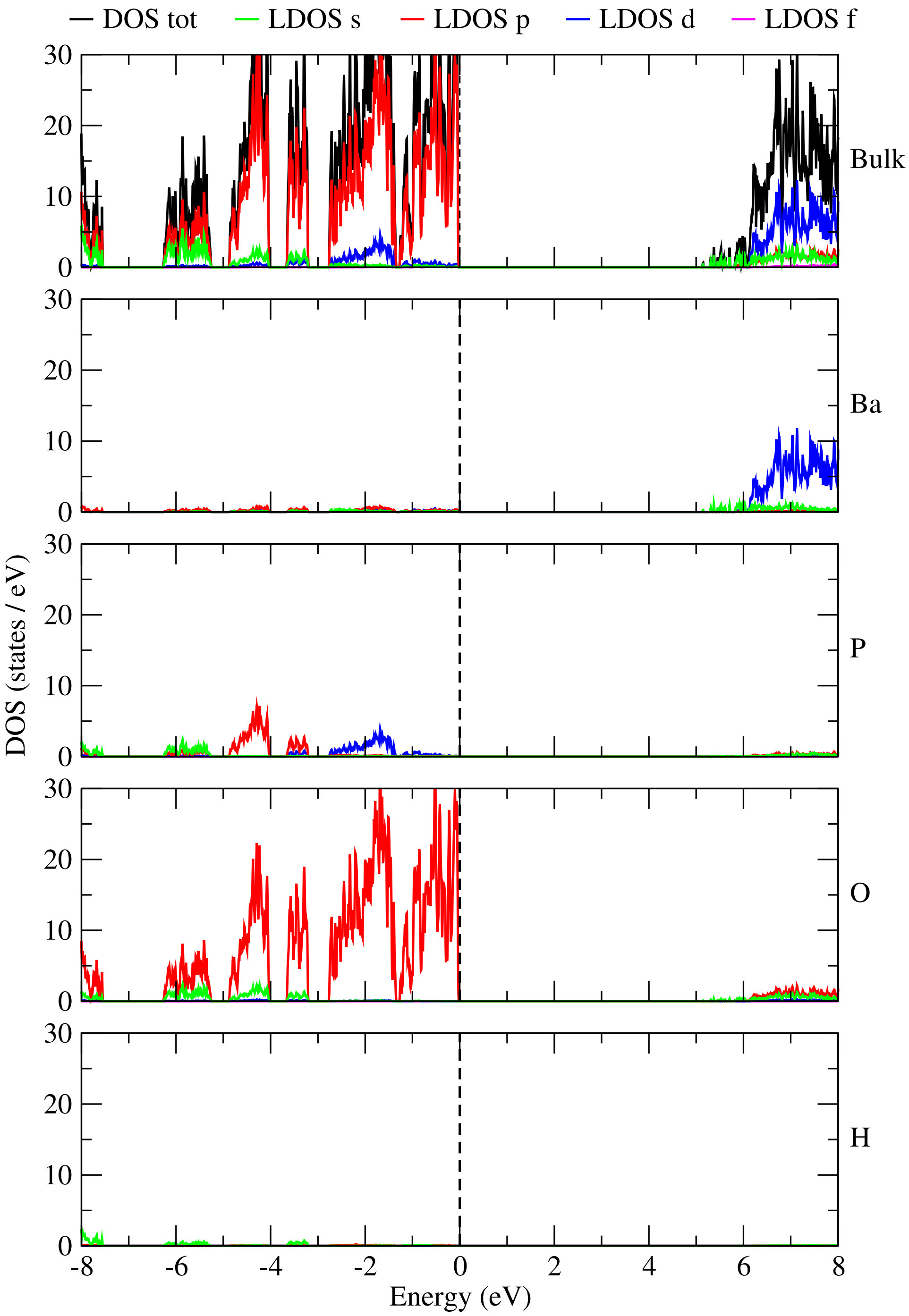

Figure S-26: Local density of states for the $BaHPO_4$ bulk phase.

Table S-29: Calculated properties for the $BaHPO_4$ bulk phase. Number of non-equivalent species, N; average distance for nearest neighbors, d_{NN}; effective coordination number, ECN; and net atomic charge, Q.

Non-equivalent species	N	d_{NN} (Å)	ECN (NNN)	Q (e^-)
Ba(I)	1	2.6963	8.9864	1.542 250
Ba(II)	1	2.6415	9.7362	1.532 051
Ba(III)	1	2.6853	10.2880	1.522 034
Ba(IV)	1	2.6497	9.1115	1.529 314
P(I)	1	1.5349	3.9168	1.520 600
P(II)	1	1.5360	3.9191	1.527 691
P(III)	1	1.5262	3.9314	1.532 370
P(IV)	1	1.5233	3.9216	1.538 500
O(I)	1	1.5233	1.0000	−0.892 329
O(II)	1	1.5400	1.9990	−0.874 771
O(III)	1	1.4966	1.9730	−0.863 832
O(IV)	1	1.0417	1.0000	−0.771 996
O(V)	1	1.0216	1.0000	−0.763 799
O(VI)	1	1.0239	1.0000	−0.762 867
O(VII)	1	1.5573	1.0000	−0.928 780
O(VIII)	1	1.5537	1.0000	−0.921 236
O(IX)	1	1.5349	1.0000	−0.900 994
O(X)	1	1.5360	1.0000	−0.902 841
O(XI)	1	1.5442	1.0000	−0.914 453
O(XII)	1	1.5468	1.0000	−0.928 276
O(XIII)	1	1.5565	1.9988	−0.876 715
O(XIV)	1	1.5488	1.9899	−0.873 966
O(XV)	1	1.5262	1.0000	−0.879 290
O(XVI)	1	1.0328	1.0000	−0.773 105
H(I)	1	1.0328	1.0002	0.403 423
H(II)	1	1.0239	1.0000	0.392 818
H(III)	1	1.0216	1.0000	0.395 573
H(IV)	1	1.0417	1.0015	0.392 627

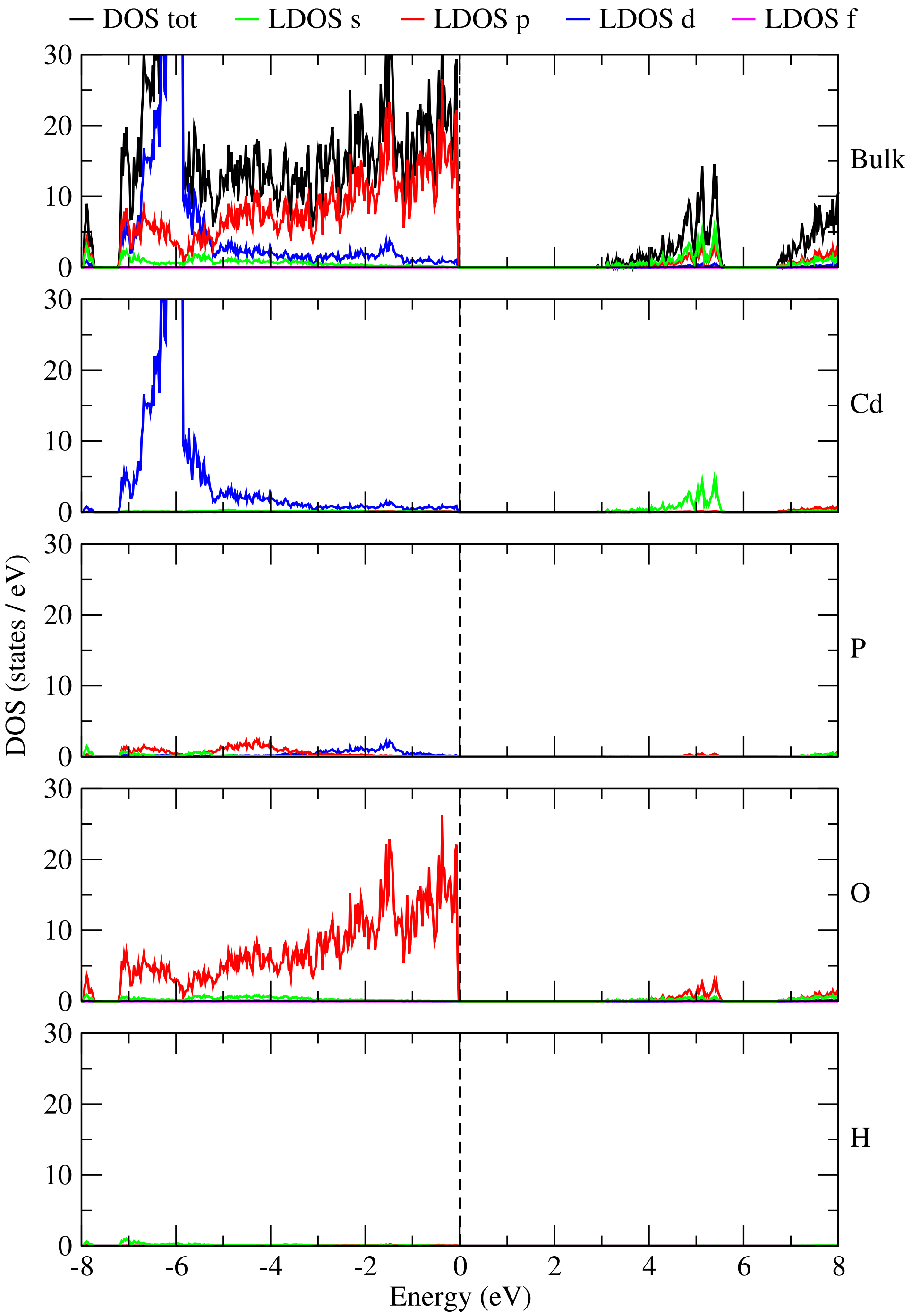

Figure S-27: Local density of states for the $CdHPO_4$ bulk phase.

Table S-30: Calculated properties for the $CdHPO_4$ bulk phase. Number of non-equivalent species, N; average distance for nearest neighbors, d_{NN}; effective coordination number, ECN; and net atomic charge, Q.

Non-equivalent species	N	d_{NN} (Å)	ECN (NNN)	Q (e^-)
Cd(I)	1	2.2439	6.0691	1.235 418
Cd(II)	1	2.2584	6.6672	1.241 012
Cd(III)	1	2.2995	7.9808	1.219 738
Cd(IV)	1	2.3178	8.0649	1.246 013
P(I)	1	1.5415	3.9551	1.544 978
P(II)	1	1.5298	3.9294	1.538 086
P(III)	1	1.5381	3.9854	1.540 190
P(IV)	1	1.5318	3.9318	1.550 061
O(I)	1	1.5318	1.0003	−0.821 195
O(II)	1	1.4078	1.8000	−0.811 435
O(III)	1	1.3560	1.6890	−0.796 335
O(IV)	1	1.0680	1.0002	−0.728 766
O(V)	1	1.0430	1.0000	−0.715 763
O(VI)	1	1.0431	1.0000	−0.708 883
O(VII)	1	1.5668	1.0001	−0.849 443
O(VIII)	1	1.5704	1.0000	−0.847 645
O(IX)	1	1.5415	1.0001	−0.816 497
O(X)	1	1.5446	1.0001	−0.826 433
O(XI)	1	1.5432	1.0006	−0.823 663
O(XII)	1	1.5298	1.0005	−0.821 215
O(XIII)	1	1.5306	1.9982	−0.795 192
O(XIV)	1	1.5152	1.9966	−0.789 375
O(XV)	1	1.5381	1.0004	−0.805 567
O(XVI)	1	1.0639	1.0000	−0.738 979
H(I)	1	1.0639	1.0417	0.396 090
H(II)	1	1.0431	1.0005	0.397 280
H(III)	1	1.0430	1.0008	0.395 788
H(IV)	1	1.0680	1.1372	0.391 731

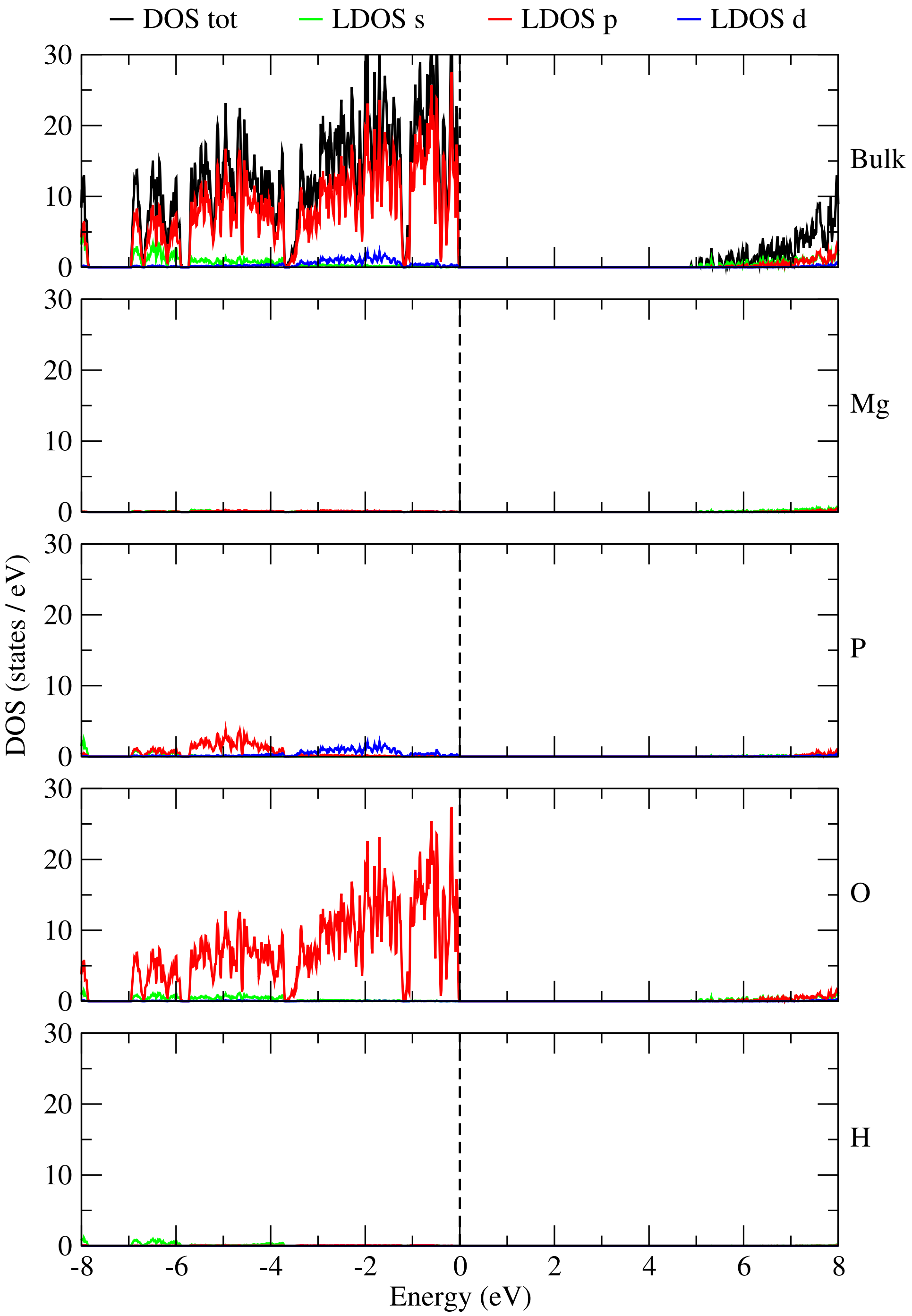

Figure S-28: Local density of states for the $MgHPO_4$ bulk phase.

Table S-31: Calculated properties for the $MgHPO_4$ bulk phase. Number of non-equivalent species, N; average distance for nearest neighbors, d_{NN}; effective coordination number, ECN; and net atomic charge, Q.

Non-equivalent species	N	d_{NN} (Å)	ECN (NNN)	Q (e^-)
Mg(I)	1	1.9534	4.8769	1.487 850
Mg(II)	1	1.9278	4.8969	1.484 108
Mg(III)	1	1.9726	4.4044	1.510 360
Mg(IV)	1	1.9584	4.8929	1.510 635
P(I)	1	1.5082	3.8412	1.636 092
P(II)	1	1.5049	3.8345	1.647 399
P(III)	1	1.5445	3.9964	1.614 982
P(IV)	1	1.5291	3.8611	1.652 742
O(I)	1	1.5399	1.0586	−0.949 381
O(II)	1	1.5640	1.8663	−0.921 115
O(III)	1	1.5318	2.0206	−0.881 011
O(IV)	1	1.0186	1.0000	−0.713 163
O(V)	1	1.0078	1.0000	−0.760 214
O(VI)	1	1.0204	1.0000	−0.763 055
O(VII)	1	1.5807	1.0608	−0.981 115
O(VIII)	1	1.5824	1.0761	−0.989 278
O(IX)	1	1.5146	1.0683	−0.904 963
O(X)	1	1.5185	1.0905	−0.940 801
O(XI)	1	1.5082	1.1152	−0.911 992
O(XII)	1	1.5049	1.0752	−0.892 804
O(XIII)	1	1.5445	2.0099	−0.902 211
O(XIV)	1	1.5291	1.8419	−0.882 851
O(XV)	1	1.5511	1.0536	−0.949 027
O(XVI)	1	1.0070	1.0000	−0.781 754
H(I)	1	1.0070	1.0000	0.406 796
H(II)	1	1.0204	1.0000	0.398 596
H(III)	1	1.0078	1.0000	0.390 989
H(IV)	1	1.0186	1.0000	0.384 185

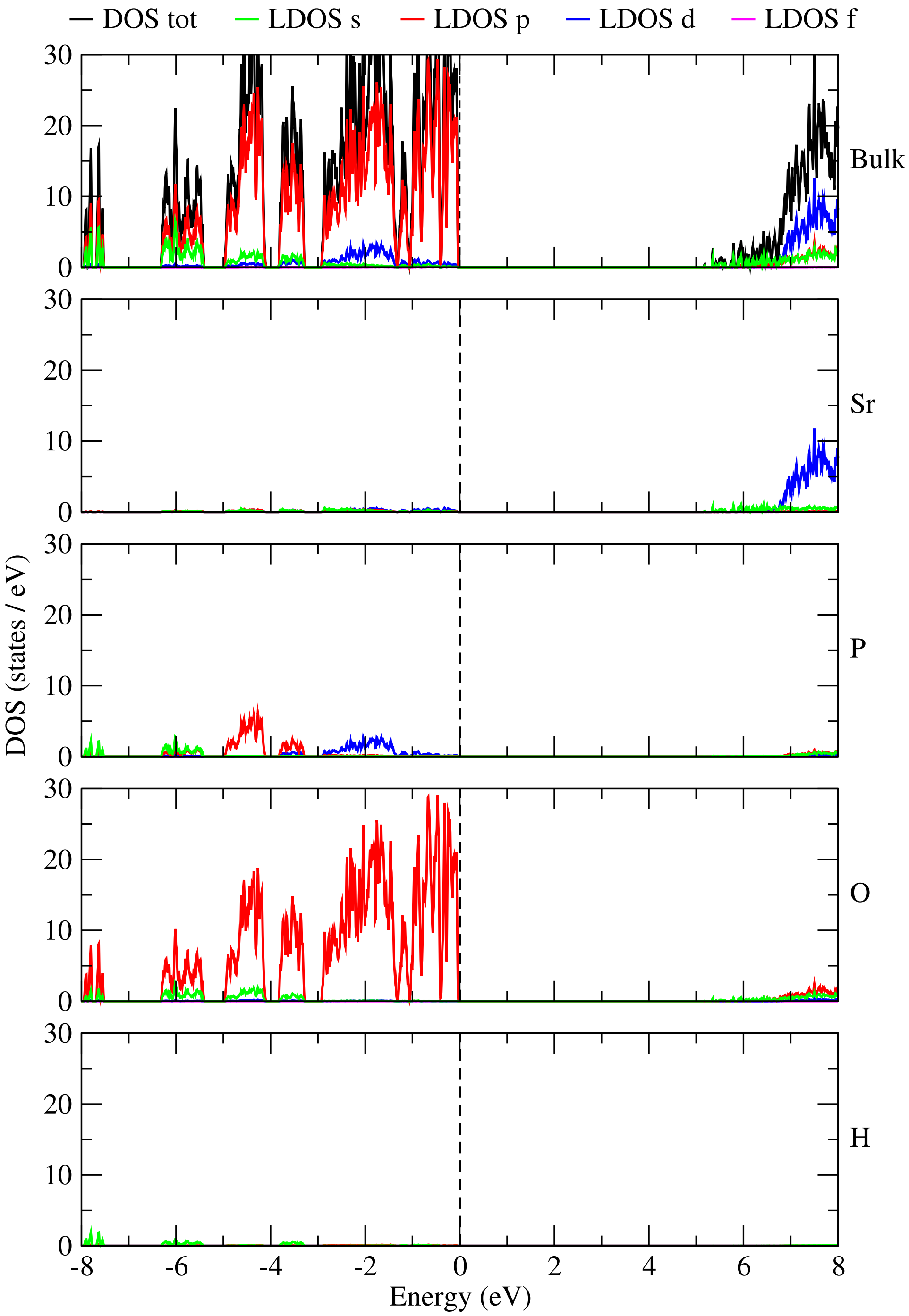

Figure S-29: Local density of states for the $SrHPO_4$ bulk phase.

Table S-32: Calculated properties for the $SrHPO_4$ bulk phase. Number of non-equivalent species, N; average distance for nearest neighbors, d_{NN}; effective coordination number, ECN; and net atomic charge, Q.

Non-equivalent species	N	d_{NN} (Å)	ECN (NNN)	Q (e^-)
Sr(I)	1	2.4927	7.0909	1.548 234
Sr(II)	1	2.4544	7.4903	1.542 436
Sr(III)	1	2.5468	9.4594	1.518 431
Sr(IV)	1	2.5310	8.5335	1.528 961
P(I)	1	1.5339	3.9279	1.548 049
P(II)	1	1.5348	3.9285	1.545 849
P(III)	1	1.5268	3.9419	1.539 006
P(IV)	1	1.5225	3.9077	1.549 709
O(I)	1	1.5225	1.0000	−0.904 308
O(II)	1	1.5263	1.9940	−0.890 453
O(III)	1	1.4815	1.9580	−0.870 271
O(IV)	1	1.0436	1.0000	−0.767 377
O(V)	1	1.0286	1.0000	−0.767 730
O(VI)	1	1.0333	1.0000	−0.766 745
O(VII)	1	1.5593	1.0000	−0.944 610
O(VIII)	1	1.5576	1.0000	−0.942 627
O(IX)	1	1.5339	1.0000	−0.899 463
O(X)	1	1.5348	1.0000	−0.907 530
O(XI)	1	1.5397	1.0000	−0.930 079
O(XII)	1	1.5393	1.0000	−0.926 744
O(XIII)	1	1.5459	1.9997	−0.858 948
O(XIV)	1	1.5416	1.9936	−0.850 623
O(XV)	1	1.5268	1.0000	−0.888 962
O(XVI)	1	1.0352	1.0000	−0.785 433
H(I)	1	1.0352	1.0004	0.398 739
H(II)	1	1.0333	1.0001	0.394 994
H(III)	1	1.0286	1.0000	0.397 017
H(IV)	1	1.0436	1.0027	0.390 479

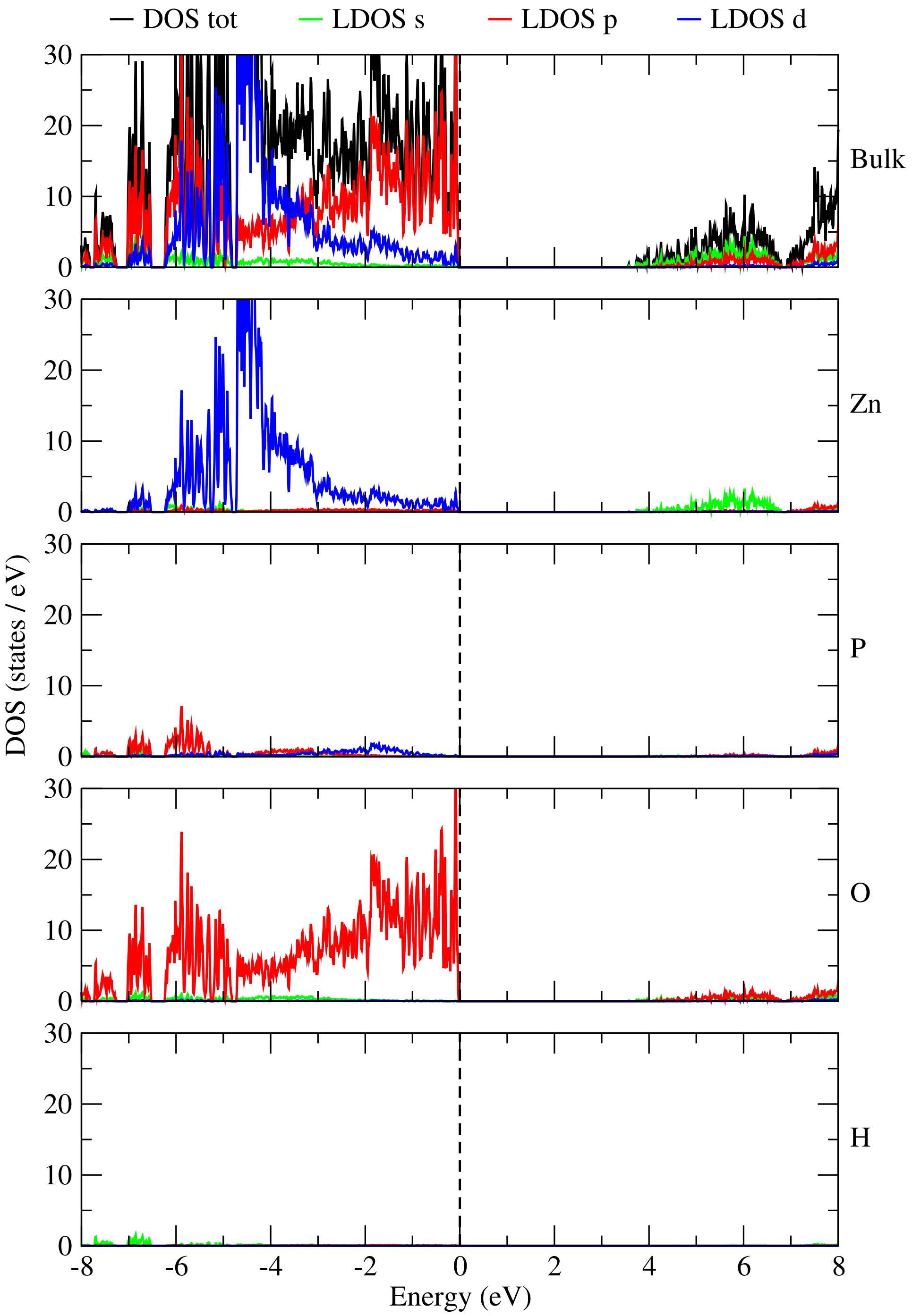

Figure S-30: Local density of states for the $ZnHPO_4$ bulk phase.

Table S-33: Calculated properties for the $ZnHPO_4$ bulk phase. Number of non-equivalent species, N; average distance for nearest neighbors, d_{NN}; effective coordination number, ECN; and net atomic charge, Q.

Non-equivalent species	N	d_{NN} (Å)	ECN (NNN)	Q (e^-)
Zn(I)	1	1.9149	3.6312	1.126 266
Zn(II)	1	1.8993	3.7180	1.129 878
Zn(III)	1	1.9228	3.9897	1.099 580
Zn(IV)	1	1.9213	3.8901	1.116 623
P(I)	1	1.5332	3.9748	1.542 005
P(II)	1	1.5268	3.9423	1.549 735
P(III)	1	1.5212	3.9479	1.522 689
P(IV)	1	1.5324	3.9795	1.593 318
O(I)	1	1.5324	1.2403	−0.801 494
O(II)	1	1.0620	1.0001	−0.694 338
O(III)	1	1.4636	1.9700	−0.828 032
O(IV)	1	1.0444	1.0001	−0.694 201
O(V)	1	1.0260	1.0000	−0.700 933
O(VI)	1	0.9853	1.0000	−0.683 010
O(VII)	1	1.5332	1.2036	−0.792 282
O(VIII)	1	1.5854	1.2960	−0.822 675
O(IX)	1	1.5361	1.1939	−0.774 300
O(X)	1	1.5334	1.1893	−0.794 876
O(XI)	1	1.5834	1.4405	−0.812 960
O(XII)	1	1.5268	1.1693	−0.777 087
O(XIII)	1	1.5212	1.0451	−0.772 583
O(XIV)	1	1.5425	2.0746	−0.813 595
O(XV)	1	1.5582	1.0592	−0.781 822
O(XVI)	1	1.5040	2.0550	−0.783 490
H(I)	1	1.0620	1.0030	0.423 718
H(II)	1	0.9853	1.0000	0.413 952
H(III)	1	1.0260	1.0001	0.396 315
H(IV)	1	1.0444	1.0049	0.413 598

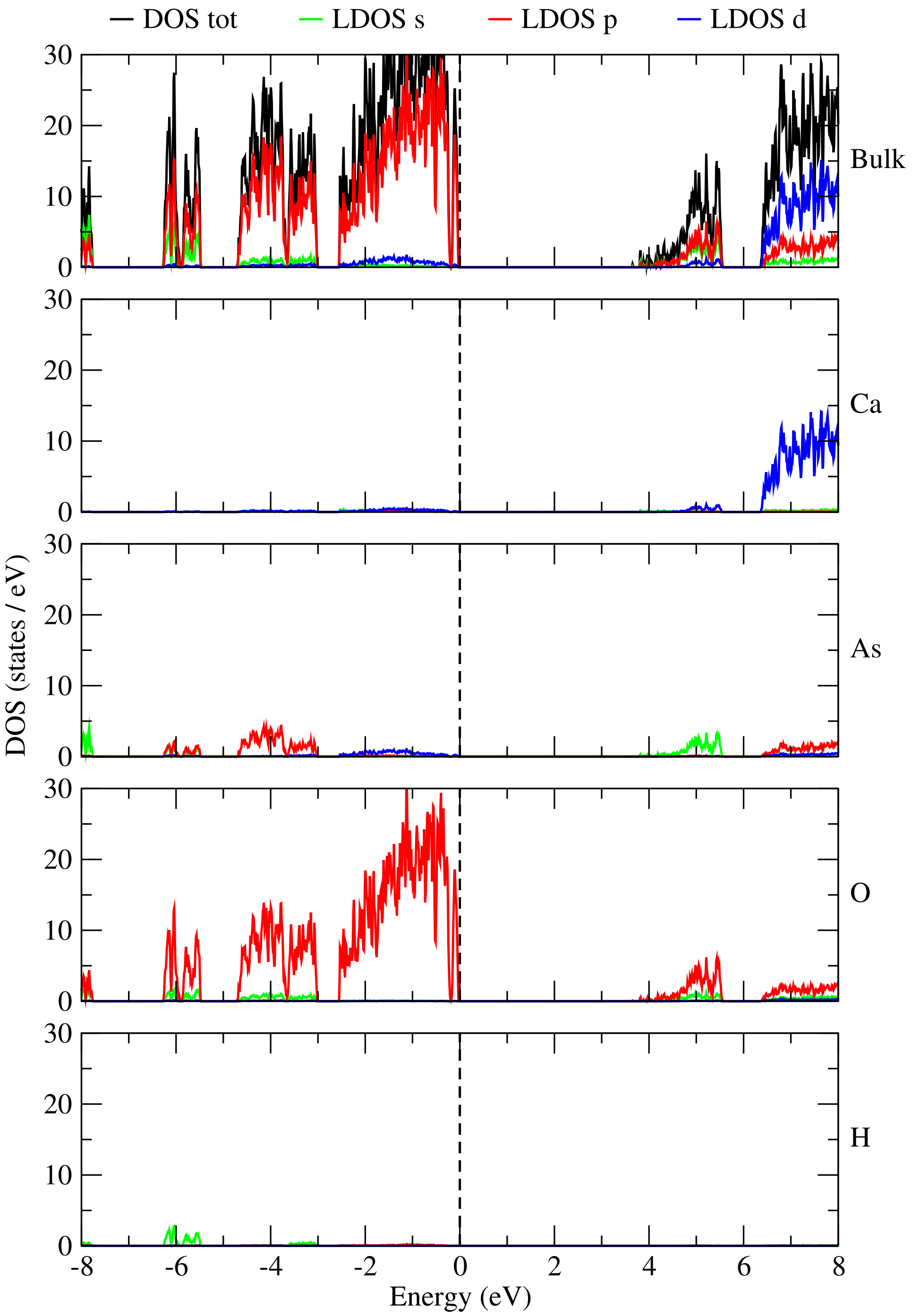

Figure S-31: Local density of states for the $CaHAsO_4$ bulk phase.

Table S-34: Calculated properties for the $CaHAsO_4$ bulk phase. Number of non-equivalent species, N; average distance for nearest neighbors, d_{NN}; effective coordination number, ECN; and net atomic charge, Q.

Non-equivalent species	N	d_{NN} (Å)	ECN (NNN)	Q (e^-)
Ca(I)	1	2.3223	6.2093	1.476 713
Ca(II)	1	2.2873	6.7830	1.473 330
Ca(III)	1	2.3941	8.5032	1.452 822
Ca(IV)	1	2.3979	8.1126	1.469 017
As(I)	1	1.6945	3.9527	1.717 466
As(II)	1	1.6928	3.9517	1.710 062
As(III)	1	1.6886	4.0046	1.698 151
As(IV)	1	1.6841	3.9302	1.713 670
O(I)	1	1.6841	1.0073	−0.925 007
O(II)	1	1.4902	1.6419	−0.909 244
O(III)	1	1.4384	1.4659	−0.872 889
O(IV)	1	1.0553	1.0000	−0.784 077
O(V)	1	1.0453	1.0000	−0.801 110
O(VI)	1	1.0498	1.0000	−0.796 080
O(VII)	1	1.7195	1.0079	−0.949 793
O(VIII)	1	1.7201	1.0084	−0.952 493
O(IX)	1	1.6972	1.0029	−0.923 352
O(X)	1	1.6975	1.0040	−0.935 365
O(XI)	1	1.6945	1.0225	−0.951 259
O(XII)	1	1.6928	1.0117	−0.941 628
O(XIII)	1	1.5216	1.7722	−0.866 389
O(XIV)	1	1.5341	1.8235	−0.863 259
O(XV)	1	1.6886	1.0053	−0.905 199
O(XVI)	1	1.0445	1.0000	−0.812 916
H(I)	1	1.0445	1.0021	0.369 146
H(II)	1	1.0498	1.0010	0.376 535
H(III)	1	1.0453	1.0005	0.373 951
H(IV)	1	1.0553	1.0150	0.359 198

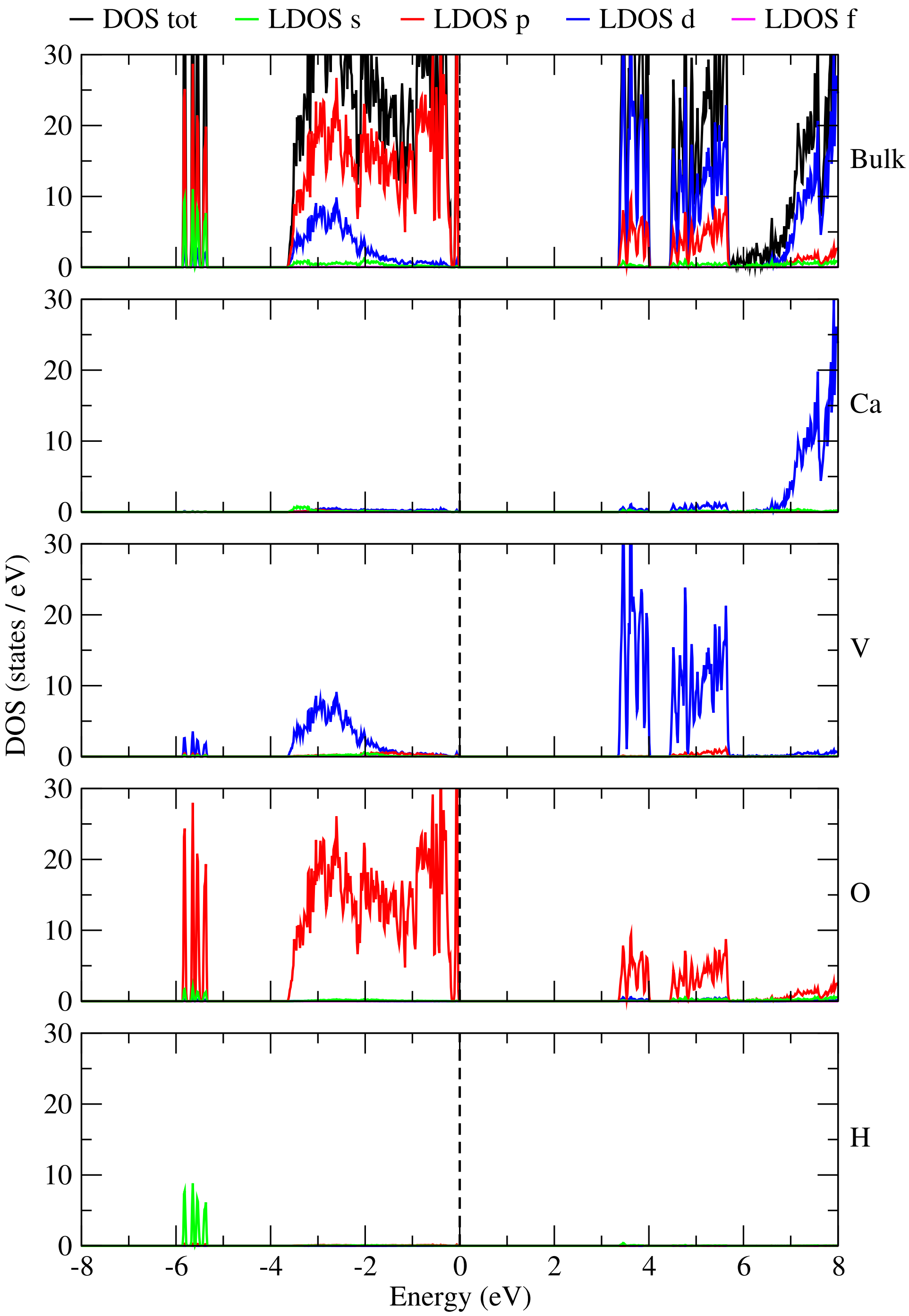

Figure S-32: Local density of states for the $CaHVO_4$ bulk phase.

Table S-35: Calculated properties for the $CaHVO_4$ bulk phase. Number of non-equivalent species, N; average distance for nearest neighbors, d_{NN}; effective coordination number, ECN; and net atomic charge, Q.

Non-equivalent species	N	d_{NN} (Å)	ECN (NNN)	Q (e^-)
Ca(I)	1	2.3423	6.5777	1.521 344
Ca(II)	1	2.3247	7.0594	1.518 171
Ca(III)	1	2.3779	8.6211	1.508 707
Ca(IV)	1	2.4455	8.2926	1.516 440
V(I)	1	1.6861	3.8613	2.021 795
V(II)	1	1.6729	3.8438	2.017 152
V(III)	1	1.6830	3.9303	1.993 481
V(IV)	1	1.6828	3.8044	1.997 023
O(I)	1	1.6828	1.0091	−0.993 535
O(II)	1	1.6149	1.9314	−1.020 406
O(III)	1	1.5402	1.8380	−0.943 381
O(IV)	1	1.0248	1.0000	−0.921 128
O(V)	1	1.0137	1.0000	−0.957 547
O(VI)	1	1.0133	1.0000	−0.953 614
O(VII)	1	1.7126	1.0090	−1.034 175
O(VIII)	1	1.7218	1.0094	−1.052 991
O(IX)	1	1.6886	1.0010	−0.988 869
O(X)	1	1.6913	1.0014	−1.010 327
O(XI)	1	1.6861	1.0100	−0.990 950
O(XII)	1	1.6729	1.0051	−0.956 821
O(XIII)	1	1.6467	1.9925	−0.924 823
O(XIV)	1	1.6510	1.9941	−0.913 094
O(XV)	1	1.6899	1.0025	−0.974 857
O(XVI)	1	1.0116	1.0000	−0.979 686
H(I)	1	1.0116	1.0000	0.379 454
H(II)	1	1.0133	1.0000	0.390 346
H(III)	1	1.0137	1.0000	0.384 761
H(IV)	1	1.0248	1.0001	0.367 530

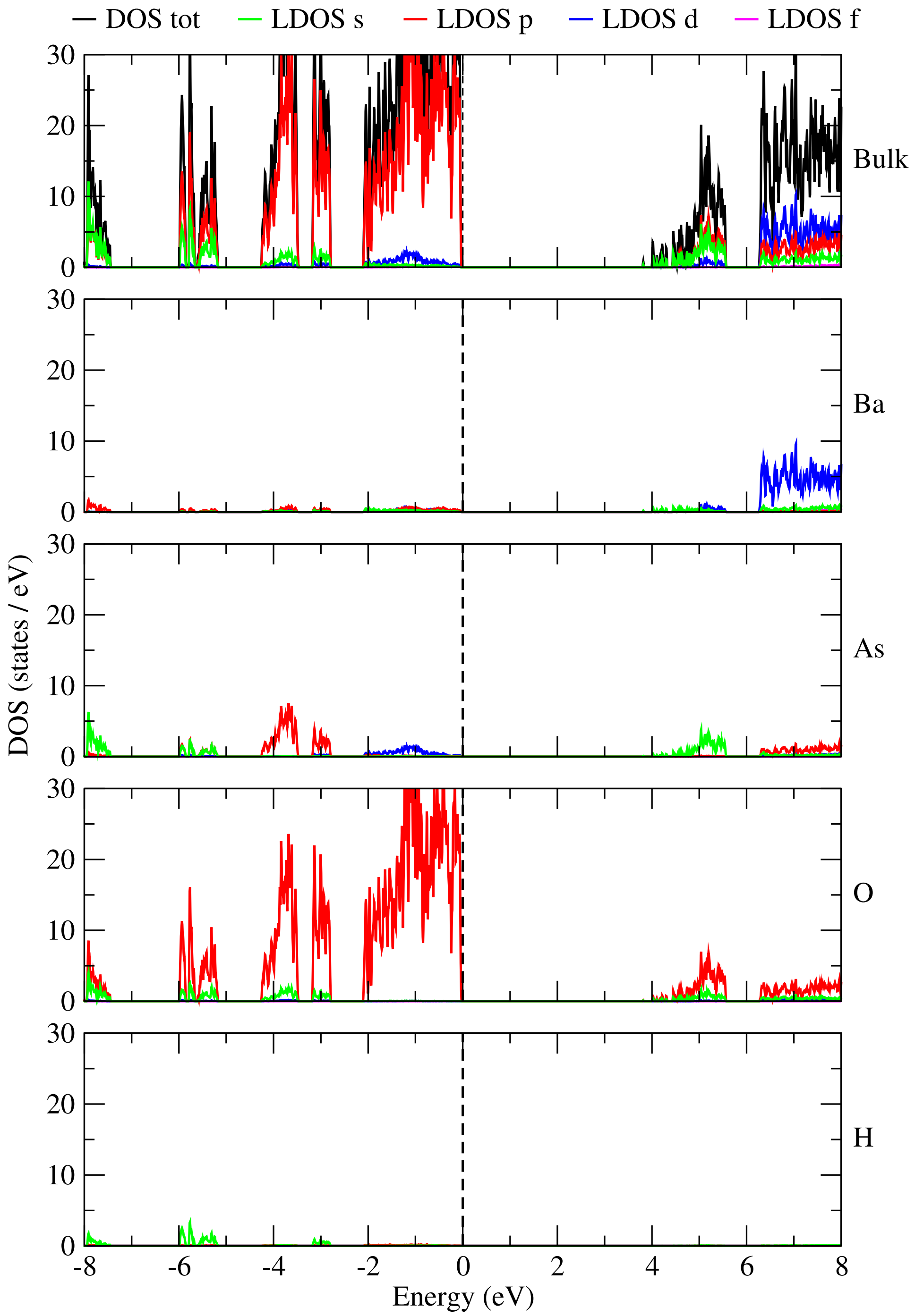

Figure S-33: Local density of states for the $BaHAsO_4$ bulk phase.

Table S-36: Calculated properties for the $BaHAsO_4$ bulk phase. Number of non-equivalent species, N; average distance for nearest neighbors, d_{NN}; effective coordination number, ECN; and net atomic charge, Q.

Non-equivalent species	N	d_{NN} (Å)	ECN (NNN)	Q (e^-)
Ba(I)	1	2.6651	7.4993	1.506 102
Ba(II)	1	2.6186	7.9965	1.500 487
Ba(III)	1	2.7017	9.5393	1.488 486
Ba(IV)	1	2.6732	8.5269	1.504 399
As(I)	1	1.6939	3.9393	1.667 201
As(II)	1	1.6946	3.9386	1.670 378
As(III)	1	1.6859	3.9598	1.676 234
As(IV)	1	1.6845	3.9314	1.681 925
O(I)	1	1.6845	1.0000	−0.918 306
O(II)	1	1.5469	1.8240	−0.896 343
O(III)	1	1.5140	1.7275	−0.876 339
O(IV)	1	1.0423	1.0000	−0.791 410
O(V)	1	1.0255	1.0000	−0.801 330
O(VI)	1	1.0301	1.0000	−0.800 827
O(VII)	1	1.7173	1.0000	−0.941 852
O(VIII)	1	1.7155	1.0000	−0.937 373
O(IX)	1	1.6939	1.0000	−0.922 687
O(X)	1	1.6946	1.0000	−0.931 549
O(XI)	1	1.7019	1.0000	−0.941 428
O(XII)	1	1.7019	1.0000	−0.946 878
O(XIII)	1	1.5745	1.8685	−0.895 891
O(XIV)	1	1.6004	1.9296	−0.892 335
O(XV)	1	1.6859	1.0000	−0.897 516
O(XVI)	1	1.0361	1.0000	−0.804 516
H(I)	1	1.0361	1.0002	0.378 800
H(II)	1	1.0301	1.0000	0.374 686
H(III)	1	1.0255	1.0000	0.379 949
H(IV)	1	1.0423	1.0008	0.367 932

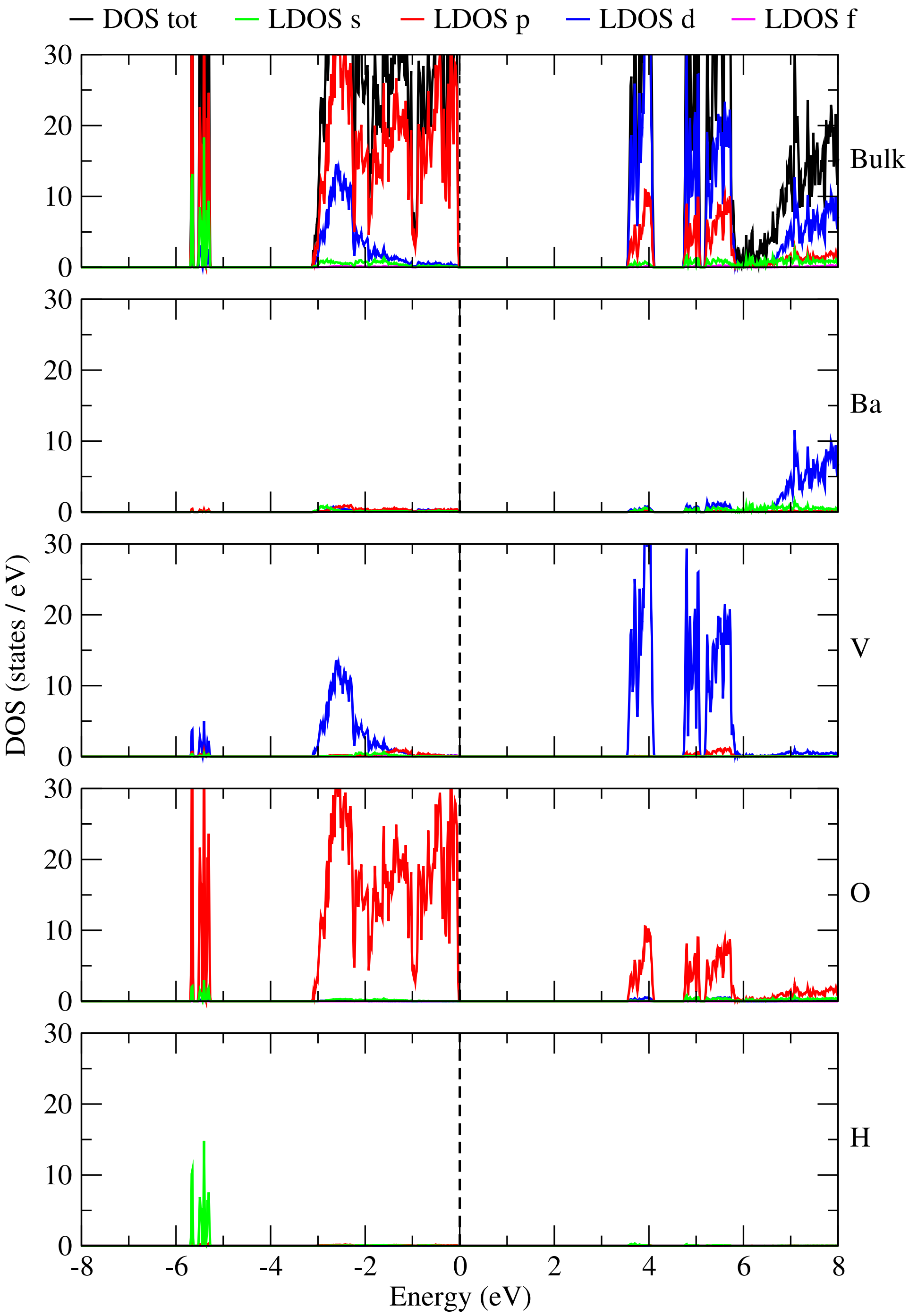

Figure S-34: Local density of states for the $BaHVO_4$ bulk phase.

Table S-37: Calculated properties for the $BaHVO_4$ bulk phase. Number of non-equivalent species, N; average distance for nearest neighbors, d_{NN}; effective coordination number, ECN; and net atomic charge, Q.

Non-equivalent species	N	d_{NN} (Å)	ECN (NNN)	Q (e^-)
Ba(I)	1	2.6881	7.9901	1.558 546
Ba(II)	1	2.6585	8.3707	1.554 416
Ba(III)	1	2.7116	9.6097	1.544 934
Ba(IV)	1	2.6921	8.9347	1.557 521
V(I)	1	1.6882	3.8501	1.982 806
V(II)	1	1.6892	3.8524	1.984 341
V(III)	1	1.6783	3.8673	1.975 544
V(IV)	1	1.6701	3.8351	1.985 305
O(I)	1	1.6701	1.0000	−0.961 949
O(II)	1	1.6509	1.9779	−1.001 416
O(III)	1	1.5607	1.8516	−0.974 066
O(IV)	1	1.0239	1.0000	−0.957 803
O(V)	1	1.0089	1.0000	−0.974 462
O(VI)	1	1.0125	1.0000	−0.972 995
O(VII)	1	1.7039	1.0000	−1.017 581
O(VIII)	1	1.7056	1.0000	−1.018 931
O(IX)	1	1.6882	1.0000	−0.997 459
O(X)	1	1.6906	1.0000	−1.014 768
O(XI)	1	1.6927	1.0000	−0.992 234
O(XII)	1	1.6892	1.0000	−0.989 859
O(XIII)	1	1.6282	1.9712	−0.962 397
O(XIV)	1	1.6617	1.9929	−0.960 142
O(XV)	1	1.6783	1.0000	−0.950 139
O(XVI)	1	1.0098	1.0000	−0.972 143
H(I)	1	1.0098	1.0000	0.395 855
H(II)	1	1.0125	1.0000	0.397 010
H(III)	1	1.0089	1.0000	0.396 591
H(IV)	1	1.0239	1.0000	0.385 472

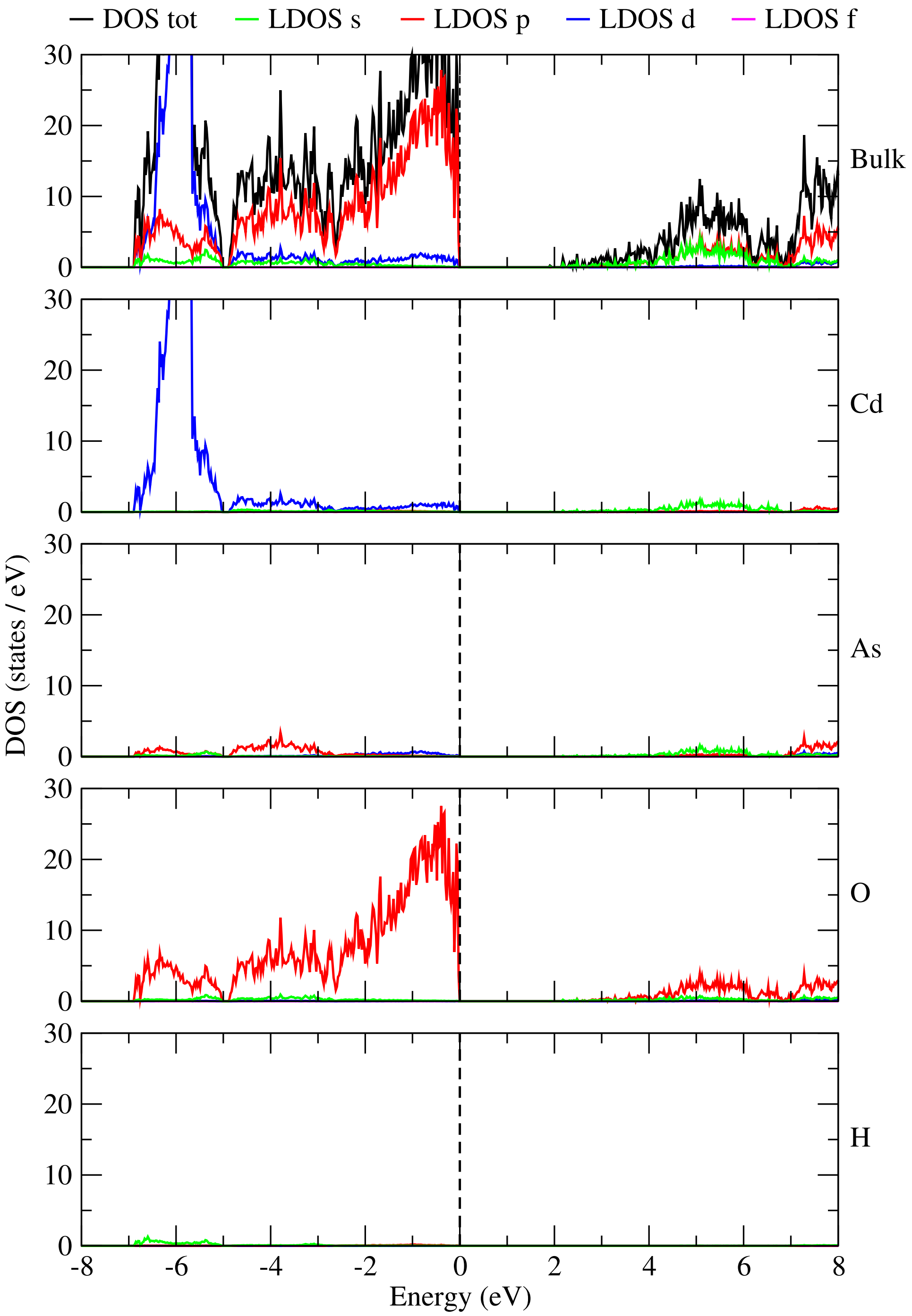

Figure S-35: Local density of states for the $CdHAsO_4$ bulk phase.

Table S-38: Calculated properties for the $CdHAsO_4$ bulk phase. Number of non-equivalent species, N; average distance for nearest neighbors, d_{NN}; effective coordination number, ECN; and net atomic charge, Q.

Non-equivalent species	N	d_{NN} (Å)	ECN (NNN)	Q (e^-)
Cd(I)	1	2.2497	6.1366	1.183 011
Cd(II)	1	2.2565	6.4102	1.193 549
Cd(III)	1	2.2835	7.9670	1.173 924
Cd(IV)	1	2.3416	7.4957	1.208 980
As(I)	1	1.6999	3.9717	1.690 922
As(II)	1	1.6881	3.9440	1.684 153
As(III)	1	1.6978	4.0237	1.695 191
As(IV)	1	1.6936	3.9451	1.707 171
O(I)	1	1.6936	1.0370	−0.847 364
O(II)	1	1.4538	1.4666	−0.842 250
O(III)	1	1.3446	1.1451	−0.796 960
O(IV)	1	1.0793	1.0000	−0.747 869
O(V)	1	1.0475	1.0000	−0.750 611
O(VI)	1	1.0543	1.0000	−0.750 957
O(VII)	1	1.7343	1.0222	−0.851 835
O(VIII)	1	1.7345	1.0143	−0.843 410
O(IX)	1	1.6999	1.0105	−0.841 657
O(X)	1	1.7083	1.0129	−0.856 050
O(XI)	1	1.7053	1.0458	−0.846 913
O(XII)	1	1.6881	1.0332	−0.845 298
O(XIII)	1	1.5056	1.7180	−0.815 009
O(XIV)	1	1.5041	1.7484	−0.818 198
O(XV)	1	1.6978	1.0249	−0.818 808
O(XVI)	1	1.0542	1.0000	−0.750 849
H(I)	1	1.0542	1.0095	0.373 627
H(II)	1	1.0543	1.0020	0.376 498
H(III)	1	1.0475	1.0015	0.373 607
H(IV)	1	1.0793	1.2150	0.363 405

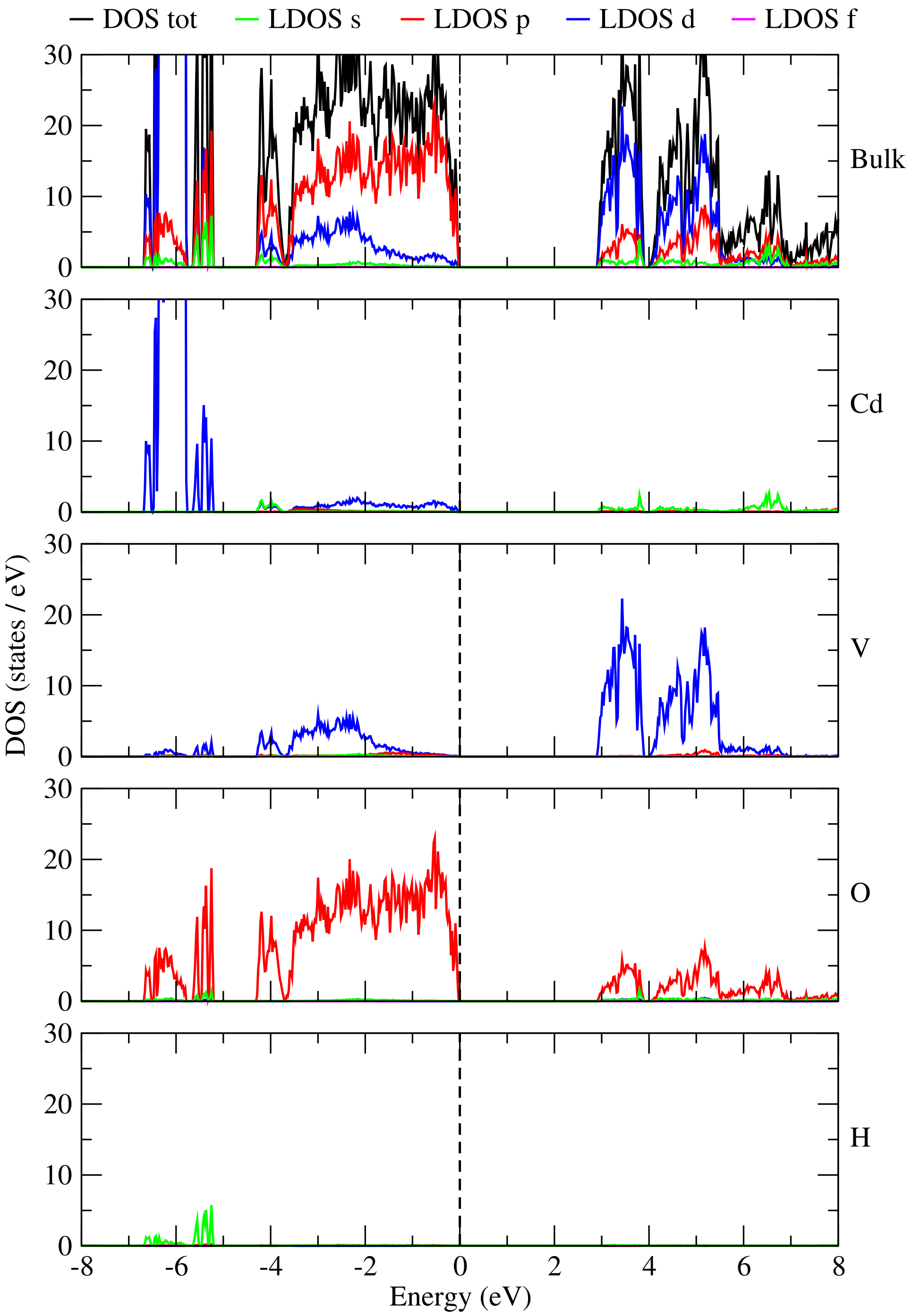

Figure S-36: Local density of states for the $CdHVO_4$ bulk phase.

Table S-39: Calculated properties for the $CdHVO_4$ bulk phase. Number of non-equivalent species, N; average distance for nearest neighbors, d_{NN}; effective coordination number, ECN; and net atomic charge, Q.

Non-equivalent species	N	d_{NN} (Å)	ECN (NNN)	Q (e^-)
Cd(I)	1	2.2468	6.3228	1.242 507
Cd(II)	1	2.2373	6.7699	1.232 080
Cd(III)	1	2.2520	6.0832	1.263 111
Cd(IV)	1	2.2723	6.4334	1.267 679
V(I)	1	1.6567	3.7208	1.997 493
V(II)	1	1.6479	3.7958	2.005 189
V(III)	1	1.6819	3.9081	2.028 364
V(IV)	1	1.6789	3.7405	1.994 527
O(I)	1	1.7007	1.0552	−0.928 628
O(II)	1	1.7169	1.0385	−0.949 517
O(III)	1	1.6792	2.0184	−0.876 898
O(IV)	1	1.0088	1.0000	−0.884 236
O(V)	1	1.0036	1.0000	−0.880 083
O(VI)	1	1.0130	1.0000	−0.880 532
O(VII)	1	1.7958	1.2013	−1.019 658
O(VIII)	1	1.7650	1.0741	−1.002 019
O(IX)	1	1.6571	1.0167	−0.888 828
O(X)	1	1.6728	2.0179	−0.938 075
O(XI)	1	1.6567	1.0210	−0.861 198
O(XII)	1	1.6479	1.0148	−0.844 983
O(XIII)	1	1.6279	1.9842	−0.884 086
O(XIV)	1	1.6789	2.0020	−0.880 316
O(XV)	1	1.7107	1.0327	−0.924 701
O(XVI)	1	1.0096	1.0000	−0.897 079
H(I)	1	1.0096	1.0000	0.378 500
H(II)	1	1.0130	1.0000	0.384 521
H(III)	1	1.0036	1.0000	0.378 941
H(IV)	1	1.0088	1.0000	0.367 926

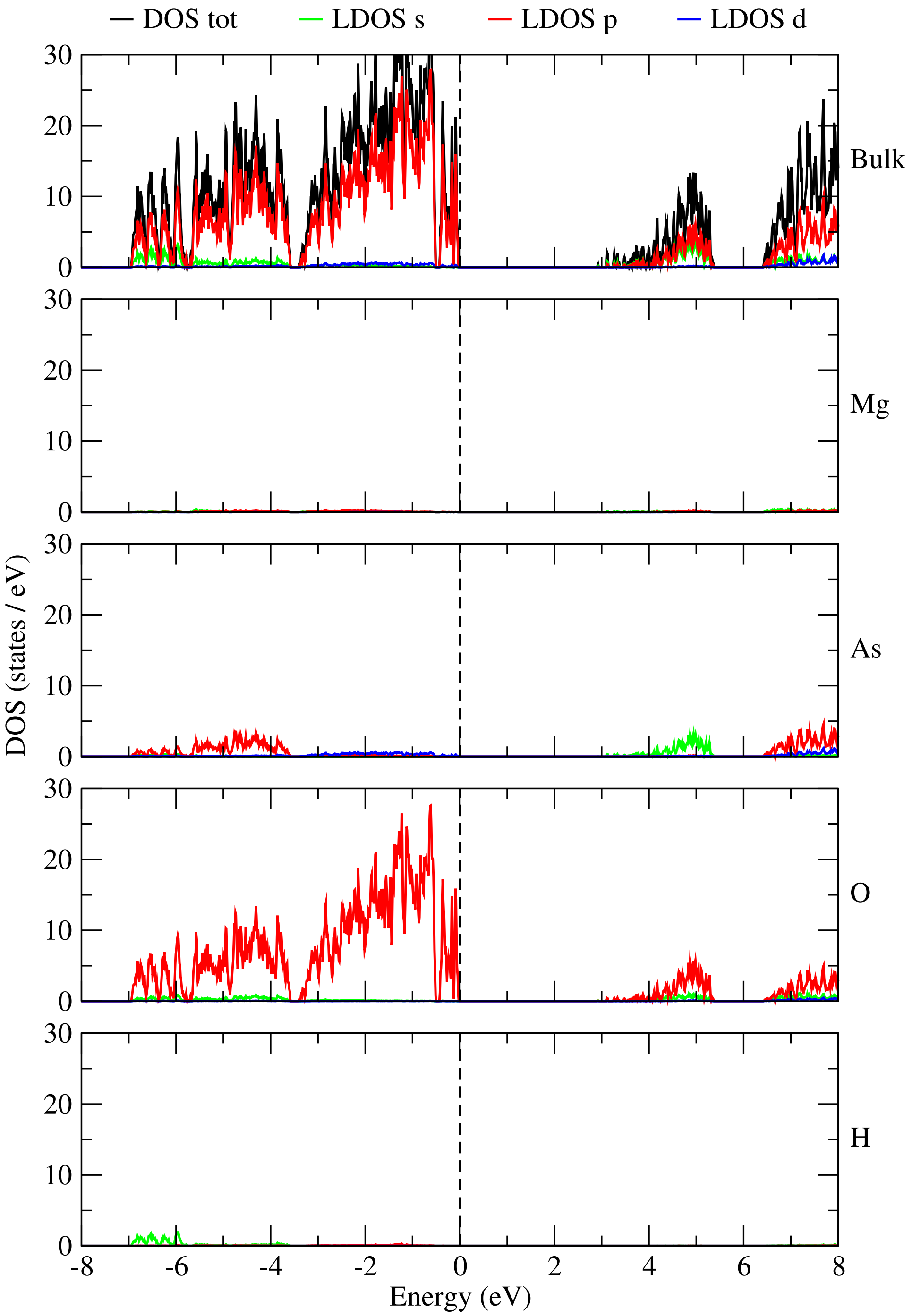

Figure S-37: Local density of states for the $MgHAsO_4$ bulk phase.

Table S-40: Calculated properties for the $MgHAsO_4$ bulk phase. Number of non-equivalent species, N; average distance for nearest neighbors, d_{NN}; effective coordination number, ECN; and net atomic charge, Q.

Non-equivalent species	N	d_{NN} (Å)	ECN (NNN)	Q (e^-)
Mg(I)	1	2.0118	5.8815	1.493 079
Mg(II)	1	1.9978	5.8231	1.476 629
Mg(III)	1	1.9894	5.4545	1.501 774
Mg(IV)	1	2.0519	5.9365	1.493 478
As(I)	1	1.6639	3.8978	1.809 601
As(II)	1	1.6525	3.9395	1.806 618
As(III)	1	1.7008	4.0574	1.838 656
As(IV)	1	1.6852	3.9600	1.797 113
O(I)	1	1.7036	1.9213	−0.986 577
O(II)	1	1.7095	1.6917	−0.982 348
O(III)	1	1.5267	1.8869	−0.891 818
O(IV)	1	1.0372	1.0000	−0.803 013
O(V)	1	1.0217	1.0000	−0.803 570
O(VI)	1	1.0790	1.0000	−0.815 016
O(VII)	1	1.7650	1.9438	−1.014 136
O(VIII)	1	1.7470	1.7272	−0.997 724
O(IX)	1	1.6722	1.4737	−0.974 438
O(X)	1	1.4350	1.4658	−0.947 461
O(XI)	1	1.6639	1.4190	−0.949 153
O(XII)	1	1.6525	1.3373	−0.923 213
O(XIII)	1	1.4150	1.3539	−0.910 225
O(XIV)	1	1.6008	2.0682	−0.913 656
O(XV)	1	1.7008	1.6338	−0.953 830
O(XVI)	1	1.0667	1.0000	−0.818 487
H(I)	1	1.0667	1.0240	0.364 821
H(II)	1	1.0790	1.0554	0.375 590
H(III)	1	1.0217	1.0000	0.370 005
H(IV)	1	1.0372	1.0004	0.357 304

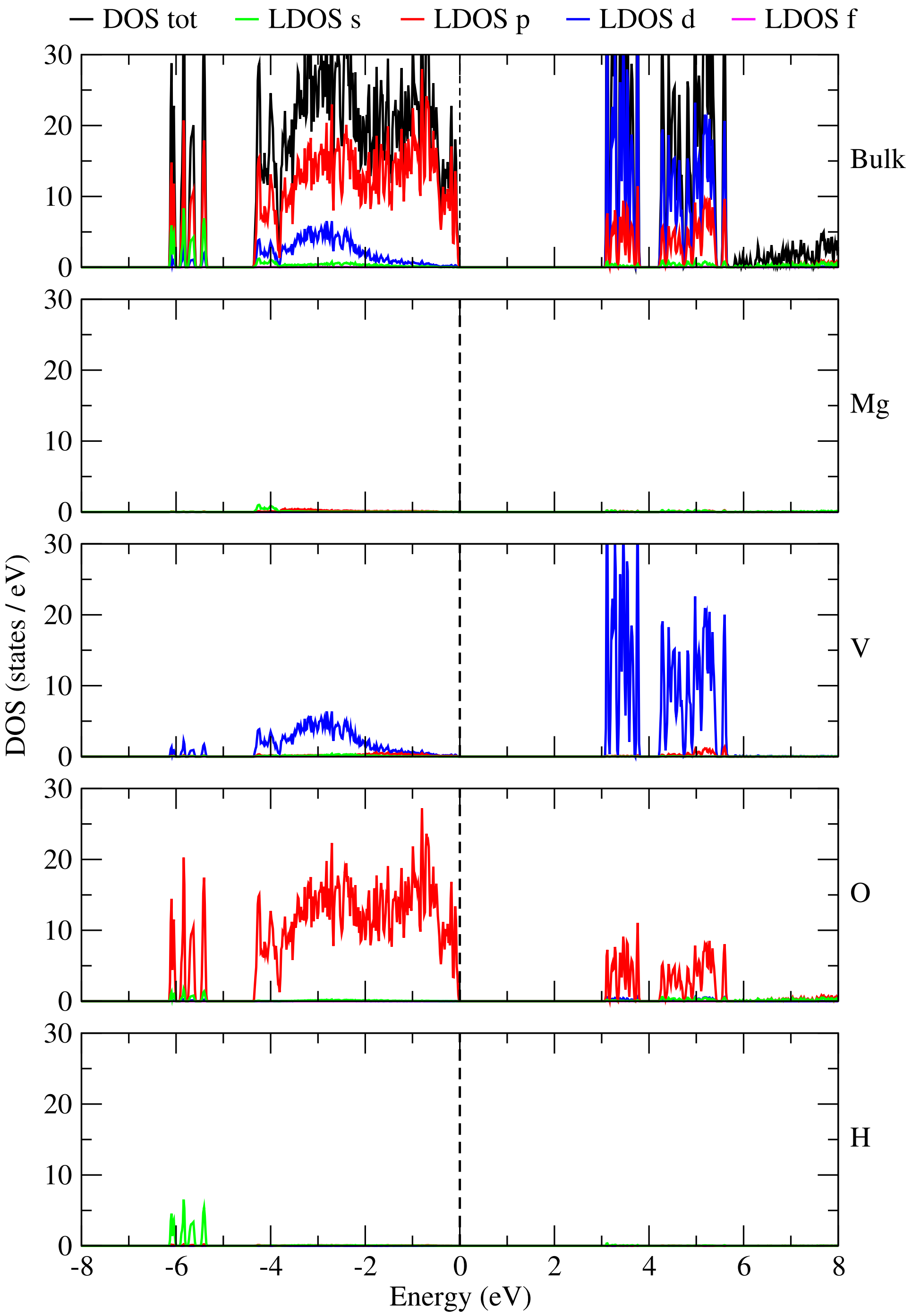

Figure S-38: Local density of states for the $MgHVO_4$ bulk phase.

Table S-41: Calculated properties for the $MgHVO_4$ bulk phase. Number of non-equivalent species, N; average distance for nearest neighbors, d_{NN}; effective coordination number, ECN; and net atomic charge, Q.

Non-equivalent species	N	d_{NN} (Å)	ECN (NNN)	Q (e^-)
Mg(I)	1	2.0677	6.2297	1.514 859
Mg(II)	1	2.0427	6.4794	1.508 156
Mg(III)	1	2.0448	6.1331	1.523 710
Mg(IV)	1	2.0683	6.3482	1.526 116
V(I)	1	1.6542	3.7234	2.063 978
V(II)	1	1.6445	3.7840	2.062 012
V(III)	1	1.6773	3.9181	2.086 168
V(IV)	1	1.6670	3.7099	2.048 829
O(I)	1	1.7034	1.8799	−1.046 668
O(II)	1	1.7165	1.9112	−1.049 519
O(III)	1	1.6670	2.0381	−0.920 916
O(IV)	1	0.9927	1.0000	−0.911 111
O(V)	1	1.0039	1.0000	−0.941 088
O(VI)	1	1.0090	1.0000	−0.934 492
O(VII)	1	1.7879	2.6513	−1.164 725
O(VIII)	1	1.7679	2.4375	−1.135 904
O(IX)	1	1.6571	1.2630	−0.967 530
O(X)	1	1.6827	2.2702	−1.001 835
O(XI)	1	1.6542	1.2163	−0.936 053
O(XII)	1	1.6445	1.1745	−0.907 663
O(XIII)	1	1.6666	2.0959	−0.925 753
O(XIV)	1	1.6835	2.3062	−0.936 596
O(XV)	1	1.7122	1.6964	−1.038 906
O(XVI)	1	1.0041	1.0000	−0.985 637
H(I)	1	1.0041	1.0000	0.368 817
H(II)	1	1.0090	1.0000	0.378 090
H(III)	1	1.0039	1.0000	0.370 872
H(IV)	1	0.9927	1.0000	0.352 786

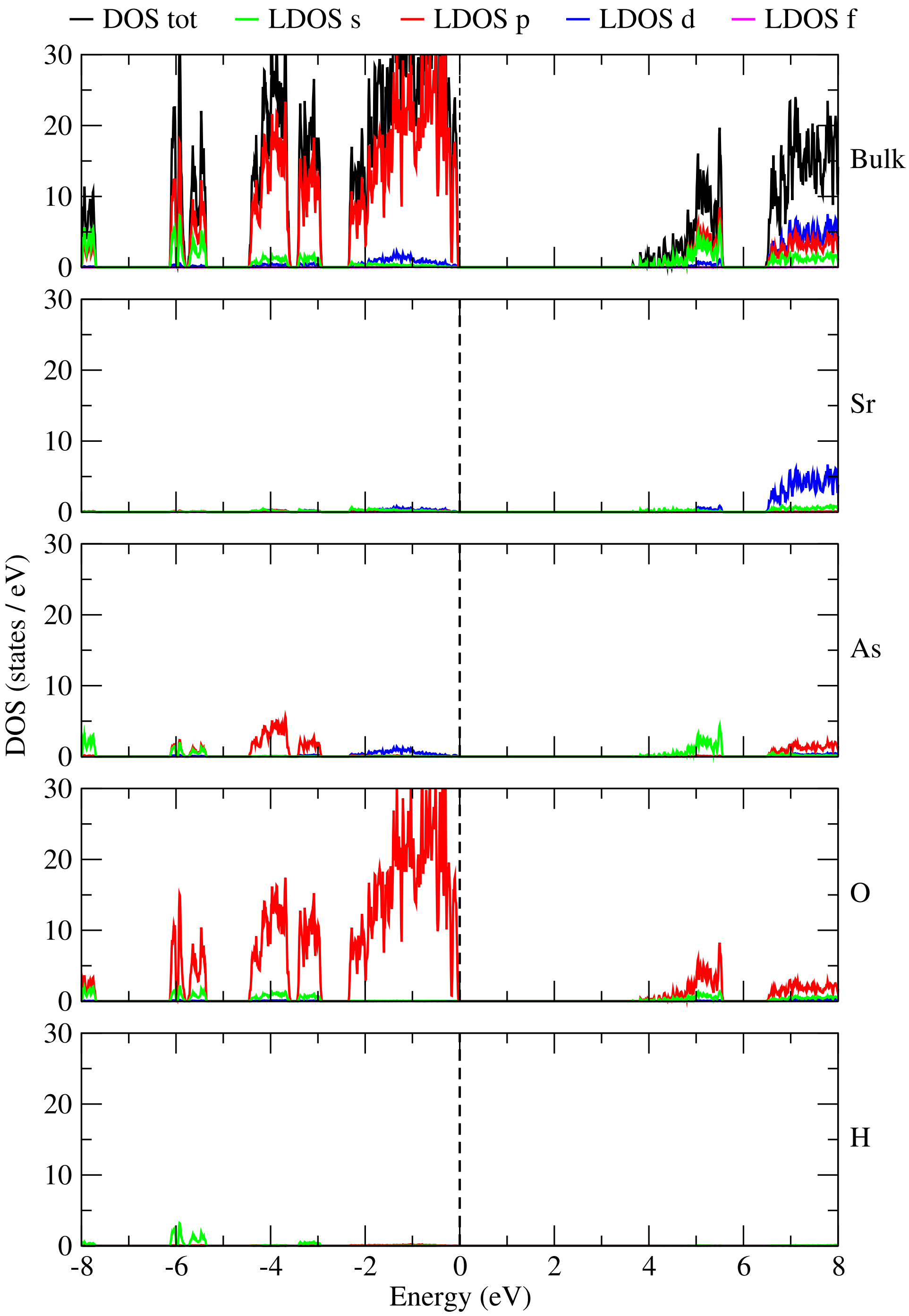

Figure S-39: Local density of states for the $SrHAsO_4$ bulk phase.

Table S-42: Calculated properties for the $SrHAsO_4$ bulk phase. Number of non-equivalent species, N; average distance for nearest neighbors, d_{NN}; effective coordination number, ECN; and net atomic charge, Q.

Non-equivalent species	N	d_{NN} (Å)	ECN (NNN)	Q (e^-)
Sr(I)	1	2.4816	6.3541	1.525 962
Sr(II)	1	2.4461	7.0845	1.522 782
Sr(III)	1	2.5648	9.0963	1.497 596
Sr(IV)	1	2.5425	8.2687	1.512 310
As(I)	1	1.6957	3.9470	1.698 107
As(II)	1	1.6961	3.9500	1.693 533
As(III)	1	1.6861	3.9747	1.682 735
As(IV)	1	1.6841	3.9205	1.697 107
O(I)	1	1.6841	1.0002	−0.931 735
O(II)	1	1.5360	1.7893	−0.914 305
O(III)	1	1.4953	1.6675	−0.884 508
O(IV)	1	1.0454	1.0000	−0.789 260
O(V)	1	1.0359	1.0000	−0.809 252
O(VI)	1	1.0433	1.0000	−0.806 774
O(VII)	1	1.7179	1.0001	−0.957 923
O(VIII)	1	1.7181	1.0002	−0.959 832
O(IX)	1	1.6957	1.0001	−0.932 062
O(X)	1	1.6961	1.0002	−0.942 687
O(XI)	1	1.6970	1.0013	−0.961 130
O(XII)	1	1.6963	1.0006	−0.956 200
O(XIII)	1	1.5331	1.7888	−0.878 496
O(XIV)	1	1.5660	1.8877	−0.874 126
O(XV)	1	1.6861	1.0001	−0.907 224
O(XVI)	1	1.0359	1.0000	−0.818 228
H(I)	1	1.0359	1.0003	0.373 690
H(II)	1	1.0433	1.0004	0.377 784
H(III)	1	1.0359	1.0001	0.379 026
H(IV)	1	1.0454	1.0019	0.363 109

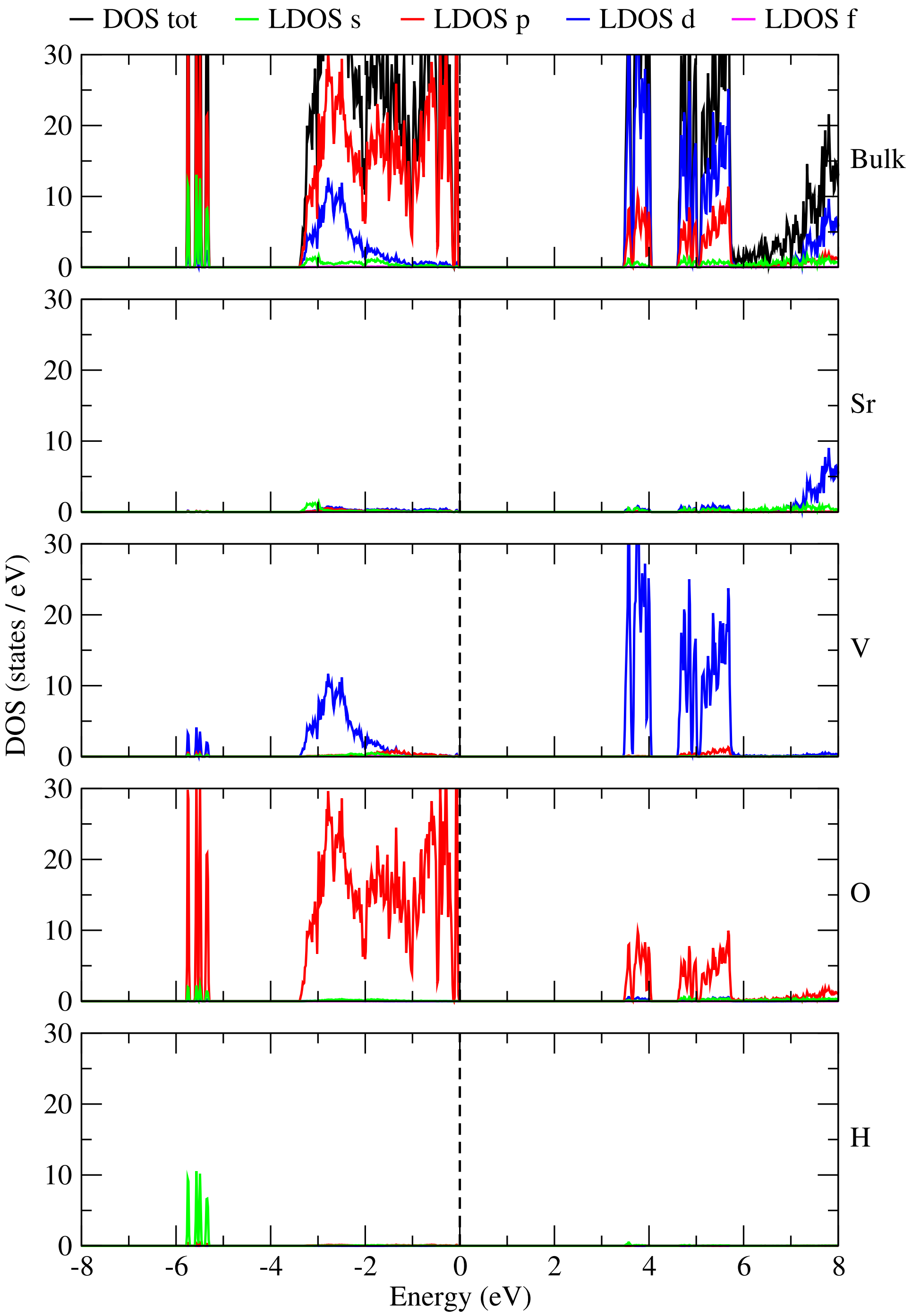

Figure S-40: Local density of states for the $SrHVO_4$ bulk phase.

Table S-43: Calculated properties for the $SrHVO_4$ bulk phase. Number of non-equivalent species, N; average distance for nearest neighbors, d_{NN}; effective coordination number, ECN; and net atomic charge, Q.

Non-equivalent species	N	d_{NN} (Å)	ECN (NNN)	Q (e^-)
Sr(I)	1	2.5023	7.0128	1.576 566
Sr(II)	1	2.4817	7.5555	1.571 914
Sr(III)	1	2.5895	9.0177	1.558 686
Sr(IV)	1	2.5852	8.5534	1.565 257
V(I)	1	1.6871	3.8623	2.011 389
V(II)	1	1.6810	3.8593	2.009 238
V(III)	1	1.6834	3.8762	1.983 373
V(IV)	1	1.6775	3.8075	1.992 108
O(I)	1	1.6775	1.0002	−0.991 466
O(II)	1	1.6803	1.9921	−1.024 412
O(III)	1	1.5860	1.9069	−0.974 924
O(IV)	1	1.0205	1.0000	−0.948 840
O(V)	1	1.0118	1.0000	−0.973 212
O(VI)	1	1.0145	1.0000	−0.969 201
O(VII)	1	1.7083	1.0001	−1.040 737
O(VIII)	1	1.7127	1.0001	−1.049 582
O(IX)	1	1.6910	1.0000	−1.009 516
O(X)	1	1.6923	1.0001	−1.026 510
O(XI)	1	1.6871	1.0004	−1.003 018
O(XII)	1	1.6810	1.0002	−0.989 809
O(XIII)	1	1.6286	1.9795	−0.937 619
O(XIV)	1	1.6586	1.9957	−0.925 932
O(XV)	1	1.6834	1.0000	−0.970 857
O(XVI)	1	1.0064	1.0000	−0.983 755
H(I)	1	1.0064	1.0000	0.385 722
H(II)	1	1.0145	1.0000	0.396 589
H(III)	1	1.0118	1.0000	0.393 303
H(IV)	1	1.0205	1.0000	0.375 245

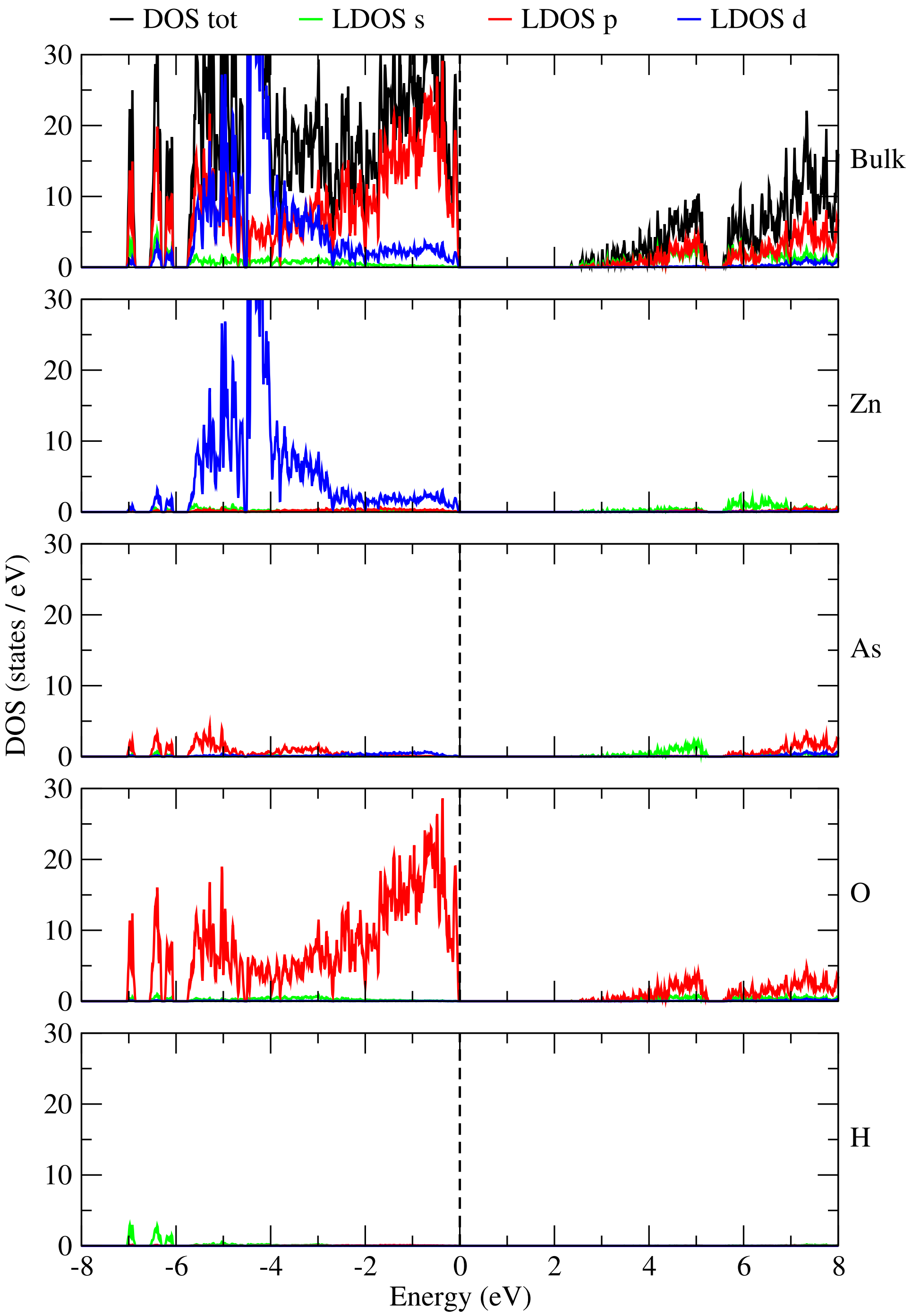

Figure S-41: Local density of states for the $ZnHAsO_4$ bulk phase.

Table S-44: Calculated properties for the $ZnHAsO_4$ bulk phase. Number of non-equivalent species, N; average distance for nearest neighbors, d_{NN}; effective coordination number, ECN; and net atomic charge, Q.

Non-equivalent species	N	d_{NN} (Å)	ECN (NNN)	Q (e^-)
Zn(I)	1	1.9295	3.7150	1.061 941
Zn(II)	1	1.9038	3.6778	1.080 350
Zn(III)	1	1.9442	3.9717	1.032 655
Zn(IV)	1	1.9263	3.8955	1.070 643
As(I)	1	1.6973	4.0159	1.684 467
As(II)	1	1.6908	3.9679	1.700 480
As(III)	1	1.6847	3.9943	1.684 493
As(IV)	1	1.6930	3.9862	1.749 261
O(I)	1	1.6930	1.7635	−0.809 225
O(II)	1	1.0507	1.0000	−0.716 834
O(III)	1	1.4749	1.6745	−0.860 278
O(IV)	1	1.0437	1.0000	−0.707 784
O(V)	1	1.0280	1.0000	−0.722 465
O(VI)	1	0.9879	1.0000	−0.721 548
O(VII)	1	1.6973	1.7152	−0.803 320
O(VIII)	1	1.7432	2.1554	−0.836 798
O(IX)	1	1.6987	1.6842	−0.781 615
O(X)	1	1.6933	1.7129	−0.815 384
O(XI)	1	1.7470	2.5446	−0.837 995
O(XII)	1	1.6908	1.6982	−0.803 285
O(XIII)	1	1.6847	1.7600	−0.775 232
O(XIV)	1	1.5457	1.9910	−0.861 047
O(XV)	1	1.7211	2.0639	−0.778 311
O(XVI)	1	1.5388	2.0368	−0.802 186
H(I)	1	1.0507	1.0005	0.408 868
H(II)	1	0.9879	1.0000	0.394 692
H(III)	1	1.0280	1.0001	0.375 790
H(IV)	1	1.0437	1.0033	0.389 667

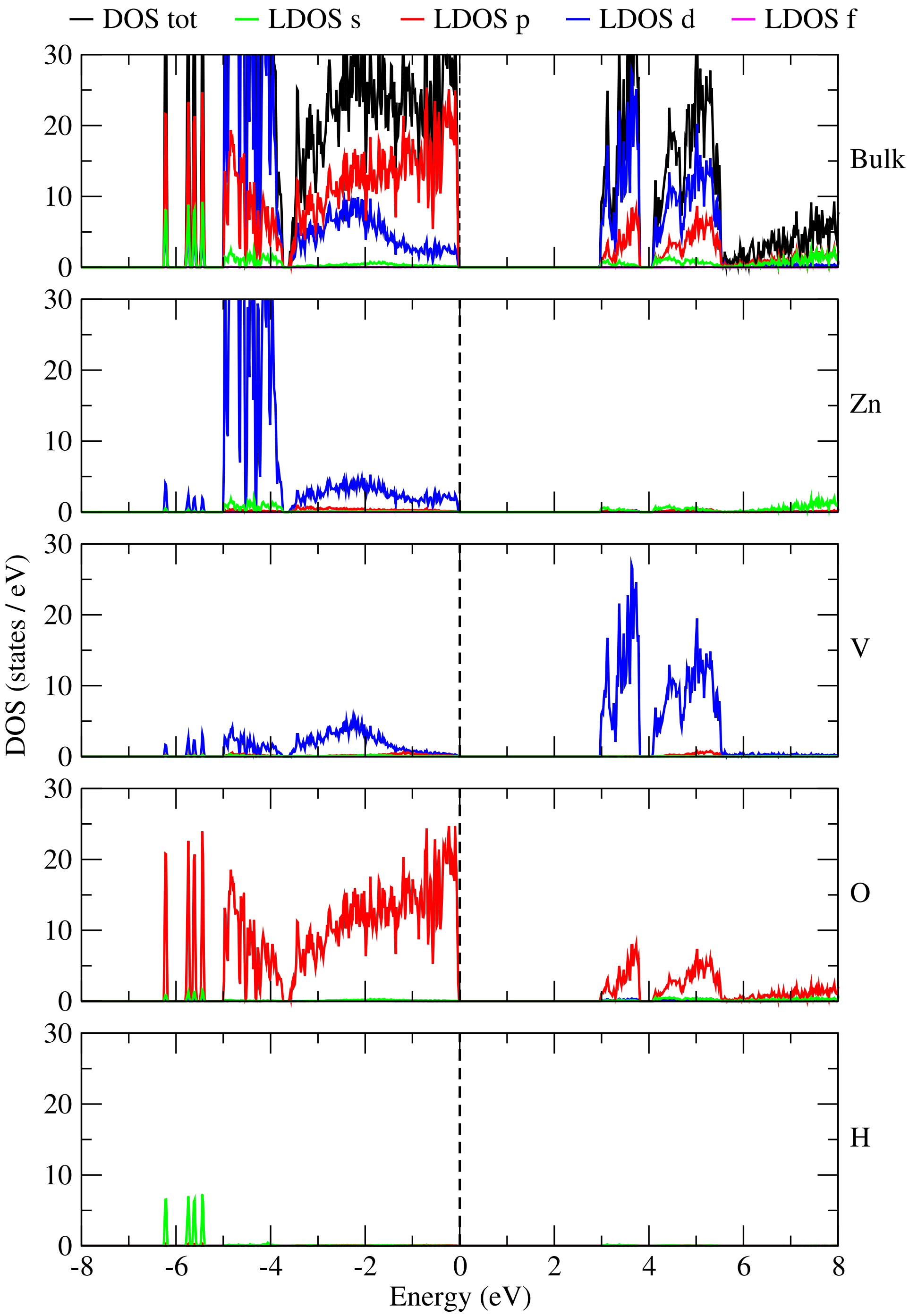

Figure S-42: Local density of states for the $ZnHVO_4$ bulk phase.

Table S-45: Calculated properties for the $ZnHVO_4$ bulk phase. Number of non-equivalent species, N; average distance for nearest neighbors, d_{NN}; effective coordination number, ECN; and net atomic charge, Q.

Non-equivalent species	N	d_{NN} (Å)	ECN (NNN)	Q (e^-)
Zn(I)	1	1.9618	4.4889	1.158 295
Zn(II)	1	1.9444	4.8537	1.160 196
Zn(III)	1	1.9517	3.9744	1.151 251
Zn(IV)	1	1.9503	4.3451	1.181 699
V(I)	1	1.6512	3.8082	2.018 671
V(II)	1	1.6597	3.7780	2.043 683
V(III)	1	1.6784	3.9729	2.019 698
V(IV)	1	1.6821	3.8010	2.028 779
O(I)	1	1.6926	1.6351	−0.891 893
O(II)	1	1.6395	2.3760	−0.923 336
O(III)	1	1.6877	2.4251	−0.917 365
O(IV)	1	1.0063	1.0000	−0.811 261
O(V)	1	0.9967	1.0000	−0.875 898
O(VI)	1	0.9949	1.0000	−0.883 735
O(VII)	1	1.7677	2.3505	−0.953 904
O(VIII)	1	1.7616	2.3025	−0.976 520
O(IX)	1	1.6904	1.6405	−0.894 682
O(X)	1	1.6769	1.6021	−0.919 166
O(XI)	1	1.6512	1.5332	−0.847 290
O(XII)	1	1.6597	1.5113	−0.846 795
O(XIII)	1	1.6784	2.3892	−0.877 127
O(XIV)	1	1.6821	2.3197	−0.884 127
O(XV)	1	1.7583	2.5040	−0.951 824
O(XVI)	1	1.0135	1.0000	−0.889 179
H(I)	1	1.0135	1.0000	0.418 049
H(II)	1	0.9949	1.0000	0.401 017
H(III)	1	0.9967	1.0000	0.385 443
H(IV)	1	1.0063	1.0000	0.377 321

S-6.3 γ-$Ca_3(PO_4)_2$-based Materials

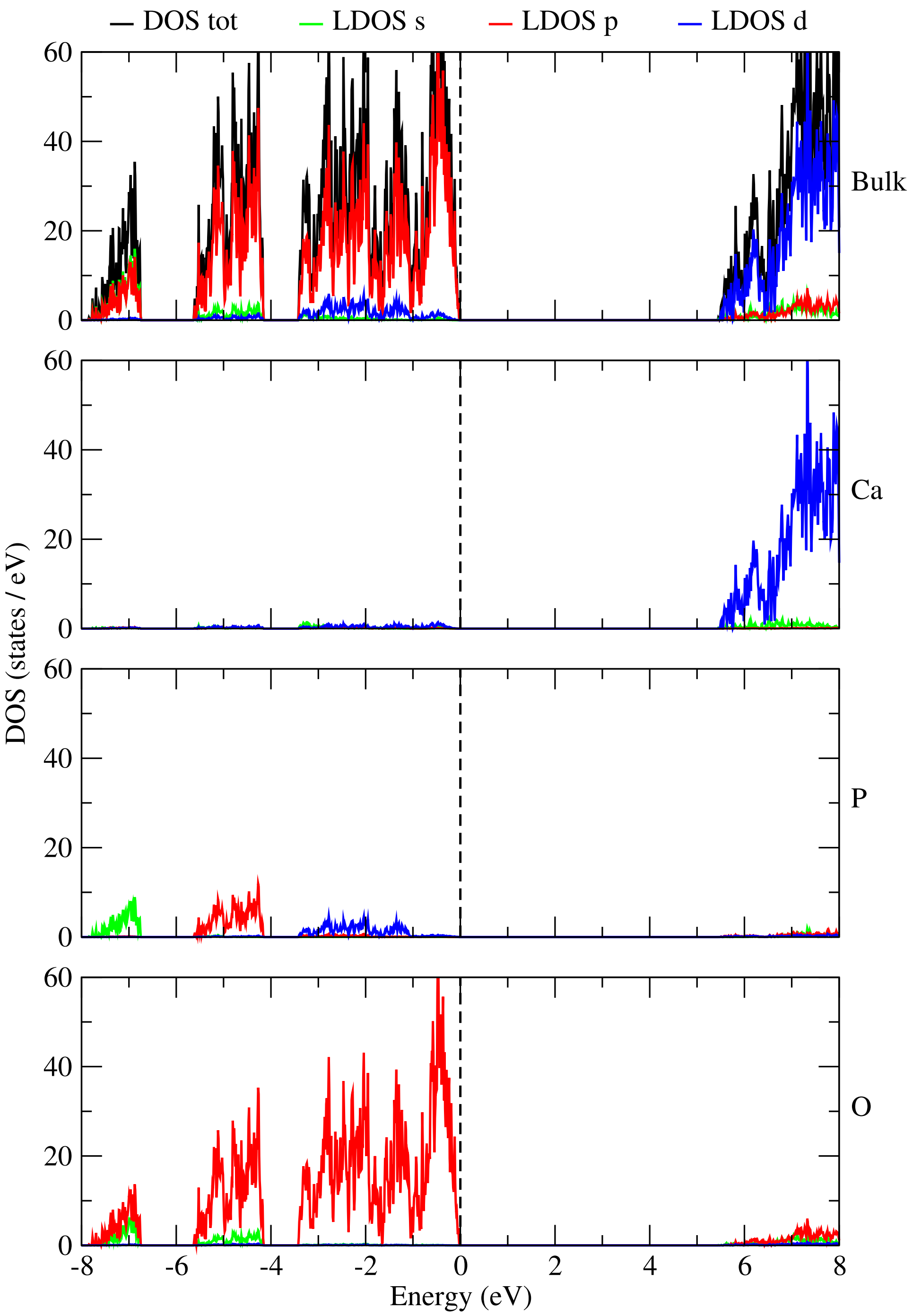

Figure S-43: Local density of states for the γ-$Ca_3(PO_4)_2$ bulk phase.

Table S-46: Calculated properties for the γ-$Ca_3(PO_4)_2$ bulk phase. Number of non-equivalent species, N; average distance for nearest neighbors, d_{NN}; effective coordination number, ECN; and net atomic charge, Q.

Non-equivalent species	N	d_{NN} (Å)	ECN (NNN)	Q (e^-)
Ca(I)	3	2.4517	7.3426	1.511 011
Ca(II)	6	2.2353	9.2599	1.477 696
P(I)	6	1.5403	3.9935	1.538 429
O(I)	6	1.5403	1.0009	−0.910 610
O(II)	18	1.5654	1.0000	−0.953 674

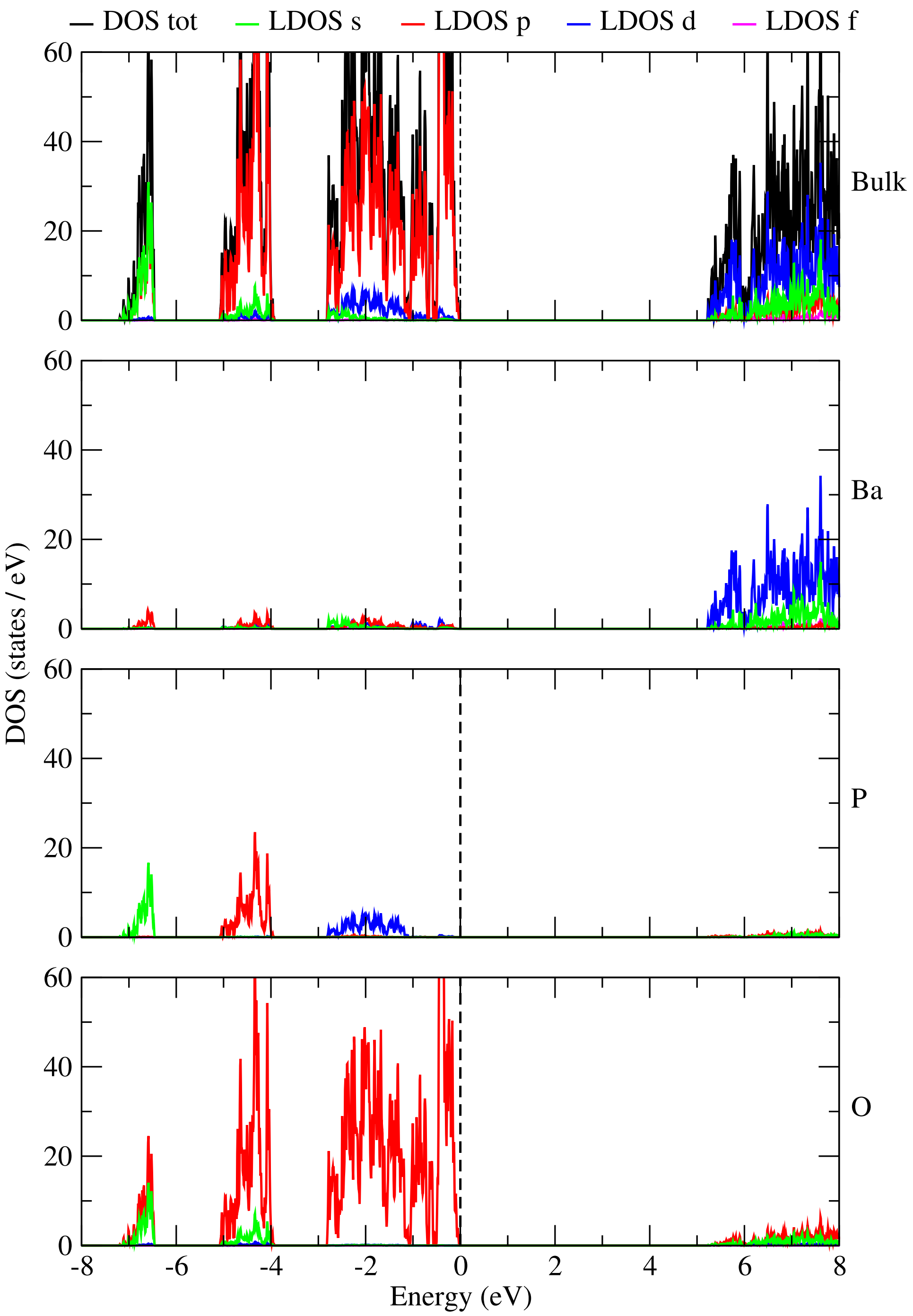

Figure S-44: Local density of states for the γ-$Ba_3(PO_4)_2$ bulk phase.

Table S-47: Calculated properties for the γ-$Ba_3(PO_4)_2$ bulk phase. Number of non-equivalent species, N; average distance for nearest neighbors, d_{NN}; effective coordination number, ECN; and net atomic charge, Q.

Non-equivalent species	N	d_{NN} (Å)	ECN (NNN)	Q (e^-)
Ba(I)	3	2.7610	10.0484	1.501 832
Ba(II)	6	2.6338	10.9359	1.492 203
P(I)	6	1.5544	3.9973	1.502 293
O(I)	6	1.5544	1.0000	−0.906 667
O(II)	18	1.5708	1.0000	−0.946 248

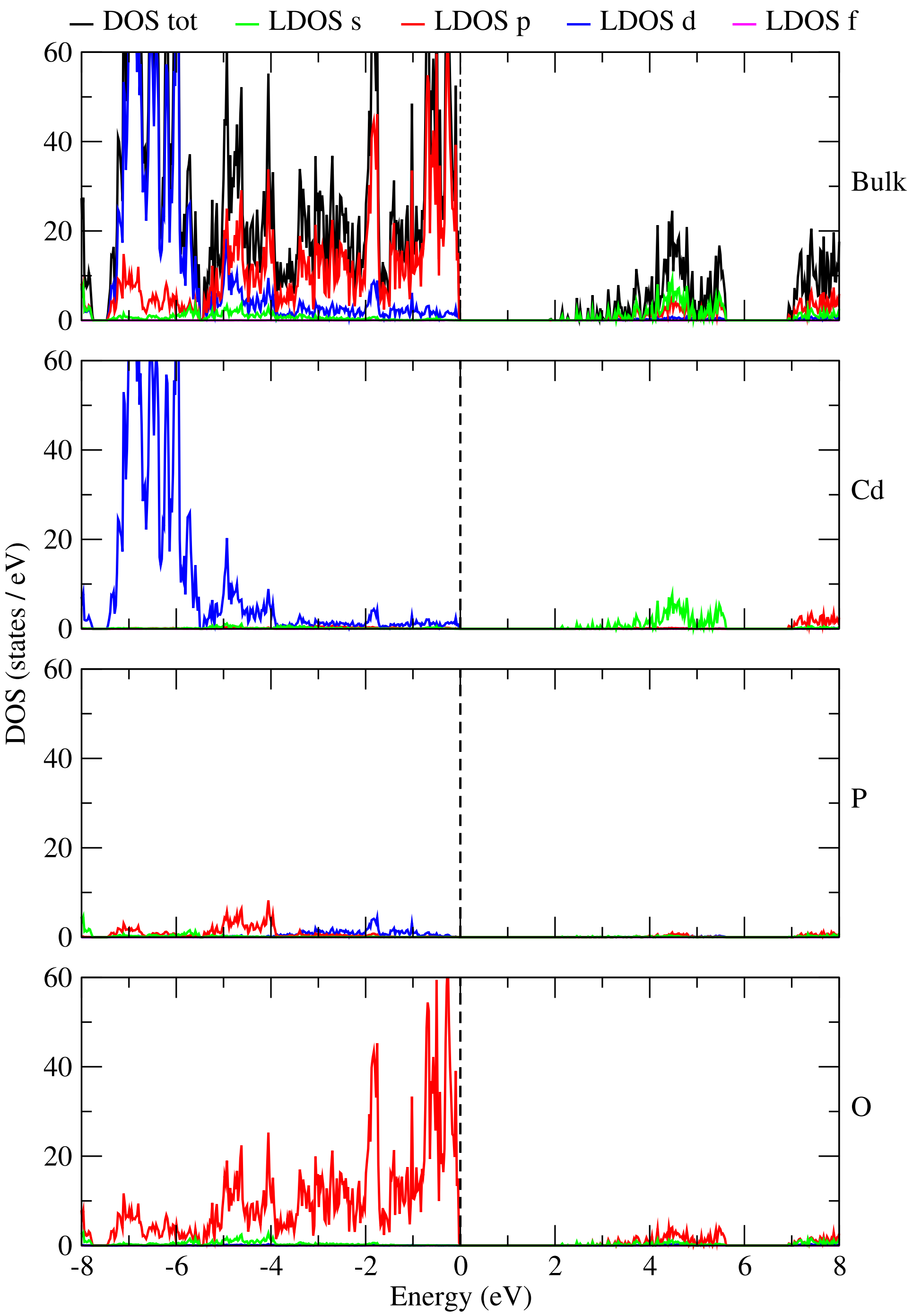

Figure S-45: Local density of states for the γ-$Cd_3(PO_4)_2$ bulk phase.

Table S-48: Calculated properties for the γ-$Cd_3(PO_4)_2$ bulk phase. Number of non-equivalent species, N; average distance for nearest neighbors, d_{NN}; effective coordination number, ECN; and net atomic charge, Q.

Non-equivalent species	N	d_{NN} (Å)	ECN (NNN)	Q (e^-)
Cd(I)	3	2.3781	6.3860	1.243 425
Cd(II)	6	2.1569	7.3140	1.228 354
P(I)	6	1.5255	3.9730	1.553 847
O(I)	6	1.5255	1.0033	−0.828 240
O(II)	18	1.5764	1.0001	−0.858 558

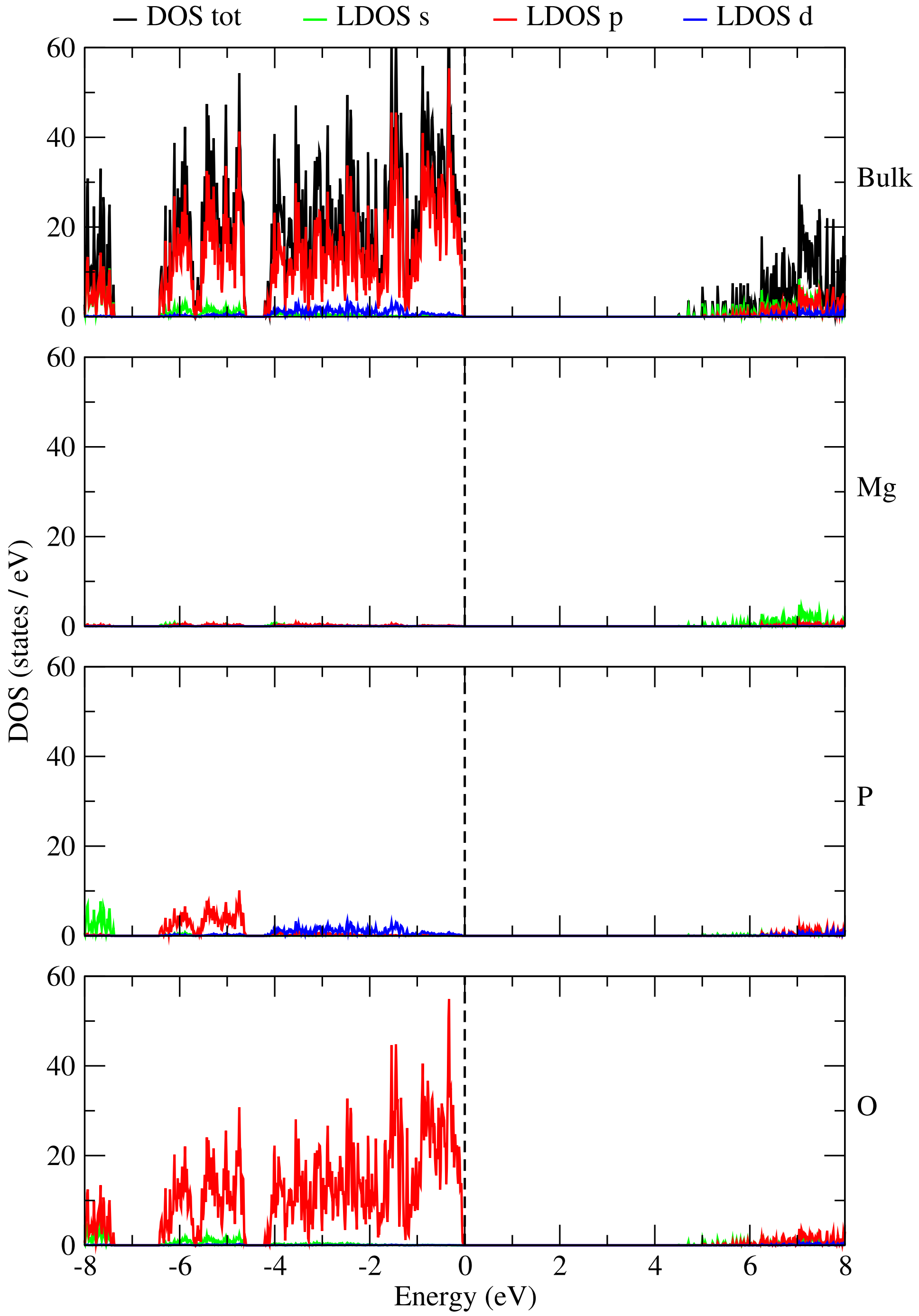

Figure S-46: Local density of states for the γ-$Mg_3(PO_4)_2$ bulk phase.

Table S-49: Calculated properties for the γ-$Mg_3(PO_4)_2$ bulk phase. Number of non-equivalent species, N; average distance for nearest neighbors, d_{NN}; effective coordination number, ECN; and net atomic charge, Q.

Non-equivalent species	N	d_{NN} (Å)	ECN (NNN)	Q (e^-)
Mg(I)	3	2.1820	6.0430	1.517 767
Mg(II)	6	1.9347	3.9265	1.553 054
P(I)	6	1.5156	3.9632	1.687 937
O(I)	6	1.5156	1.1195	−0.948 580
O(II)	18	1.5748	1.0268	−1.017 098

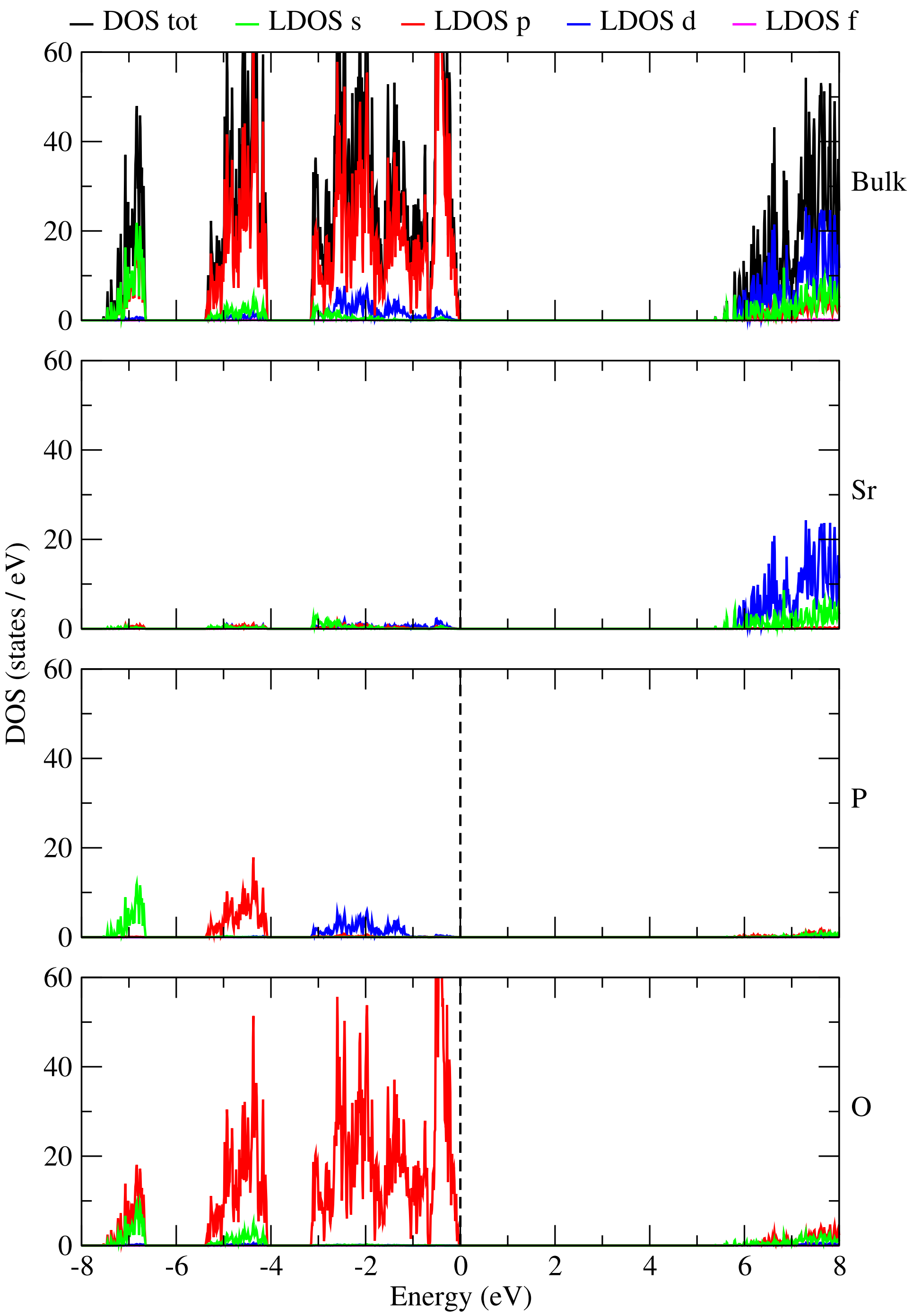

Figure S-47: Local density of states for the γ-$Sr_3(PO_4)_2$ bulk phase.

Table S-50: Calculated properties for the γ-$Sr_3(PO_4)_2$ bulk phase. Number of non-equivalent species, N; average distance for nearest neighbors, d_{NN}; effective coordination number, ECN; and net atomic charge, Q.

Non-equivalent species	N	d_{NN} (Å)	ECN (NNN)	Q (e^-)
Sr(I)	3	2.6006	8.8175	1.533 767
Sr(II)	6	2.4338	10.4510	1.510 496
P(I)	6	1.5475	3.9957	1.513 437
O(I)	6	1.5475	1.0000	−0.915 085
O(II)	18	1.5680	1.0000	−0.958 577

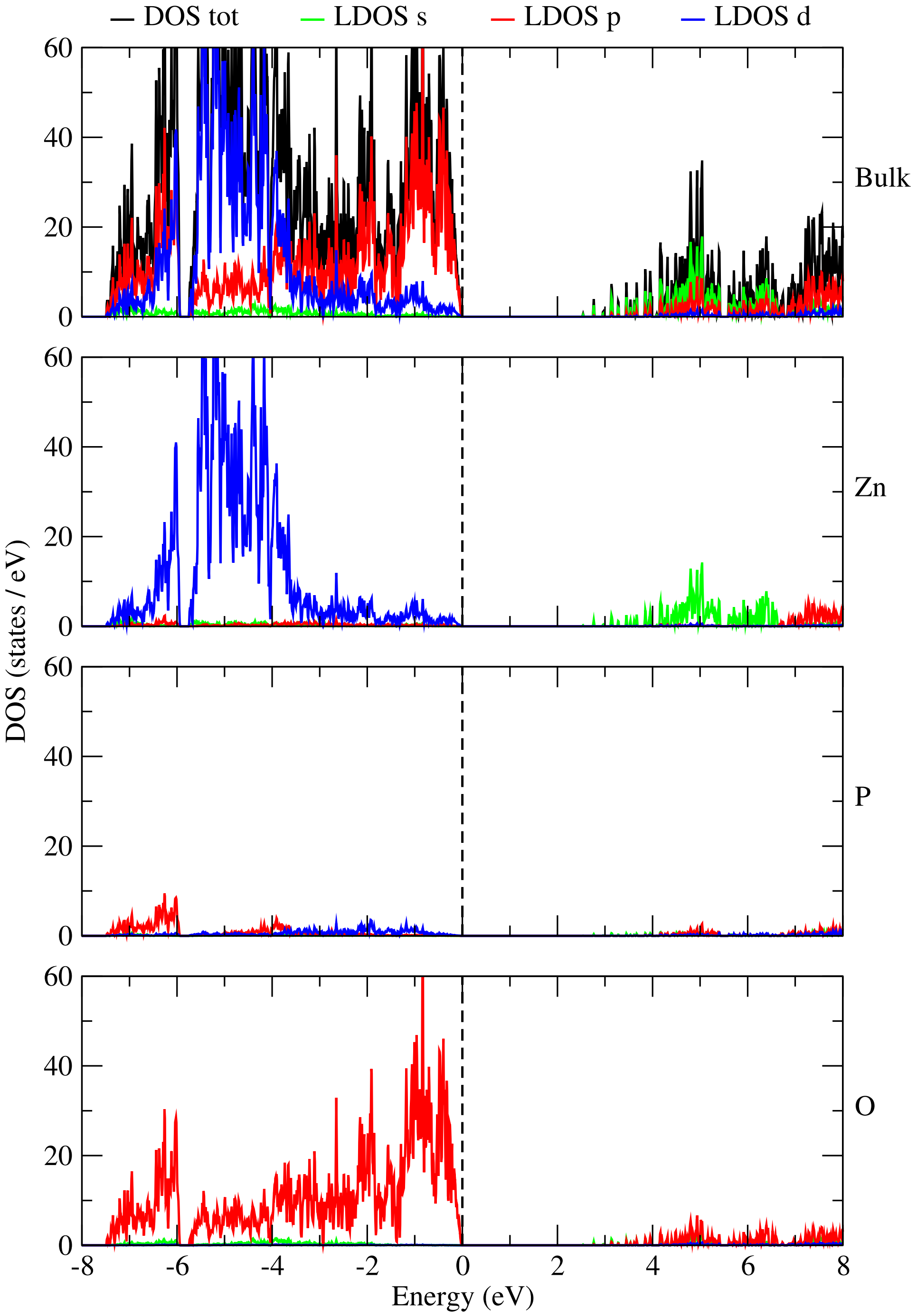

Figure S-48: Local density of states for the γ-$Zn_3(PO_4)_2$ bulk phase.

Table S-51: Calculated properties for the γ-$Zn_3(PO_4)_2$ bulk phase. Number of non-equivalent species, N; average distance for nearest neighbors, d_{NN}; effective coordination number, ECN; and net atomic charge, Q.

Non-equivalent species	N	d_{NN} (Å)	ECN (NNN)	Q (e^-)
Zn(I)	3	2.1570	6.0528	1.137 779
Zn(II)	6	1.8949	3.6937	1.171 939
P(I)	6	1.5182	3.9614	1.634 574
O(I)	6	1.5182	1.2065	−0.815 465
O(II)	18	1.5789	1.0386	−0.853 313

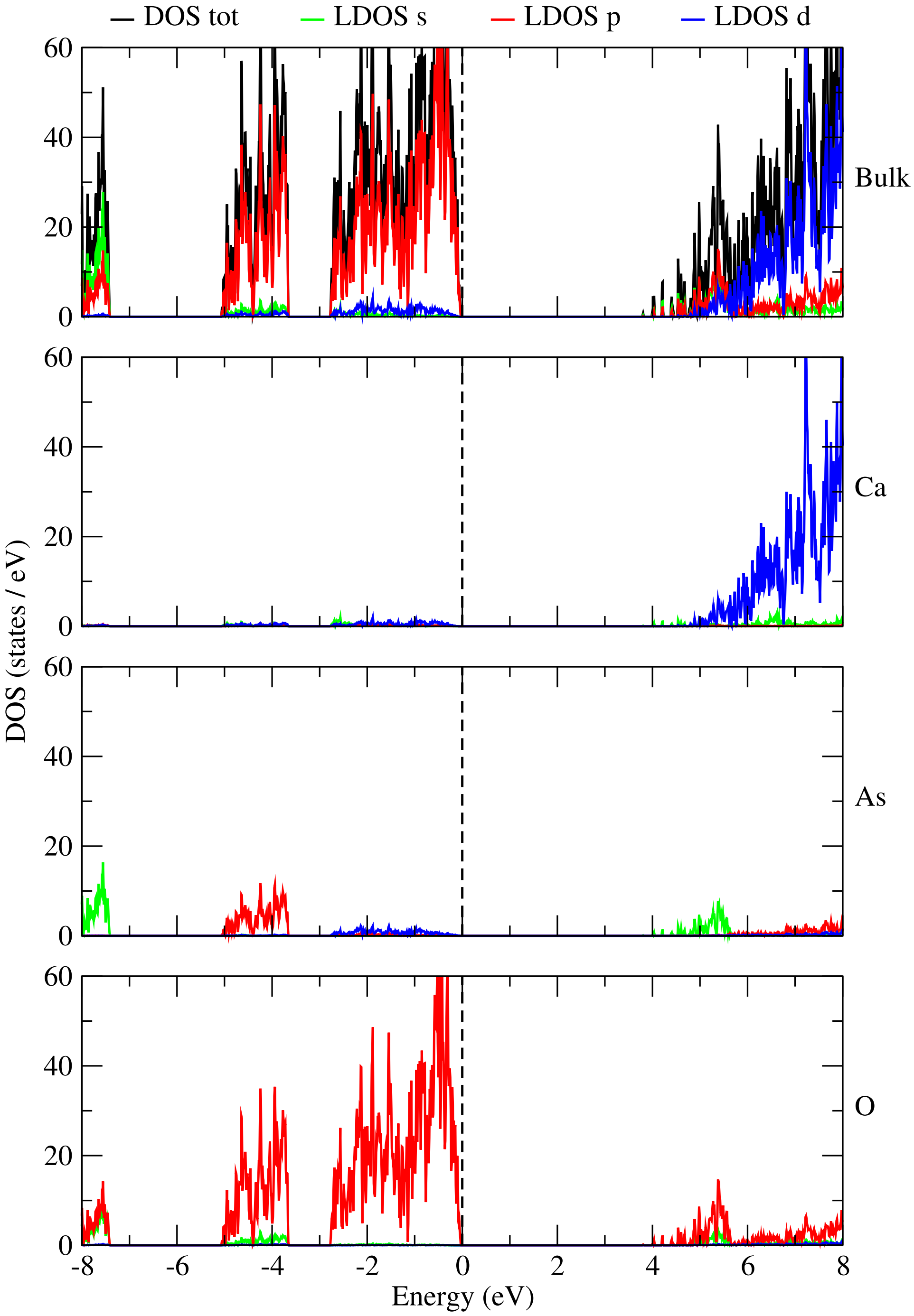

Figure S-49: Local density of states for the γ-$Ca_3(AsO_4)_2$ bulk phase.

Table S-52: Calculated properties for the γ-$Ca_3(AsO_4)_2$ bulk phase. Number of non-equivalent species, N; average distance for nearest neighbors, d_{NN}; effective coordination number, ECN; and net atomic charge, Q.

Non-equivalent species	N	d_{NN} (Å)	ECN (NNN)	Q (e^-)
Ca(I)	3	2.4674	6.3873	1.472 240
Ca(II)	6	2.2022	6.6720	1.480 418
As(I)	6	1.6932	3.9934	1.719 834
O(I)	6	1.6932	1.0710	−0.984 841
O(II)	18	1.7210	1.0042	−0.983 843

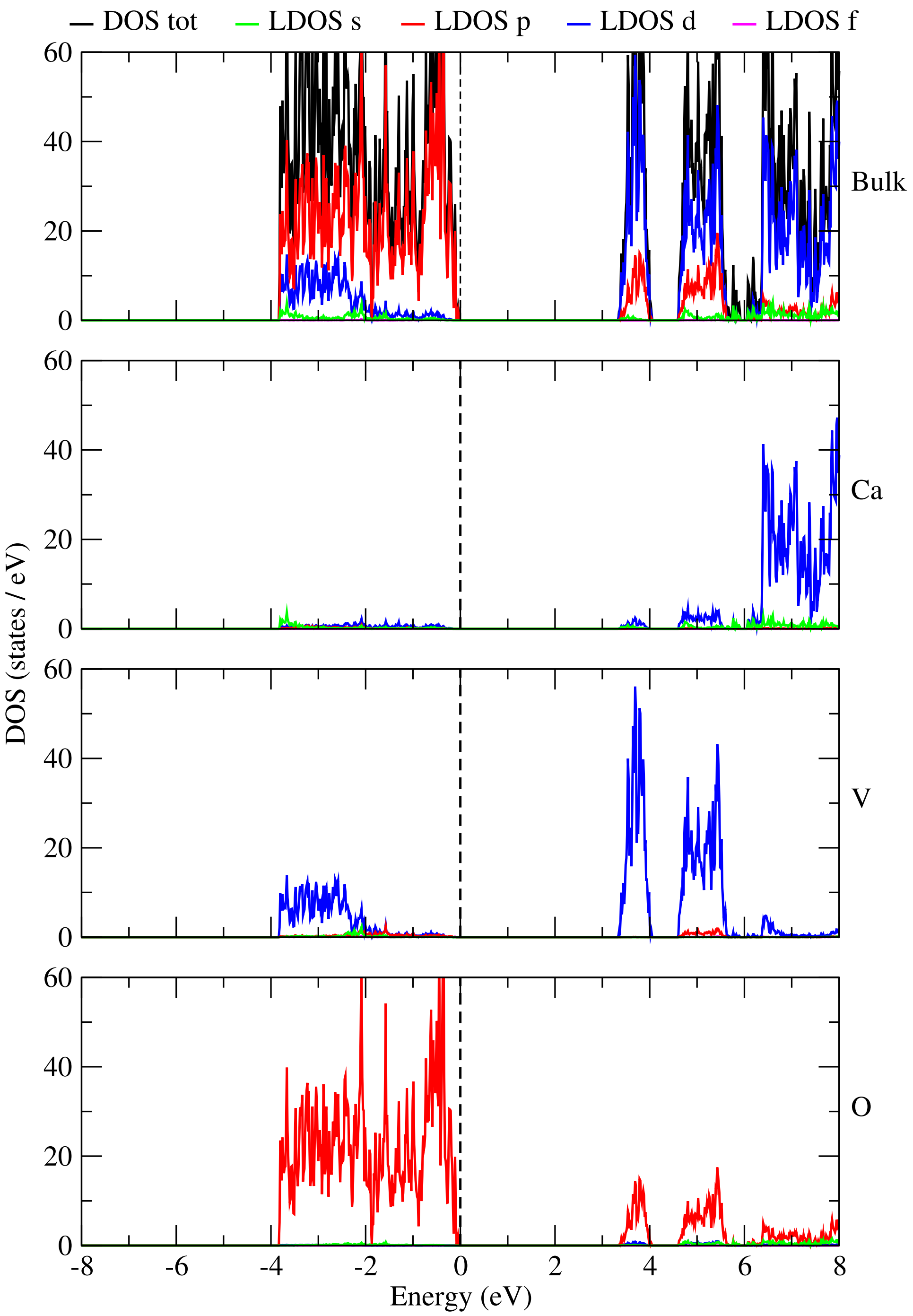

Figure S-50: Local density of states for the γ-$Ca_3(VO_4)_2$ bulk phase.

Table S-53: Calculated properties for the γ-$Ca_3(VO_4)_2$ bulk phase. Number of non-equivalent species, N; average distance for nearest neighbors, d_{NN}; effective coordination number, ECN; and net atomic charge, Q.

Non-equivalent species	N	d_{NN} (Å)	ECN (NNN)	Q (e^-)
Ca(I)	3	2.4689	6.3282	1.520 019
Ca(II)	6	2.2320	6.0409	1.512 255
V(I)	6	1.6895	3.9868	2.026 368
O(I)	6	1.6895	1.0440	−1.059 739
O(II)	18	1.7287	1.0094	−1.079 631

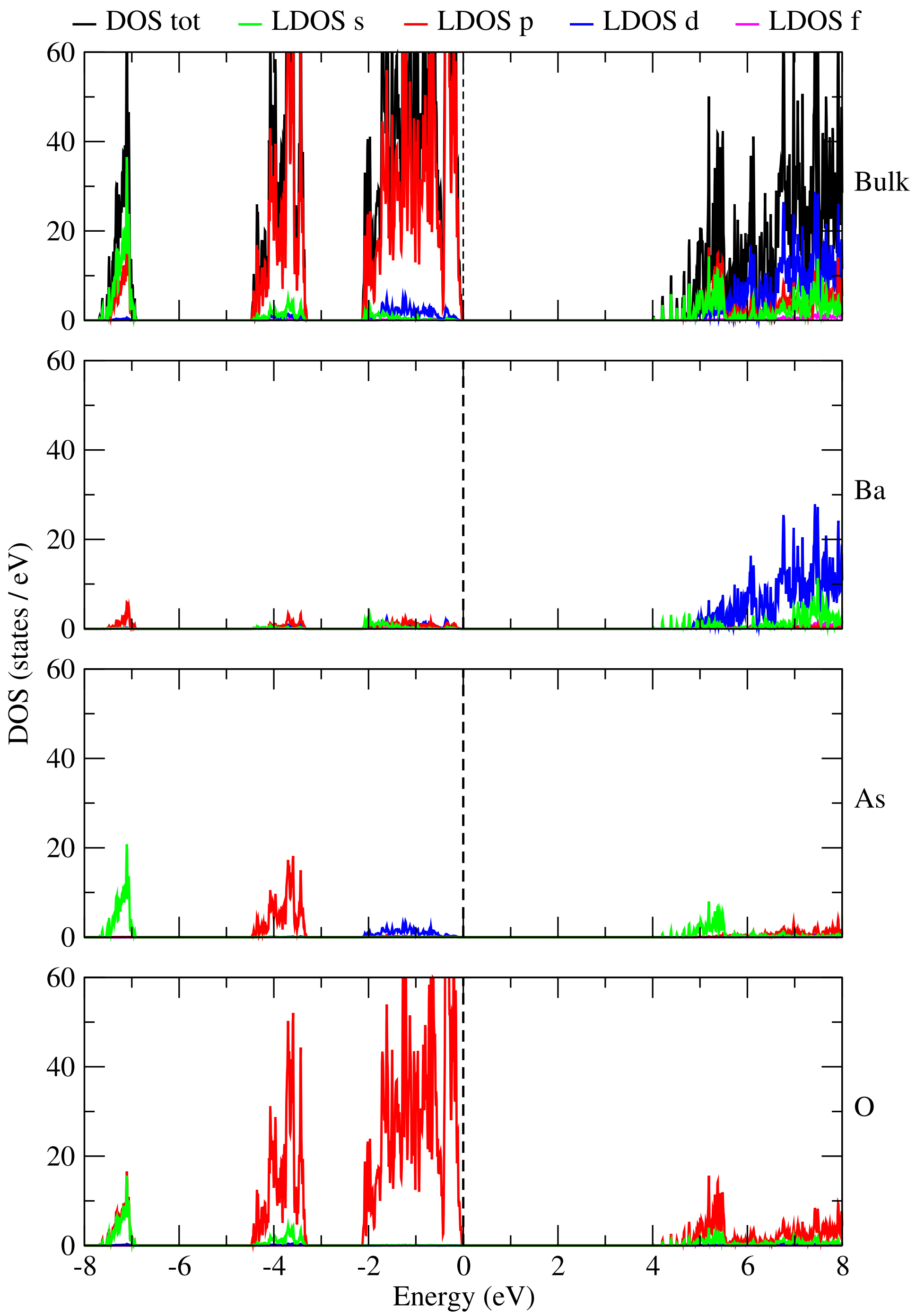

Figure S-51: Local density of states for the γ-$Ba_3(AsO_4)_2$ bulk phase.

Table S-54: Calculated properties for the γ-$Ba_3(AsO_4)_2$ bulk phase. Number of non-equivalent species, N; average distance for nearest neighbors, d_{NN}; effective coordination number, ECN; and net atomic charge, Q.

Non-equivalent species	N	d_{NN} (Å)	ECN (NNN)	Q (e^-)
Ba(I)	3	2.7778	8.5439	1.447 814
Ba(II)	6	2.5756	10.1744	1.468 712
As(I)	6	1.7124	3.9980	1.663 838
O(I)	6	1.7124	1.0001	−0.957 085
O(II)	18	1.7279	1.0000	−0.966 458

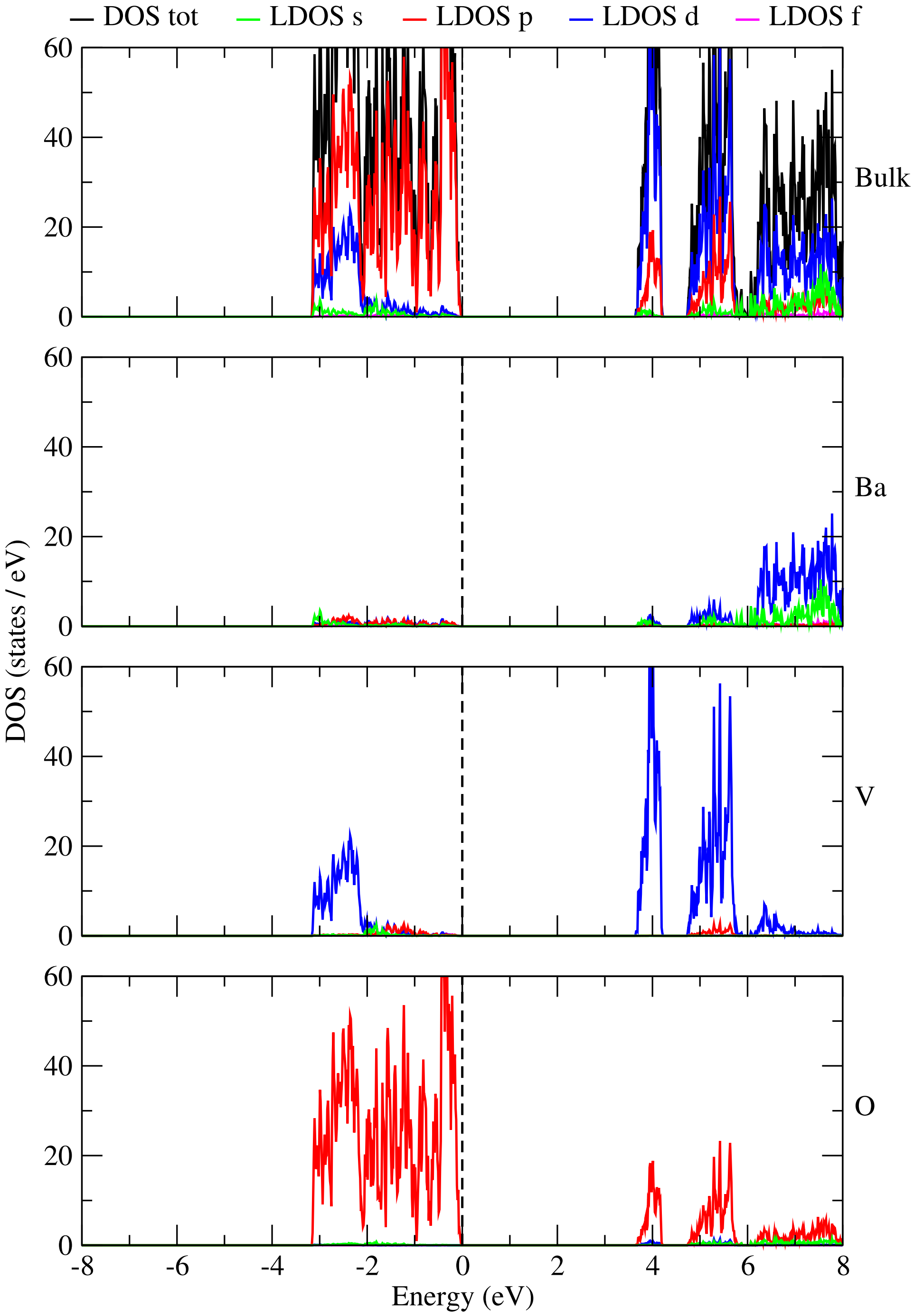

Figure S-52: Local density of states for the γ-$Ba_3(VO_4)_2$ bulk phase.

Table S-55: Calculated properties for the γ-$Ba_3(VO_4)_2$ bulk phase. Number of non-equivalent species, N; average distance for nearest neighbors, d_{NN}; effective coordination number, ECN; and net atomic charge, Q.

Non-equivalent species	N	d_{NN} (Å)	ECN (NNN)	Q (e^-)
Ba(I)	3	2.7721	8.5181	1.507 533
Ba(II)	6	2.6058	10.2218	1.500 831
V(I)	6	1.7074	3.9962	1.976 270
O(I)	6	1.7074	1.0000	−1.038 575
O(II)	18	1.7286	1.0000	−1.064 097

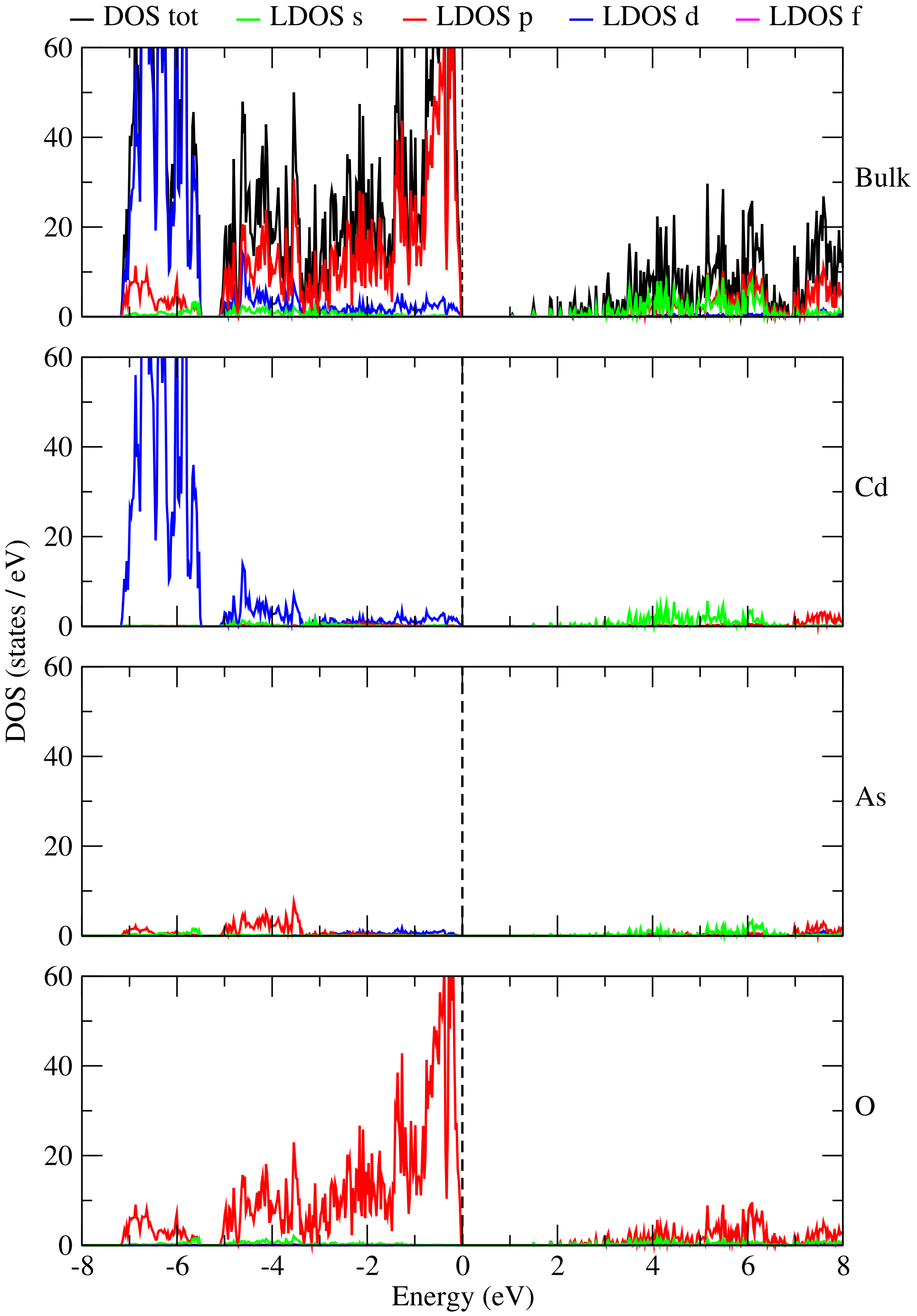

Figure S-53: Local density of states for the γ-$Cd_3(AsO_4)_2$ bulk phase.

Table S-56: Calculated properties for the γ-$Cd_3(AsO_4)_2$ bulk phase. Number of non-equivalent species, N; average distance for nearest neighbors, d_{NN}; effective coordination number, ECN; and net atomic charge, Q.

Non-equivalent species	N	d_{NN} (Å)	ECN (NNN)	Q (e^-)
Cd(I)	3	2.3836	6.0694	1.168 715
Cd(II)	6	2.1235	4.4112	1.204 567
As(I)	6	1.6801	3.9771	1.735 472
O(I)	6	1.6801	1.1539	−0.894 682
O(II)	18	1.7318	1.0275	−0.876 571

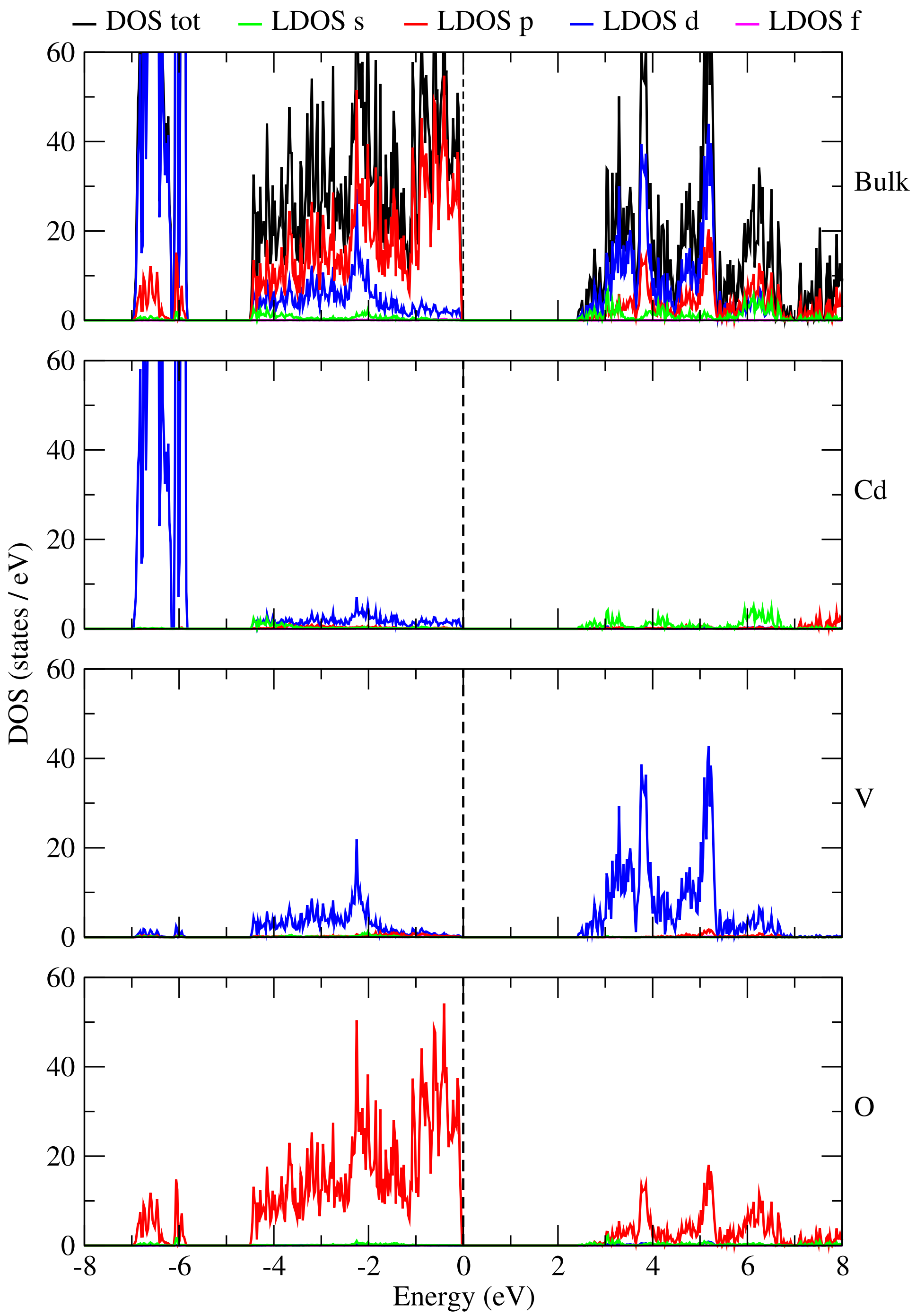

Figure S-54: Local density of states for the γ-$Cd_3(VO_4)_2$ bulk phase.

Table S-57: Calculated properties for the γ-$Cd_3(VO_4)_2$ bulk phase. Number of non-equivalent species, N; average distance for nearest neighbors, d_{NN}; effective coordination number, ECN; and net atomic charge, Q.

Non-equivalent species	N	d_{NN} (Å)	ECN (NNN)	Q (e^-)
Cd(I)	3	2.3664	6.0331	1.207 021
Cd(II)	6	2.1095	3.9379	1.243 547
V(I)	6	1.6764	3.9620	2.038 227
O(I)	6	1.6764	1.1713	−0.939 169
O(II)	18	1.7430	1.0673	−0.982 039

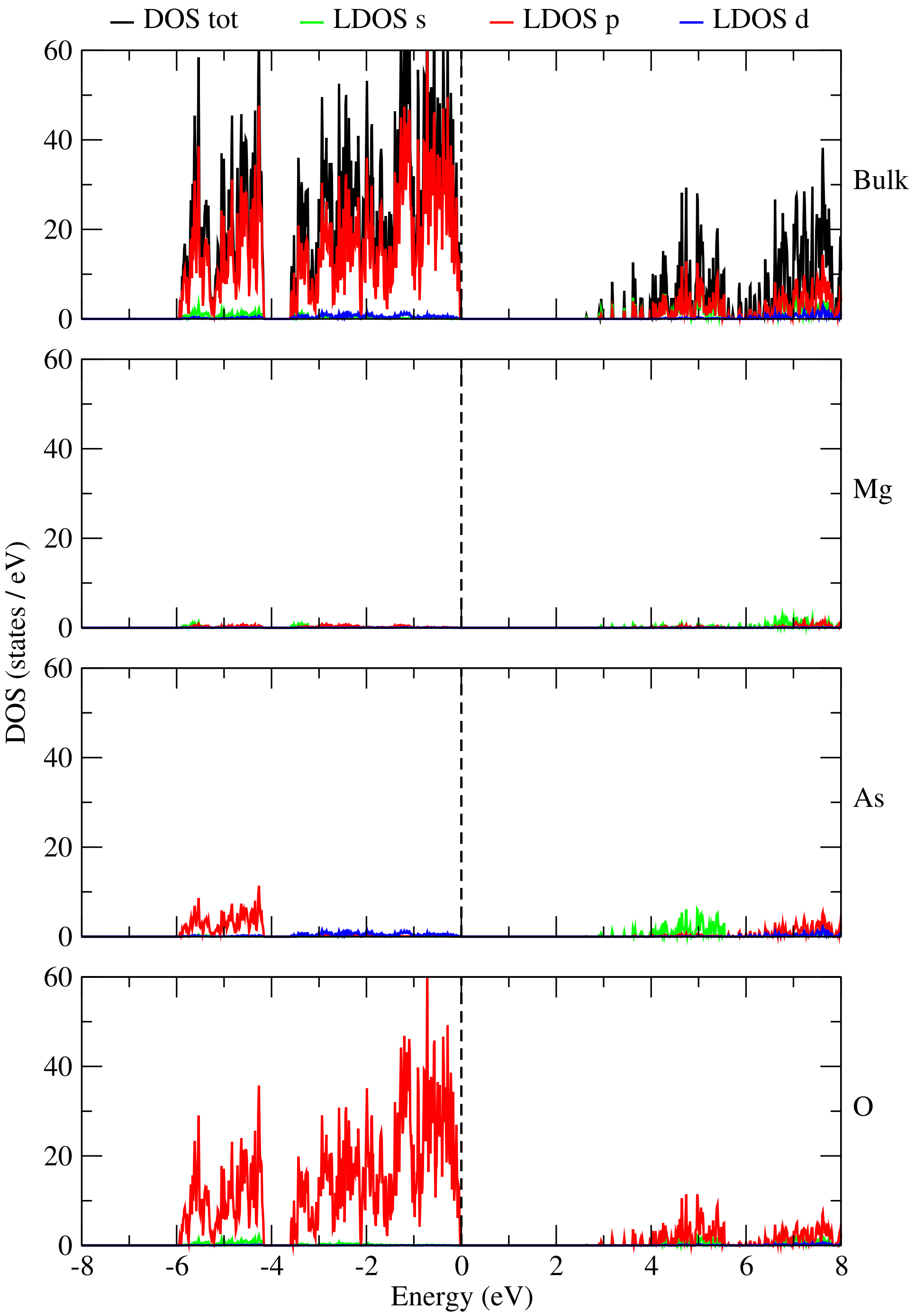

Figure S-55: Local density of states for the γ-$Mg_3(AsO_4)_2$ bulk phase.

Table S-58: Calculated properties for the γ-$Mg_3(AsO_4)_2$ bulk phase. Number of non-equivalent species, N; average distance for nearest neighbors, d_{NN}; effective coordination number, ECN; and net atomic charge, Q.

Non-equivalent species	N	d_{NN} (Å)	ECN (NNN)	Q (e^-)
Mg(I)	3	2.1894	6.0372	1.485 408
Mg(II)	6	1.9155	3.8156	1.562 499
As(I)	6	1.6675	3.9663	1.867 988
O(I)	6	1.6675	1.6664	−1.022 724
O(II)	18	1.7298	1.7505	−1.050 156

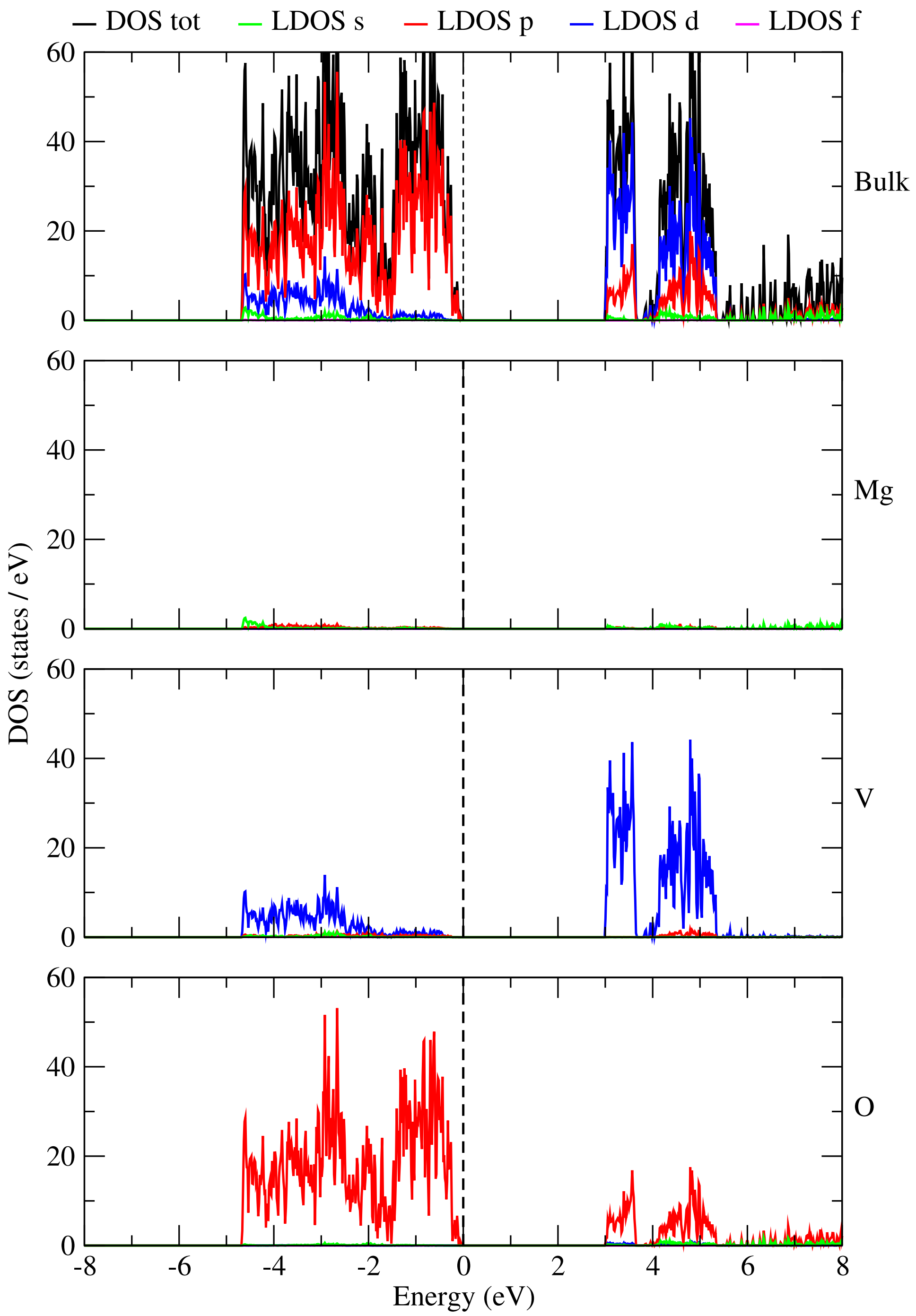

Figure S-56: Local density of states for the γ-$Mg_3(VO_4)_2$ bulk phase.

Table S-59: Calculated properties for the γ-$Mg_3(VO_4)_2$ bulk phase. Number of non-equivalent species, N; average distance for nearest neighbors, d_{NN}; effective coordination number, ECN; and net atomic charge, Q.

Non-equivalent species	N	d_{NN} (Å)	ECN (NNN)	Q (e^-)
Mg(I)	3	2.1755	6.1262	1.508 386
Mg(II)	6	1.9473	3.9318	1.558 765
V(I)	6	1.6612	3.9355	2.128 900
O(I)	6	1.6612	1.5591	−1.020 975
O(II)	18	1.7475	2.0093	−1.140 294

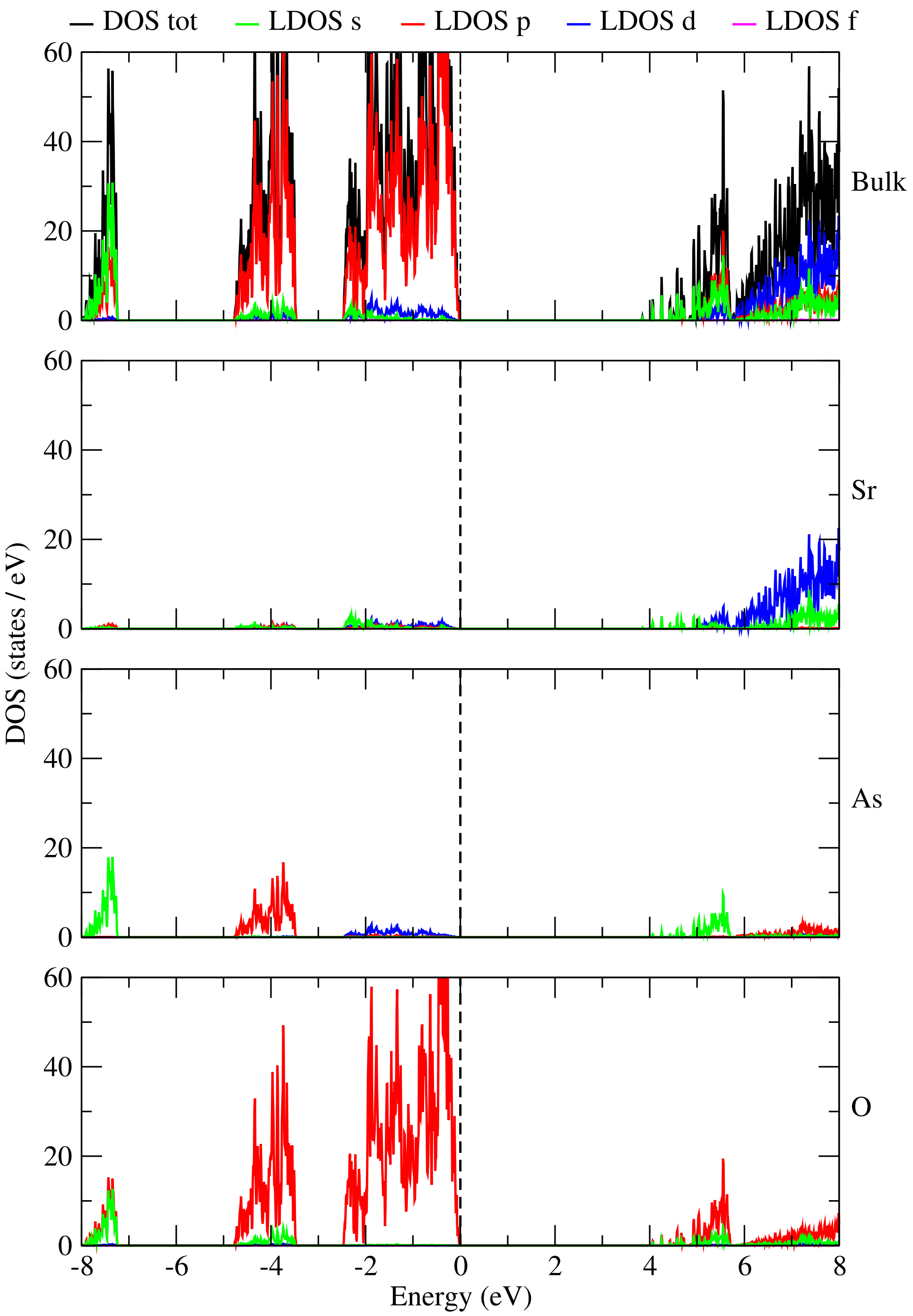

Figure S-57: Local density of states for the γ-$Sr_3(AsO_4)_2$ bulk phase.

Table S-60: Calculated properties for the γ-$Sr_3(AsO_4)_2$ bulk phase. Number of non-equivalent species, N; average distance for nearest neighbors, d_{NN}; effective coordination number, ECN; and net atomic charge, Q.

Non-equivalent species	N	d_{NN} (Å)	ECN (NNN)	Q (e^-)
Sr(I)	3	2.6211	7.3514	1.491 699
Sr(II)	6	2.3917	9.0660	1.496 690
As(I)	6	1.7026	3.9963	1.683 538
O(I)	6	1.7026	1.0044	−0.978 608
O(II)	18	1.7235	1.0001	−0.982 490

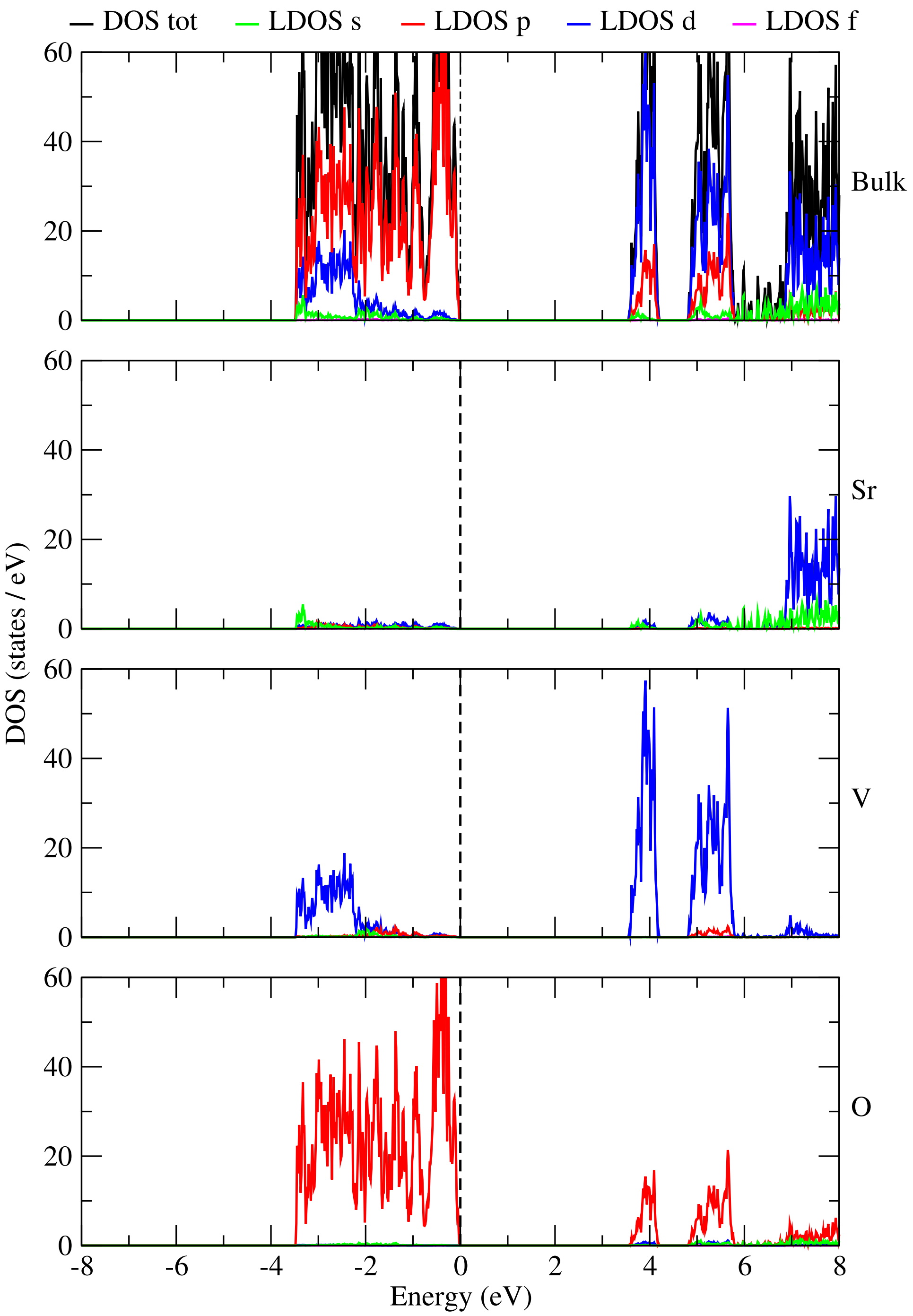

Figure S-58: Local density of states for the γ-$Sr_3(VO_4)_2$ bulk phase.

Table S-61: Calculated properties for the γ-$Sr_3(VO_4)_2$ bulk phase. Number of non-equivalent species, N; average distance for nearest neighbors, d_{NN}; effective coordination number, ECN; and net atomic charge, Q.

Non-equivalent species	N	d_{NN} (Å)	ECN (NNN)	Q (e^-)
Sr(I)	3	2.6239	7.3493	1.546 663
Sr(II)	6	2.4172	8.9213	1.530 824
V(I)	6	1.7005	3.9943	1.995 421
O(I)	6	1.7005	1.0025	−1.063 673
O(II)	18	1.7263	1.0001	−1.078 634

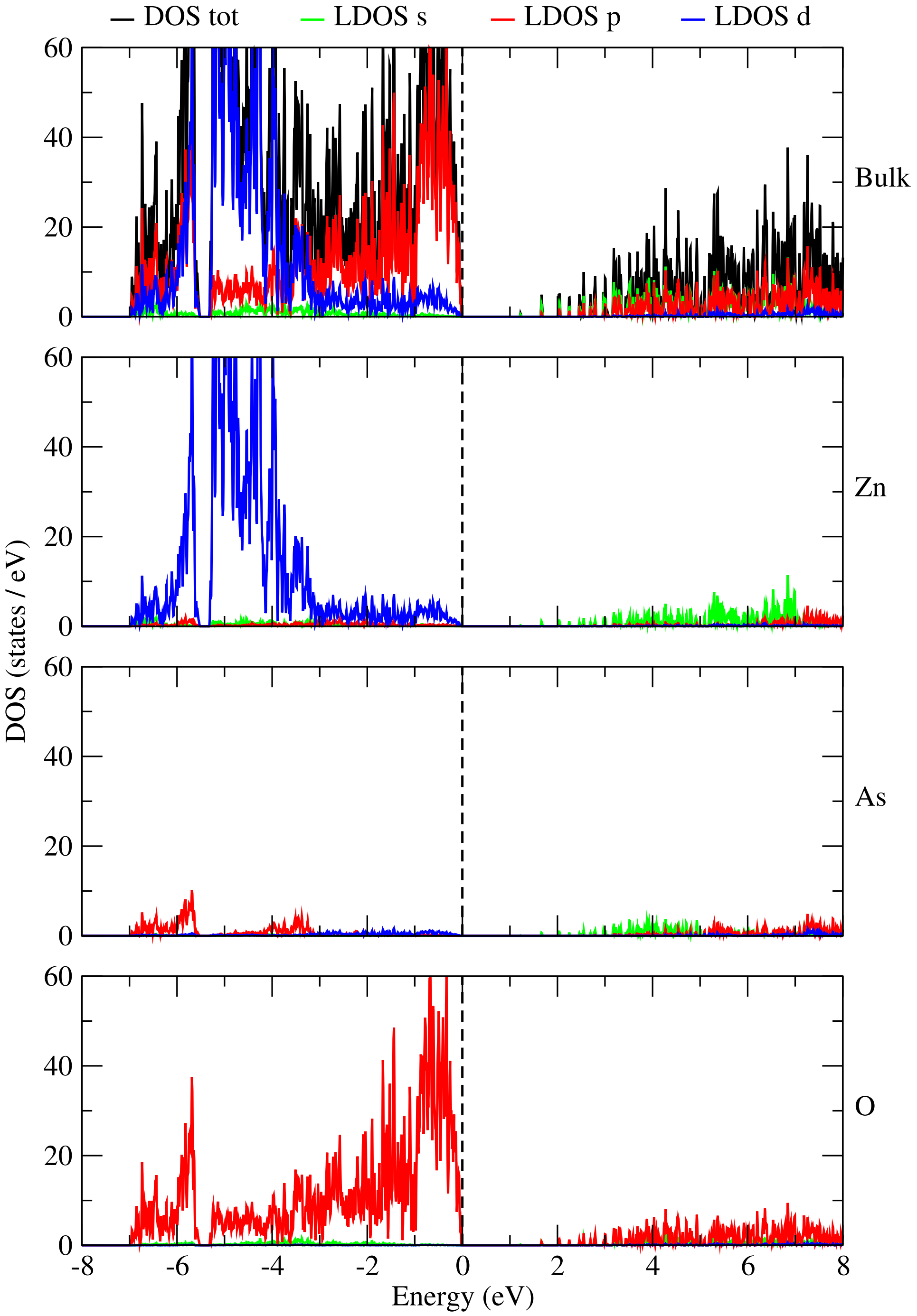

Figure S-59: Local density of states for the γ-$Zn_3(AsO_4)_2$ bulk phase.

Table S-62: Calculated properties for the γ-$Zn_3(AsO_4)_2$ bulk phase. Number of non-equivalent species, N; average distance for nearest neighbors, d_{NN}; effective coordination number, ECN; and net atomic charge, Q.

Non-equivalent species	N	d_{NN} (Å)	ECN (NNN)	Q (e^-)
Zn(I)	3	2.1609	6.0555	1.089 133
Zn(II)	6	1.8743	3.7093	1.177 736
As(I)	6	1.6724	3.9652	1.802 765
O(I)	6	1.6724	1.7775	−0.889 617
O(II)	18	1.7359	1.8960	−0.878 483

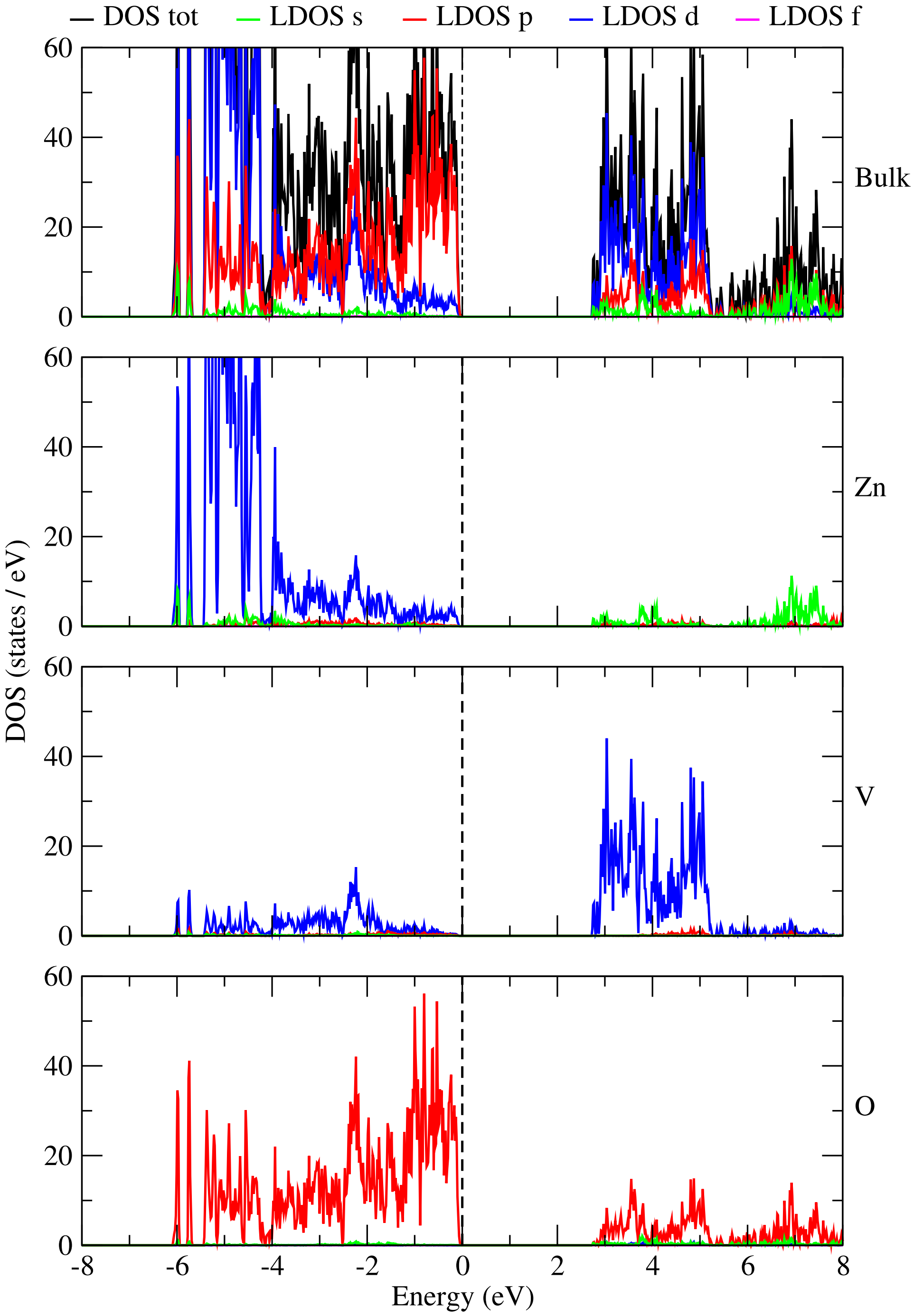

Figure S-60: Local density of states for the γ-$Zn_3(VO_4)_2$ bulk phase.

Table S-63: Calculated properties for the γ-$Zn_3(VO_4)_2$ bulk phase. Number of non-equivalent species, N; average distance for nearest neighbors, d_{NN}; effective coordination number, ECN; and net atomic charge, Q.

Non-equivalent species	N	d_{NN} (Å)	ECN (NNN)	Q (e^-)
Zn(I)	3	2.1562	6.2450	1.097 210
Zn(II)	6	1.8870	3.8275	1.199 053
V(I)	6	1.6731	3.9503	2.059 920
O(I)	6	1.6731	1.7510	−0.905 490
O(II)	18	1.7491	2.0788	−0.967 362

S-6.4 $Ca_{10}(PO_4)_6(OH)_2$-based Materials

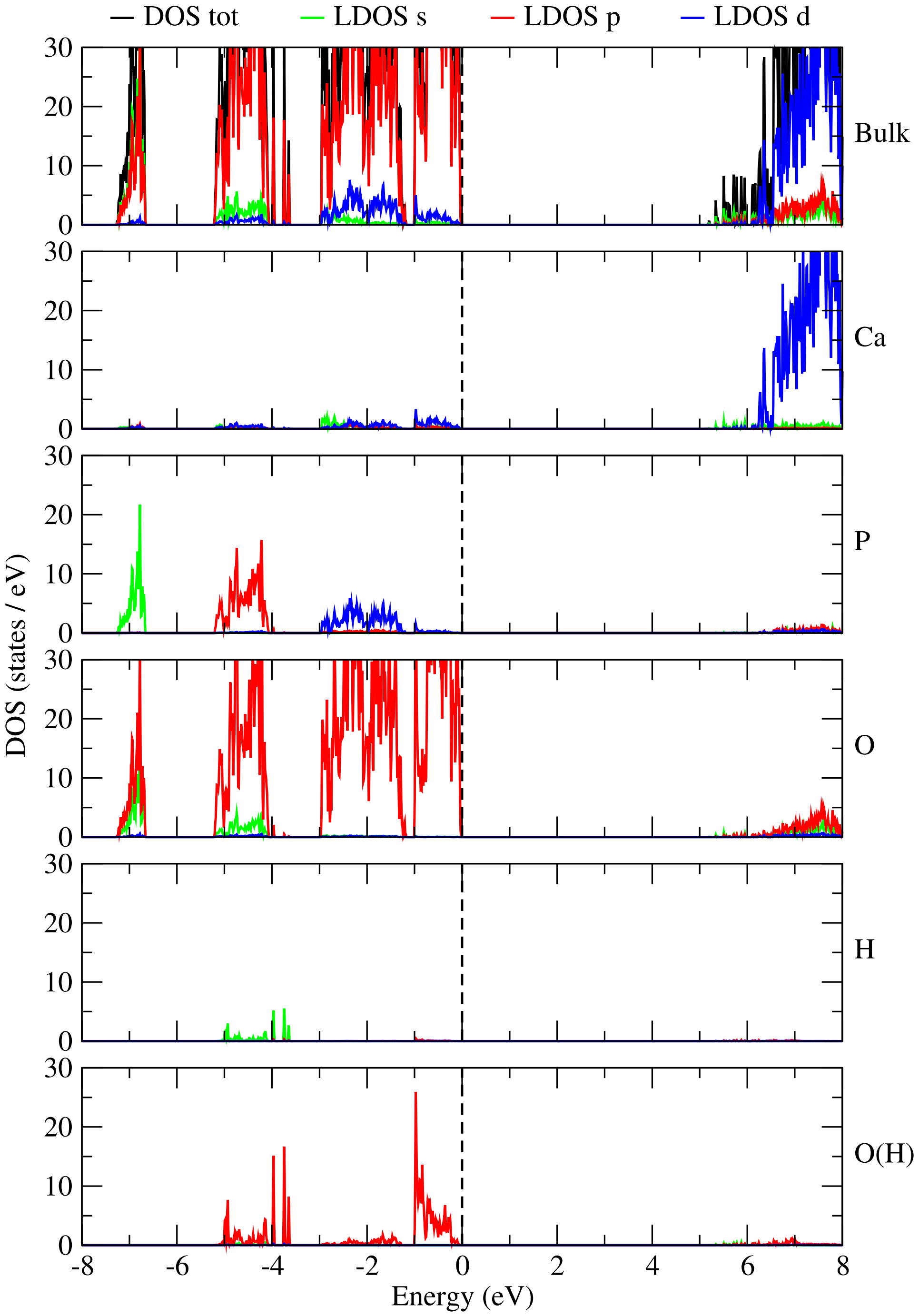

Figure S-61: Local density of states for the $Ca_{10}(PO_4)_6(OH)_2$ bulk phase.

Table S-64: Calculated properties for the $Ca_{10}(PO_4)_6(OH)_2$ bulk phase. Number of non-equivalent species, N; average distance for nearest neighbors, d_{NN}; effective coordination number, ECN; and net atomic charge, Q.

Non-equivalent species	N	d_{NN} (Å)	ECN (NNN)	Q (e^-)
Ca(I)	2	2.4297	8.1014	1.443 859
Ca(II)	2	2.4027	7.3003	1.446 659
Ca(III)	6	2.3488	7.2074	1.469 612
P(I)	6	1.5541	3.9985	1.517 284
O(I)	6	1.5541	1.0000	−0.907 555
O(II)	6	1.5682	1.0001	−0.952 993
O(III)	6	1.5588	1.0001	−0.925 786
O(IV)	6	1.5571	1.0001	−0.930 393
O(V)	2	0.9753	1.0000	−1.065 262
H(I)	2	0.9753	1.0000	0.364 236

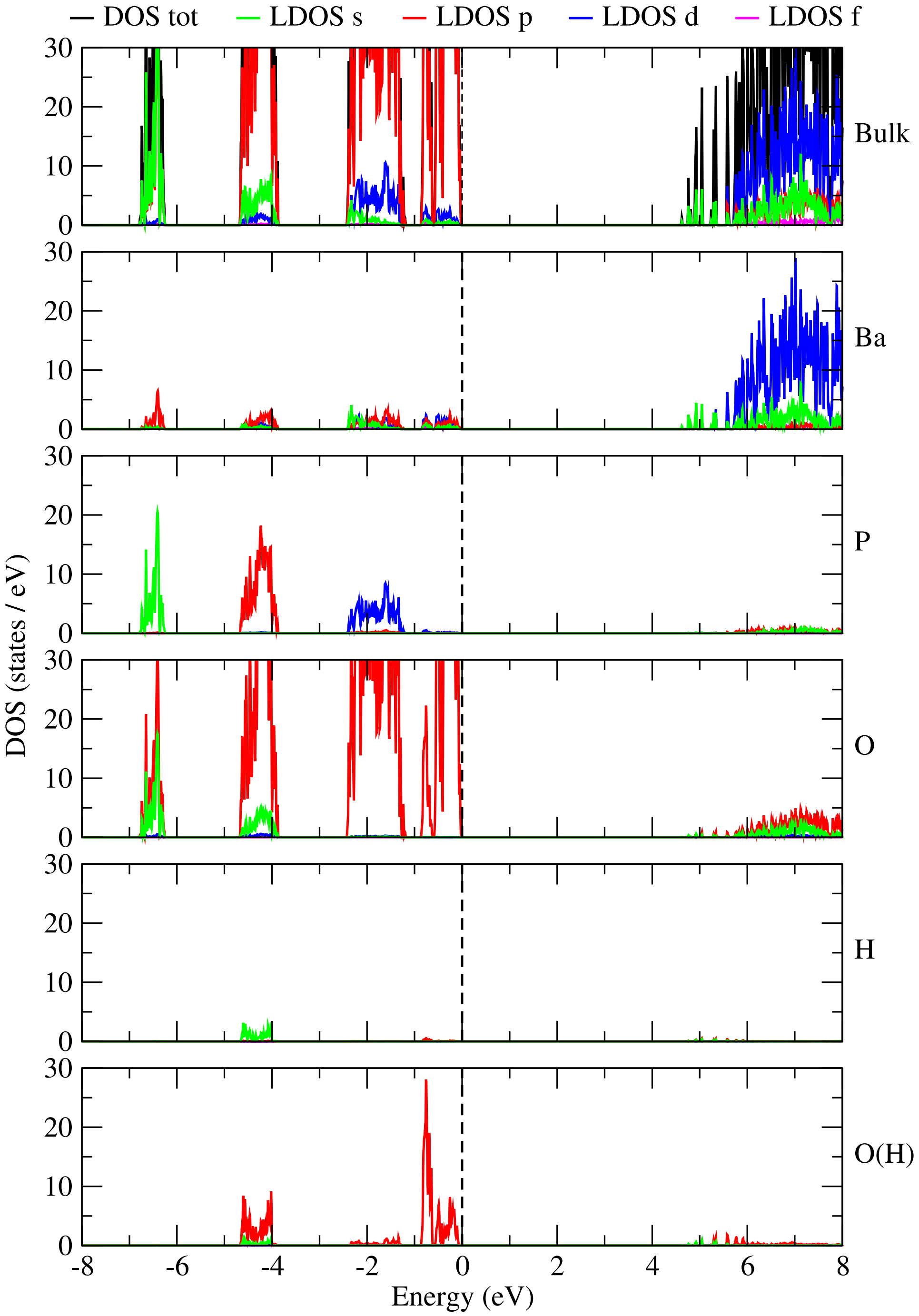

Figure S-62: Local density of states for the $Ba_{10}(PO_4)_6(OH)_2$ bulk phase.

Table S-65: Calculated properties for the $Ba_{10}(PO_4)_6(OH)_2$ bulk phase. Number of non-equivalent species, N; average distance for nearest neighbors, d_{NN}; effective coordination number, ECN; and net atomic charge, Q.

Non-equivalent species	N	d_{NN} (Å)	ECN (NNN)	Q (e^{-})
Ba(I)	2	2.7483	9.1305	1.480 193
Ba(II)	2	2.7129	8.2629	1.482 320
Ba(III)	6	2.6631	8.1631	1.502 756
P(I)	6	1.5637	3.9998	1.505 525
O(I)	6	1.5662	1.0000	−0.946 047
O(II)	6	1.5694	1.0000	−0.943 828
O(III)	6	1.5665	1.0000	−0.931 686
O(IV)	6	1.5637	1.0000	−0.937 161
O(V)	2	0.9725	1.0000	−1.066 972
H(I)	2	0.9725	1.0000	0.355 779

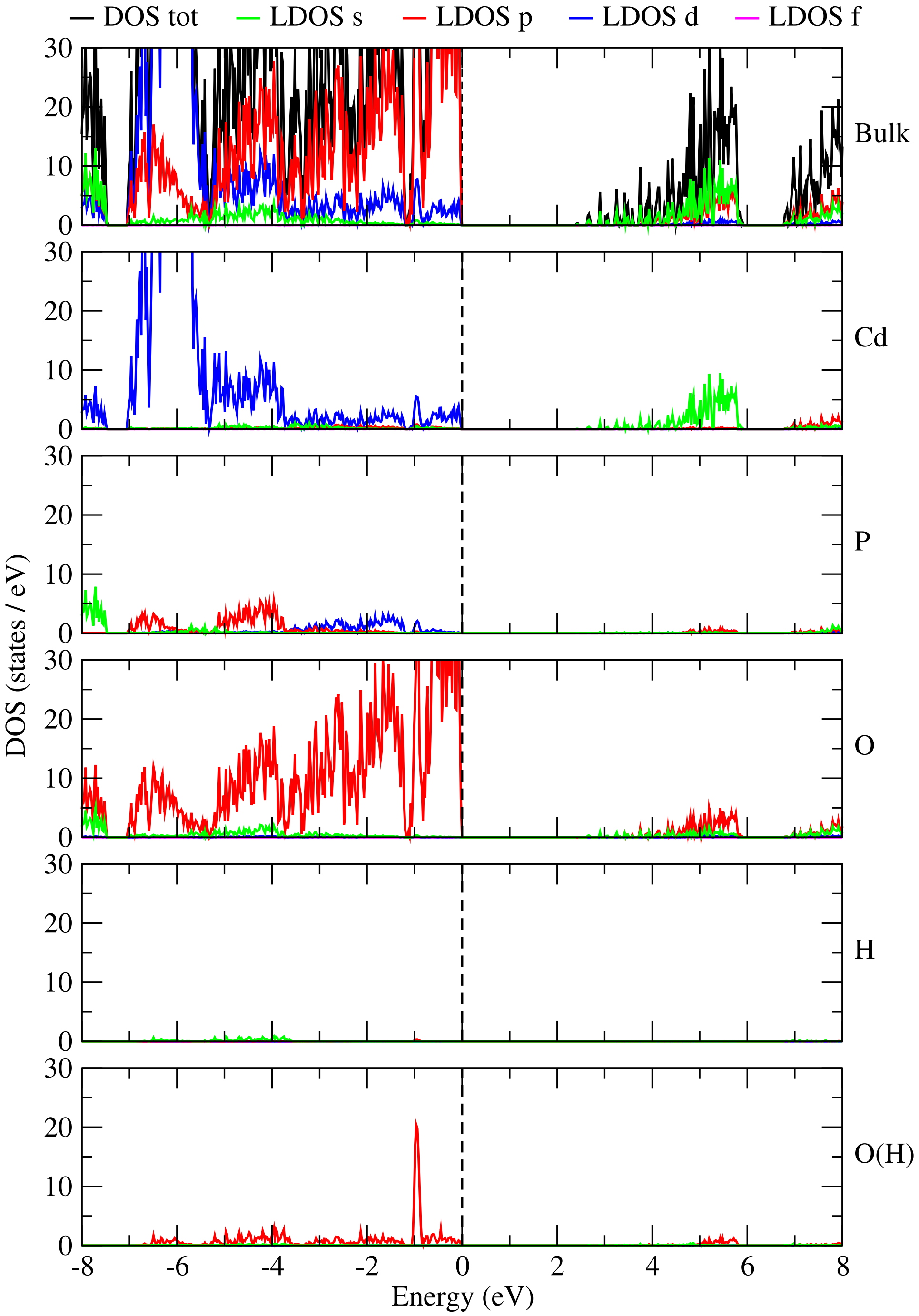

Figure S-63: Local density of states for the $Cd_{10}(PO_4)_6(OH)_2$ bulk phase.

Table S-66: Calculated properties for the $Cd_{10}(PO_4)_6(OH)_2$ bulk phase. Number of non-equivalent species, N; average distance for nearest neighbors, d_{NN}; effective coordination number, ECN; and net atomic charge, Q.

Non-equivalent species	N	d_{NN} (Å)	ECN (NNN)	Q (e^-)
Cd(I)	2	2.3463	6.8265	1.180 928
Cd(II)	2	2.3355	6.5063	1.179 502
Cd(III)	6	2.2384	6.5834	1.209 824
P(I)	6	1.5505	3.9925	1.502 805
O(I)	6	1.5505	1.0002	−0.803 857
O(II)	6	1.5829	1.0001	−0.838 689
O(III)	6	1.5615	1.0011	−0.840 435
O(IV)	6	1.5601	1.0016	−0.840 485
O(V)	2	0.9766	1.0000	−0.890 648
H(I)	2	0.9766	1.0000	0.362 728

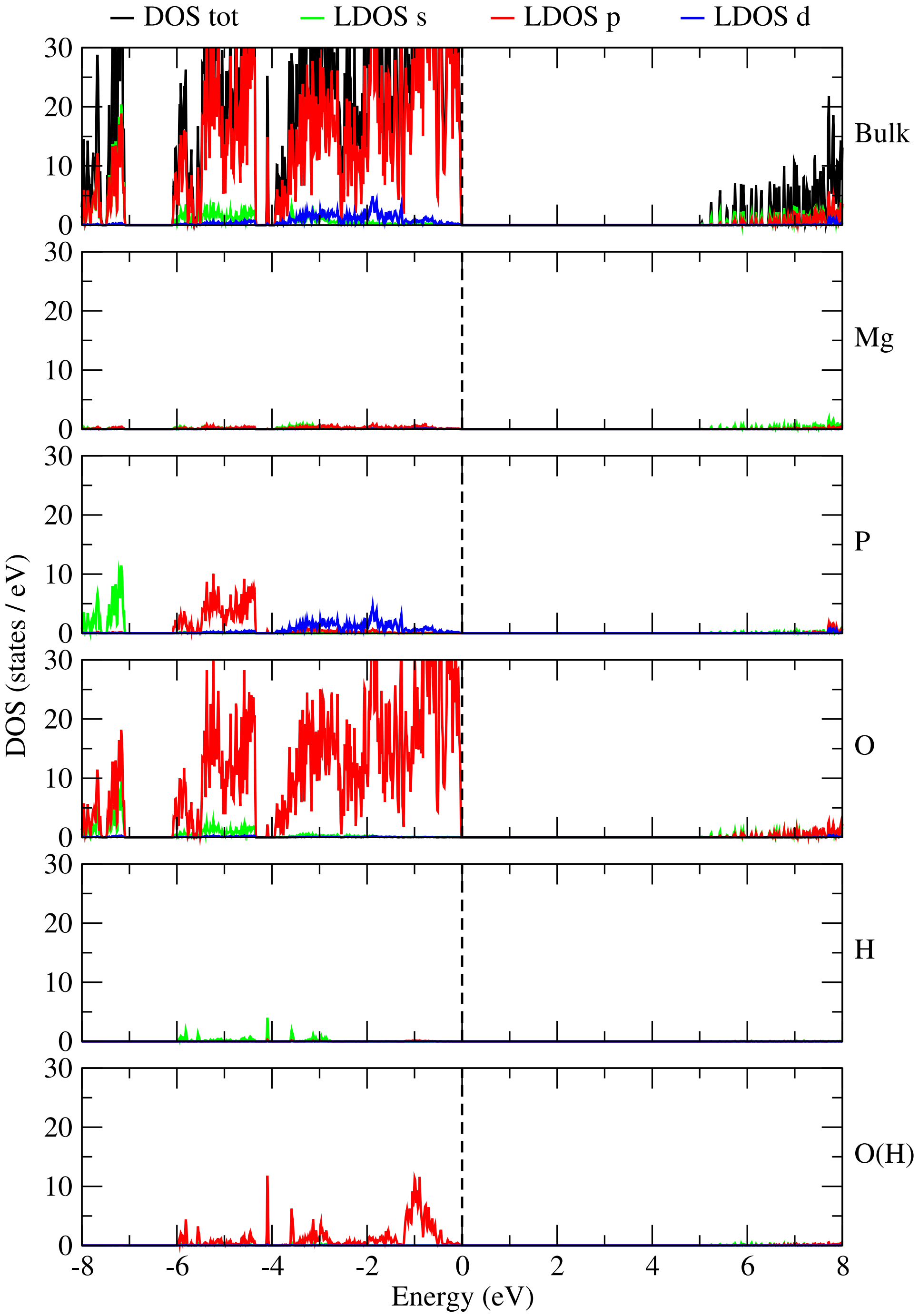

Figure S-64: Local density of states for the $Mg_{10}(PO_4)_6(OH)_2$ bulk phase.

Table S-67: Calculated properties for the $Mg_{10}(PO_4)_6(OH)_2$ bulk phase. Number of non-equivalent species, N; average distance for nearest neighbors, d_{NN}; effective coordination number, ECN; and net atomic charge, Q.

Non-equivalent species	N	d_{NN} (Å)	ECN (NNN)	Q (e^-)
Mg(I)	2	2.1086	6.0408	1.498 712
Mg(II)	2	2.1078	6.0340	1.498 551
Mg(III)	6	2.0376	6.0611	1.490 752
P(I)	6	1.5309	3.9837	1.597 091
O(I)	6	1.5309	1.0210	−0.922 543
O(II)	6	1.5782	1.0179	−0.993 193
O(III)	6	1.5631	1.0749	−0.967 569
O(IV)	6	1.5622	1.0763	−0.966 426
O(V)	2	0.9802	1.0000	−1.074 429
H(I)	2	0.9802	1.0000	0.362 828

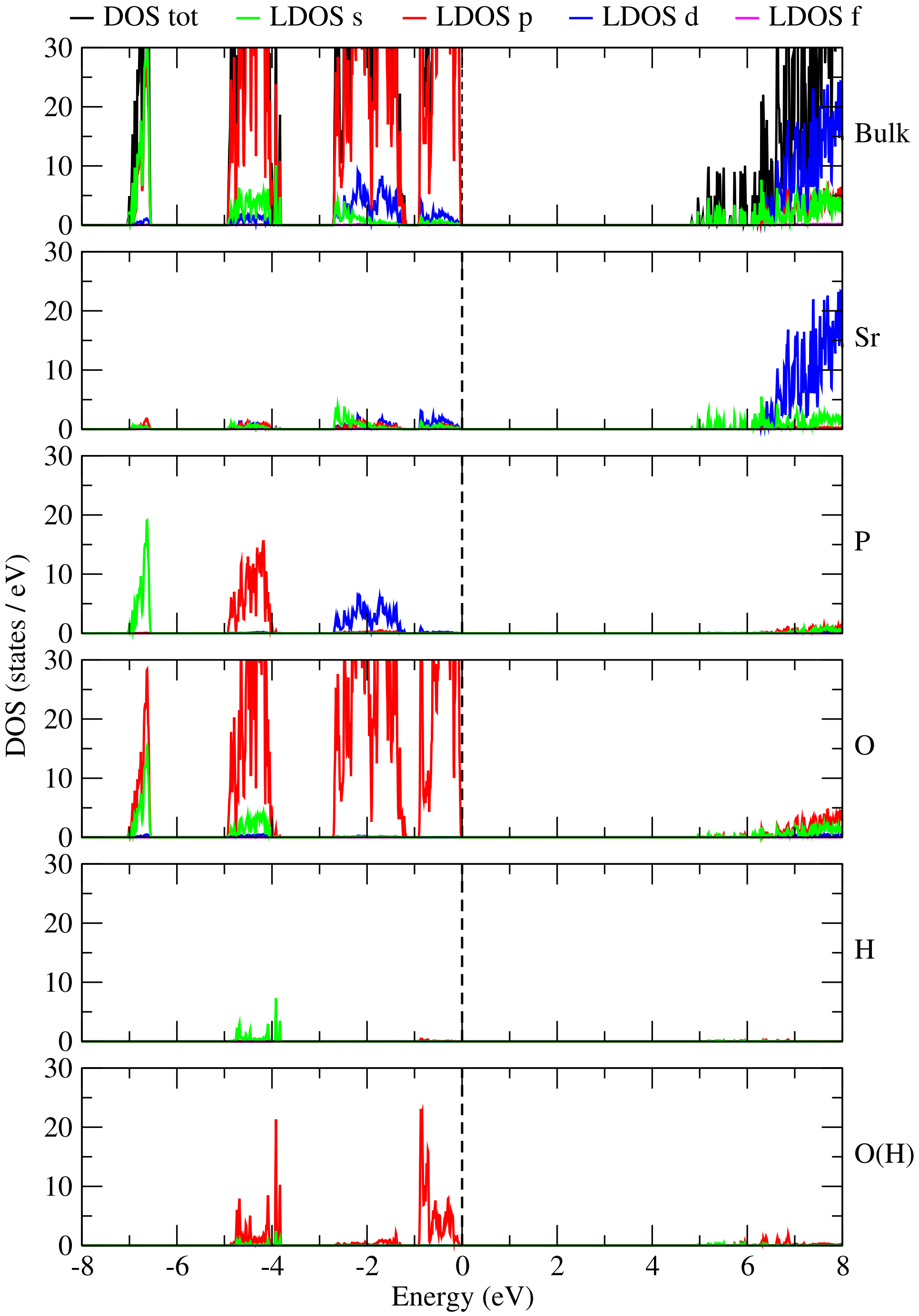

Figure S-65: Local density of states for the $Sr_{10}(PO_4)_6(OH)_2$ bulk phase.

Table S-68: Calculated properties for the $Sr_{10}(PO_4)_6(OH)_2$ bulk phase. Number of non-equivalent species, N; average distance for nearest neighbors, d_{NN}; effective coordination number, ECN; and net atomic charge, Q.

Non-equivalent species	N	d_{NN} (Å)	ECN (NNN)	Q (e^-)
Sr(I)	2	2.6114	8.8935	1.487 125
Sr(II)	2	2.5715	7.9265	1.489 916
Sr(III)	6	2.5023	7.7642	1.513 882
P(I)	6	1.5601	3.9997	1.507 718
O(I)	6	1.5639	1.0000	−0.936 883
O(II)	6	1.5668	1.0000	−0.958 865
O(III)	6	1.5624	1.0000	−0.935 053
O(IV)	6	1.5601	1.0000	−0.941 476
O(V)	2	0.9732	1.0000	−1.084 366
H(I)	2	0.9732	1.0000	0.359 361

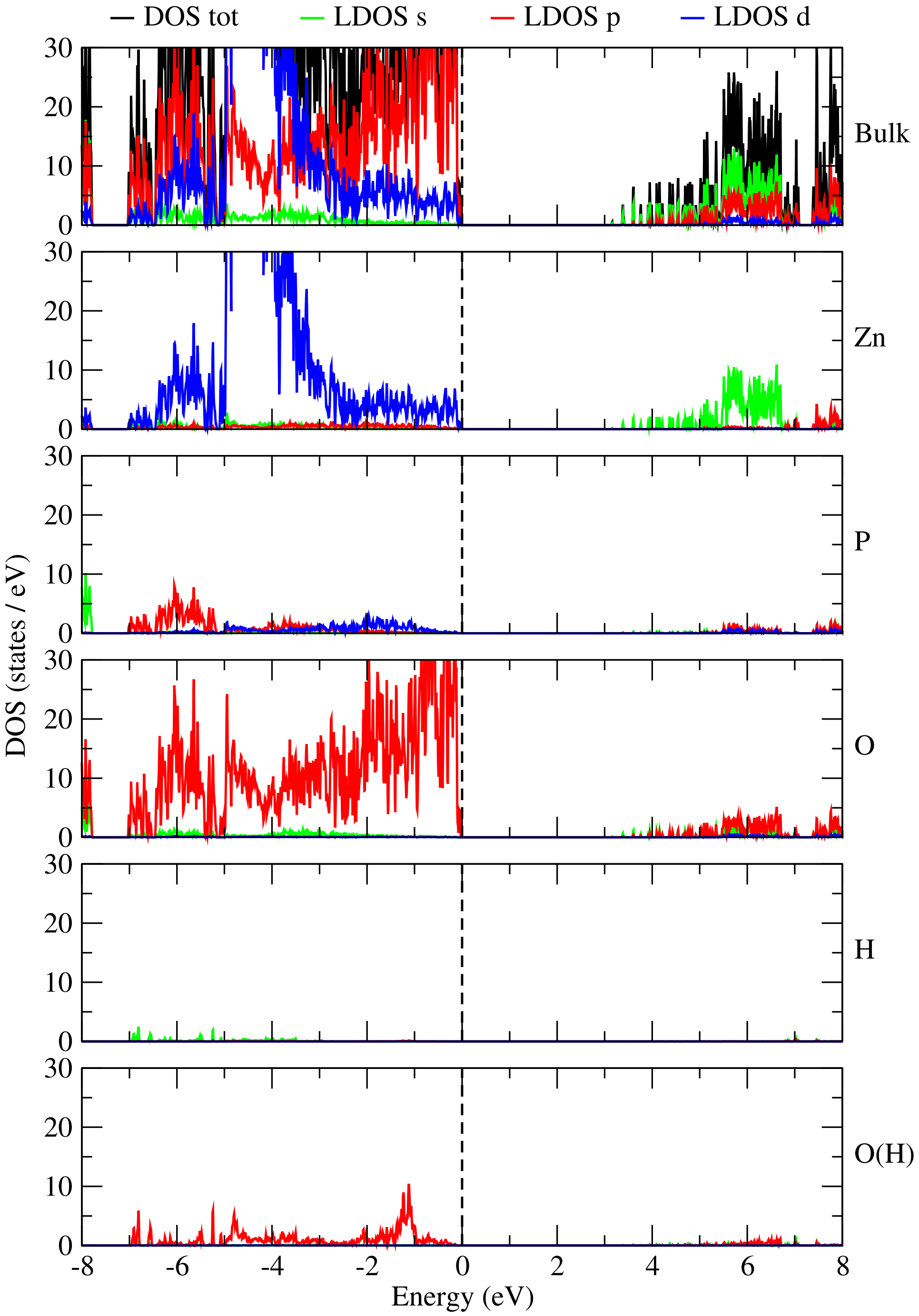

Figure S-66: Local density of states for the $Zn_{10}(PO_4)_6(OH)_2$ bulk phase.

Table S-69: Calculated properties for the $Zn_{10}(PO_4)_6(OH)_2$ bulk phase. Number of non-equivalent species, N; average distance for nearest neighbors, d_{NN}; effective coordination number, ECN; and net atomic charge, Q.

Non-equivalent species	N	d_{NN} (Å)	ECN (NNN)	Q (e^-)
Zn(I)	2	2.1333	6.0320	1.125 687
Zn(II)	2	2.1346	6.0334	1.124 420
Zn(III)	6	1.9873	5.2831	1.125 377
P(I)	6	1.5386	3.9883	1.555 173
O(I)	6	1.5386	1.0156	−0.783 983
O(II)	6	1.5760	1.0204	−0.831 576
O(III)	6	1.5701	1.1417	−0.821 176
O(IV)	6	1.5691	1.1556	−0.821 136
O(V)	2	0.9800	1.0000	−0.887 320
H(I)	2	0.9800	1.0000	0.369 183

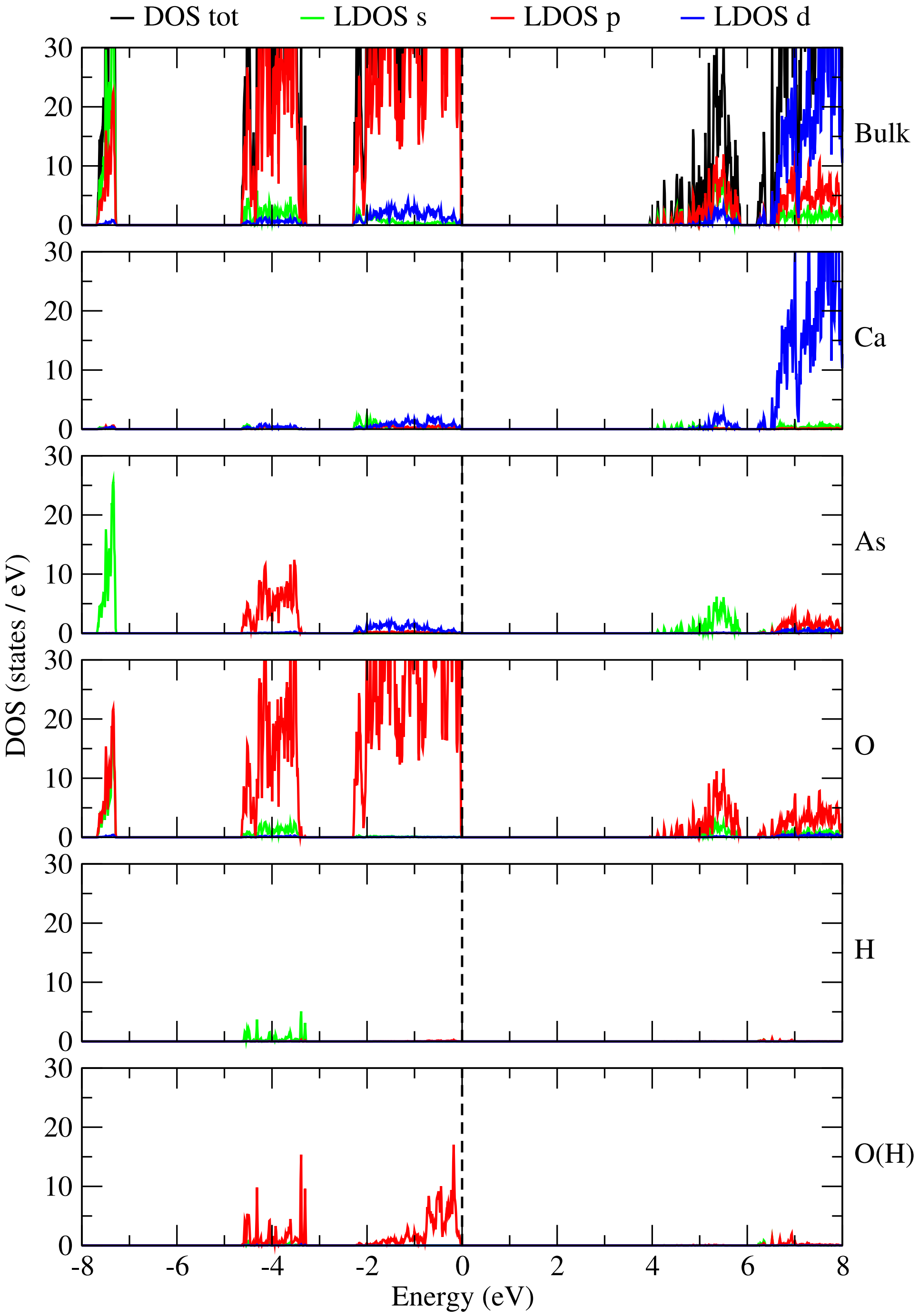

Figure S-67: Local density of states for the $Ca_{10}(AsO_4)_6(OH)_2$ bulk phase.

Table S-70: Calculated properties for the $Ca_{10}(AsO_4)_6(OH)_2$ bulk phase. Number of non-equivalent species, N; average distance for nearest neighbors, d_{NN}; effective coordination number, ECN; and net atomic charge, Q.

Non-equivalent species	N	d_{NN} (Å)	ECN (NNN)	Q (e^-)
Ca(I)	2	2.3856	6.9814	1.420 469
Ca(II)	2	2.3703	6.5789	1.421 839
Ca(III)	6	2.3408	6.6687	1.442 995
As(I)	6	1.6999	3.9947	1.675 267
O(I)	6	1.6999	1.0109	−0.918 197
O(II)	6	1.7295	1.0104	−0.996 239
O(III)	6	1.7217	1.0167	−0.958 188
O(IV)	6	1.7189	1.0166	−0.959 168
O(V)	2	0.9759	1.0000	−1.060 477
H(I)	2	0.9759	1.0000	0.358 761

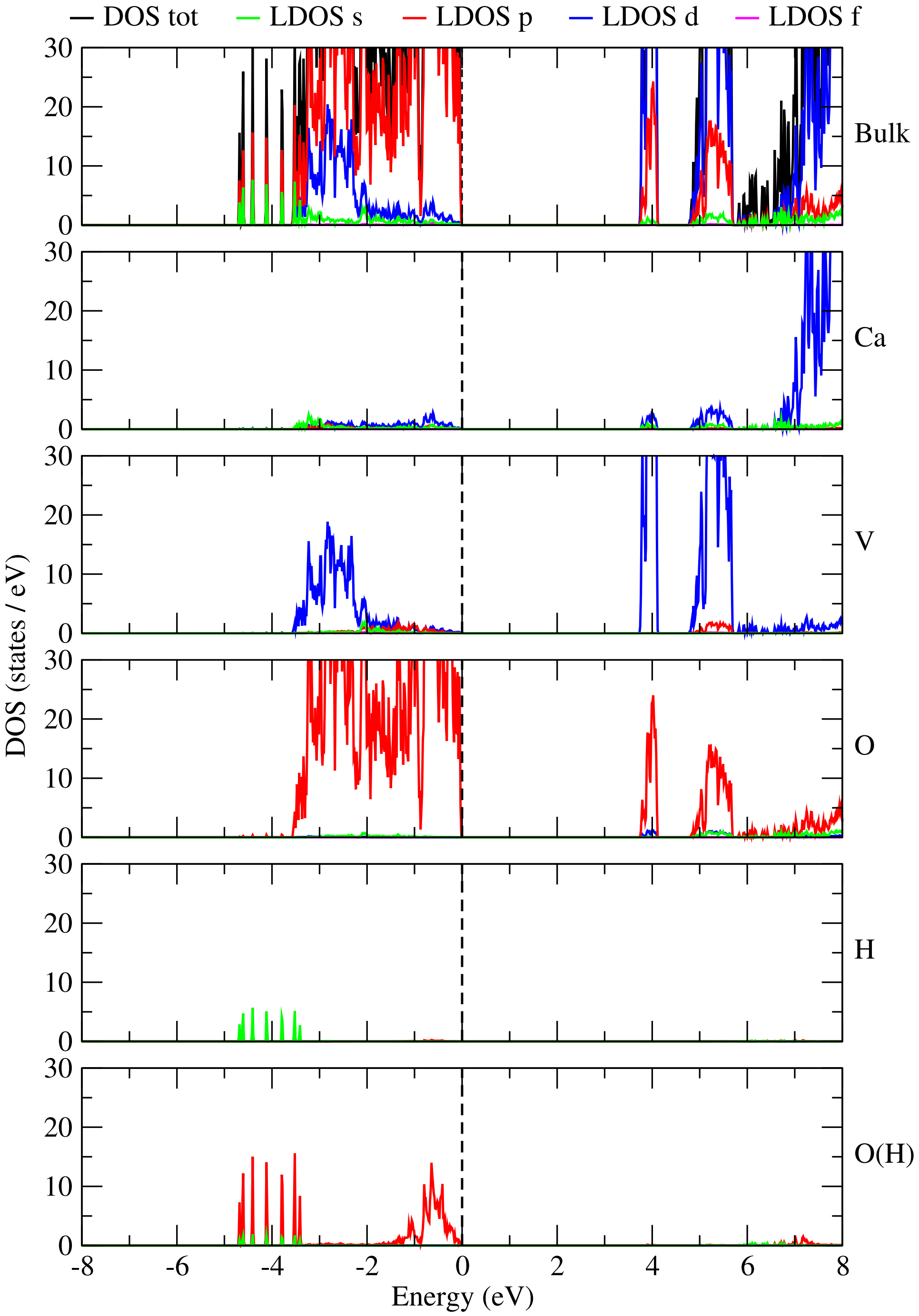

Figure S-68: Local density of states for the $Ca_{10}(VO_4)_6(OH)_2$ bulk phase.

Table S-71: Calculated properties for the $Ca_{10}(VO_4)_6(OH)_2$ bulk phase. Number of non-equivalent species, N; average distance for nearest neighbors, d_{NN}; effective coordination number, ECN; and net atomic charge, Q.

Non-equivalent species	N	d_{NN} (Å)	ECN (NNN)	Q (e^-)
Ca(I)	2	2.3821	6.7190	1.472 580
Ca(II)	2	2.3738	6.5165	1.472 317
Ca(III)	6	2.3313	6.6162	1.476 553
V(I)	6	1.7037	3.9919	1.985 667
O(I)	6	1.7037	1.0119	−1.012 300
O(II)	6	1.7412	1.0170	−1.128 111
O(III)	6	1.7187	1.0196	−1.036 224
O(IV)	6	1.7167	1.0189	−1.036 237
O(V)	2	0.9762	1.0000	−1.053 025
H(I)	2	0.9762	1.0000	0.360 088

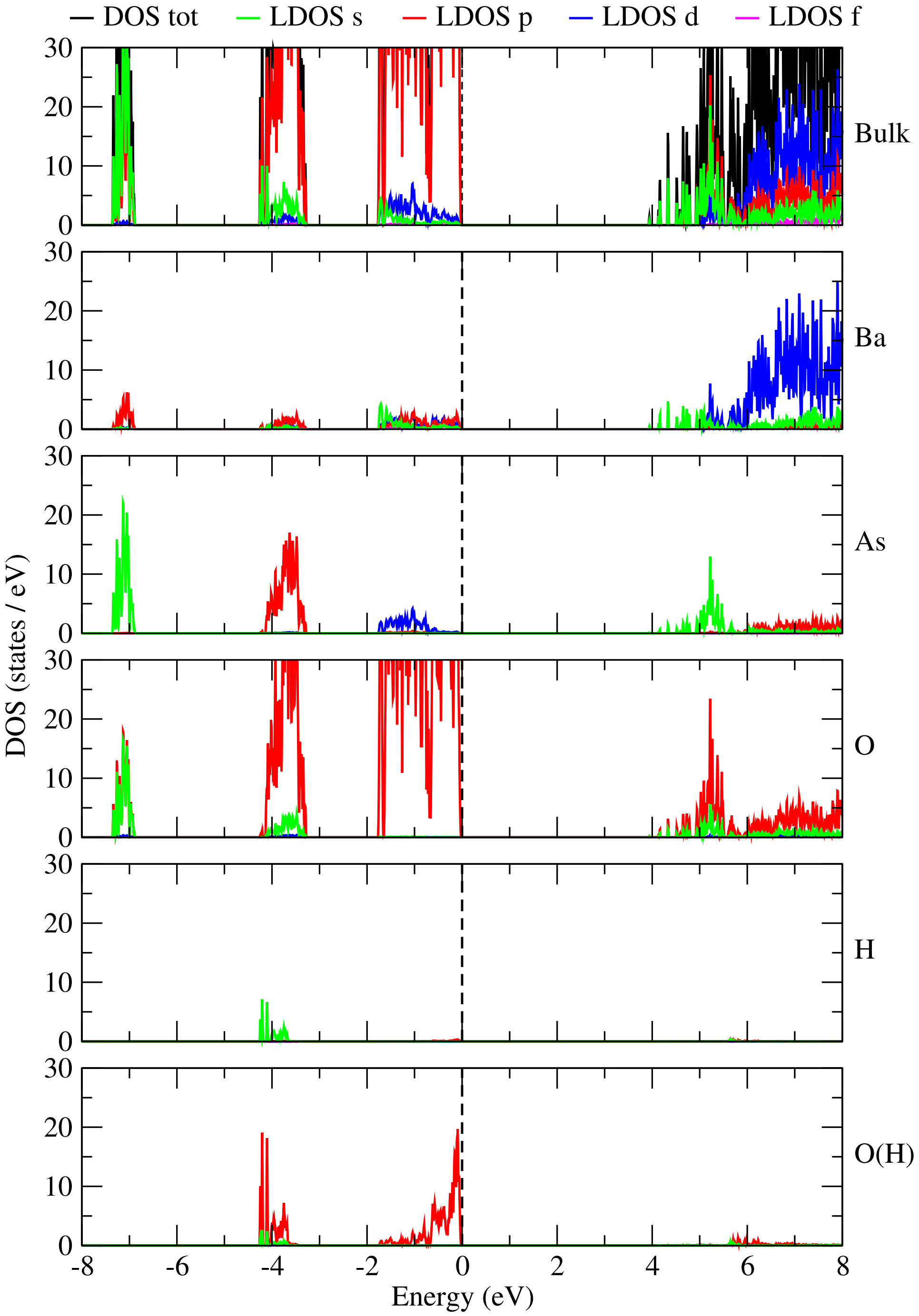

Figure S-69: Local density of states for the $Ba_{10}(AsO_4)_6(OH)_2$ bulk phase.

Table S-72: Calculated properties for the $Ba_{10}(AsO_4)_6(OH)_2$ bulk phase. Number of non-equivalent species, N; average distance for nearest neighbors, d_{NN}; effective coordination number, ECN; and net atomic charge, Q.

Non-equivalent species	N	d_{NN} (Å)	ECN (NNN)	Q (e^-)
Ba(I)	2	2.7902	8.8421	1.439 357
Ba(II)	2	2.7491	7.9037	1.441 849
Ba(III)	6	2.6800	7.7602	1.467 902
As(I)	6	1.7216	3.9997	1.650 621
O(I)	6	1.7238	1.0000	−0.957 028
O(II)	6	1.7288	1.0000	−0.977 276
O(III)	6	1.7234	1.0000	−0.951 888
O(IV)	6	1.7216	1.0000	−0.957 079
O(V)	2	0.9734	1.0000	−1.062 136
H(I)	2	0.9734	1.0000	0.355 179

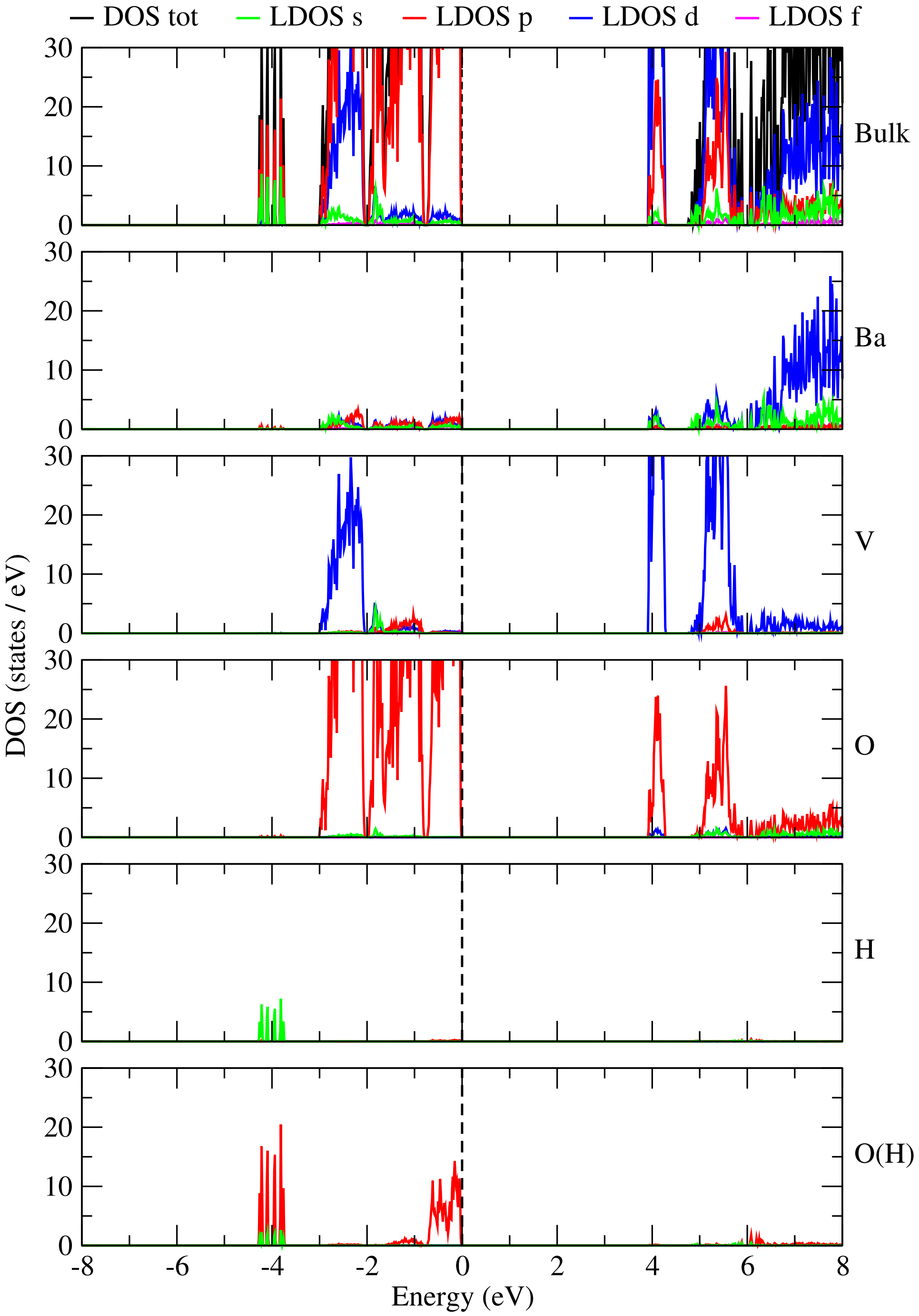

Figure S-70: Local density of states for the $Ba_{10}(VO_4)_6(OH)_2$ bulk phase.

Table S-73: Calculated properties for the $Ba_{10}(VO_4)_6(OH)_2$ bulk phase. Number of non-equivalent species, N; average distance for nearest neighbors, d_{NN}; effective coordination number, ECN; and net atomic charge, Q.

Non-equivalent species	N	d_{NN} (Å)	ECN (NNN)	Q (e^-)
Ba(I)	2	2.7688	8.7813	1.498 015
Ba(II)	2	2.7371	7.9834	1.499 039
Ba(III)	6	2.6580	7.7907	1.503 372
V(I)	6	1.7154	3.9979	1.963 229
O(I)	6	1.7325	1.0000	−1.077 901
O(II)	6	1.7290	1.0000	−1.089 824
O(III)	6	1.7197	1.0000	−1.031 261
O(IV)	6	1.7154	1.0000	−1.032 816
O(V)	2	0.9735	1.0000	−1.059 008
H(I)	2	0.9735	1.0000	0.357 557

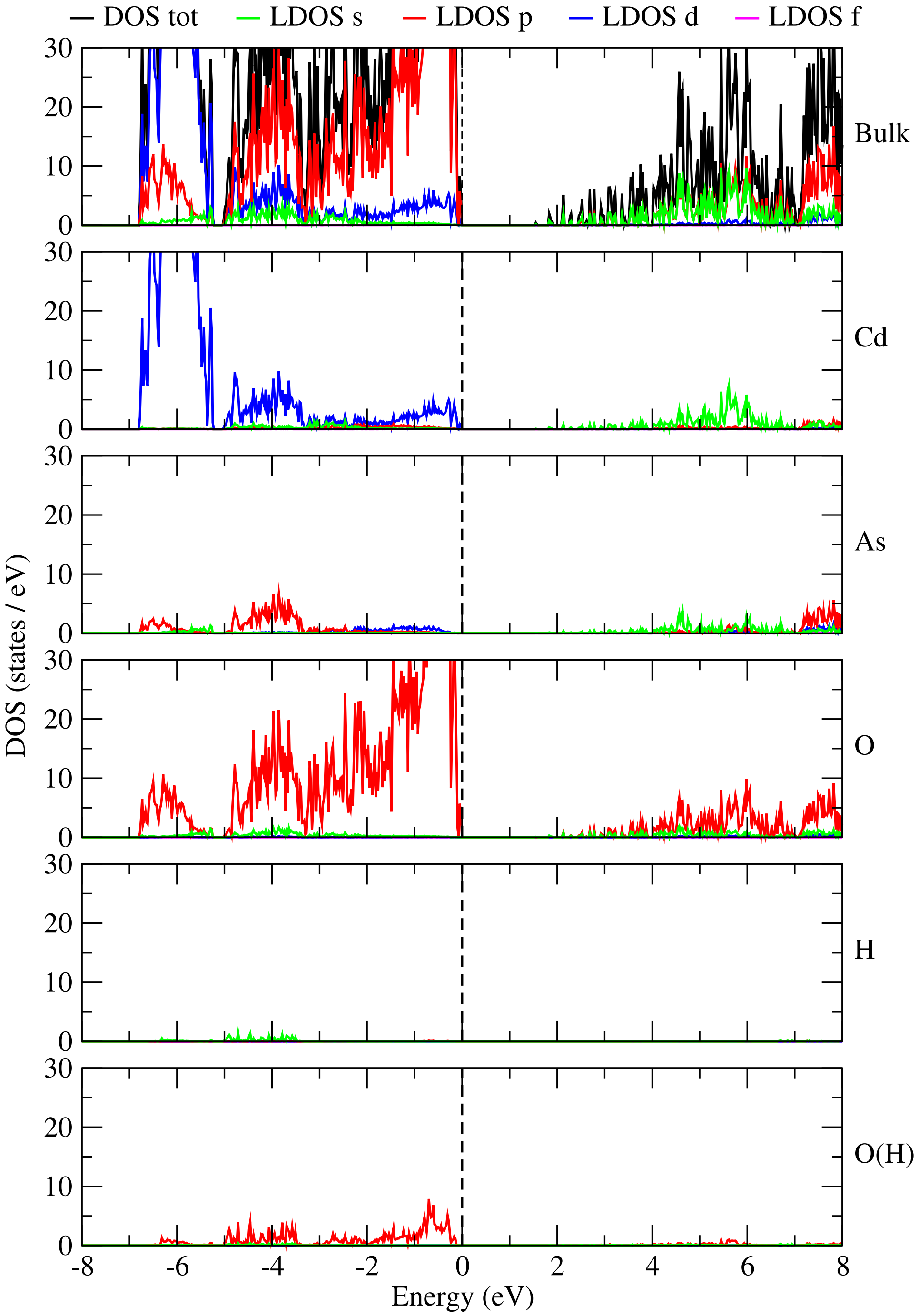

Figure S-71: Local density of states for the $Cd_{10}(AsO_4)_6(OH)_2$ bulk phase.

Table S-74: Calculated properties for the $Cd_{10}(AsO_4)_6(OH)_2$ bulk phase. Number of non-equivalent species, N; average distance for nearest neighbors, d_{NN}; effective coordination number, ECN; and net atomic charge, Q.

Non-equivalent species	N	d_{NN} (Å)	ECN (NNN)	Q (e^-)
Cd(I)	2	2.3214	6.1974	1.125 863
Cd(II)	2	2.3155	6.1153	1.124 192
Cd(III)	6	2.2417	6.3192	1.144 407
As(I)	6	1.6973	3.9870	1.663 391
O(I)	6	1.6973	1.0292	−0.807 019
O(II)	6	1.7445	1.0246	−0.870 308
O(III)	6	1.7278	1.0787	−0.852 222
O(IV)	6	1.7269	1.0838	−0.852 141
O(V)	2	0.9778	1.0000	−0.888 049
H(I)	2	0.9778	1.0000	0.359 676

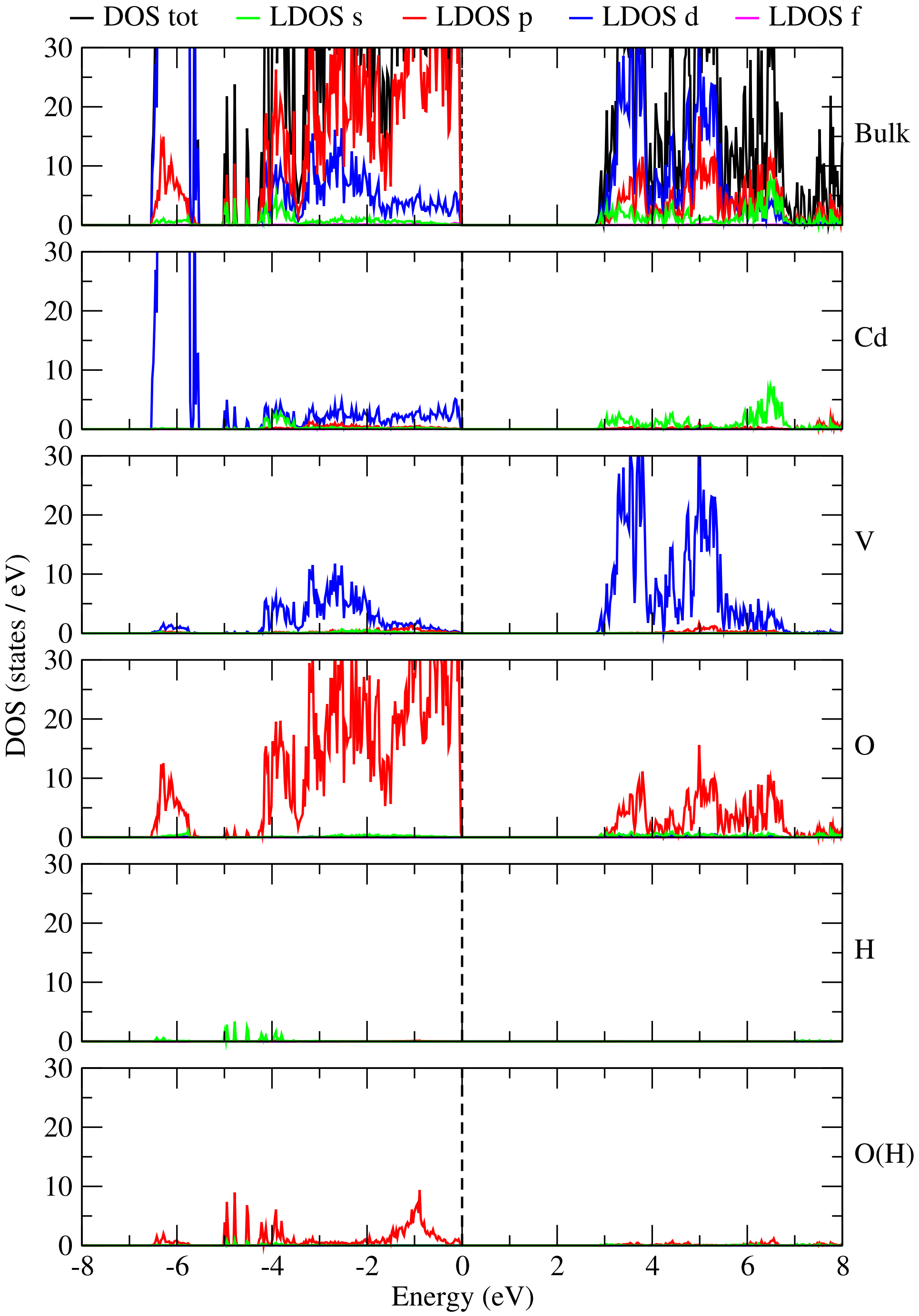

Figure S-72: Local density of states for the $Cd_{10}(VO_4)_6(OH)_2$ bulk phase.

Table S-75: Calculated properties for the $Cd_{10}(VO_4)_6(OH)_2$ bulk phase. Number of non-equivalent species, N; average distance for nearest neighbors, d_{NN}; effective coordination number, ECN; and net atomic charge, Q.

Non-equivalent species	N	d_{NN} (Å)	ECN (NNN)	Q (e^-)
Cd(I)	2	2.3236	6.0919	1.187 028
Cd(II)	2	2.3214	6.0651	1.184 541
Cd(III)	6	2.2017	6.1067	1.199 602
V(I)	6	1.6969	3.9731	1.976 286
O(I)	6	1.6969	1.0269	−0.887 729
O(II)	6	1.7657	1.0518	−1.016 208
O(III)	6	1.7234	1.1093	−0.946 276
O(IV)	6	1.7207	1.1161	−0.944 466
O(V)	2	0.9773	1.0000	−0.878 709
H(I)	2	0.9773	1.0000	0.363 514

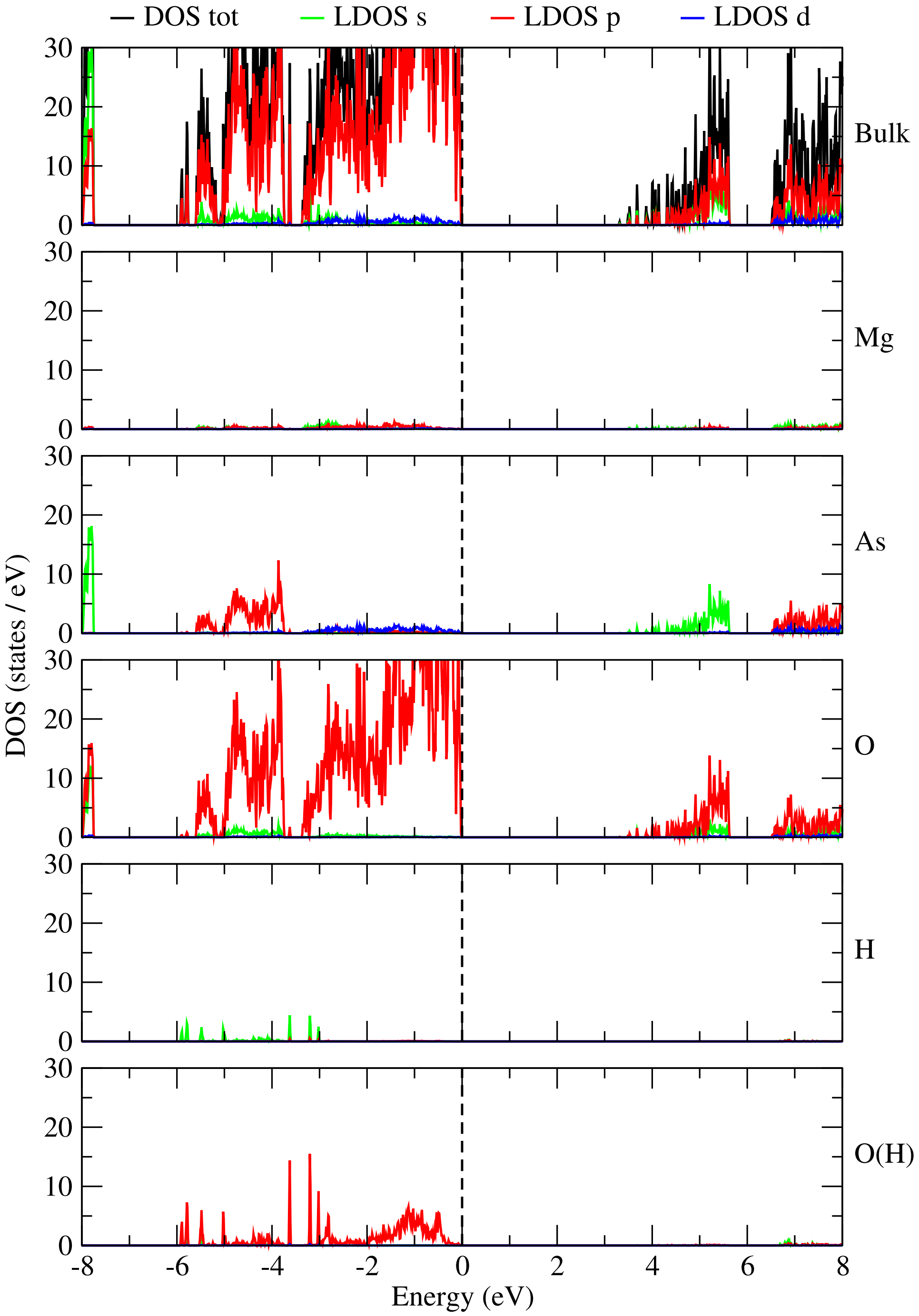

Figure S-73: Local density of states for the $Mg_{10}(AsO_4)_6(OH)_2$ bulk phase.

Table S-76: Calculated properties for the $Mg_{10}(AsO_4)_6(OH)_2$ bulk phase. Number of non-equivalent species, N; average distance for nearest neighbors, d_{NN}; effective coordination number, ECN; and net atomic charge, Q.

Non-equivalent species	N	d_{NN} (Å)	ECN (NNN)	Q (e^-)
Mg(I)	2	2.1035	5.9642	1.488 418
Mg(II)	2	2.1035	5.9676	1.487 680
Mg(III)	6	2.0328	5.4601	1.463 632
As(I)	6	1.6836	3.9810	1.762 482
O(I)	6	1.6836	1.5960	−0.946 718
O(II)	6	1.7362	1.5722	−1.029 487
O(III)	6	1.7279	2.0063	−1.003 608
O(IV)	6	1.7254	1.9737	−1.001 469
O(V)	2	0.9799	1.0000	−1.072 702
H(I)	2	0.9799	1.0000	0.362 108

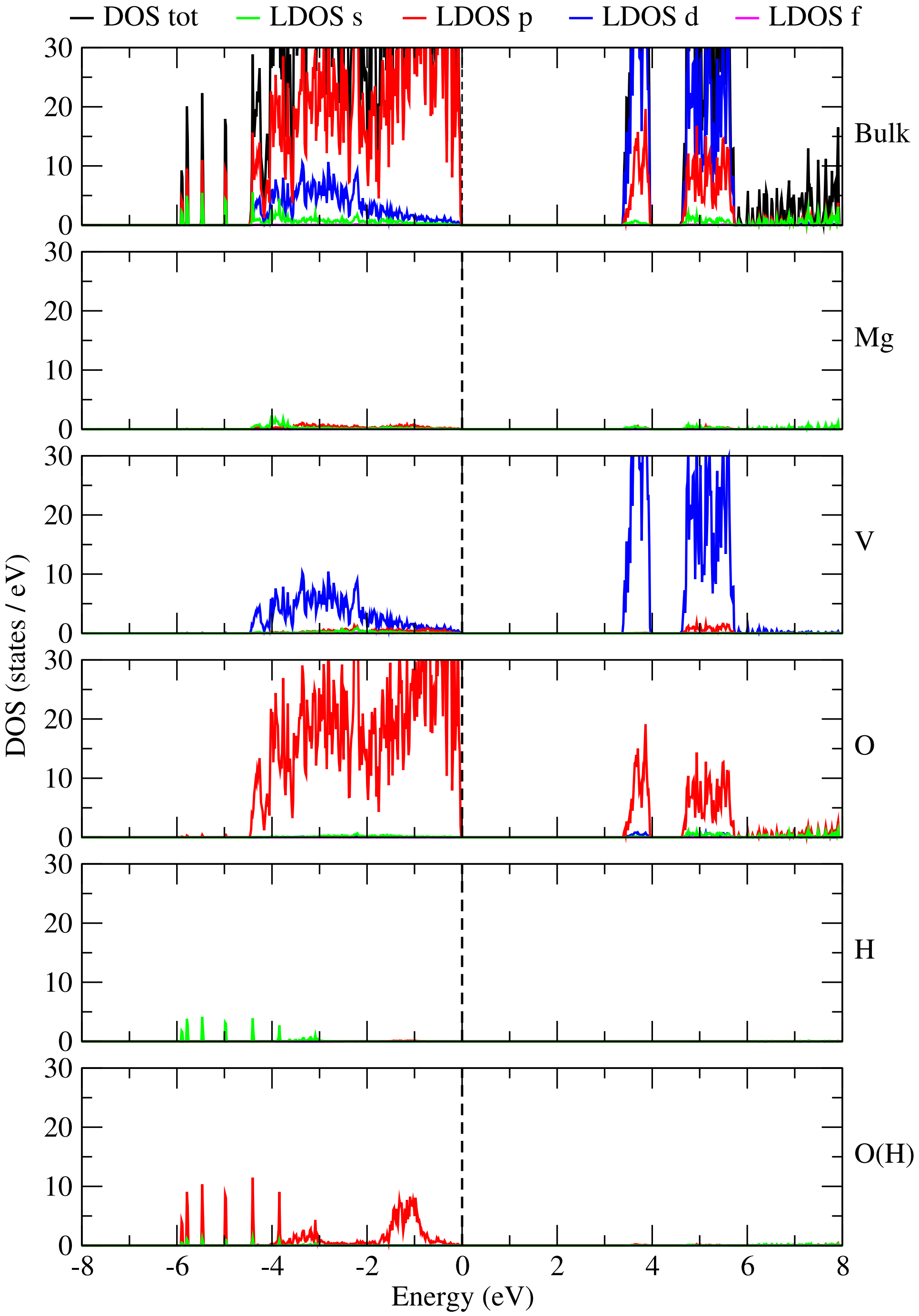

Figure S-74: Local density of states for the $Mg_{10}(VO_4)_6(OH)_2$ bulk phase.

Table S-77: Calculated properties for the $Mg_{10}(VO_4)_6(OH)_2$ bulk phase. Number of non-equivalent species, N; average distance for nearest neighbors, d_{NN}; effective coordination number, ECN; and net atomic charge, Q.

Non-equivalent species	N	d_{NN} (Å)	ECN (NNN)	Q (e^-)
Mg(I)	2	2.1189	5.9885	1.525 707
Mg(II)	2	2.1187	5.9897	1.525 220
Mg(III)	6	2.0218	5.5340	1.495 345
V(I)	6	1.6873	3.9748	2.051 120
O(I)	6	1.6873	1.5251	−1.008 861
O(II)	6	1.7544	1.7398	−1.171 777
O(III)	6	1.7233	1.9477	−1.077 083
O(IV)	6	1.7224	1.9335	−1.075 085
O(V)	2	0.9802	1.0000	−1.056 033
H(I)	2	0.9802	1.0000	0.364 129

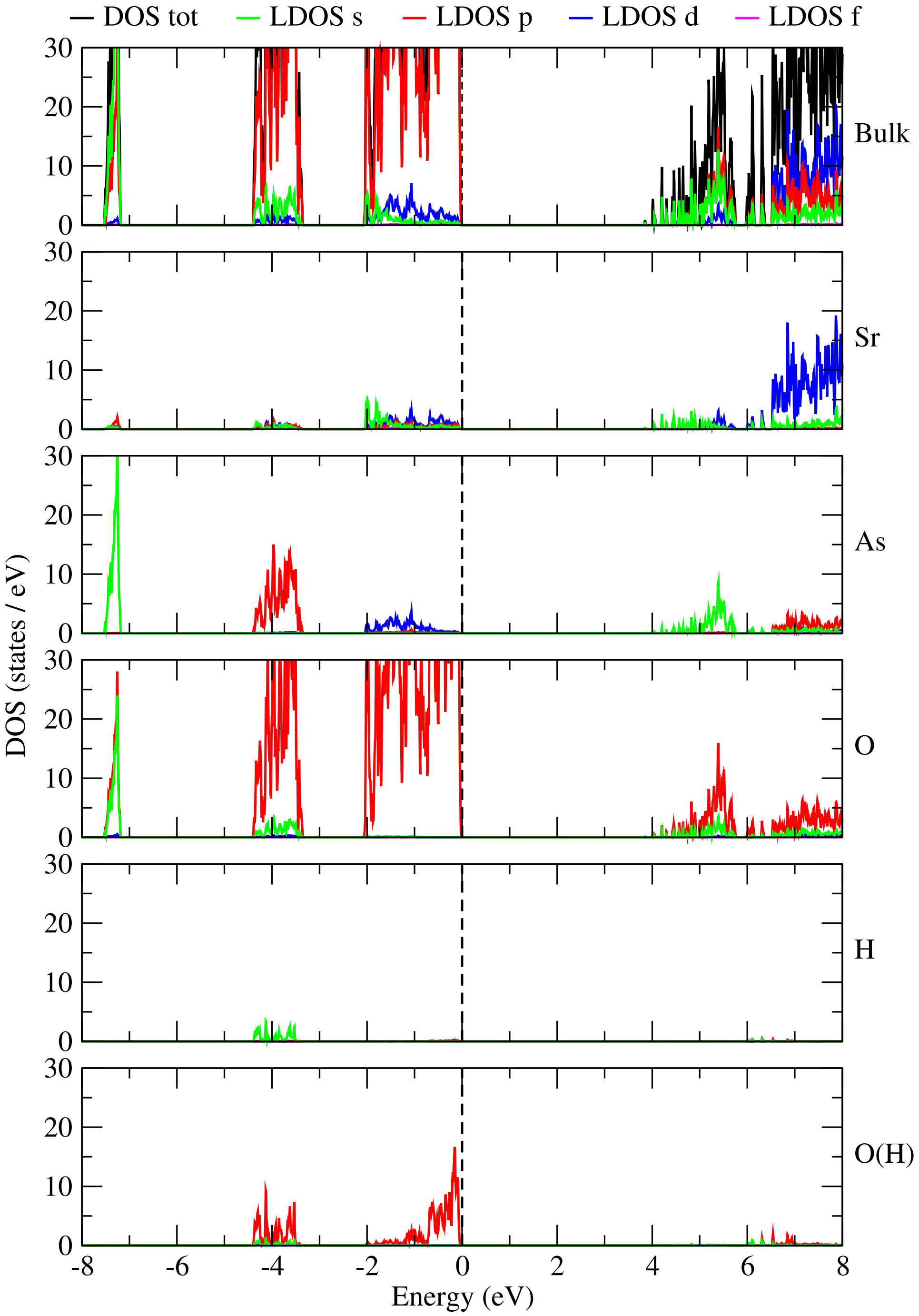

Figure S-75: Local density of states for the $Sr_{10}(AsO_4)_6(OH)_2$ bulk phase.

Table S-78: Calculated properties for the $Sr_{10}(AsO_4)_6(OH)_2$ bulk phase. Number of non-equivalent species, N; average distance for nearest neighbors, d_{NN}; effective coordination number, ECN; and net atomic charge, Q.

Non-equivalent species	N	d_{NN} (Å)	ECN (NNN)	Q (e^-)
Sr(I)	2	2.6065	8.4285	1.452 913
Sr(II)	2	2.5704	7.4653	1.456 972
Sr(III)	6	2.5205	7.4522	1.483 820
As(I)	6	1.7175	3.9995	1.653 030
O(I)	6	1.7192	1.0002	−0.946 081
O(II)	6	1.7262	1.0007	−0.995 934
O(III)	6	1.7199	1.0004	−0.959 367
O(IV)	6	1.7175	1.0005	−0.964 276
O(V)	2	0.9742	1.0000	−1.080 827
H(I)	2	0.9742	1.0000	0.357 367

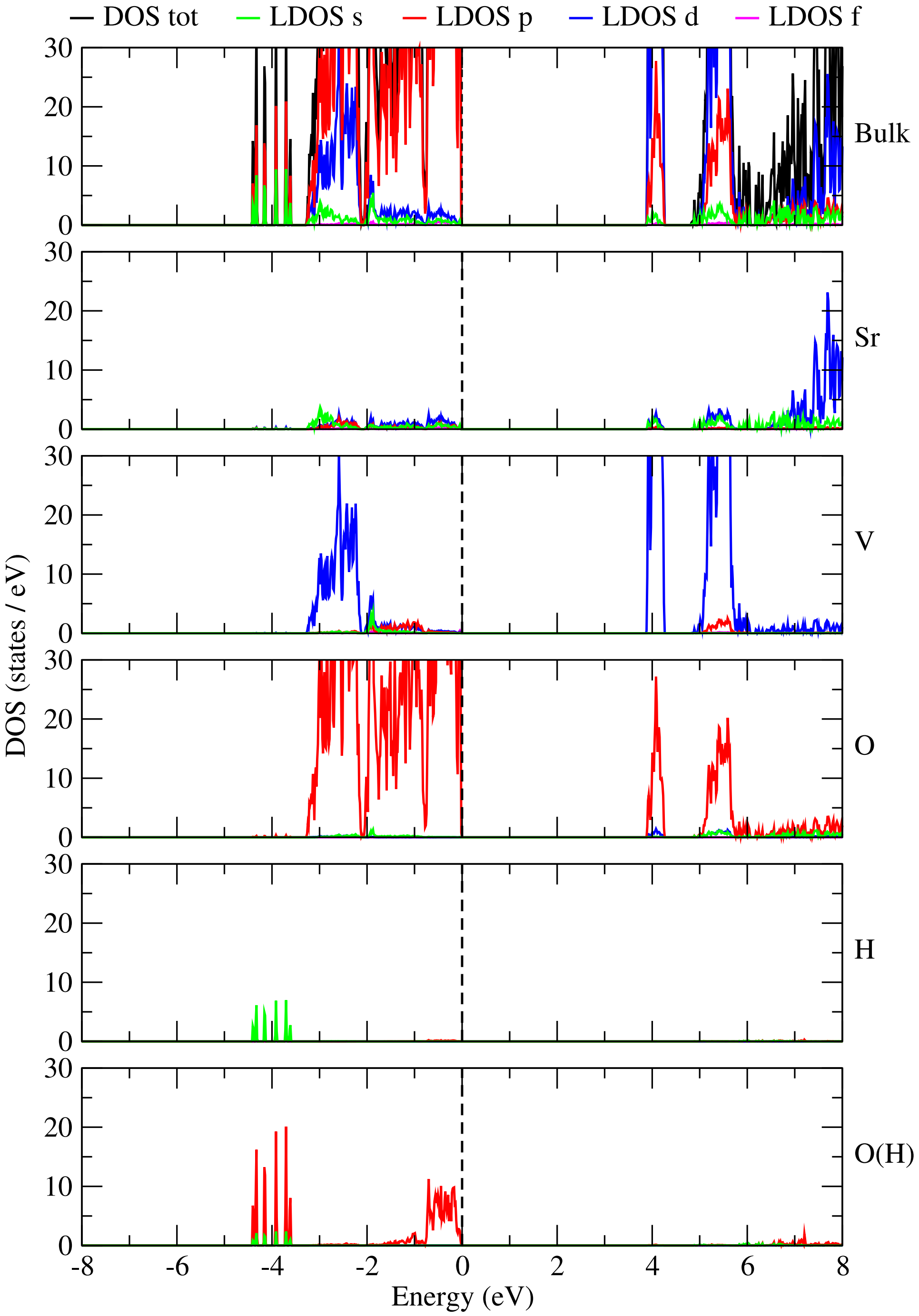

Figure S-76: Local density of states for the $Sr_{10}(VO_4)_6(OH)_2$ bulk phase.

Table S-79: Calculated properties for the $Sr_{10}(VO_4)_6(OH)_2$ bulk phase. Number of non-equivalent species, N; average distance for nearest neighbors, d_{NN}; effective coordination number, ECN; and net atomic charge, Q.

Non-equivalent species	N	d_{NN} (Å)	ECN (NNN)	Q (e^-)
Sr(I)	2	2.5942	8.2479	1.512 447
Sr(II)	2	2.5644	7.4111	1.514 679
Sr(III)	6	2.5164	7.4847	1.518 167
V(I)	6	1.7120	3.9972	1.967 282
O(I)	6	1.7268	1.0003	−1.066 957
O(II)	6	1.7313	1.0009	−1.117 080
O(III)	6	1.7151	1.0004	−1.035 713
O(IV)	6	1.7120	1.0004	−1.036 900
O(V)	2	0.9743	1.0000	−1.074 421
H(I)	2	0.9743	1.0000	0.360 899

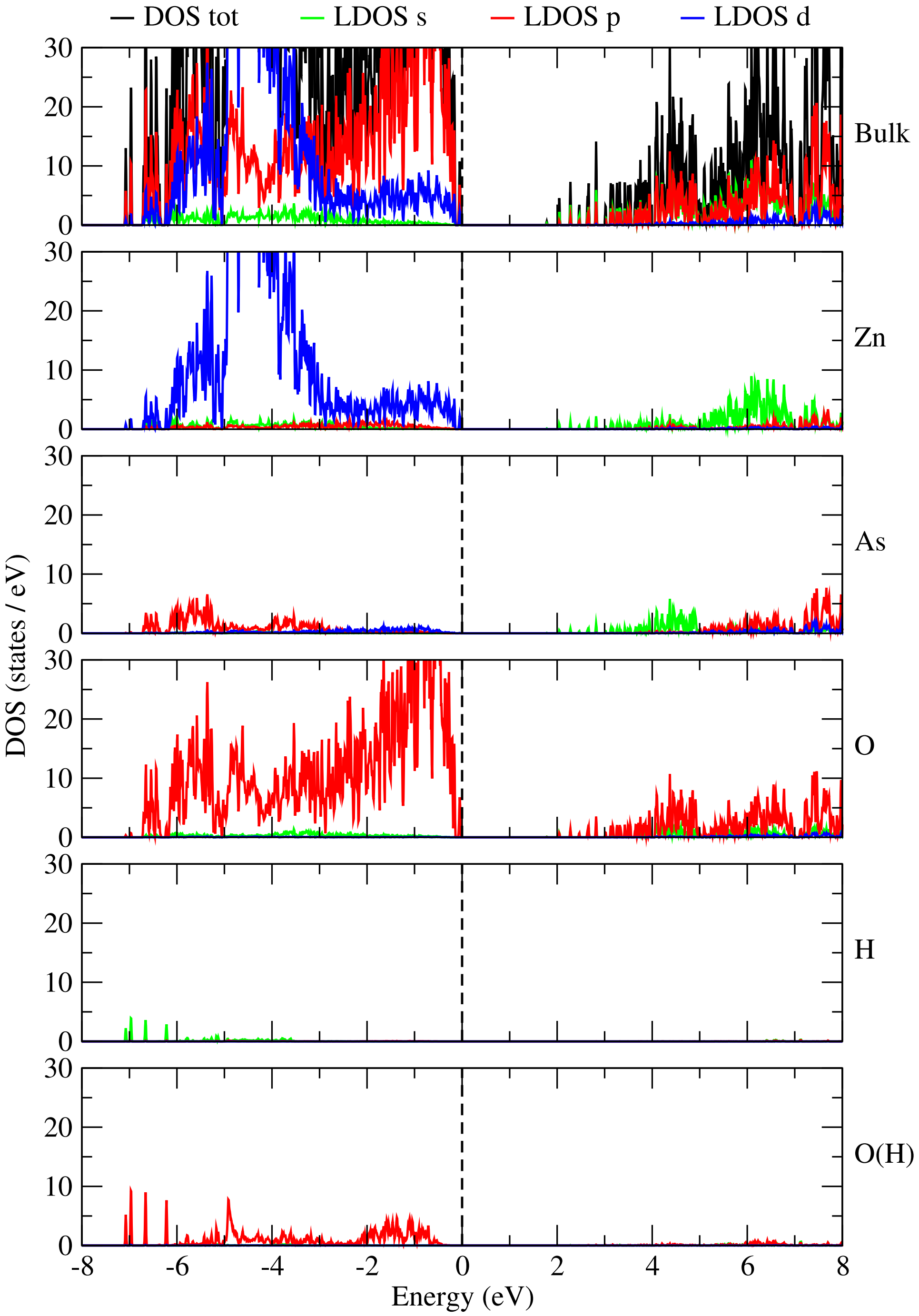

Figure S-77: Local density of states for the $Zn_{10}(AsO_4)_6(OH)_2$ bulk phase.

Table S-80: Calculated properties for the $Zn_{10}(AsO_4)_6(OH)_2$ bulk phase. Number of non-equivalent species, N; average distance for nearest neighbors, d_{NN}; effective coordination number, ECN; and net atomic charge, Q.

Non-equivalent species	N	d_{NN} (Å)	ECN (NNN)	Q (e^-)
Zn(I)	2	2.1289	6.0148	1.097 996
Zn(II)	2	2.1273	6.0141	1.096 802
Zn(III)	6	1.9941	5.1903	1.060 032
As(I)	6	1.6965	3.9869	1.712 476
O(I)	6	1.6965	1.5398	−0.804 367
O(II)	6	1.7368	1.6333	−0.862 686
O(III)	6	1.7362	2.0846	−0.836 044
O(IV)	6	1.7345	2.0445	−0.835 396
O(V)	2	0.9801	1.0000	−0.867 314
H(I)	2	0.9801	1.0000	0.370 471

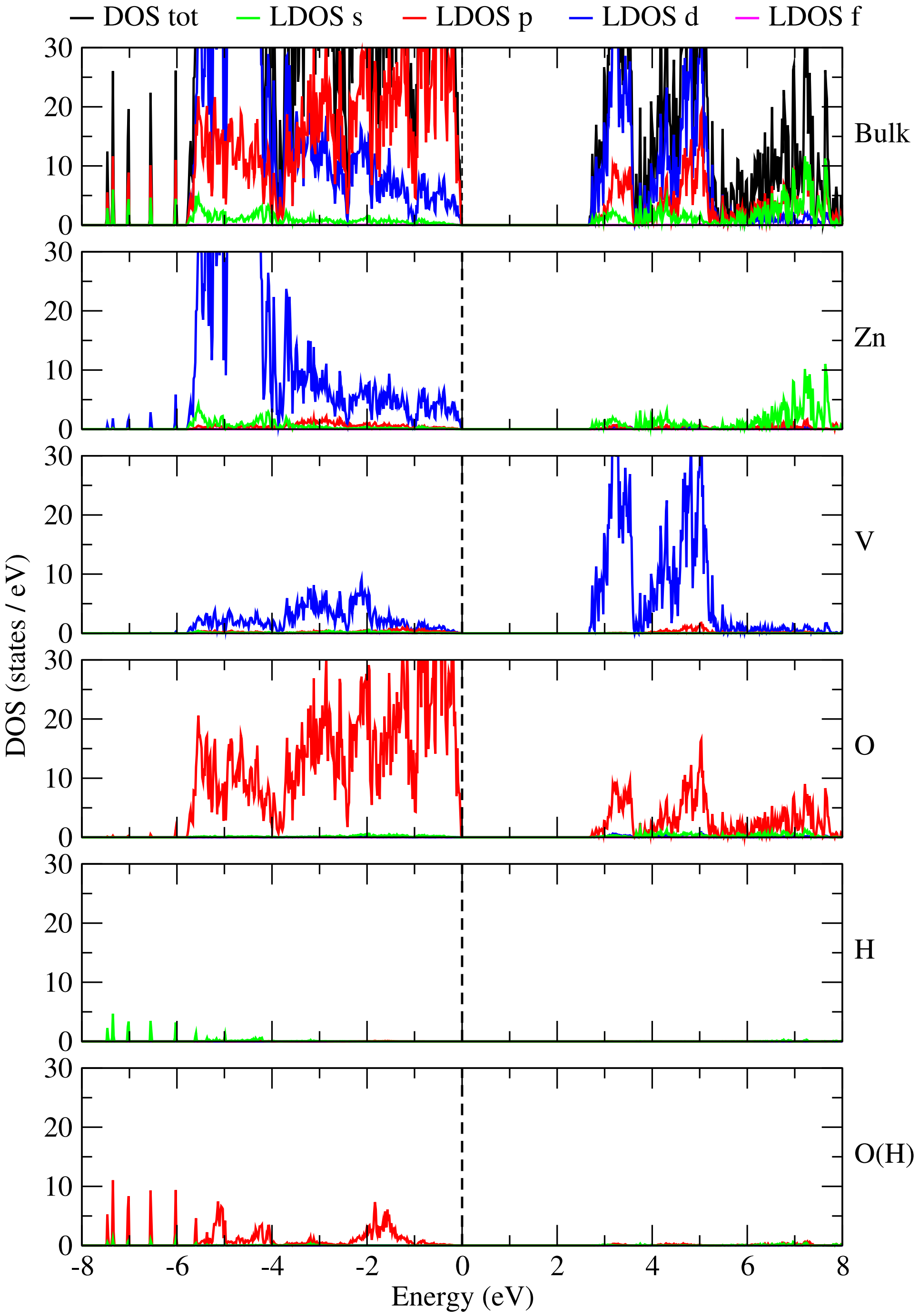

Figure S-78: Local density of states for the $Zn_{10}(VO_4)_6(OH)_2$ bulk phase.

Table S-81: Calculated properties for the $Zn_{10}(VO_4)_6(OH)_2$ bulk phase. Number of non-equivalent species, N; average distance for nearest neighbors, d_{NN}; effective coordination number, ECN; and net atomic charge, Q.

Non-equivalent species	N	d_{NN} (Å)	ECN (NNN)	Q (e^-)
Zn(I)	2	2.1407	6.0307	1.165 854
Zn(II)	2	2.1394	6.0301	1.164 686
Zn(III)	6	1.9515	4.9333	1.101 338
V(I)	6	1.7042	3.9918	2.009 081
O(I)	6	1.7042	1.5250	−0.889 354
O(II)	6	1.7392	1.7149	−0.981 247
O(III)	6	1.7346	2.0809	−0.931 059
O(IV)	6	1.7289	1.9840	−0.926 171
O(V)	2	0.9804	1.0000	−0.851 927
H(I)	2	0.9804	1.0000	0.373 624

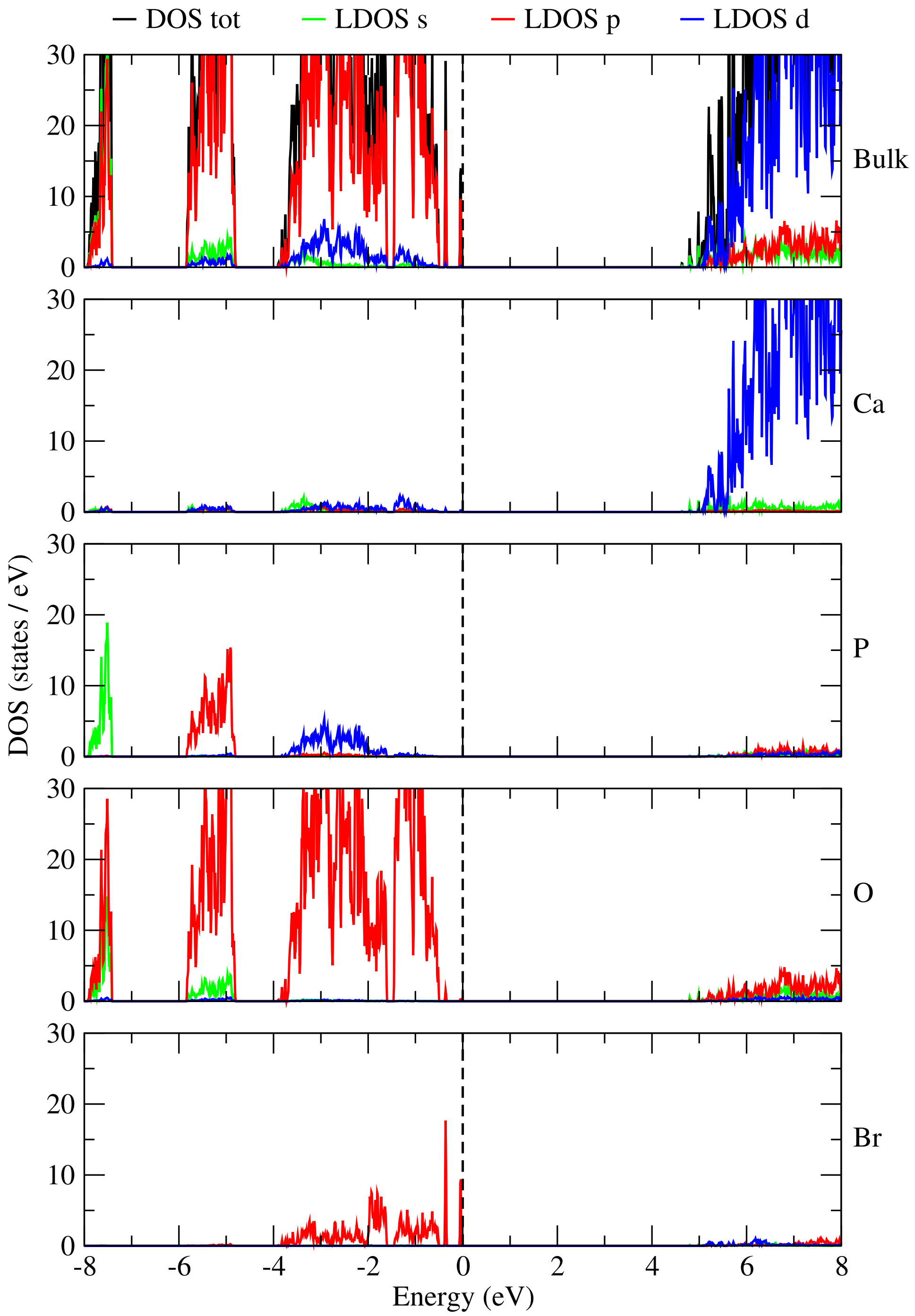

Figure S-79: Local density of states for the $Ca_{10}(PO_4)_6Br_2$ bulk phase.

Table S-82: Calculated properties for the $Ca_{10}(PO_4)_6Br_2$ bulk phase. Number of non-equivalent species, N; average distance for nearest neighbors, d_{NN}; effective coordination number, ECN; and net atomic charge, Q.

Non-equivalent species	N	d_{NN} (Å)	ECN (NNN)	Q (e^-)
Ca(I)	2	2.4217	7.6367	1.443 198
Ca(II)	2	2.4109	7.3371	1.443 859
Ca(III)	6	2.2894	5.0490	1.460 584
P(I)	6	1.5506	3.9949	1.541 166
O(I)	6	1.5506	1.0000	−0.899 175
O(II)	6	1.5780	1.0009	−0.963 247
O(III)	6	1.5618	1.0000	−0.930 051
O(IV)	6	1.5614	1.0001	−0.931 705
Br(I)	2	3.0436	13.7458	−0.719 772

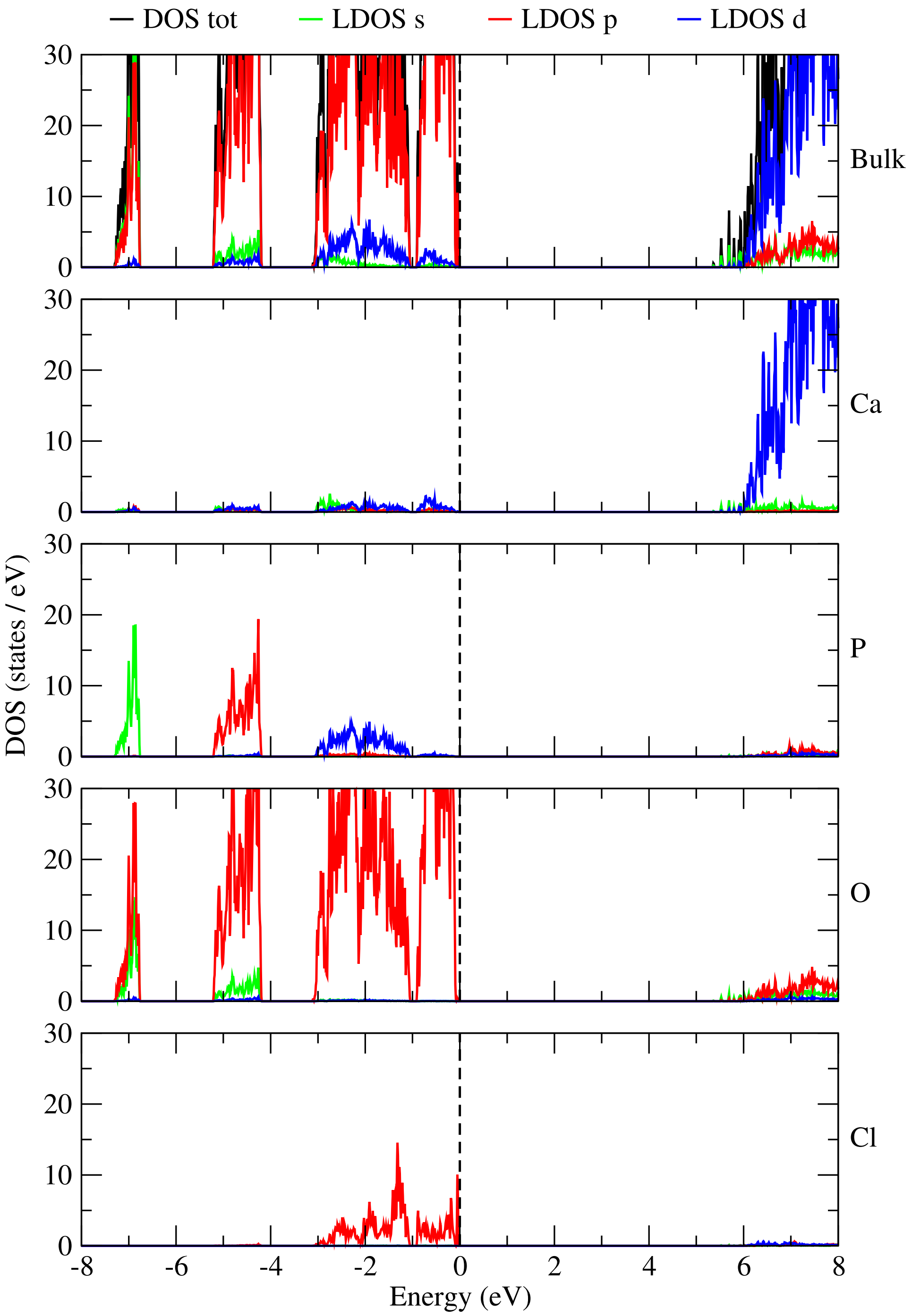

Figure S-80: Local density of states for the $Ca_{10}(PO_4)_6Cl_2$ bulk phase.

Table S-83: Calculated properties for the $Ca_{10}(PO_4)_6Cl_2$ bulk phase. Number of non-equivalent species, N; average distance for nearest neighbors, d_{NN}; effective coordination number, ECN; and net atomic charge, Q.

Non-equivalent species	N	d_{NN} (Å)	ECN (NNN)	Q (e^-)
Ca(I)	2	2.4368	8.0266	1.440 767
Ca(II)	2	2.3968	6.9227	1.444 443
Ca(III)	6	2.3036	5.4640	1.467 365
P(I)	6	1.5512	3.9955	1.536 229
O(I)	6	1.5512	1.0000	−0.902 255
O(II)	6	1.5764	1.0006	−0.964 538
O(III)	6	1.5608	1.0001	−0.928 751
O(IV)	6	1.5595	1.0001	−0.932 502
Cl(I)	2	2.7505	11.0195	−0.711 852

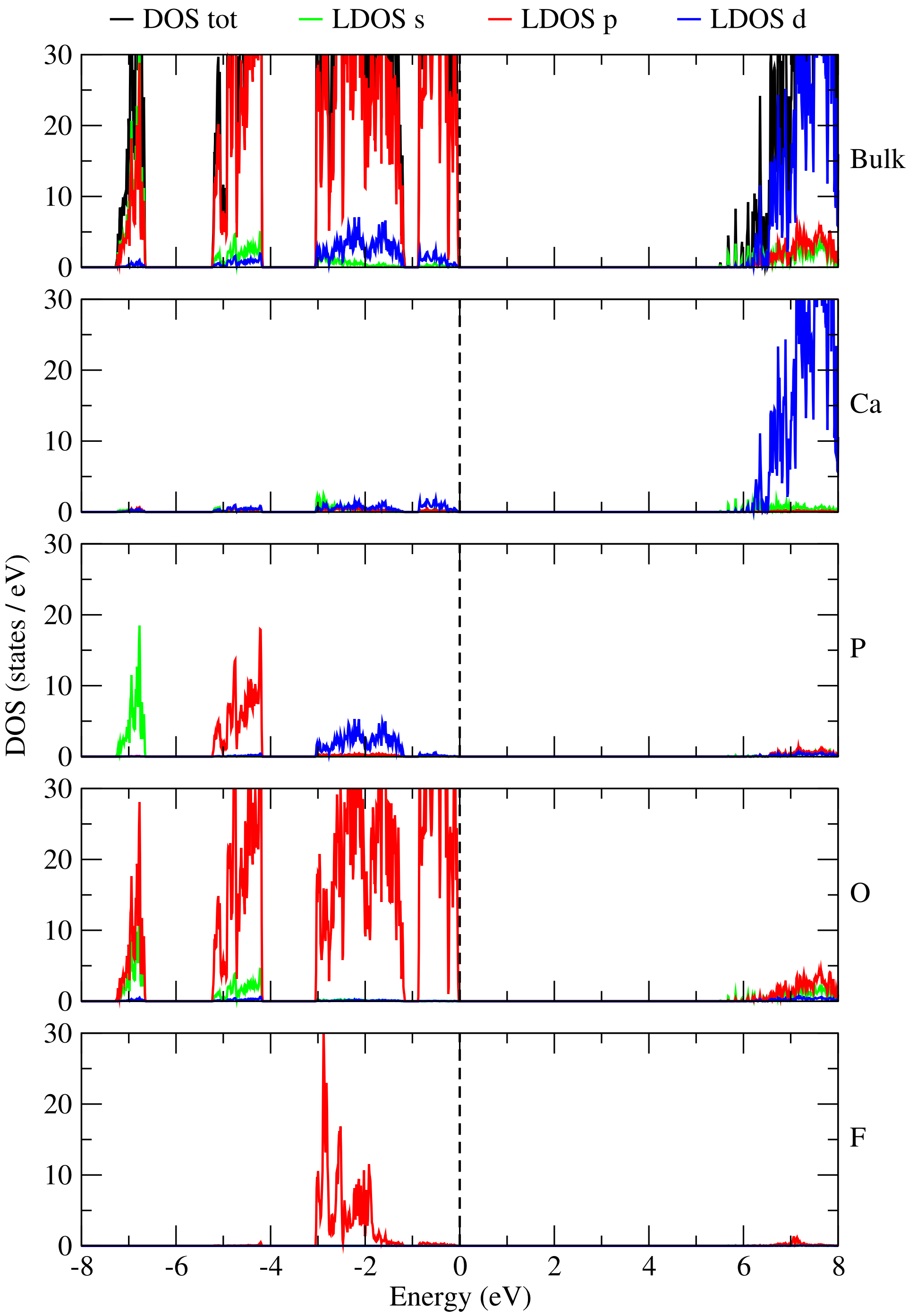

Figure S-81: Local density of states for the $Ca_{10}(PO_4)_6F_2$ bulk phase.

Table S-84: Calculated properties for the $Ca_{10}(PO_4)_6F_2$ bulk phase. Number of non-equivalent species, N; average distance for nearest neighbors, d_{NN}; effective coordination number, ECN; and net atomic charge, Q.

Non-equivalent species	N	d_{NN} (Å)	ECN (NNN)	Q (e^-)
Ca(I)	2	2.4122	7.8482	1.446 490
Ca(II)	2	2.4014	7.5505	1.447 901
Ca(III)	6	2.3231	6.4704	1.479 939
P(I)	6	1.5528	3.9988	1.511 274
O(I)	6	1.5528	1.0000	−0.908 113
O(II)	6	1.5659	1.0000	−0.947 039
O(III)	6	1.5581	1.0001	−0.930 225
O(IV)	6	1.5575	1.0001	−0.930 421
F(I)	2	2.3231	3.0906	−0.720 637

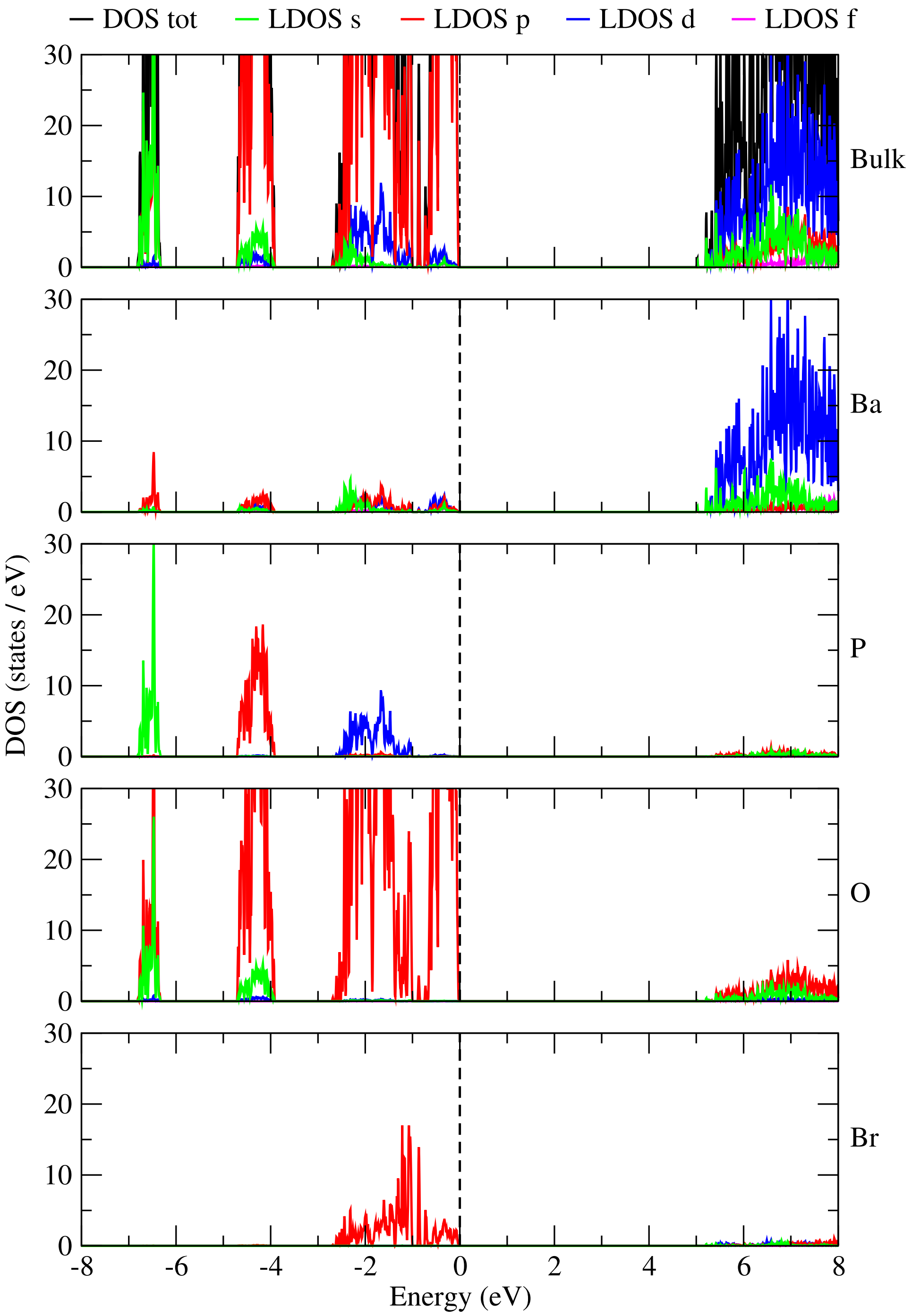

Figure S-82: Local density of states for the $Ba_{10}(PO_4)_6Br_2$ bulk phase.

Table S-85: Calculated properties for the $Ba_{10}(PO_4)_6Br_2$ bulk phase. Number of non-equivalent species, N; average distance for nearest neighbors, d_{NN}; effective coordination number, ECN; and net atomic charge, Q.

Non-equivalent species	N	d_{NN} (Å)	ECN (NNN)	Q (e^-)
Ba(I)	2	2.7381	9.1780	1.466 490
Ba(II)	2	2.7364	9.1368	1.466 670
Ba(III)	6	2.5847	6.4898	1.486 736
P(I)	6	1.5629	3.9987	1.513 019
O(I)	6	1.5659	1.0000	−0.935 364
O(II)	6	1.5751	1.0000	−0.939 037
O(III)	6	1.5631	1.0000	−0.926 292
O(IV)	6	1.5629	1.0000	−0.926 323
Br(I)	2	3.3445	13.4793	−0.751 375

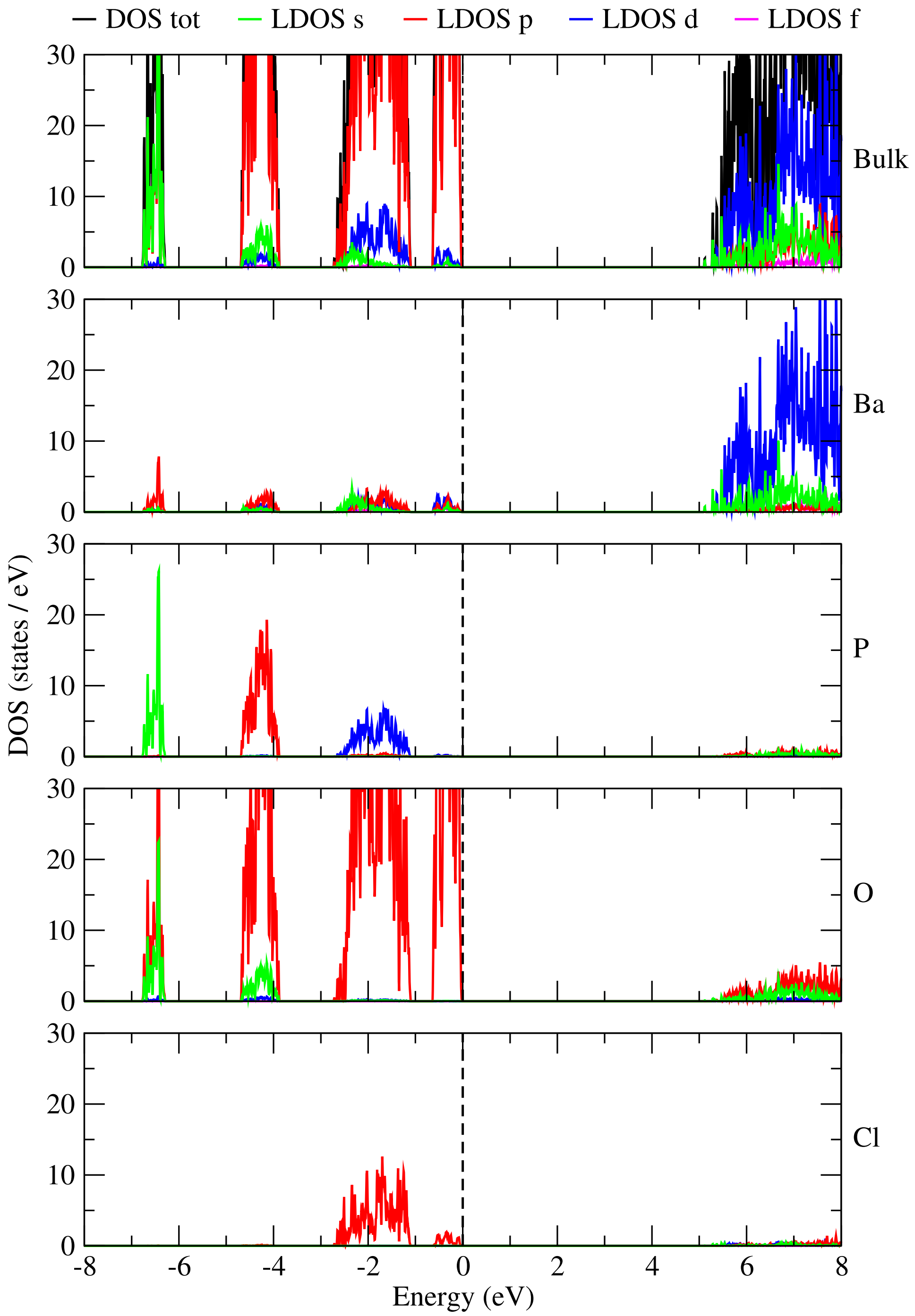

Figure S-83: Local density of states for the $Ba_{10}(PO_4)_6Cl_2$ bulk phase.

Table S-86: Calculated properties for the $Ba_{10}(PO_4)_6Cl_2$ bulk phase. Number of non-equivalent species, N; average distance for nearest neighbors, d_{NN}; effective coordination number, ECN; and net atomic charge, Q.

Non-equivalent species	N	d_{NN} (Å)	ECN (NNN)	Q (e^-)
Ba(I)	2	2.7485	9.1198	1.471 465
Ba(II)	2	2.7473	9.0929	1.471 657
Ba(III)	6	2.6045	6.9879	1.495 807
P(I)	6	1.5635	3.9994	1.507 554
O(I)	6	1.5644	1.0000	−0.933 921
O(II)	6	1.5715	1.0000	−0.936 933
O(III)	6	1.5636	1.0000	−0.929 467
O(IV)	6	1.5635	1.0000	−0.929 130
Cl(I)	2	3.2568	13.0645	−0.764 853

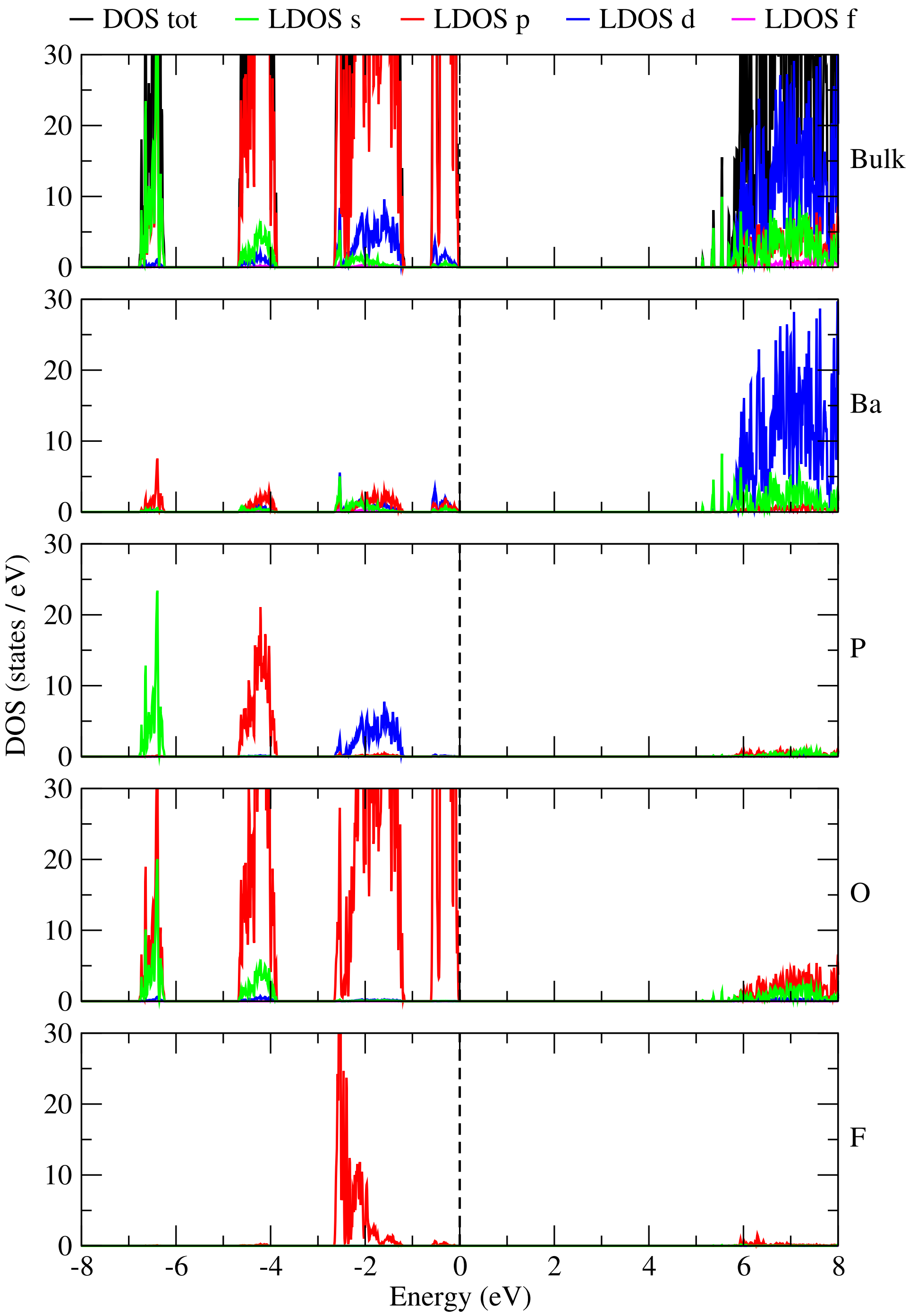

Figure S-84: Local density of states for the $Ba_{10}(PO_4)_6F_2$ bulk phase.

Table S-87: Calculated properties for the $Ba_{10}(PO_4)_6F_2$ bulk phase. Number of non-equivalent species, N; average distance for nearest neighbors, d_{NN}; effective coordination number, ECN; and net atomic charge, Q.

Non-equivalent species	N	d_{NN} (Å)	ECN (NNN)	Q (e^-)
Ba(I)	2	2.7413	8.8810	1.483 058
Ba(II)	2	2.7301	8.6117	1.484 128
Ba(III)	6	2.5718	7.0370	1.510 313
P(I)	6	1.5649	3.9999	1.500 874
O(I)	6	1.5650	1.0000	−0.943 854
O(II)	6	1.5677	1.0000	−0.941 209
O(III)	6	1.5655	1.0000	−0.935 237
O(IV)	6	1.5649	1.0000	−0.935 804
F(I)	2	2.5718	3.1484	−0.732 438

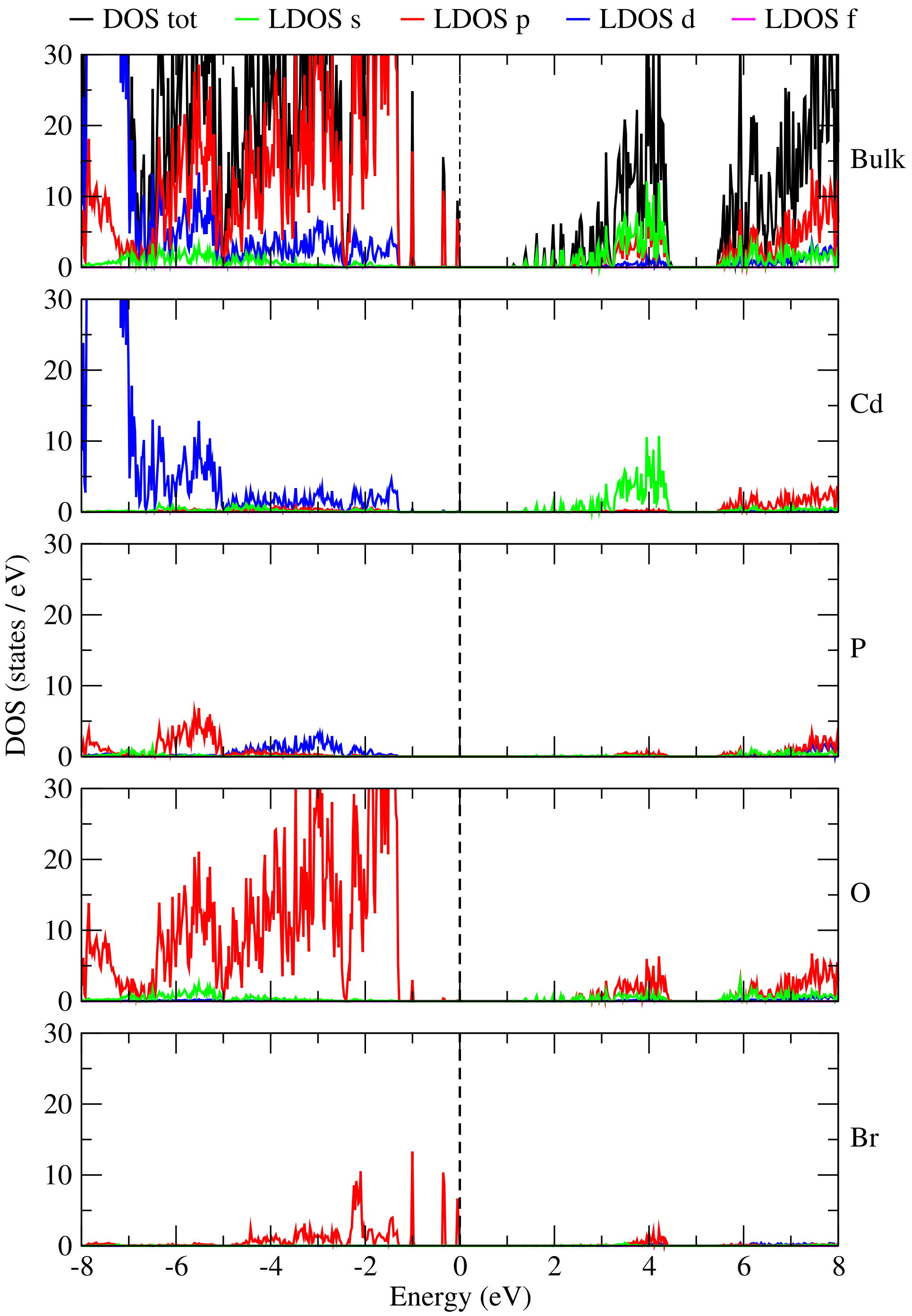

Figure S-85: Local density of states for the $Cd_{10}(PO_4)_6Br_2$ bulk phase.

Table S-88: Calculated properties for the $Cd_{10}(PO_4)_6Br_2$ bulk phase. Number of non-equivalent species, N; average distance for nearest neighbors, d_{NN}; effective coordination number, ECN; and net atomic charge, Q.

Non-equivalent species	N	d_{NN} (Å)	ECN (NNN)	Q (e^-)
Cd(I)	2	2.3665	6.7142	1.183 215
Cd(II)	2	2.3543	6.4190	1.182 584
Cd(III)	6	2.2544	5.2653	1.159 966
P(I)	6	1.5524	3.9831	1.512 527
O(I)	6	1.5524	1.0001	−0.809 064
O(II)	6	1.5982	1.0020	−0.842 909
O(III)	6	1.5597	1.0005	−0.832 054
O(IV)	6	1.5601	1.0011	−0.827 902
Br(I)	2	2.6962	7.8406	−0.447 495

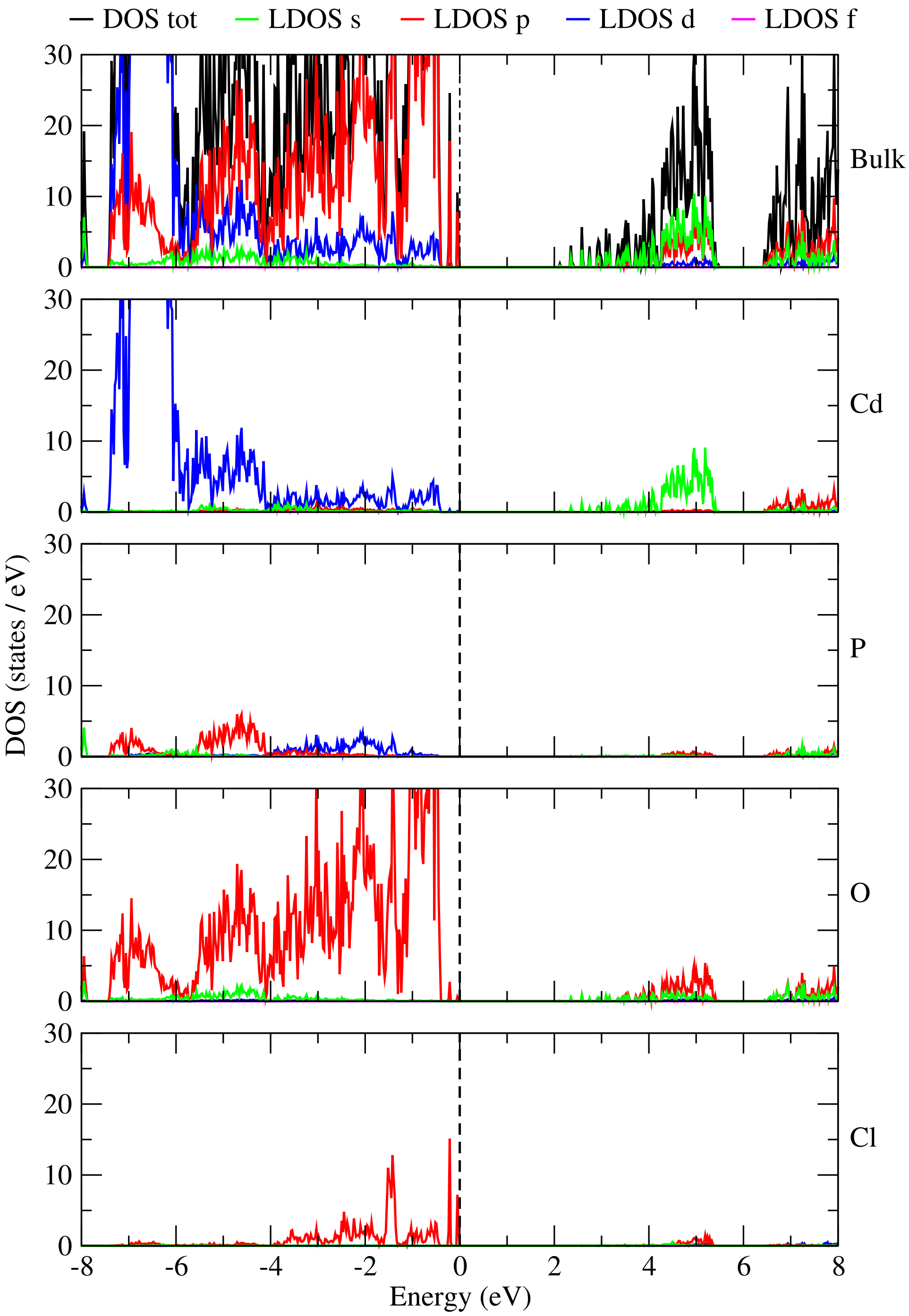

Figure S-86: Local density of states for the $Cd_{10}(PO_4)_6Cl_2$ bulk phase.

Table S-89: Calculated properties for the $Cd_{10}(PO_4)_6Cl_2$ bulk phase. Number of non-equivalent species, N; average distance for nearest neighbors, d_{NN}; effective coordination number, ECN; and net atomic charge, Q.

Non-equivalent species	N	d_{NN} (Å)	ECN (NNN)	Q (e^-)
Cd(I)	2	2.3522	6.5814	1.181 027
Cd(II)	2	2.3476	6.4983	1.181 589
Cd(III)	6	2.2547	5.5193	1.188 794
P(I)	6	1.5508	3.9860	1.513 761
O(I)	6	1.5508	1.0001	−0.808 175
O(II)	6	1.5935	1.0009	−0.846 112
O(III)	6	1.5600	1.0010	−0.834 094
O(IV)	6	1.5601	1.0011	−0.833 524
Cl(I)	2	2.6054	4.9801	−0.504 570

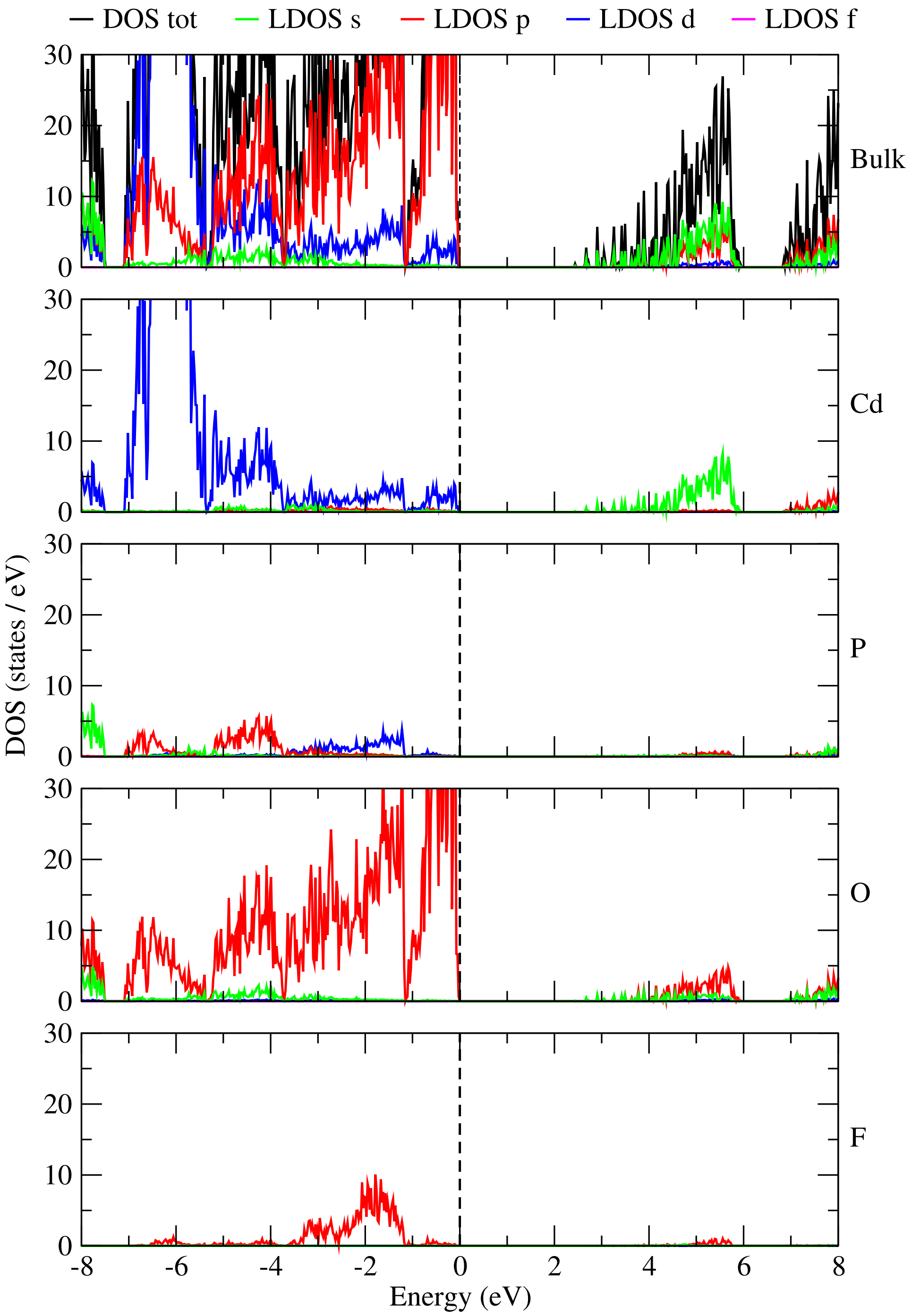

Figure S-87: Local density of states for the $Cd_{10}(PO_4)_6F_2$ bulk phase.

Table S-90: Calculated properties for the $Cd_{10}(PO_4)_6F_2$ bulk phase. Number of non-equivalent species, N; average distance for nearest neighbors, d_{NN}; effective coordination number, ECN; and net atomic charge, Q.

Non-equivalent species	N	d_{NN} (Å)	ECN (NNN)	Q (e^-)
Cd(I)	2	2.3367	6.5945	1.181 196
Cd(II)	2	2.3327	6.5255	1.181 113
Cd(III)	6	2.2409	5.8747	1.241 793
P(I)	6	1.5493	3.9931	1.503 448
O(I)	6	1.5493	1.0002	−0.803 095
O(II)	6	1.5810	1.0001	−0.840 730
O(III)	6	1.5620	1.0016	−0.842 278
O(IV)	6	1.5620	1.0016	−0.842 347
F(I)	2	2.3526	3.1522	−0.612 681

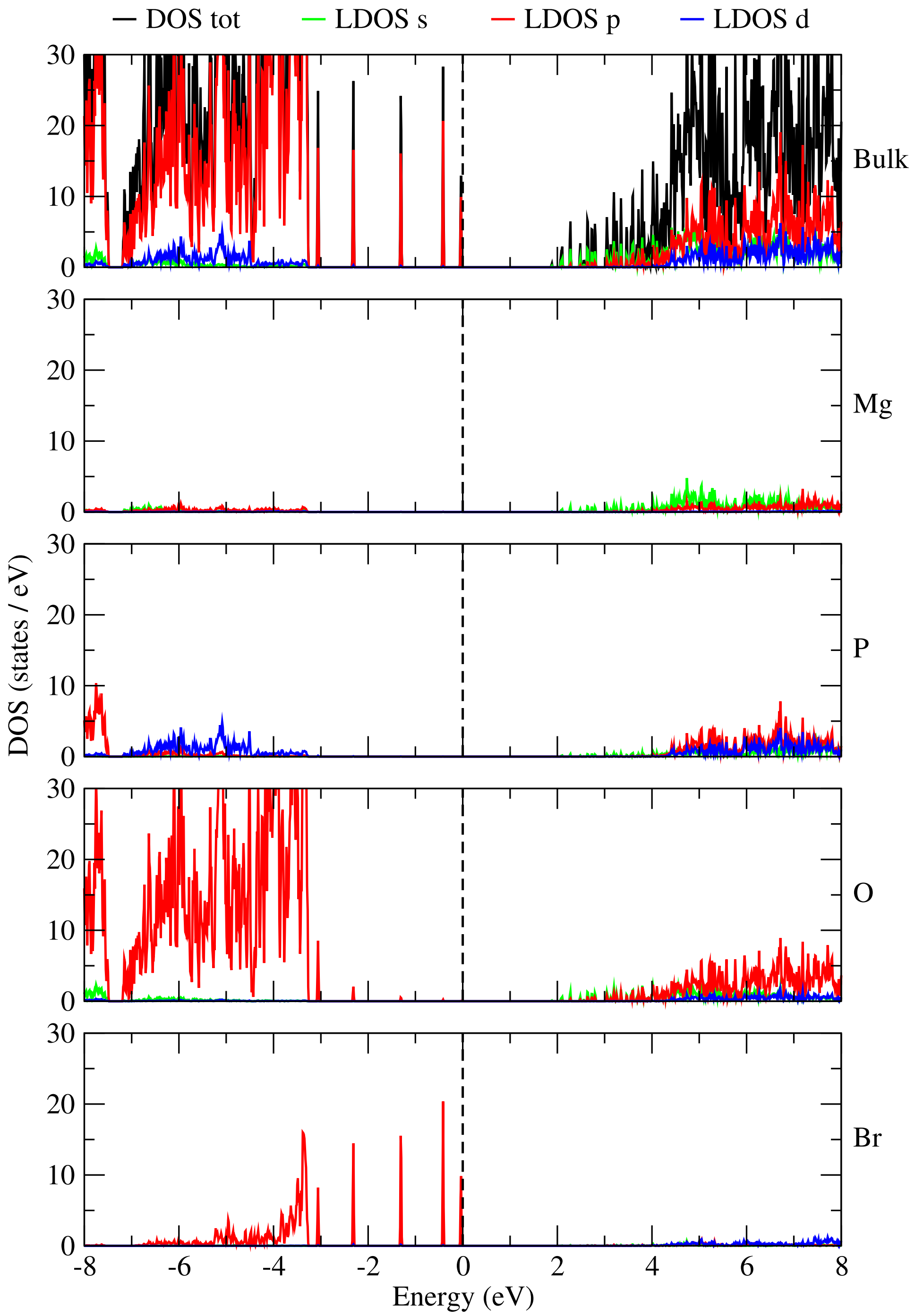

Figure S-88: Local density of states for the $Mg_{10}(PO_4)_6Br_2$ bulk phase.

Table S-91: Calculated properties for the $Mg_{10}(PO_4)_6Br_2$ bulk phase. Number of non-equivalent species, N; average distance for nearest neighbors, d_{NN}; effective coordination number, ECN; and net atomic charge, Q.

Non-equivalent species	N	d_{NN} (Å)	ECN (NNN)	Q (e^-)
Mg(I)	2	2.1471	6.1482	1.505 308
Mg(II)	2	2.1469	6.1421	1.505 122
Mg(III)	6	2.0832	5.0429	1.458 662
P(I)	6	1.5421	3.9775	1.581 507
O(I)	6	1.5421	1.0133	−0.927 540
O(II)	6	1.5973	1.0750	−1.023 829
O(III)	6	1.5566	1.0284	−0.938 251
O(IV)	6	1.5566	1.0294	−0.937 487
Br(I)	2	2.5893	9.4738	−0.649 617

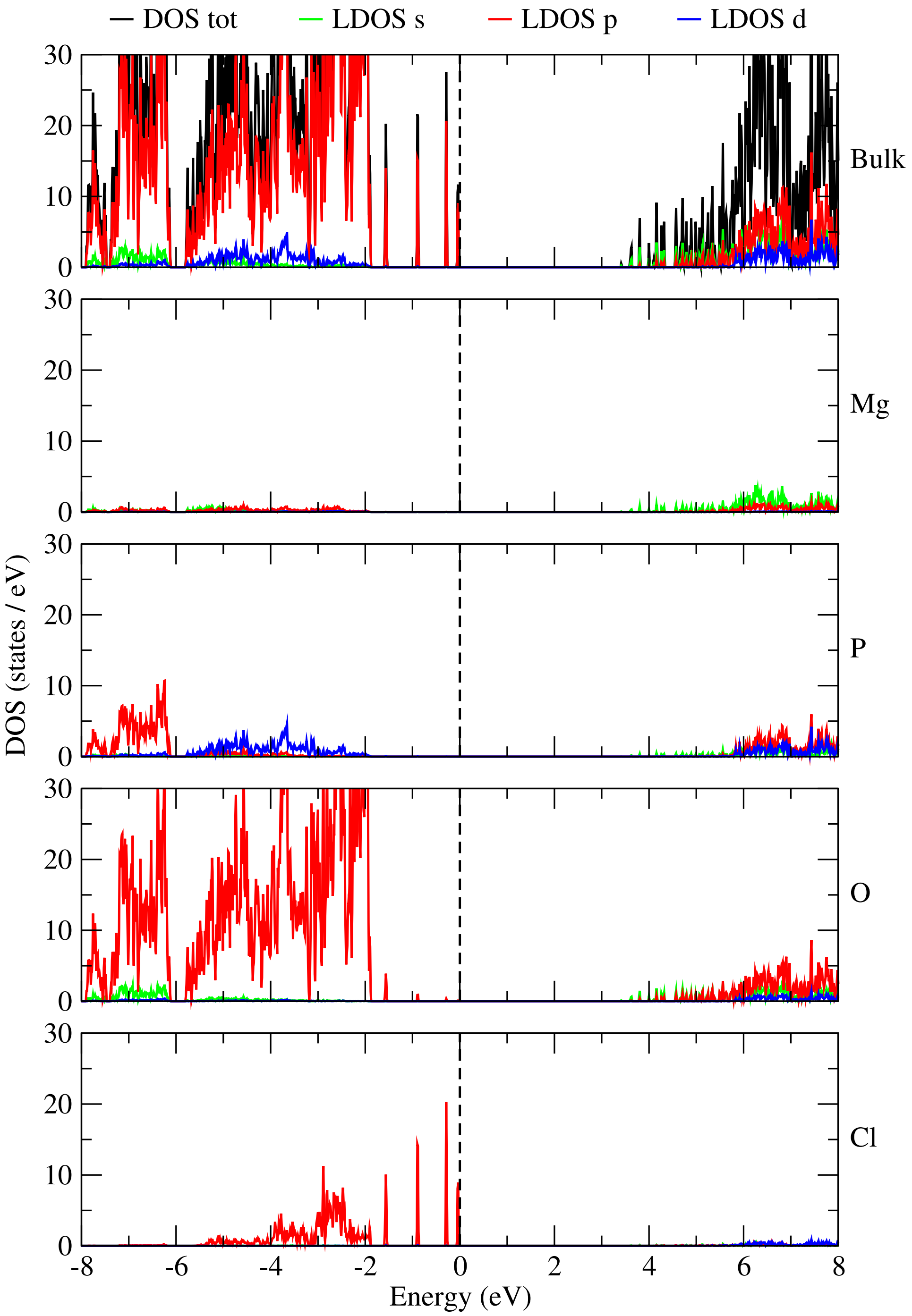

Figure S-89: Local density of states for the $Mg_{10}(PO_4)_6Cl_2$ bulk phase.

Table S-92: Calculated properties for the $Mg_{10}(PO_4)_6Cl_2$ bulk phase. Number of non-equivalent species, N; average distance for nearest neighbors, d_{NN}; effective coordination number, ECN; and net atomic charge, Q.

Non-equivalent species	N	d_{NN} (Å)	ECN (NNN)	Q (e^-)
Mg(I)	2	2.1306	6.1168	1.504 081
Mg(II)	2	2.1297	6.1107	1.503 798
Mg(III)	6	2.0679	5.3364	1.472 446
P(I)	6	1.5380	3.9794	1.586 225
O(I)	6	1.5380	1.0166	−0.925 405
O(II)	6	1.5925	1.0420	−1.018 413
O(III)	6	1.5579	1.0396	−0.946 683
O(IV)	6	1.5578	1.0399	−0.946 412
Cl(I)	2	2.5025	7.3540	−0.673 152

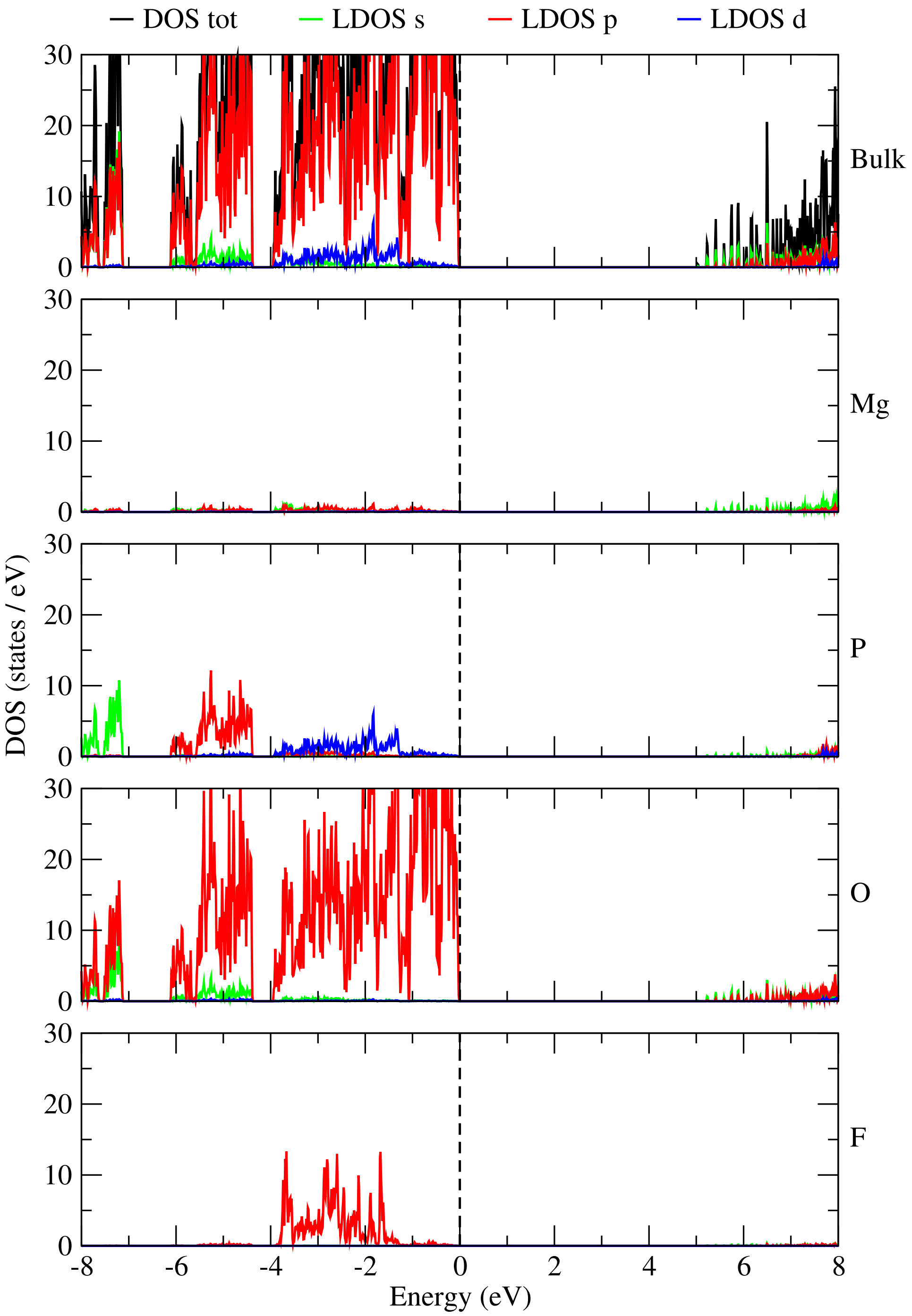

Figure S-90: Local density of states for the $Mg_{10}(PO_4)_6F_2$ bulk phase.

Table S-93: Calculated properties for the $Mg_{10}(PO_4)_6F_2$ bulk phase. Number of non-equivalent species, N; average distance for nearest neighbors, d_{NN}; effective coordination number, ECN; and net atomic charge, Q.

Non-equivalent species	N	d_{NN} (Å)	ECN (NNN)	Q (e^-)
Mg(I)	2	2.1052	6.0145	1.496 477
Mg(II)	2	2.1052	6.0145	1.496 477
Mg(III)	6	2.0310	5.3774	1.509 673
P(I)	6	1.5309	3.9855	1.602 776
O(I)	6	1.5309	1.0222	−0.920 929
O(II)	6	1.5730	1.0190	−0.982 300
O(III)	6	1.5647	1.0931	−0.982 005
O(IV)	6	1.5647	1.0931	−0.982 005
F(I)	2	2.1812	3.0858	−0.728 583

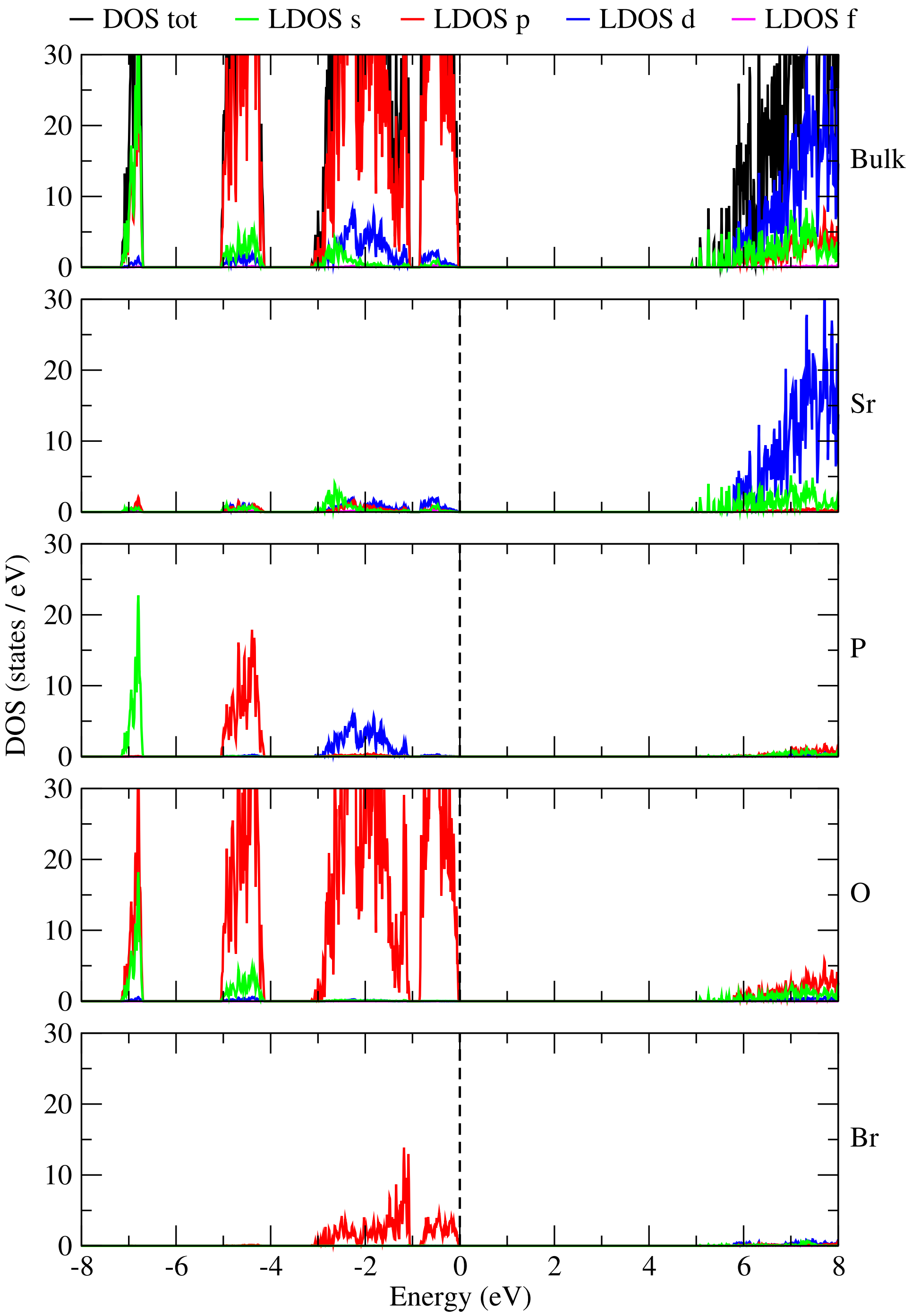

Figure S-91: Local density of states for the $Sr_{10}(PO_4)_6Br_2$ bulk phase.

Table S-94: Calculated properties for the $Sr_{10}(PO_4)_6Br_2$ bulk phase. Number of non-equivalent species, N; average distance for nearest neighbors, d_{NN}; effective coordination number, ECN; and net atomic charge, Q.

Non-equivalent species	N	d_{NN} (Å)	ECN (NNN)	Q (e^-)
Sr(I)	2	2.5982	8.7586	1.477 929
Sr(II)	2	2.5902	8.5752	1.478 445
Sr(III)	6	2.4266	5.6110	1.502 250
P(I)	6	1.5607	3.9986	1.521 590
O(I)	6	1.5619	1.0000	−0.923 350
O(II)	6	1.5731	1.0000	−0.960 260
O(III)	6	1.5607	1.0000	−0.936 735
O(IV)	6	1.5613	1.0000	−0.938 308
Br(I)	2	3.1888	13.5801	−0.751 938

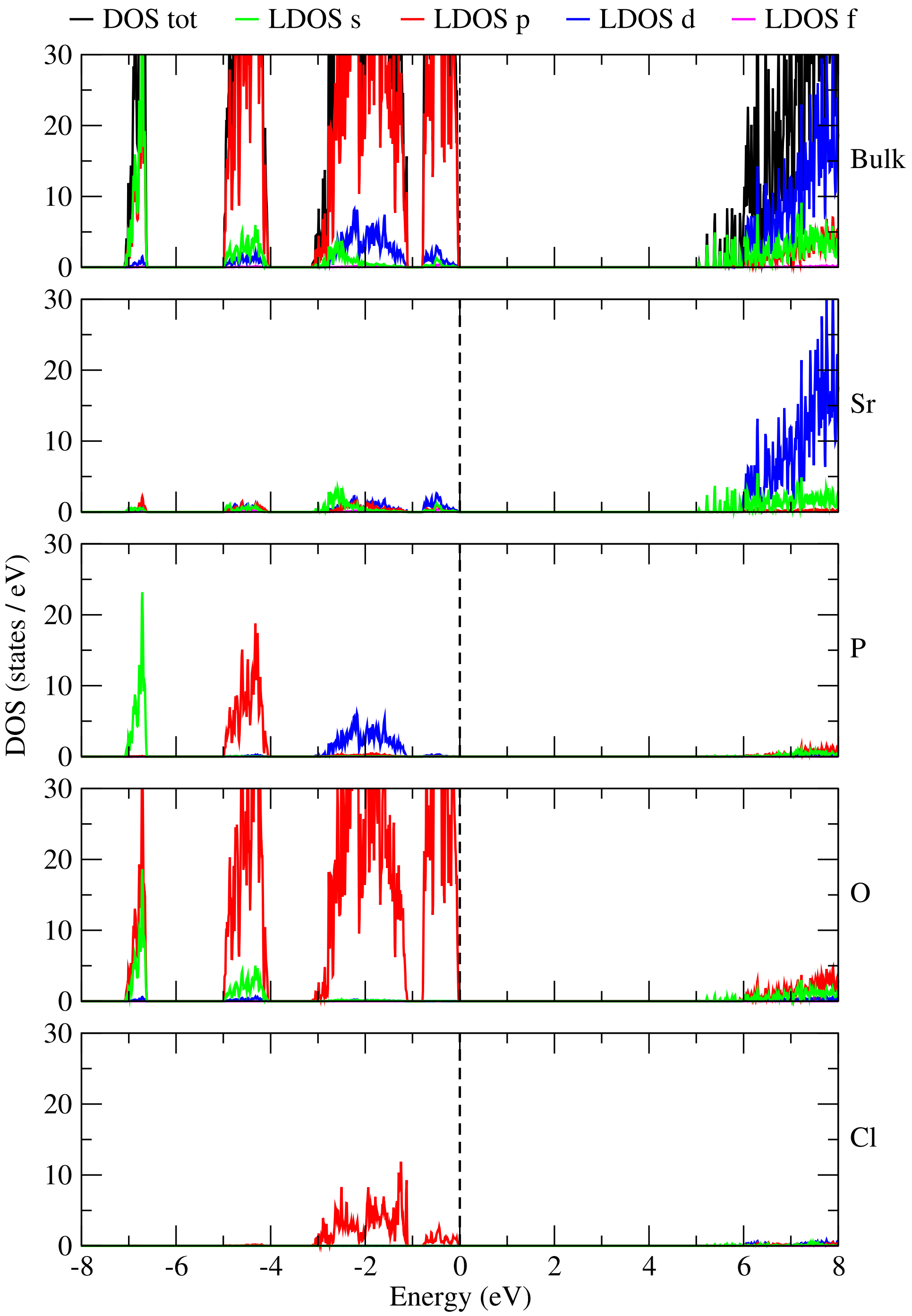

Figure S-92: Local density of states for the $Sr_{10}(PO_4)_6Cl_2$ bulk phase.

Table S-95: Calculated properties for the $Sr_{10}(PO_4)_6Cl_2$ bulk phase. Number of non-equivalent species, N; average distance for nearest neighbors, d_{NN}; effective coordination number, ECN; and net atomic charge, Q.

Non-equivalent species	N	d_{NN} (Å)	ECN (NNN)	Q (e^-)
Sr(I)	2	2.5979	8.7284	1.479 872
Sr(II)	2	2.5888	8.5461	1.480 516
Sr(III)	6	2.4478	6.1440	1.509 383
P(I)	6	1.5601	3.9991	1.516 536
O(I)	6	1.5614	1.0000	−0.923 763
O(II)	6	1.5699	1.0000	−0.955 510
O(III)	6	1.5601	1.0000	−0.939 374
O(IV)	6	1.5603	1.0000	−0.940 285
Cl(I)	2	3.1065	13.2202	−0.761 347

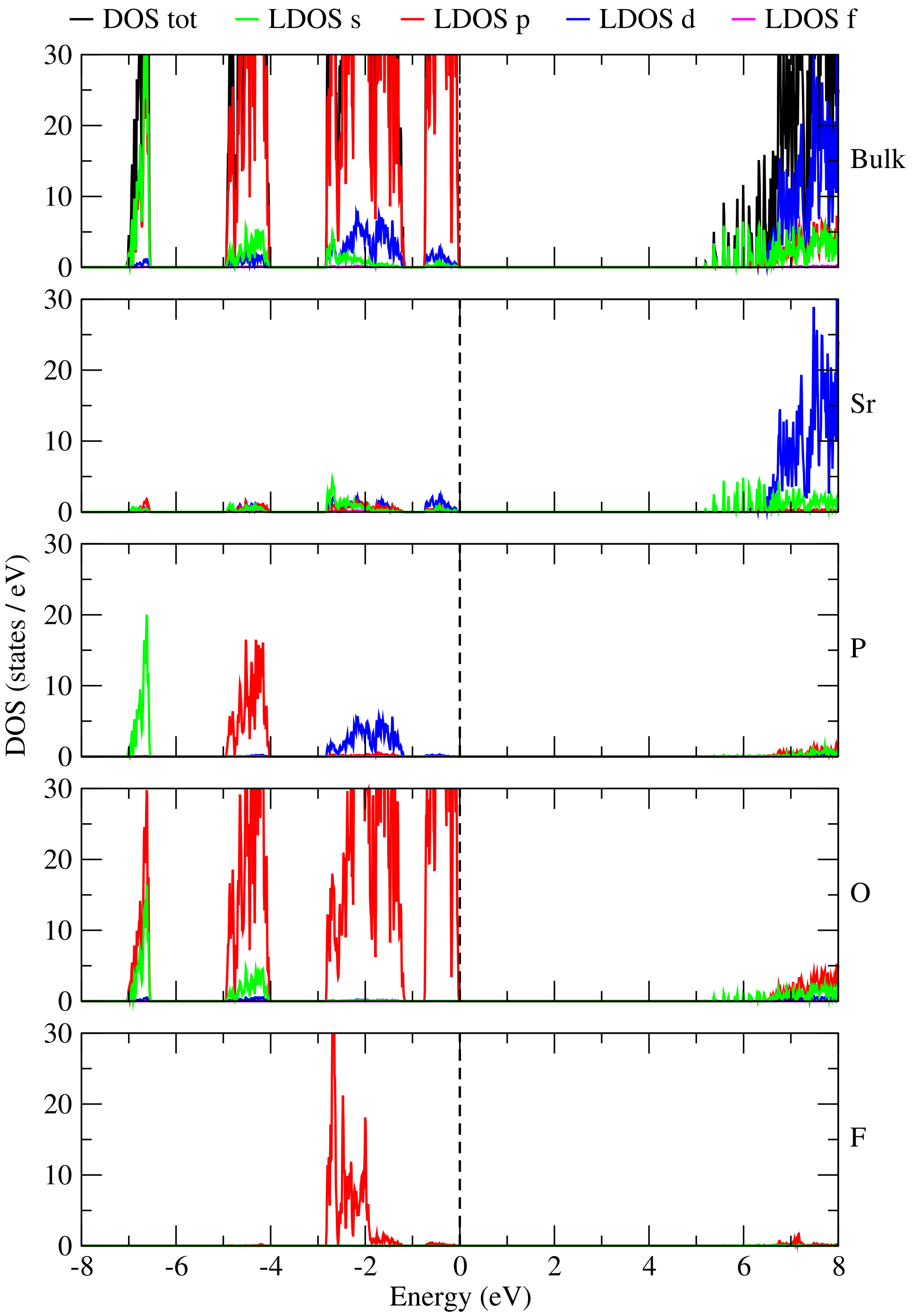

Figure S-93: Local density of states for the $Sr_{10}(PO_4)_6F_2$ bulk phase.

Table S-96: Calculated properties for the $Sr_{10}(PO_4)_6F_2$ bulk phase. Number of non-equivalent species, N; average distance for nearest neighbors, d_{NN}; effective coordination number, ECN; and net atomic charge, Q.

Non-equivalent species	N	d_{NN} (Å)	ECN (NNN)	Q (e^-)
Sr(I)	2	2.5952	8.5953	1.490 790
Sr(II)	2	2.5790	8.2209	1.492 024
Sr(III)	6	2.4350	6.8359	1.522 049
P(I)	6	1.5610	3.9998	1.500 531
O(I)	6	1.5628	1.0000	−0.935 761
O(II)	6	1.5655	1.0000	−0.954 524
O(III)	6	1.5616	1.0000	−0.939 159
O(IV)	6	1.5610	1.0000	−0.939 703
F(I)	2	2.4350	3.1130	−0.743 116

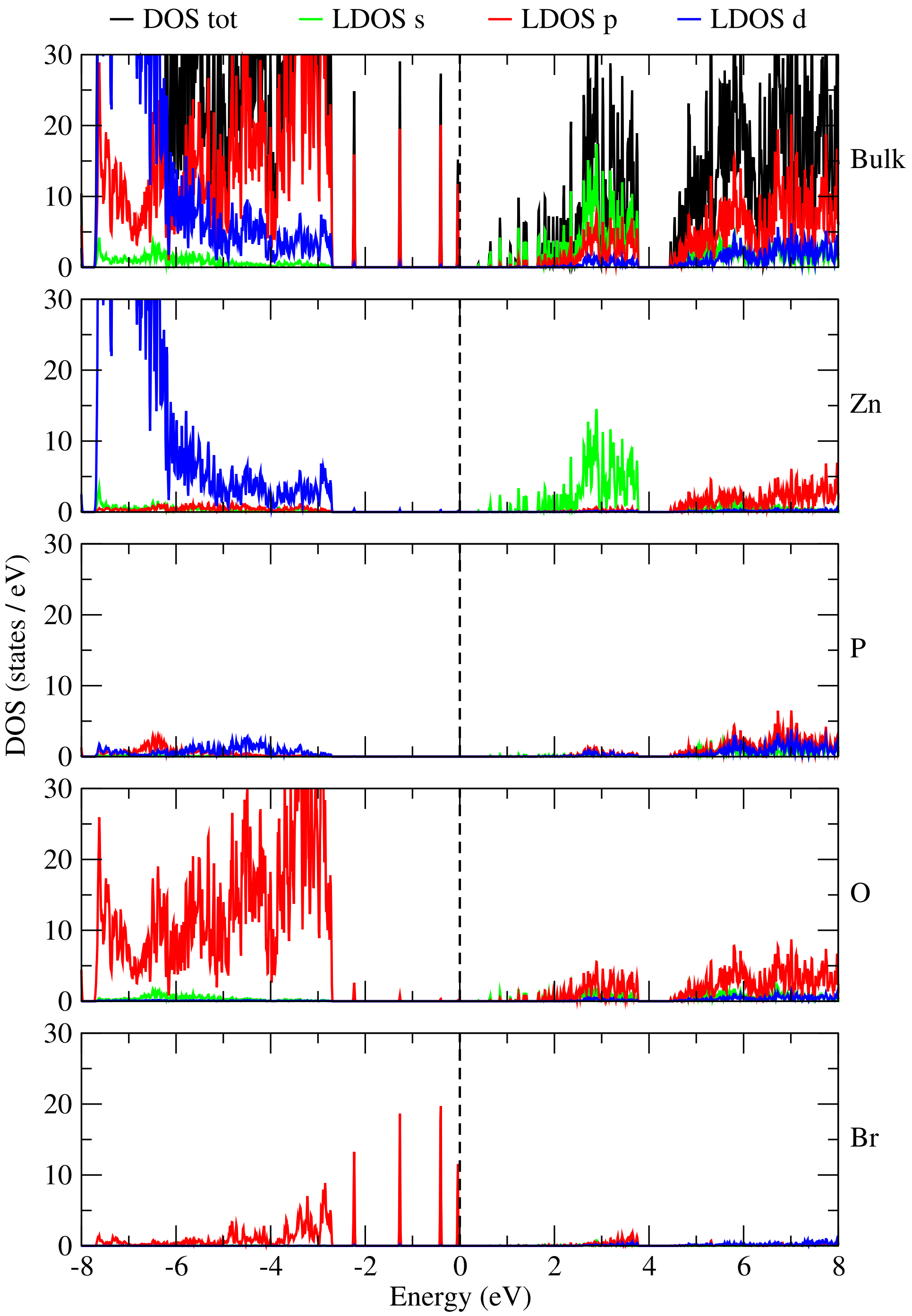

Figure S-94: Local density of states for the $Zn_{10}(PO_4)_6Br_2$ bulk phase.

Table S-97: Calculated properties for the $Zn_{10}(PO_4)_6Br_2$ bulk phase. Number of non-equivalent species, N; average distance for nearest neighbors, d_{NN}; effective coordination number, ECN; and net atomic charge, Q.

Non-equivalent species	N	d_{NN} (Å)	ECN (NNN)	Q (e^-)
Zn(I)	2	2.1479	6.0690	1.126 000
Zn(II)	2	2.1473	6.0632	1.125 785
Zn(III)	6	2.0453	4.8451	1.079 426
P(I)	6	1.5449	3.9720	1.530 900
O(I)	6	1.5449	1.0142	−0.784 019
O(II)	6	1.6051	1.0378	−0.834 869
O(III)	6	1.5573	1.0500	−0.803 823
O(IV)	6	1.5575	1.0533	−0.802 896
Br(I)	2	2.5529	8.4300	−0.405 940

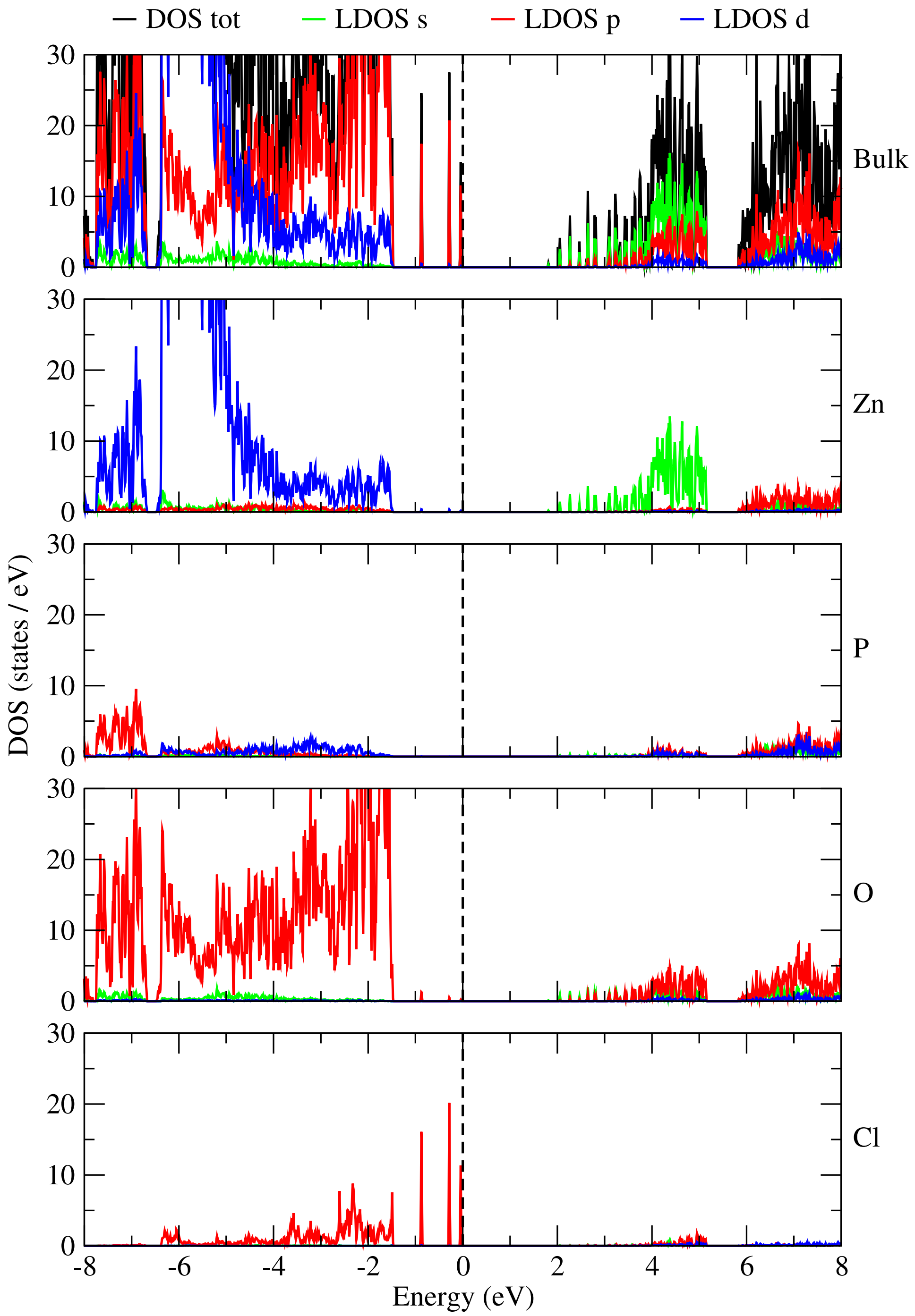

Figure S-95: Local density of states for the $Zn_{10}(PO_4)_6Cl_2$ bulk phase.

Table S-98: Calculated properties for the $Zn_{10}(PO_4)_6Cl_2$ bulk phase. Number of non-equivalent species, N; average distance for nearest neighbors, d_{NN}; effective coordination number, ECN; and net atomic charge, Q.

Non-equivalent species	N	d_{NN} (Å)	ECN (NNN)	Q (e^-)
Zn(I)	2	2.1385	6.0596	1.125 090
Zn(II)	2	2.1363	6.0489	1.125 116
Zn(III)	6	2.0292	5.1086	1.104 820
P(I)	6	1.5419	3.9788	1.536 965
O(I)	6	1.5419	1.0160	−0.783 197
O(II)	6	1.5970	1.0222	−0.838 271
O(III)	6	1.5610	1.0721	−0.807 438
O(IV)	6	1.5607	1.0728	−0.807 190
Cl(I)	2	2.4628	5.8985	−0.467 272

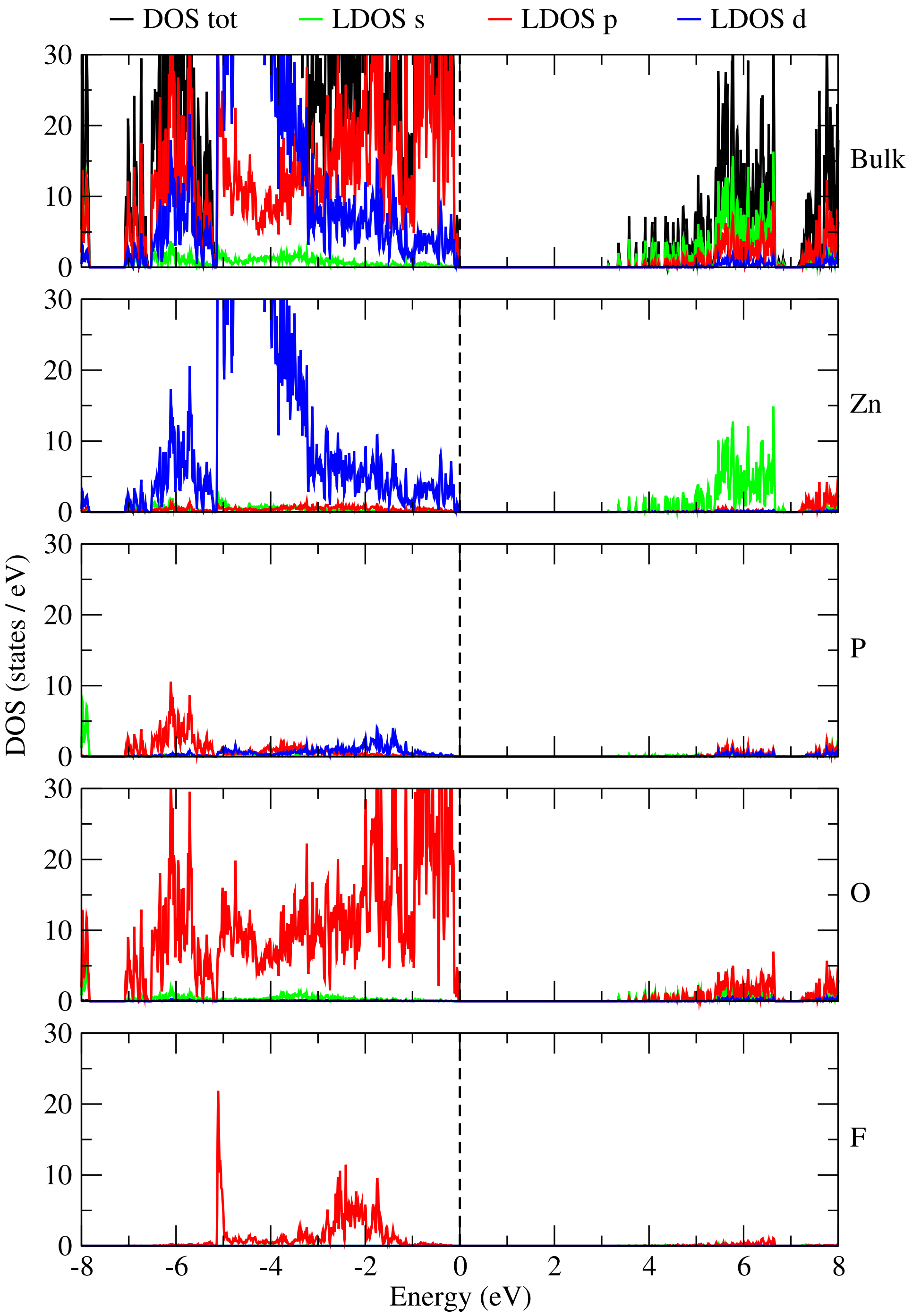

Figure S-96: Local density of states for the $Zn_{10}(PO_4)_6F_2$ bulk phase.

Table S-99: Calculated properties for the $Zn_{10}(PO_4)_6F_2$ bulk phase. Number of non-equivalent species, N; average distance for nearest neighbors, d_{NN}; effective coordination number, ECN; and net atomic charge, Q.

Non-equivalent species	N	d_{NN} (Å)	ECN (NNN)	Q (e^-)
Zn(I)	2	2.1342	6.0341	1.126 523
Zn(II)	2	2.1328	6.0320	1.126 501
Zn(III)	6	1.9846	4.7571	1.151 421
P(I)	6	1.5385	3.9891	1.559 972
O(I)	6	1.5385	1.0157	−0.784 381
O(II)	6	1.5722	1.0209	−0.830 275
O(III)	6	1.5705	1.1720	−0.827 780
O(IV)	6	1.5705	1.1688	−0.827 971
F(I)	2	2.1609	3.0751	−0.575 986

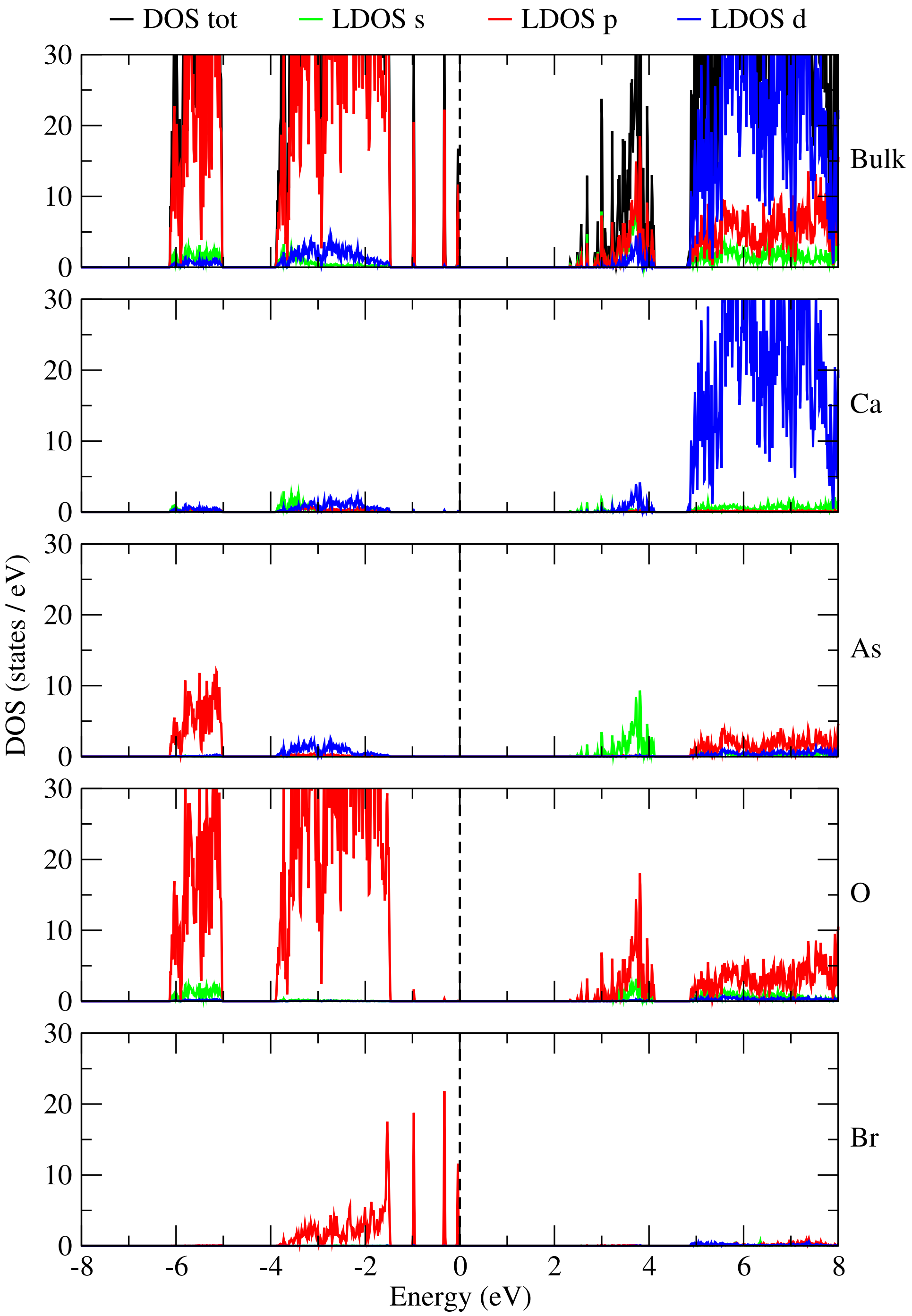

Figure S-97: Local density of states for the $Ca_{10}(AsO_4)_6Br_2$ bulk phase.

Table S-100: Calculated properties for the $Ca_{10}(AsO_4)_6Br_2$ bulk phase. Number of non-equivalent species, N; average distance for nearest neighbors, d_{NN}; effective coordination number, ECN; and net atomic charge, Q.

Non-equivalent species	N	d_{NN} (Å)	ECN (NNN)	Q (e^-)
Ca(I)	2	2.4118	6.8995	1.435 792
Ca(II)	2	2.3917	6.4166	1.436 997
Ca(III)	6	2.3144	5.1469	1.422 472
As(I)	6	1.7052	3.9920	1.685 734
O(I)	6	1.7052	1.0081	−0.930 332
O(II)	6	1.7427	1.0477	−1.012 293
O(III)	6	1.7211	1.0102	−0.948 612
O(IV)	6	1.7191	1.0164	−0.946 621
Br(I)	2	2.8714	10.5853	−0.683 831

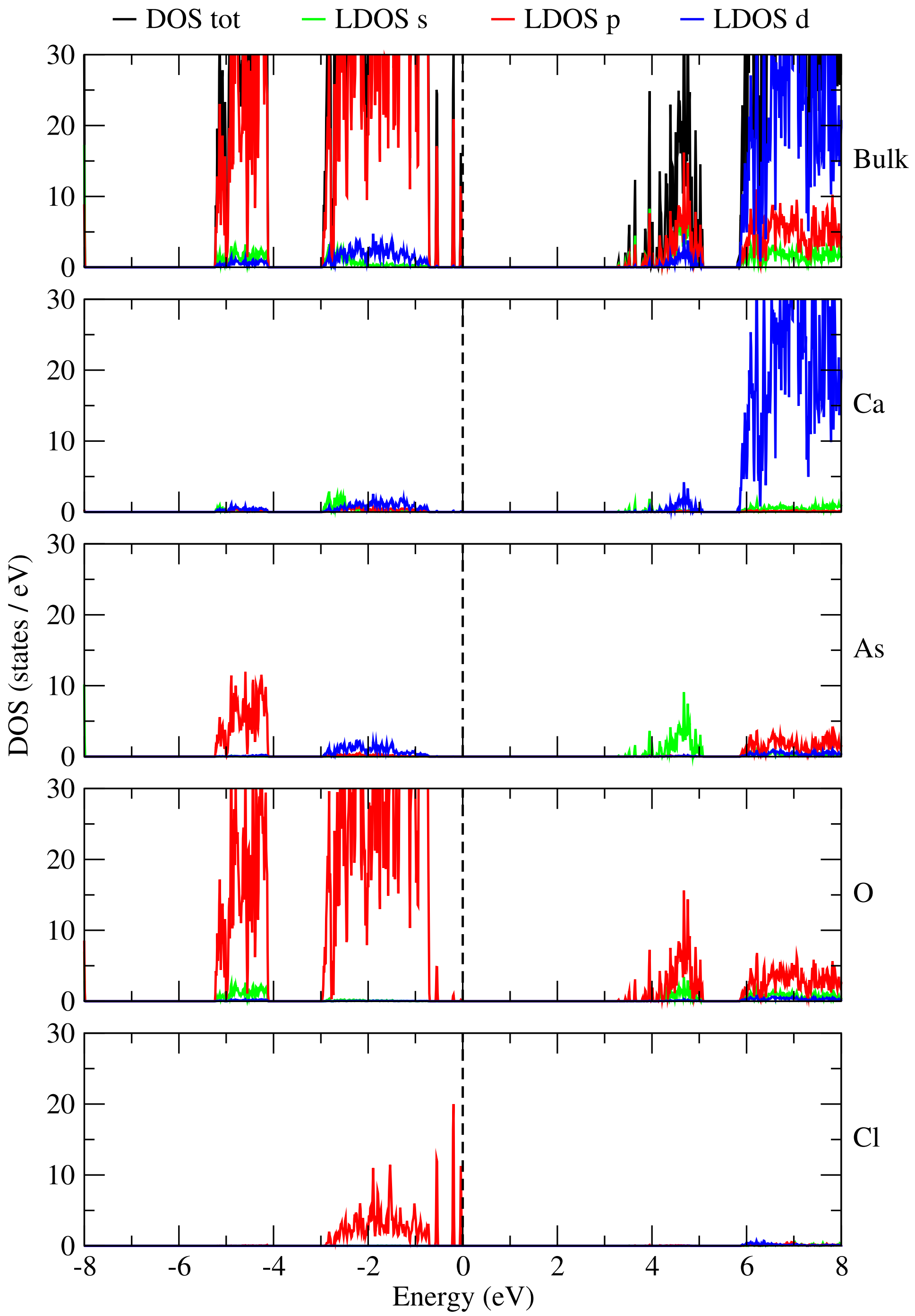

Figure S-98: Local density of states for the $Ca_{10}(AsO_4)_6Cl_2$ bulk phase.

Table S-101: Calculated properties for the $Ca_{10}(AsO_4)_6Cl_2$ bulk phase. Number of non-equivalent species, N; average distance for nearest neighbors, d_{NN}; effective coordination number, ECN; and net atomic charge, Q.

Non-equivalent species	N	d_{NN} (Å)	ECN (NNN)	Q (e^-)
Ca(I)	2	2.3997	6.7928	1.432 780
Ca(II)	2	2.3863	6.4721	1.434 413
Ca(III)	6	2.3334	5.4582	1.430 986
As(I)	6	1.7034	3.9927	1.686 513
O(I)	6	1.7034	1.0090	−0.929 432
O(II)	6	1.7396	1.0359	−1.011 801
O(III)	6	1.7202	1.0145	−0.950 245
O(IV)	6	1.7202	1.0163	−0.949 982
Cl(I)	2	2.7372	5.7768	−0.695 310

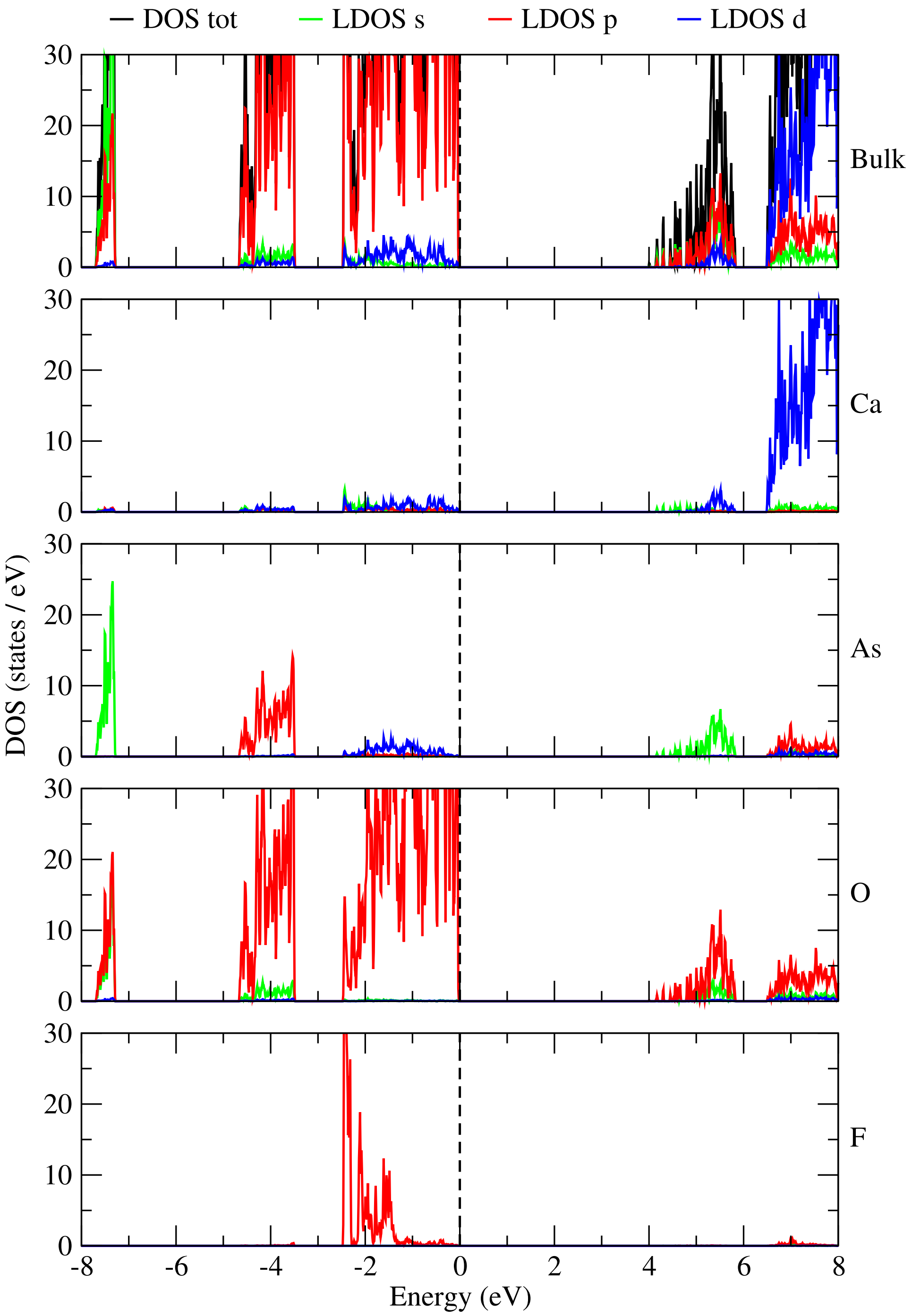

Figure S-99: Local density of states for the $Ca_{10}(AsO_4)_6F_2$ bulk phase.

Table S-102: Calculated properties for the $Ca_{10}(AsO_4)_6F_2$ bulk phase. Number of non-equivalent species, N; average distance for nearest neighbors, d_{NN}; effective coordination number, ECN; and net atomic charge, Q.

Non-equivalent species	N	d_{NN} (Å)	ECN (NNN)	Q (e^-)
Ca(I)	2	2.3749	6.7613	1.417 442
Ca(II)	2	2.3728	6.7099	1.417 903
Ca(III)	6	2.3456	6.0576	1.457 696
As(I)	6	1.7002	3.9953	1.670 174
O(I)	6	1.7002	1.0117	−0.916 632
O(II)	6	1.7282	1.0068	−0.990 048
O(III)	6	1.7188	1.0157	−0.962 361
O(IV)	6	1.7194	1.0165	−0.962 229
F(I)	2	2.3890	3.0585	−0.725 150

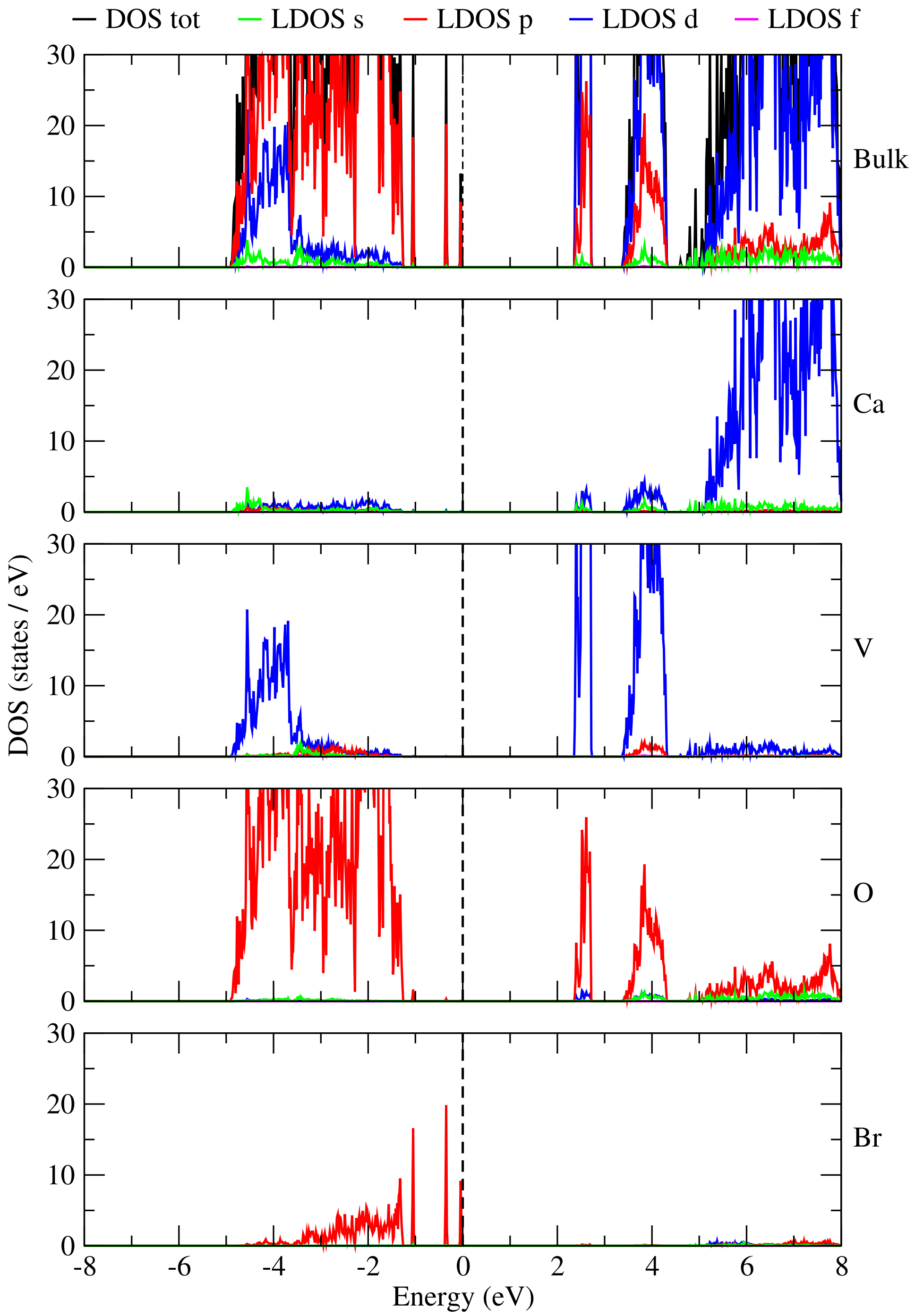

Figure S-100: Local density of states for the $Ca_{10}(VO_4)_6Br_2$ bulk phase.

Table S-103: Calculated properties for the $Ca_{10}(VO_4)_6Br_2$ bulk phase. Number of non-equivalent species, N; average distance for nearest neighbors, d_{NN}; effective coordination number, ECN; and net atomic charge, Q.

Non-equivalent species	N	d_{NN} (Å)	ECN (NNN)	Q (e^-)
Ca(I)	2	2.4046	6.7562	1.487 918
Ca(II)	2	2.3873	6.3912	1.486 758
Ca(III)	6	2.3085	5.0949	1.458 883
V(I)	6	1.7092	3.9873	1.994 031
O(I)	6	1.7092	1.0099	−1.022 551
O(II)	6	1.7527	1.0685	−1.140 127
O(III)	6	1.7179	1.0107	−1.030 007
O(IV)	6	1.7154	1.0152	−1.027 612
Br(I)	2	2.9282	12.1460	−0.672 523

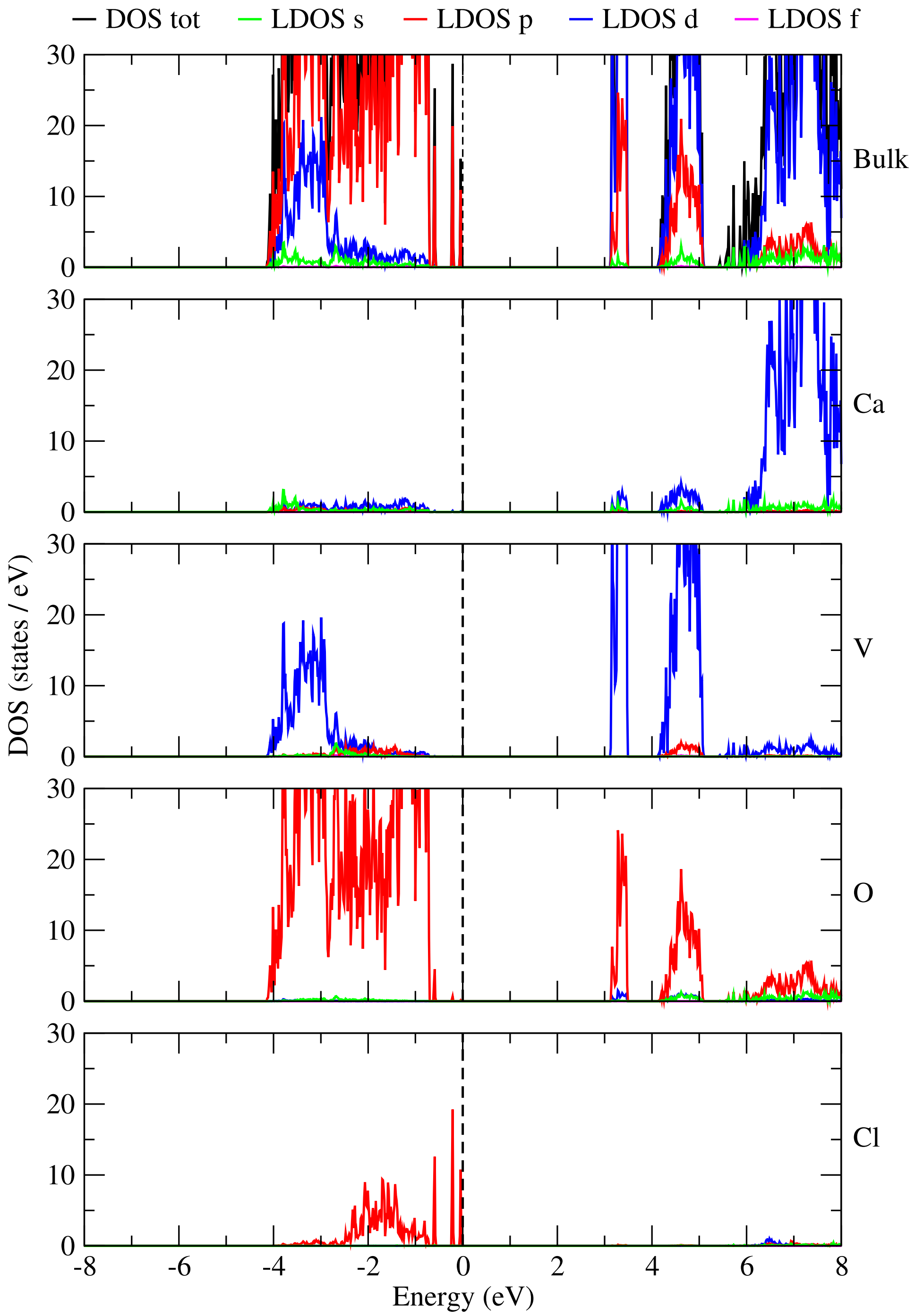

Figure S-101: Local density of states for the $Ca_{10}(VO_4)_6Cl_2$ bulk phase.

Table S-104: Calculated properties for the $Ca_{10}(VO_4)_6Cl_2$ bulk phase. Number of non-equivalent species, N; average distance for nearest neighbors, d_{NN}; effective coordination number, ECN; and net atomic charge, Q.

Non-equivalent species	N	d_{NN} (Å)	ECN (NNN)	Q (e^-)
Ca(I)	2	2.4002	6.6558	1.484 731
Ca(II)	2	2.3899	6.4389	1.485 480
Ca(III)	6	2.3235	5.4523	1.464 771
V(I)	6	1.7093	3.9888	1.993 722
O(I)	6	1.7093	1.0101	−1.024 125
O(II)	6	1.7507	1.0559	−1.141 464
O(III)	6	1.7177	1.0156	−1.028 513
O(IV)	6	1.7163	1.0165	−1.026 037
Cl(I)	2	2.7417	6.1369	−0.685 272

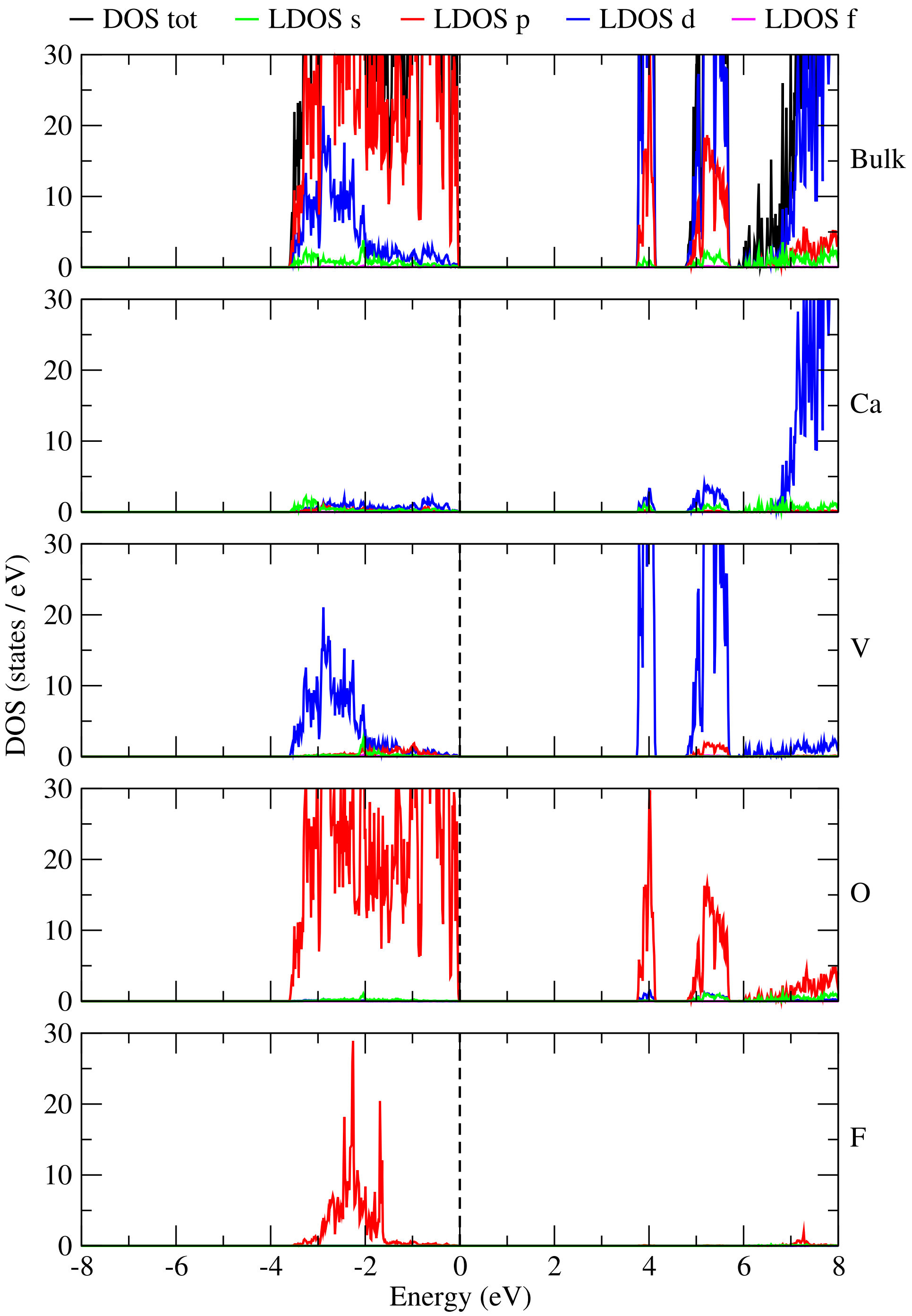

Figure S-102: Local density of states for the $Ca_{10}(VO_4)_6F_2$ bulk phase.

Table S-105: Calculated properties for the $Ca_{10}(VO_4)_6F_2$ bulk phase. Number of non-equivalent species, N; average distance for nearest neighbors, d_{NN}; effective coordination number, ECN; and net atomic charge, Q.

Non-equivalent species	N	d_{NN} (Å)	ECN (NNN)	Q (e^-)
Ca(I)	2	2.3767	6.6401	1.468 255
Ca(II)	2	2.3762	6.6328	1.468 150
Ca(III)	6	2.3369	6.0417	1.490 936
V(I)	6	1.7033	3.9914	1.979 390
O(I)	6	1.7033	1.0121	−1.010 060
O(II)	6	1.7413	1.0124	−1.125 213
O(III)	6	1.7152	1.0174	−1.036 585
O(IV)	6	1.7151	1.0176	−1.036 718
F(I)	2	2.3857	3.0590	−0.721 655

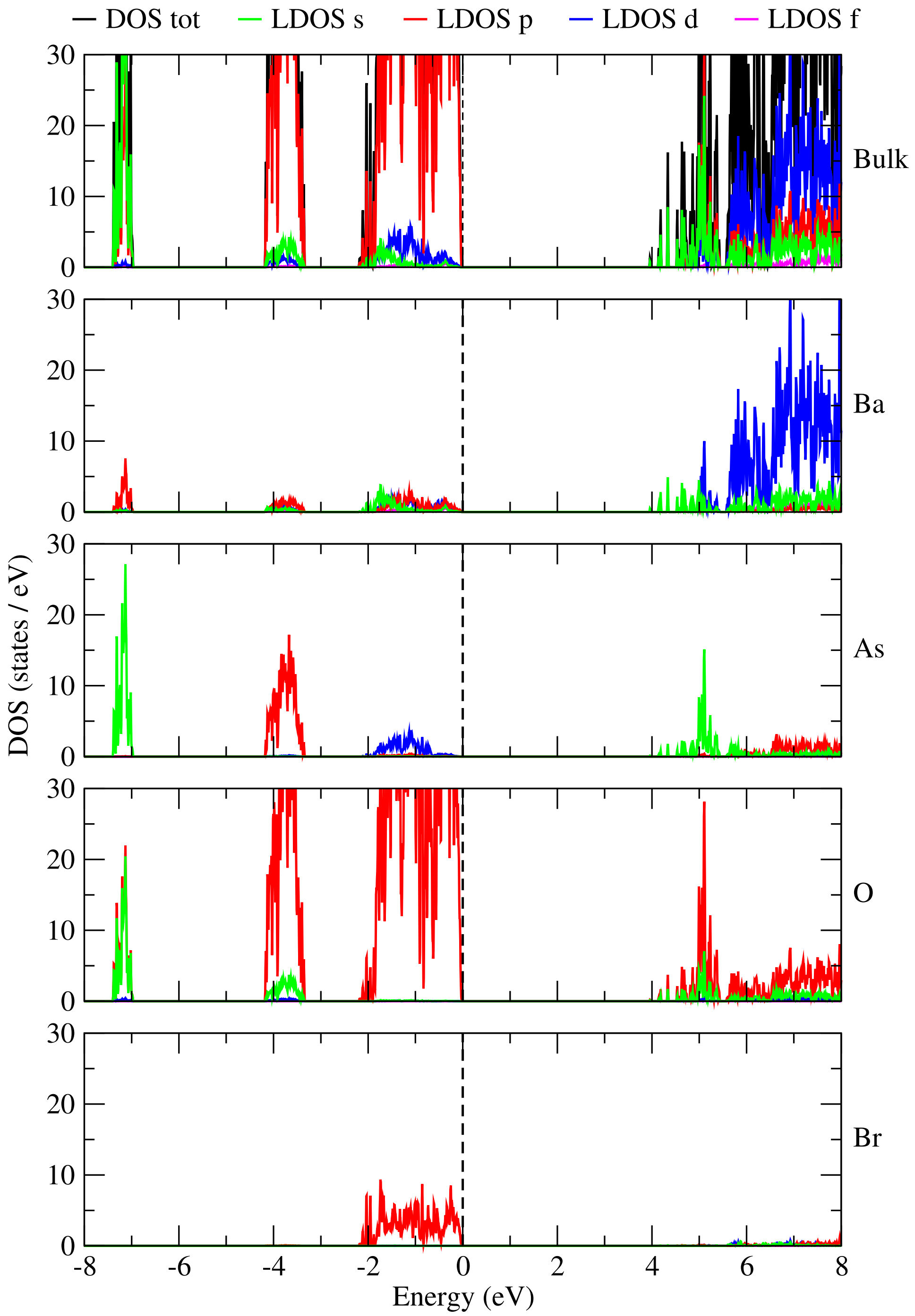

Figure S-103: Local density of states for the $Ba_{10}(AsO_4)_6Br_2$ bulk phase.

Table S-106: Calculated properties for the $Ba_{10}(AsO_4)_6Br_2$ bulk phase. Number of non-equivalent species, N; average distance for nearest neighbors, d_{NN}; effective coordination number, ECN; and net atomic charge, Q.

Non-equivalent species	N	d_{NN} (Å)	ECN (NNN)	Q (e^-)
Ba(I)	2	2.7847	8.8598	1.431 757
Ba(II)	2	2.7734	8.6367	1.432 771
Ba(III)	6	2.5988	5.8113	1.455 225
As(I)	6	1.7210	3.9990	1.656 441
O(I)	6	1.7233	1.0000	−0.946 849
O(II)	6	1.7328	1.0001	−0.972 930
O(III)	6	1.7210	1.0000	−0.949 414
O(IV)	6	1.7218	1.0000	−0.950 411
Br(I)	2	3.3962	13.4299	−0.740 716

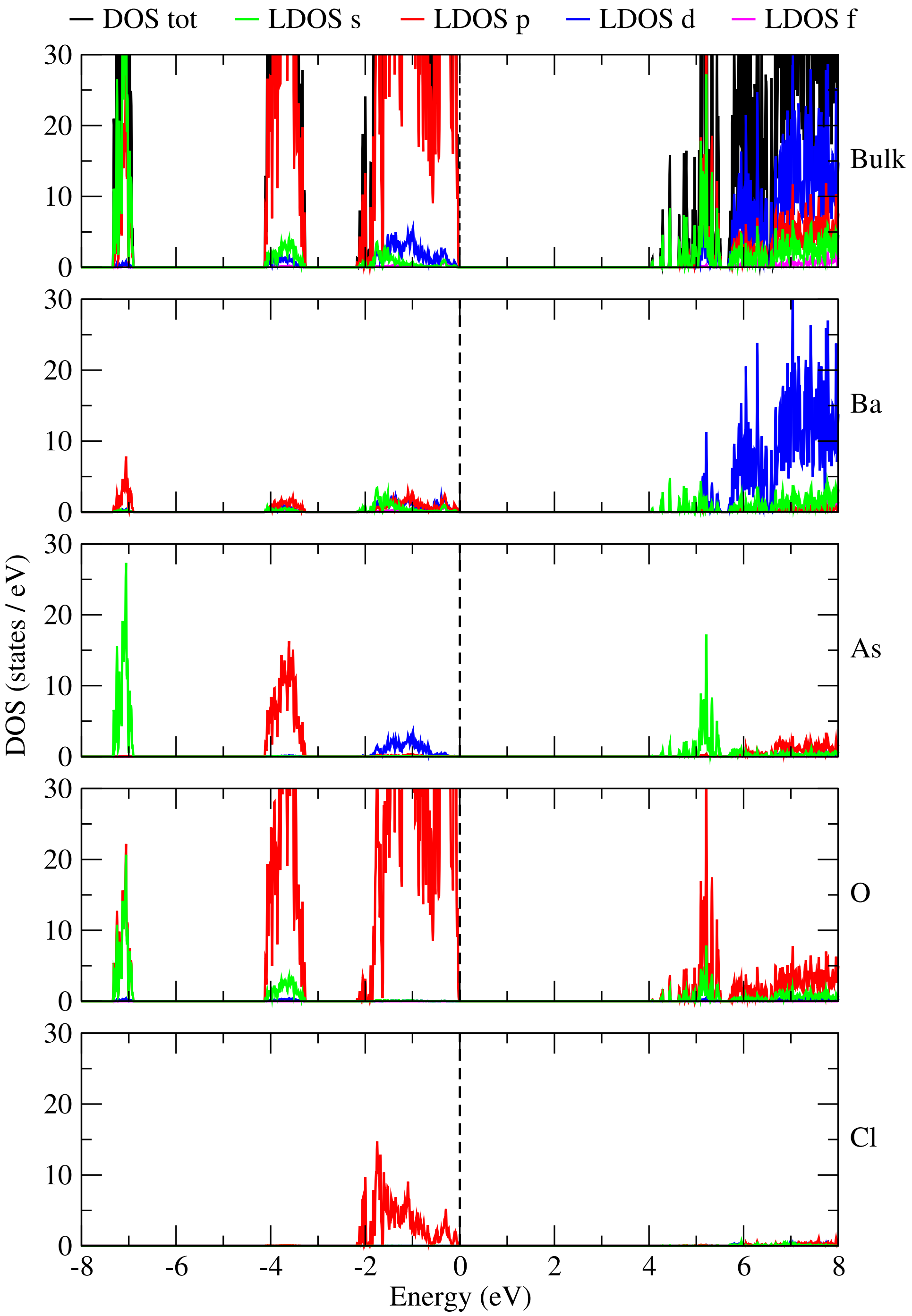

Figure S-104: Local density of states for the $Ba_{10}(AsO_4)_6Cl_2$ bulk phase.

Table S-107: Calculated properties for the $Ba_{10}(AsO_4)_6Cl_2$ bulk phase. Number of non-equivalent species, N; average distance for nearest neighbors, d_{NN}; effective coordination number, ECN; and net atomic charge, Q.

Non-equivalent species	N	d_{NN} (Å)	ECN (NNN)	Q (e^-)
Ba(I)	2	2.7759	8.8474	1.430 273
Ba(II)	2	2.7662	8.6568	1.431 366
Ba(III)	6	2.6239	6.2709	1.463 824
As(I)	6	1.7206	3.9994	1.652 226
O(I)	6	1.7225	1.0000	−0.945 181
O(II)	6	1.7304	1.0001	−0.970 771
O(III)	6	1.7206	1.0000	−0.950 539
O(IV)	6	1.7226	1.0000	−0.952 476
Cl(I)	2	3.3288	13.1298	−0.752 885

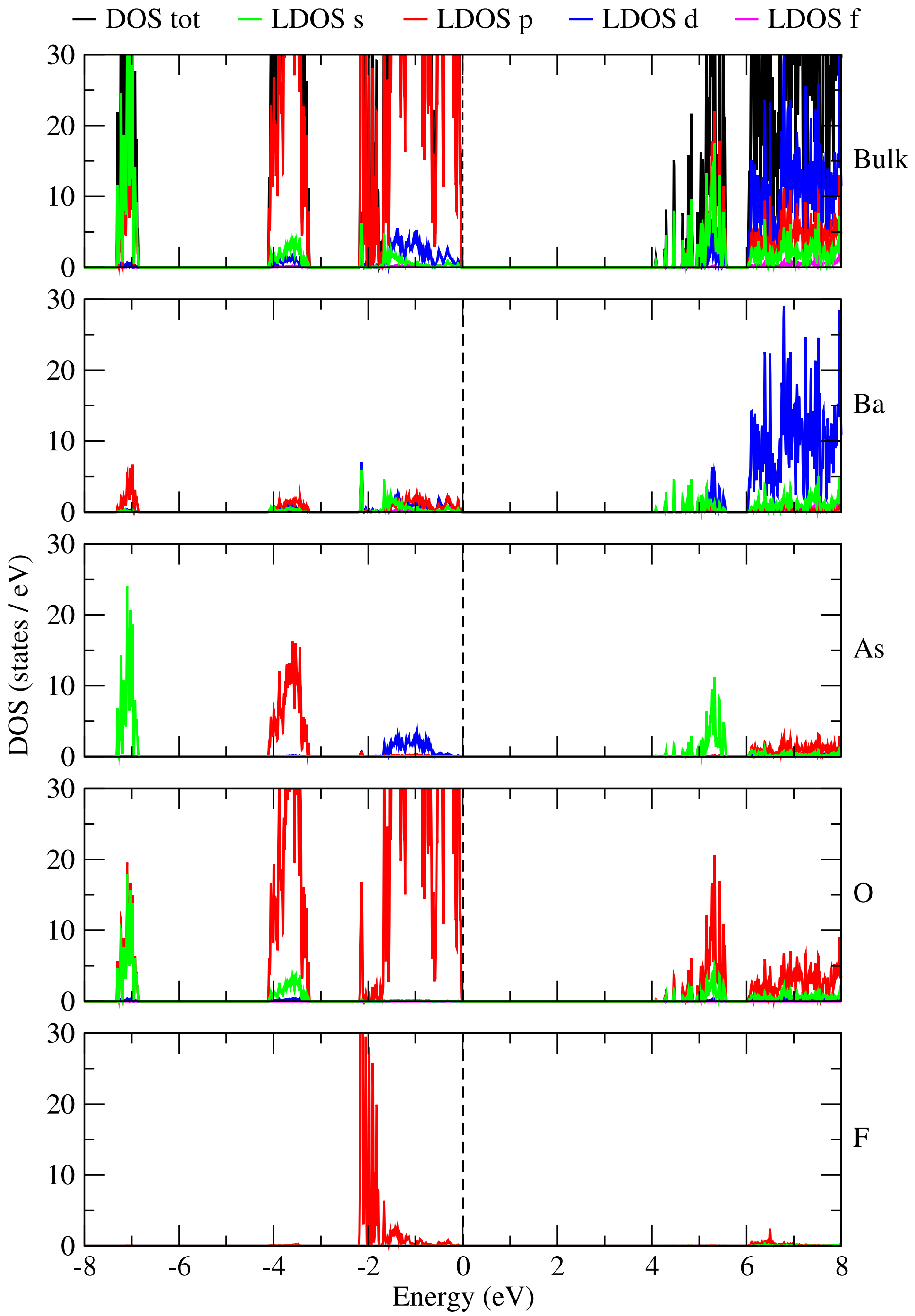

Figure S-105: Local density of states for the $Ba_{10}(AsO_4)_6F_2$ bulk phase.

Table S-108: Calculated properties for the $Ba_{10}(AsO_4)_6F_2$ bulk phase. Number of non-equivalent species, N; average distance for nearest neighbors, d_{NN}; effective coordination number, ECN; and net atomic charge, Q.

Non-equivalent species	N	d_{NN} (Å)	ECN (NNN)	Q (e^-)
Ba(I)	2	2.7656	8.5898	1.438 980
Ba(II)	2	2.7480	8.1971	1.440 318
Ba(III)	6	2.6110	6.7969	1.478 035
As(I)	6	1.7228	3.9999	1.645 036
O(I)	6	1.7229	1.0000	−0.954 185
O(II)	6	1.7270	1.0000	−0.973 894
O(III)	6	1.7228	1.0000	−0.954 962
O(IV)	6	1.7232	1.0000	−0.955 696
F(I)	2	2.6110	3.0903	−0.732 297

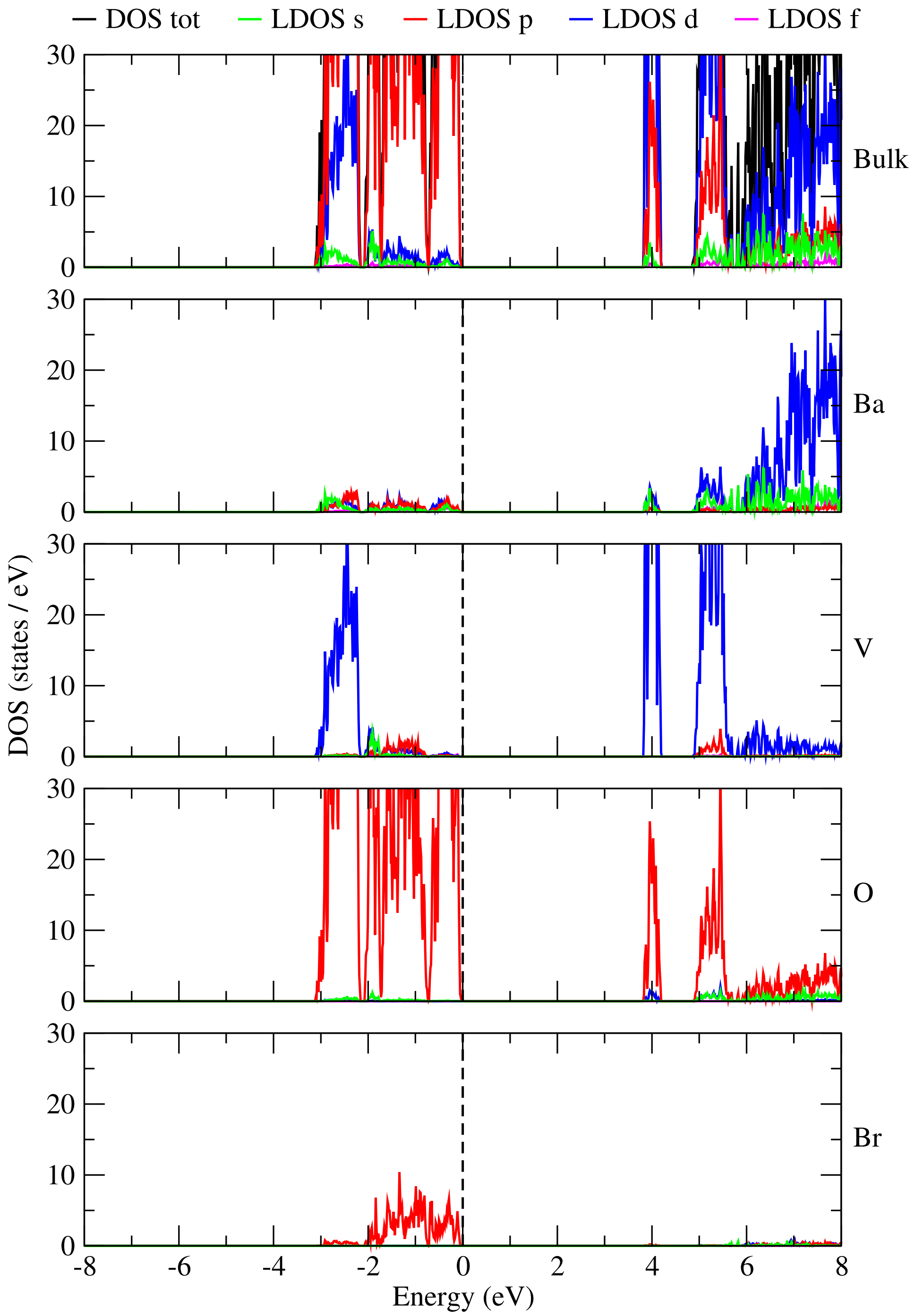

Figure S-106: Local density of states for the $Ba_{10}(VO_4)_6Br_2$ bulk phase.

Table S-109: Calculated properties for the $Ba_{10}(VO_4)_6Br_2$ bulk phase. Number of non-equivalent species, N; average distance for nearest neighbors, d_{NN}; effective coordination number, ECN; and net atomic charge, Q.

Non-equivalent species	N	d_{NN} (Å)	ECN (NNN)	Q (e^-)
Ba(I)	2	2.7646	8.7154	1.495 360
Ba(II)	2	2.7578	8.6010	1.495 520
Ba(III)	6	2.5834	5.8987	1.489 083
V(I)	6	1.7152	3.9964	1.966 034
O(I)	6	1.7324	1.0000	−1.067 320
O(II)	6	1.7346	1.0002	−1.087 187
O(III)	6	1.7152	1.0000	−1.028 523
O(IV)	6	1.7159	1.0000	−1.029 251
Br(I)	2	3.3944	13.4439	−0.719 388

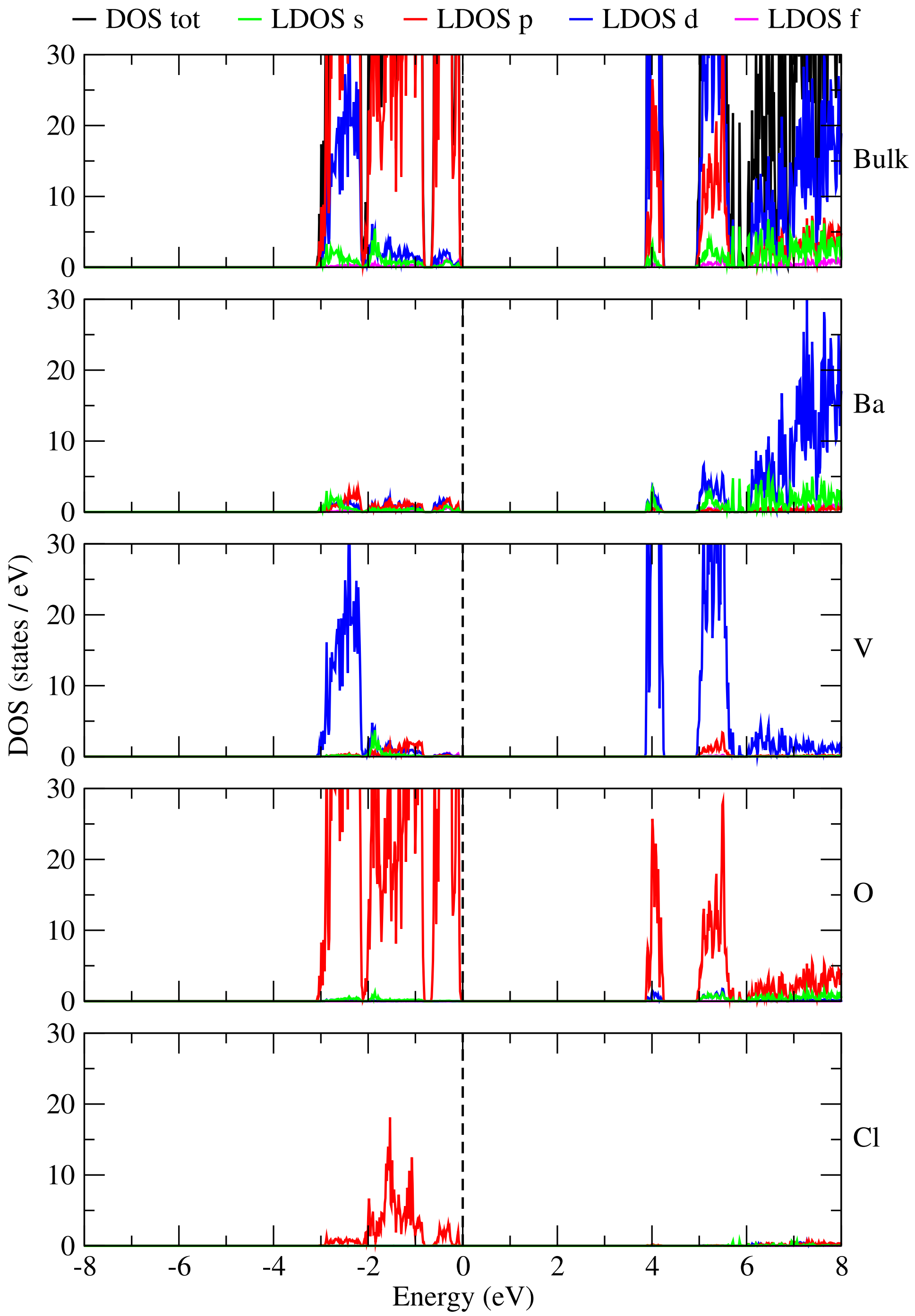

Figure S-107: Local density of states for the $Ba_{10}(VO_4)_6Cl_2$ bulk phase.

Table S-110: Calculated properties for the $Ba_{10}(VO_4)_6Cl_2$ bulk phase. Number of non-equivalent species, N; average distance for nearest neighbors, d_{NN}; effective coordination number, ECN; and net atomic charge, Q.

Non-equivalent species	N	d_{NN} (Å)	ECN (NNN)	Q (e^-)
Ba(I)	2	2.7540	8.6896	1.492 201
Ba(II)	2	2.7458	8.5270	1.493 492
Ba(III)	6	2.6046	6.3128	1.498 191
V(I)	6	1.7150	3.9967	1.963 218
O(I)	6	1.7311	1.0000	−1.065 384
O(II)	6	1.7341	1.0001	−1.086 736
O(III)	6	1.7158	1.0000	−1.030 068
O(IV)	6	1.7150	1.0000	−1.029 491
Cl(I)	2	3.3135	13.1211	−0.734 882

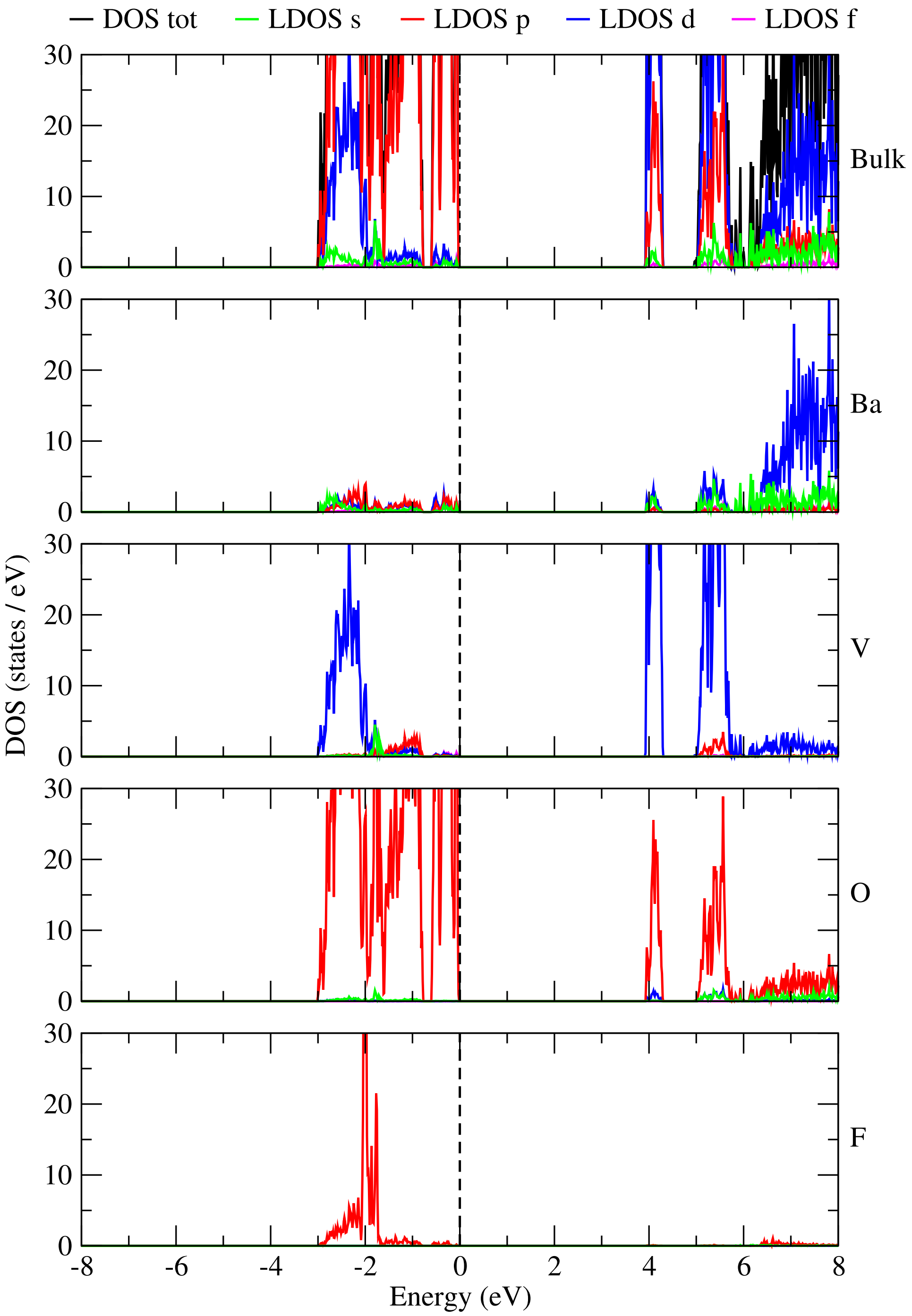

Figure S-108: Local density of states for the $Ba_{10}(VO_4)_6F_2$ bulk phase.

Table S-111: Calculated properties for the $Ba_{10}(VO_4)_6F_2$ bulk phase. Number of non-equivalent species, N; average distance for nearest neighbors, d_{NN}; effective coordination number, ECN; and net atomic charge, Q.

Non-equivalent species	N	d_{NN} (Å)	ECN (NNN)	Q (e^-)
Ba(I)	2	2.7554	8.5396	1.497 646
Ba(II)	2	2.7406	8.2502	1.498 619
Ba(III)	6	2.6106	6.8646	1.514 773
V(I)	6	1.7172	3.9981	1.960 190
O(I)	6	1.7311	1.0000	−1.075 956
O(II)	6	1.7289	1.0000	−1.089 372
O(III)	6	1.7172	1.0000	−1.032 163
O(IV)	6	1.7172	1.0000	−1.032 214
F(I)	2	2.6106	3.1120	−0.732 038

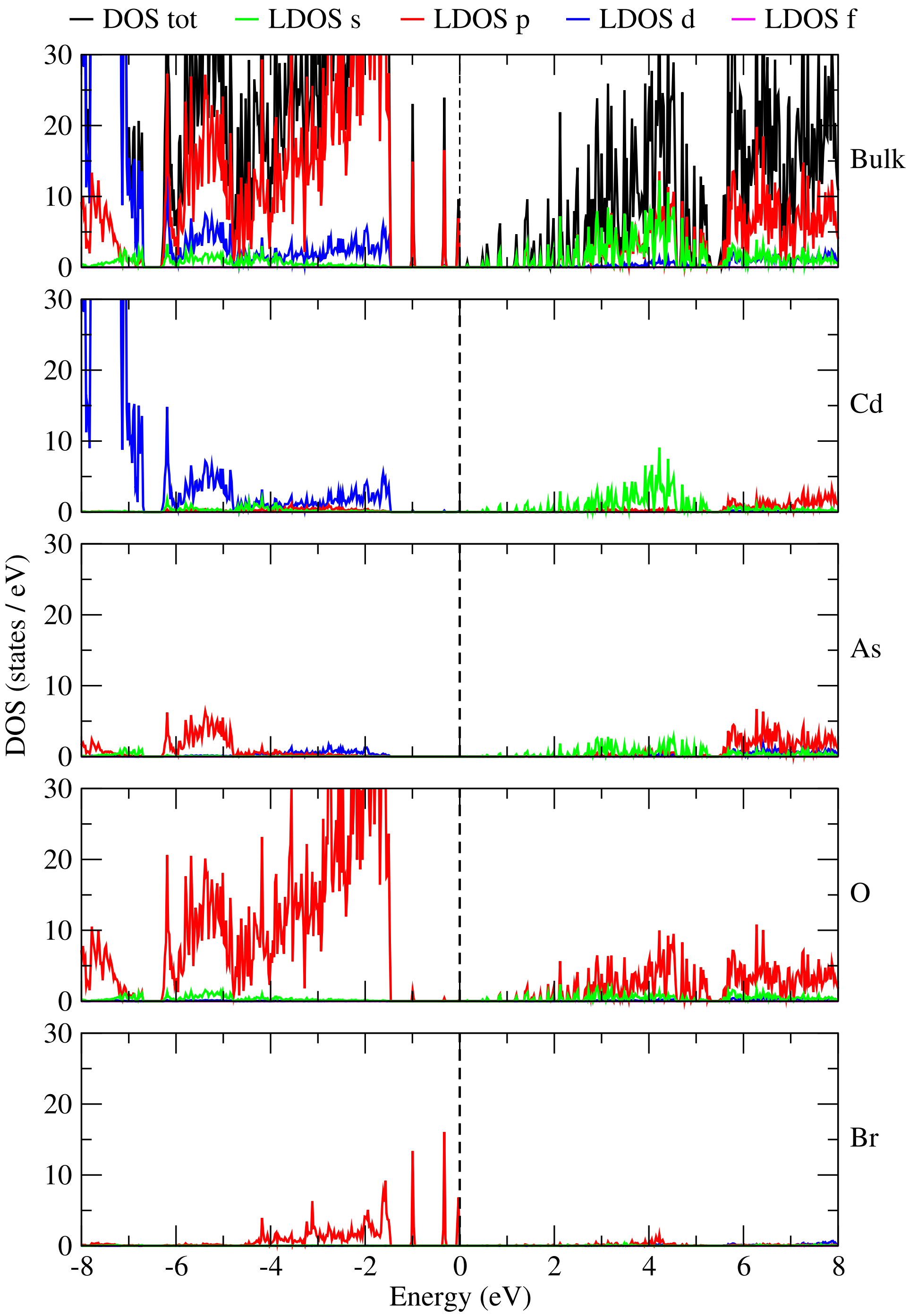

Figure S-109: Local density of states for the $Cd_{10}(AsO_4)_6Br_2$ bulk phase.

Table S-112: Calculated properties for the $Cd_{10}(AsO_4)_6Br_2$ bulk phase. Number of non-equivalent species, N; average distance for nearest neighbors, d_{NN}; effective coordination number, ECN; and net atomic charge, Q.

Non-equivalent species	N	d_{NN} (Å)	ECN (NNN)	Q (e^-)
Cd(I)	2	2.3427	6.2739	1.139 384
Cd(II)	2	2.3382	6.2061	1.138 700
Cd(III)	6	2.2726	5.2636	1.099 545
As(I)	6	1.7042	3.9830	1.658 824
O(I)	6	1.7042	1.0235	−0.817 729
O(II)	6	1.7587	1.0742	−0.872 989
O(III)	6	1.7235	1.0407	−0.840 510
O(IV)	6	1.7240	1.0486	−0.838 301
Br(I)	2	2.7395	6.8131	−0.444 605

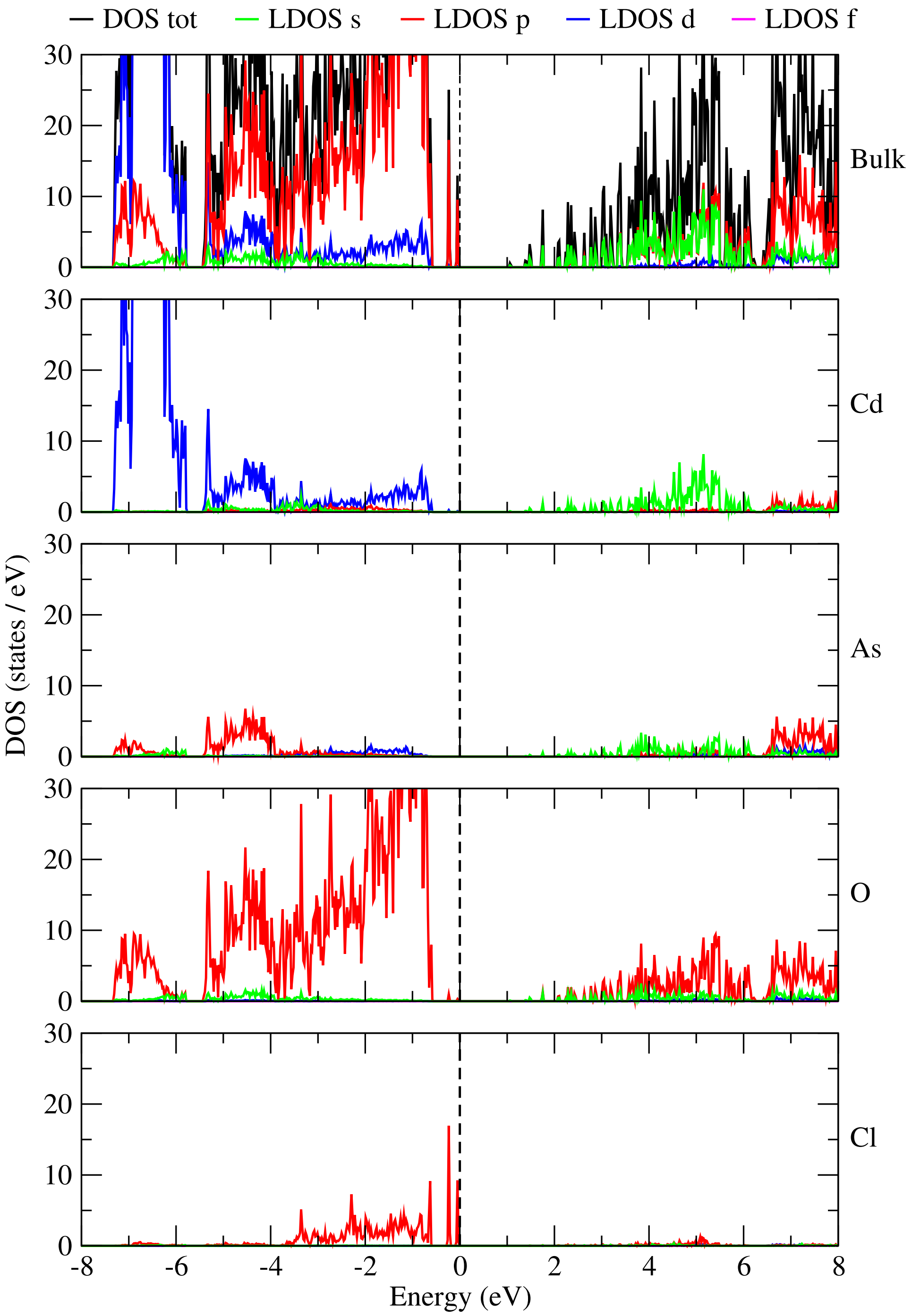

Figure S-110: Local density of states for the $Cd_{10}(AsO_4)_6Cl_2$ bulk phase.

Table S-113: Calculated properties for the $Cd_{10}(AsO_4)_6Cl_2$ bulk phase. Number of non-equivalent species, N; average distance for nearest neighbors, d_{NN}; effective coordination number, ECN; and net atomic charge, Q.

Non-equivalent species	N	d_{NN} (Å)	ECN (NNN)	Q (e^-)
Cd(I)	2	2.3352	6.2373	1.135 995
Cd(II)	2	2.3309	6.1938	1.135 629
Cd(III)	6	2.2618	5.4844	1.126 762
As(I)	6	1.7015	3.9838	1.661 305
O(I)	6	1.7015	1.0251	−0.815 133
O(II)	6	1.7554	1.0485	−0.876 482
O(III)	6	1.7255	1.0578	−0.843 101
O(IV)	6	1.7237	1.0581	−0.841 281
Cl(I)	2	2.6560	4.7015	−0.507 834

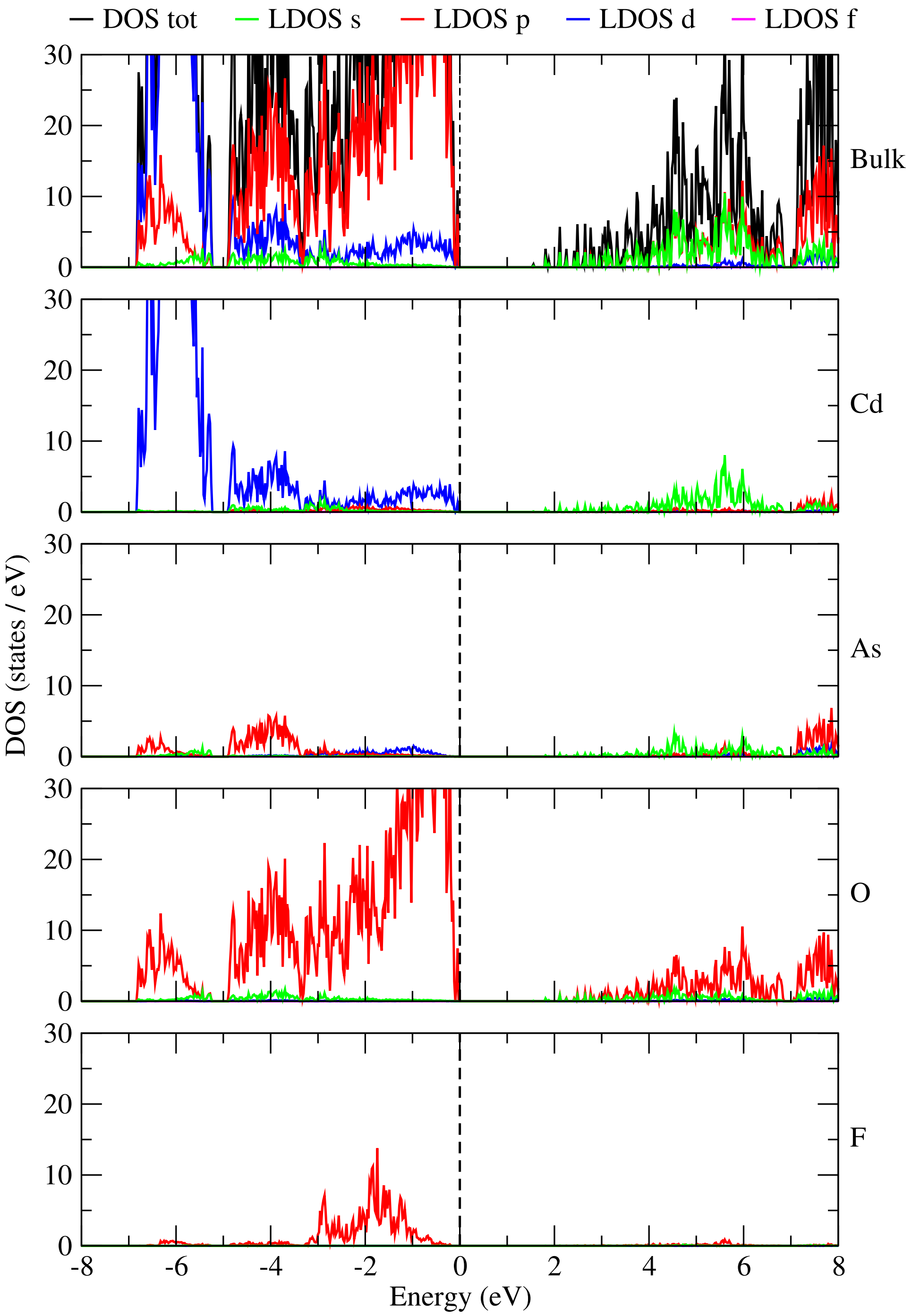

Figure S-111: Local density of states for the $Cd_{10}(AsO_4)_6F_2$ bulk phase.

Table S-114: Calculated properties for the $Cd_{10}(AsO_4)_6F_2$ bulk phase. Number of non-equivalent species, N; average distance for nearest neighbors, d_{NN}; effective coordination number, ECN; and net atomic charge, Q.

Non-equivalent species	N	d_{NN} (Å)	ECN (NNN)	Q (e^-)
Cd(I)	2	2.3169	6.1378	1.123 529
Cd(II)	2	2.3166	6.1343	1.123 584
Cd(III)	6	2.2405	5.6542	1.176 753
As(I)	6	1.6973	3.9883	1.663 768
O(I)	6	1.6973	1.0301	−0.806 474
O(II)	6	1.7412	1.0233	−0.870 477
O(III)	6	1.7281	1.0927	−0.853 914
O(IV)	6	1.7281	1.0920	−0.854 030
F(I)	2	2.3955	3.0860	−0.613 989

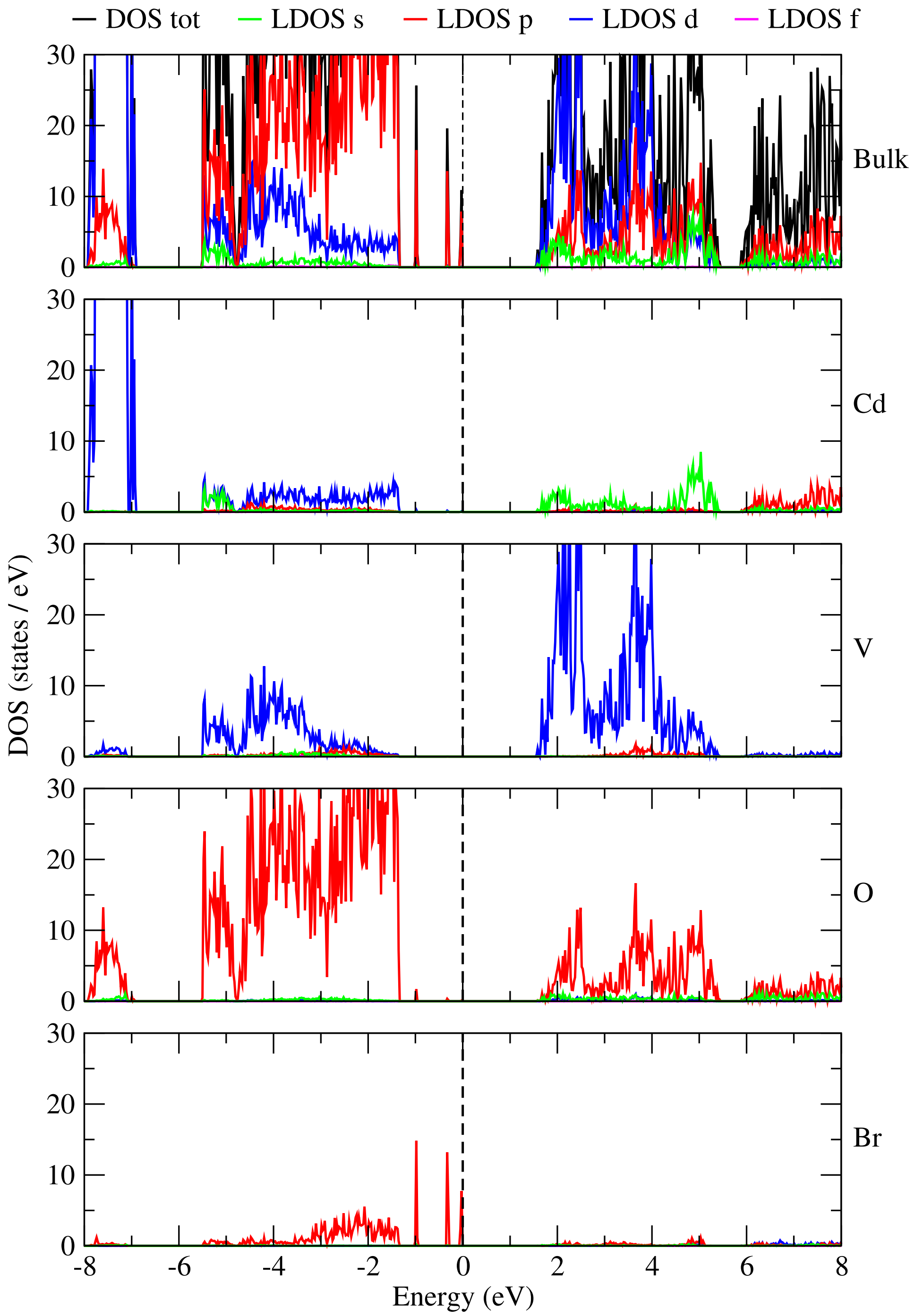

Figure S-112: Local density of states for the $Cd_{10}(VO_4)_6Br_2$ bulk phase.

Table S-115: Calculated properties for the $Cd_{10}(VO_4)_6Br_2$ bulk phase. Number of non-equivalent species, N; average distance for nearest neighbors, d_{NN}; effective coordination number, ECN; and net atomic charge, Q.

Non-equivalent species	N	d_{NN} (Å)	ECN (NNN)	Q (e^-)
Cd(I)	2	2.3425	6.1260	1.201 741
Cd(II)	2	2.3412	6.1034	1.201 290
Cd(III)	6	2.2319	4.9787	1.153 711
V(I)	6	1.7069	3.9572	1.970 202
O(I)	6	1.7069	1.0245	−0.905 529
O(II)	6	1.7839	1.1573	−1.023 587
O(III)	6	1.7135	1.0581	−0.928 052
O(IV)	6	1.7127	1.0675	−0.925 282
Br(I)	2	2.7390	7.3266	−0.427 420

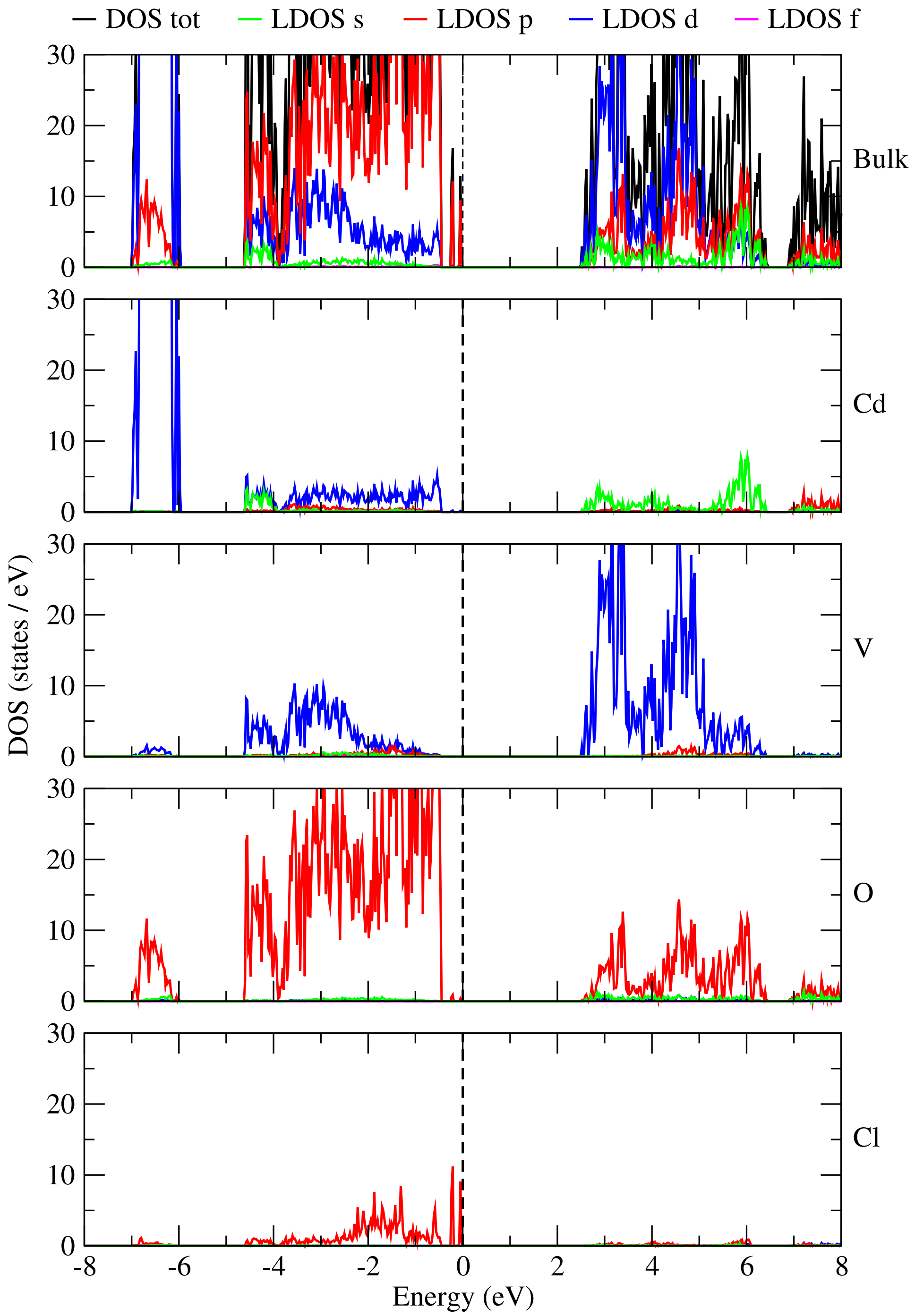

Figure S-113: Local density of states for the $Cd_{10}(VO_4)_6Cl_2$ bulk phase.

Table S-116: Calculated properties for the $Cd_{10}(VO_4)_6Cl_2$ bulk phase. Number of non-equivalent species, N; average distance for nearest neighbors, d_{NN}; effective coordination number, ECN; and net atomic charge, Q.

Non-equivalent species	N	d_{NN} (Å)	ECN (NNN)	Q (e^-)
Cd(I)	2	2.3326	6.1066	1.197 129
Cd(II)	2	2.3296	6.0790	1.197 207
Cd(III)	6	2.2254	5.2385	1.182 494
V(I)	6	1.7026	3.9600	1.971 209
O(I)	6	1.7026	1.0266	−0.898 506
O(II)	6	1.7808	1.1188	−1.025 813
O(III)	6	1.7167	1.0774	−0.933 039
O(IV)	6	1.7151	1.0772	−0.931 015
Cl(I)	2	2.6544	4.7375	−0.490 332

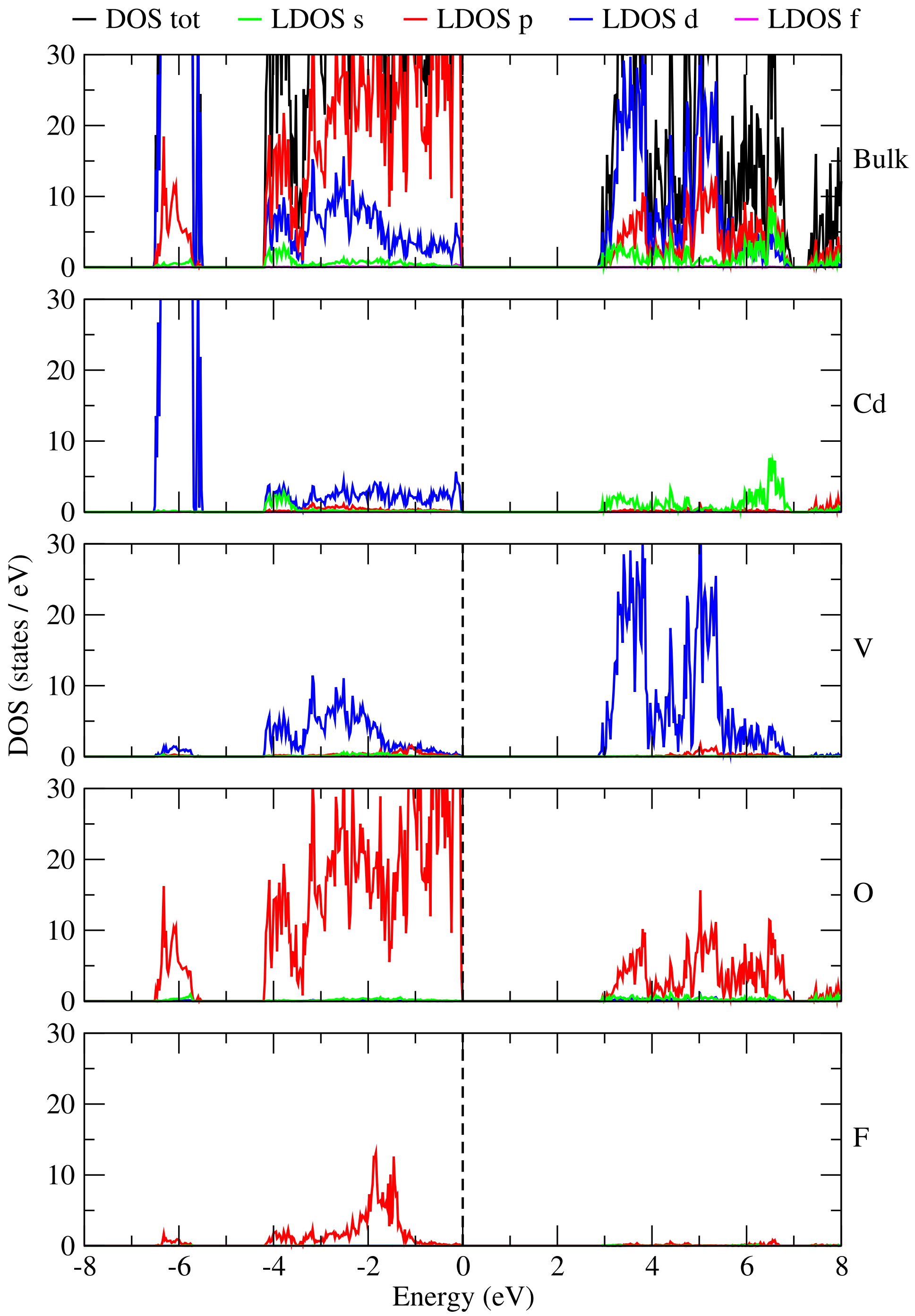

Figure S-114: Local density of states for the $Cd_{10}(VO_4)_6F_2$ bulk phase.

Table S-117: Calculated properties for the $Cd_{10}(VO_4)_6F_2$ bulk phase. Number of non-equivalent species, N; average distance for nearest neighbors, d_{NN}; effective coordination number, ECN; and net atomic charge, Q.

Non-equivalent species	N	d_{NN} (Å)	ECN (NNN)	Q (e^-)
Cd(I)	2	2.3218	6.0703	1.185 672
Cd(II)	2	2.3218	6.0703	1.185 672
Cd(III)	6	2.2000	5.4467	1.233 283
V(I)	6	1.6967	3.9761	1.977 340
O(I)	6	1.6967	1.0272	−0.886 414
O(II)	6	1.7621	1.0469	−1.015 476
O(III)	6	1.7235	1.1267	−0.948 331
O(IV)	6	1.7235	1.1267	−0.948 331
F(I)	2	2.3898	3.0725	−0.607 560

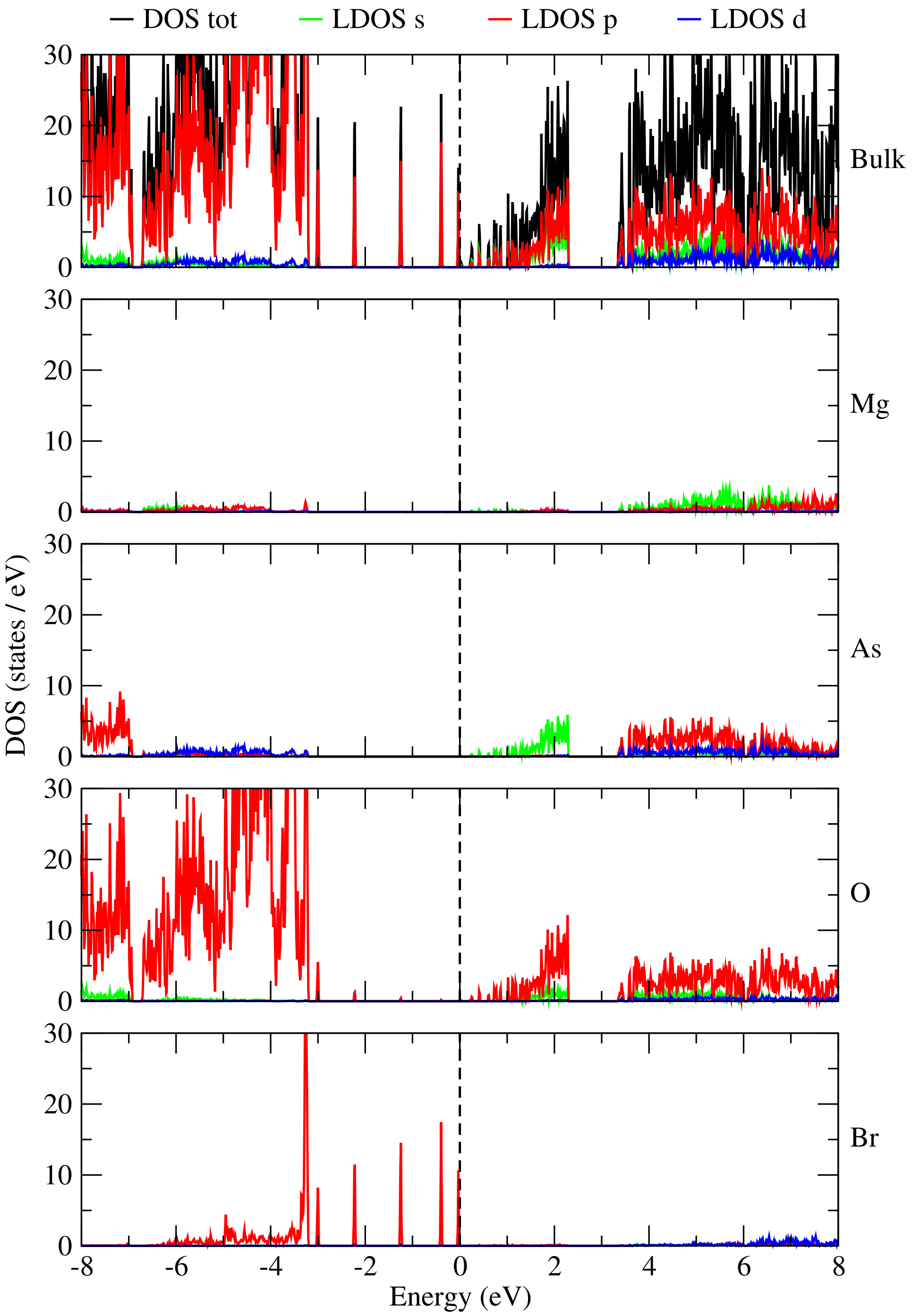

Figure S-115: Local density of states for the $Mg_{10}(AsO_4)_6Br_2$ bulk phase.

Table S-118: Calculated properties for the $Mg_{10}(AsO_4)_6Br_2$ bulk phase. Number of non-equivalent species, N; average distance for nearest neighbors, d_{NN}; effective coordination number, ECN; and net atomic charge, Q.

Non-equivalent species	N	d_{NN} (Å)	ECN (NNN)	Q (e^-)
Mg(I)	2	2.1278	5.9394	1.497 213
Mg(II)	2	2.1281	5.9405	1.497 024
Mg(III)	6	2.0882	5.0077	1.432 587
As(I)	6	1.6910	3.9716	1.728 583
O(I)	6	1.6910	1.4969	−0.948 664
O(II)	6	1.7615	2.2391	−1.069 080
O(III)	6	1.7161	1.4462	−0.962 632
O(IV)	6	1.7171	1.4460	−0.963 375
Br(I)	2	2.6409	9.9544	−0.646 497

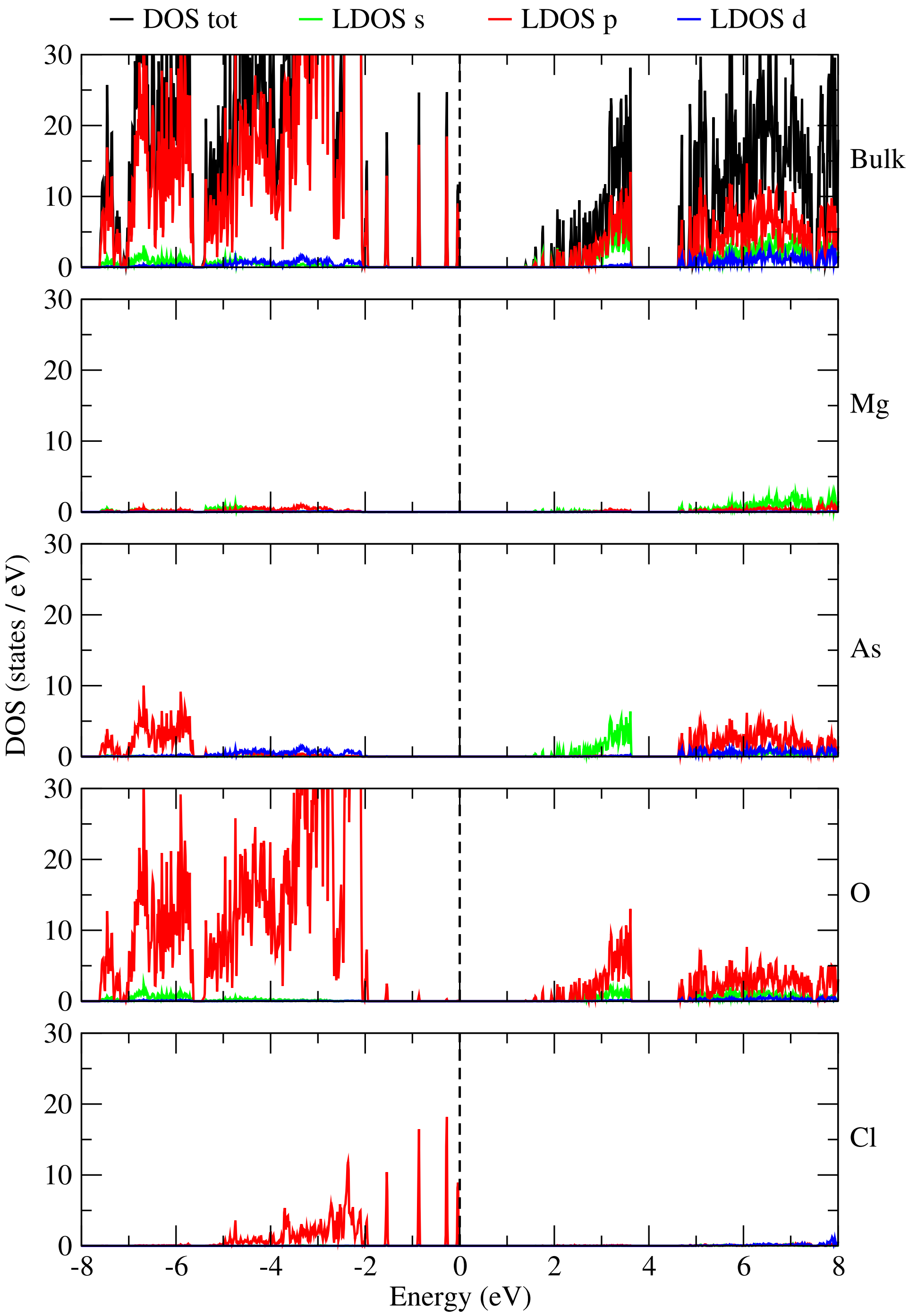

Figure S-116: Local density of states for the $Mg_{10}(AsO_4)_6Cl_2$ bulk phase.

Table S-119: Calculated properties for the $Mg_{10}(AsO_4)_6Cl_2$ bulk phase. Number of non-equivalent species, N; average distance for nearest neighbors, d_{NN}; effective coordination number, ECN; and net atomic charge, Q.

Non-equivalent species	N	d_{NN} (Å)	ECN (NNN)	Q (e^-)
Mg(I)	2	2.1147	5.9369	1.494 439
Mg(II)	2	2.1136	5.9316	1.494 525
Mg(III)	6	2.0727	5.2271	1.445 982
As(I)	6	1.6862	3.9723	1.736 849
O(I)	6	1.6862	1.5468	−0.946 524
O(II)	6	1.7567	2.0548	−1.063 100
O(III)	6	1.7189	1.5992	−0.973 022
O(IV)	6	1.7188	1.5981	−0.972 350
Cl(I)	2	2.5463	7.7405	−0.672 467

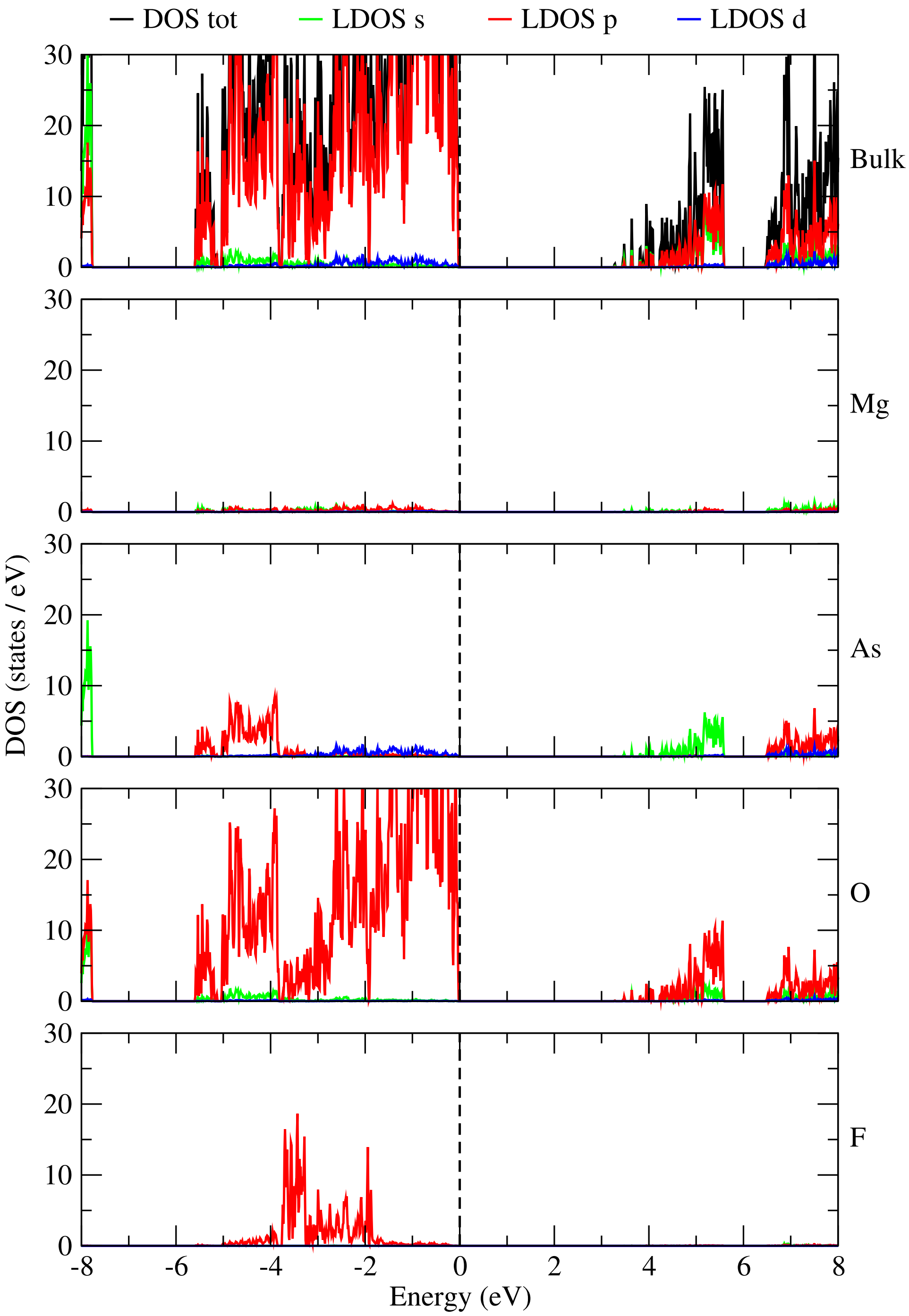

Figure S-117: Local density of states for the $Mg_{10}(AsO_4)_6F_2$ bulk phase.

Table S-120: Calculated properties for the $Mg_{10}(AsO_4)_6F_2$ bulk phase. Number of non-equivalent species, N; average distance for nearest neighbors, d_{NN}; effective coordination number, ECN; and net atomic charge, Q.

Non-equivalent species	N	d_{NN} (Å)	ECN (NNN)	Q (e^-)
Mg(I)	2	2.1076	5.9907	1.488 085
Mg(II)	2	2.1076	5.9907	1.488 085
Mg(III)	6	2.0241	4.9239	1.480 934
As(I)	6	1.6864	3.9846	1.776 209
O(I)	6	1.6864	1.5908	−0.949 256
O(II)	6	1.7277	1.6005	−1.017 132
O(III)	6	1.7292	2.0897	−1.020 520
O(IV)	6	1.7292	2.0897	−1.020 520
F(I)	2	2.1452	3.0320	−0.725 318

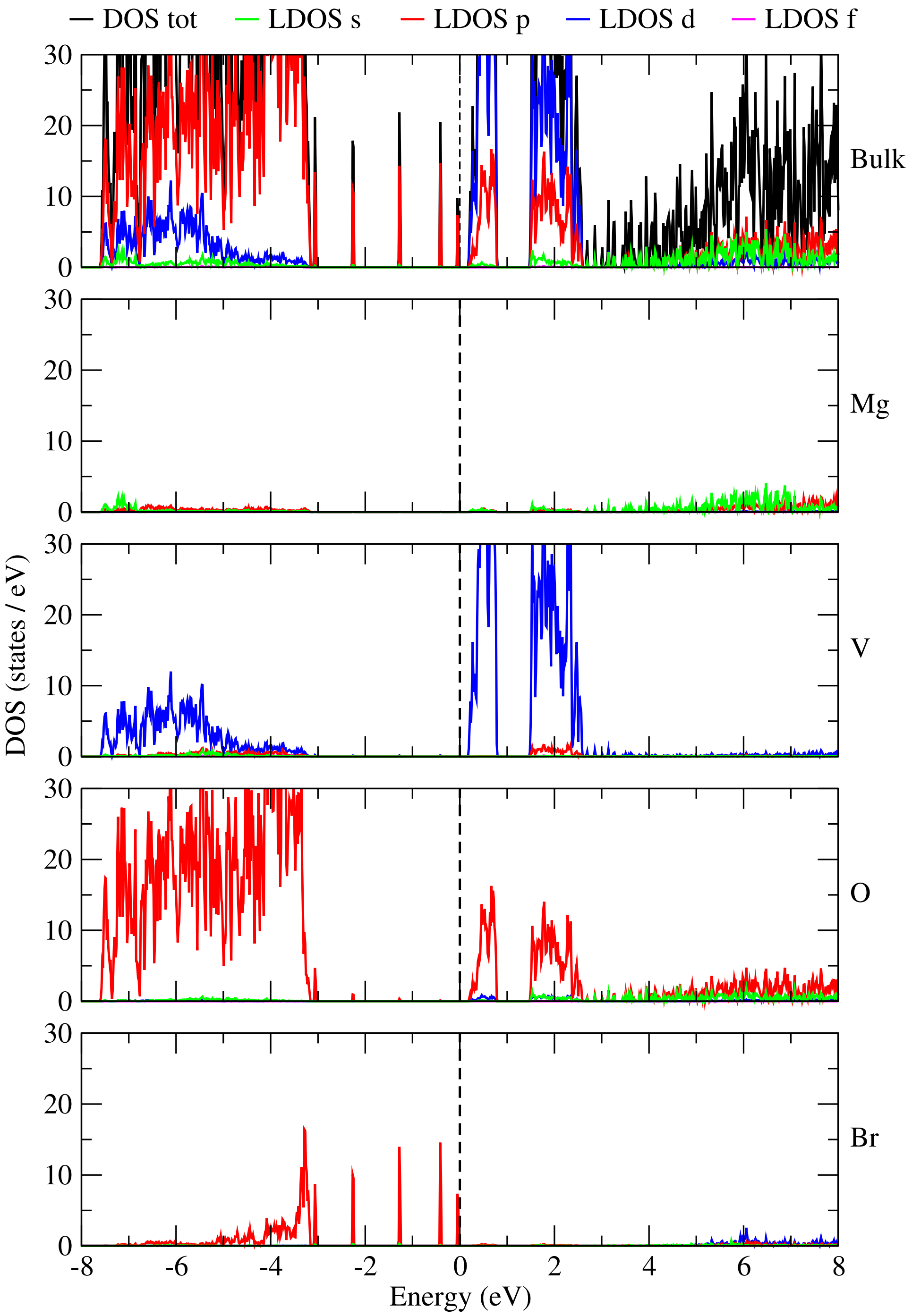

Figure S-118: Local density of states for the $Mg_{10}(VO_4)_6Br_2$ bulk phase.

Table S-121: Calculated properties for the $Mg_{10}(VO_4)_6Br_2$ bulk phase. Number of non-equivalent species, N; average distance for nearest neighbors, d_{NN}; effective coordination number, ECN; and net atomic charge, Q.

Non-equivalent species	N	d_{NN} (Å)	ECN (NNN)	Q (e^-)
Mg(I)	2	2.1466	5.9932	1.536 141
Mg(II)	2	2.1463	5.9917	1.535 821
Mg(III)	6	2.0724	4.8829	1.470 084
V(I)	6	1.6961	3.9472	2.026 015
O(I)	6	1.6961	1.4247	−1.018 845
O(II)	6	1.7855	2.6906	−1.226 635
O(III)	6	1.7105	1.4550	−1.034 466
O(IV)	6	1.7104	1.4534	−1.034 766
Br(I)	2	2.6486	10.2950	−0.616 124

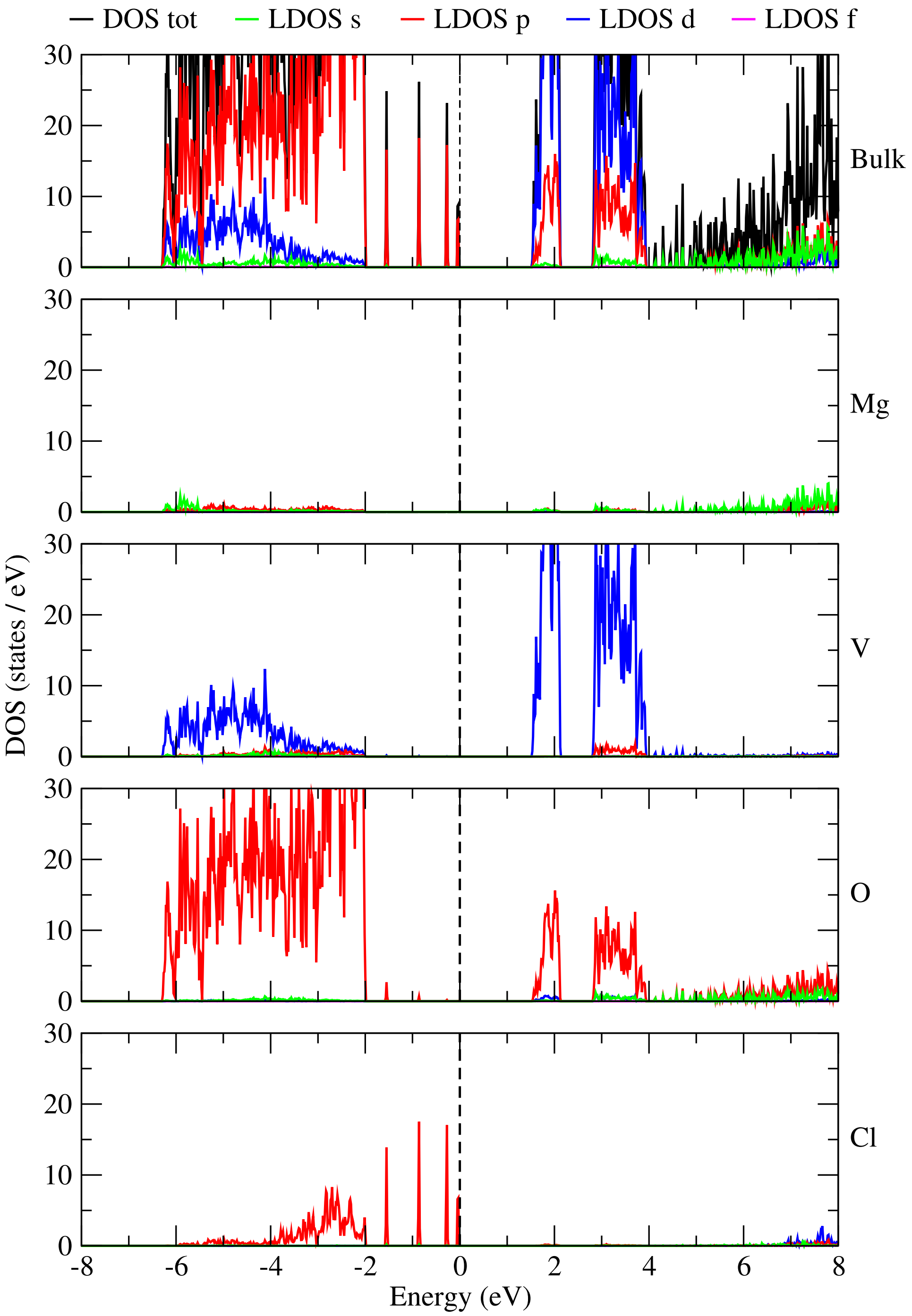

Figure S-119: Local density of states for the $Mg_{10}(VO_4)_6Cl_2$ bulk phase.

Table S-122: Calculated properties for the $Mg_{10}(VO_4)_6Cl_2$ bulk phase. Number of non-equivalent species, N; average distance for nearest neighbors, d_{NN}; effective coordination number, ECN; and net atomic charge, Q.

Non-equivalent species	N	d_{NN} (Å)	ECN (NNN)	Q (e^-)
Mg(I)	2	2.1342	5.9874	1.532 569
Mg(II)	2	2.1338	5.9858	1.532 656
Mg(III)	6	2.0583	5.1404	1.483 370
V(I)	6	1.6917	3.9530	2.031 564
O(I)	6	1.6917	1.4658	−1.012 834
O(II)	6	1.7792	2.5742	−1.219 964
O(III)	6	1.7128	1.5789	−1.044 110
O(IV)	6	1.7126	1.5755	−1.043 330
Cl(I)	2	2.5540	8.0765	−0.649 313

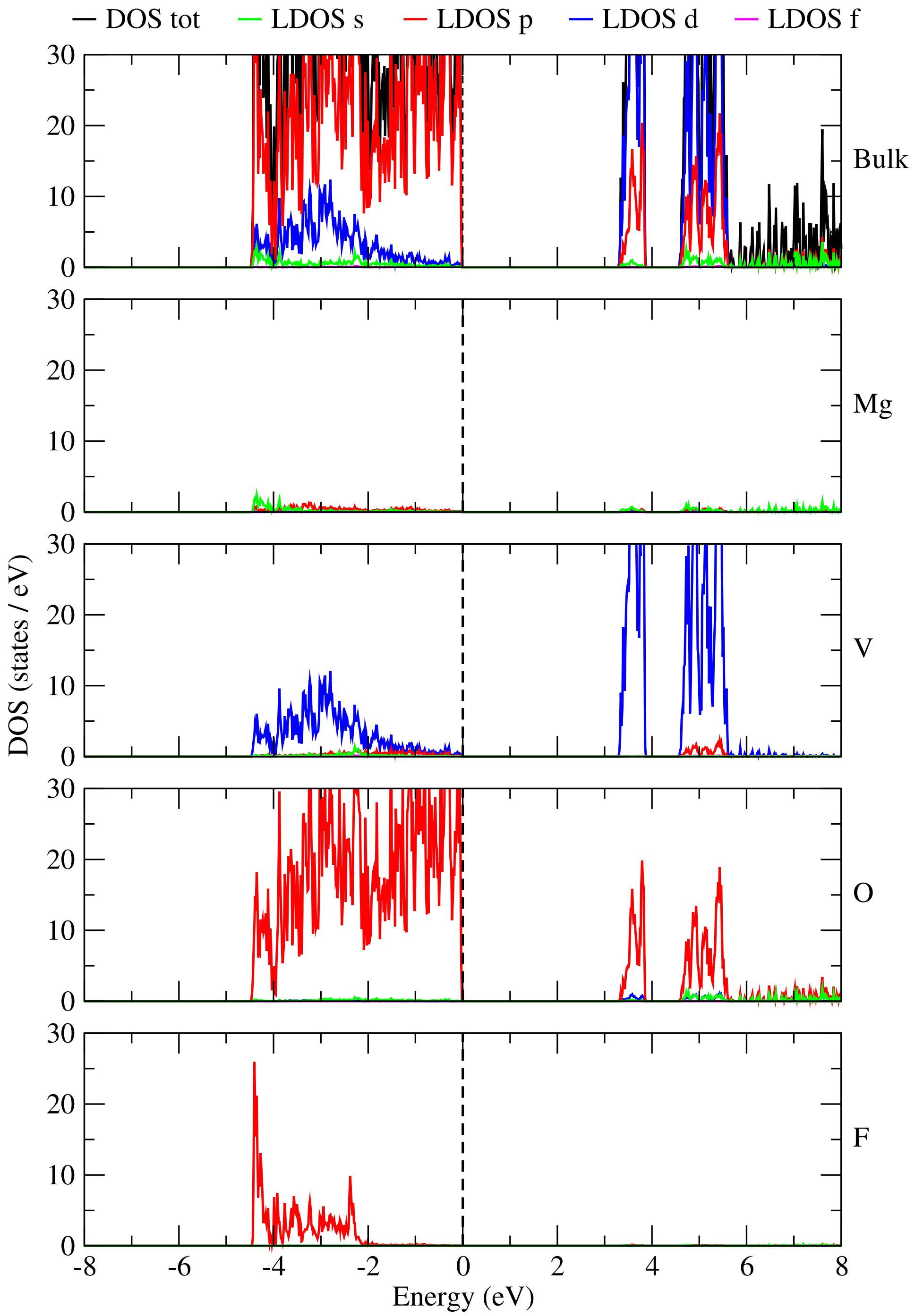

Figure S-120: Local density of states for the $Mg_{10}(VO_4)_6F_2$ bulk phase.

Table S-123: Calculated properties for the $Mg_{10}(VO_4)_6F_2$ bulk phase. Number of non-equivalent species, N; average distance for nearest neighbors, d_{NN}; effective coordination number, ECN; and net atomic charge, Q.

Non-equivalent species	N	d_{NN} (Å)	ECN (NNN)	Q (e^-)
Mg(I)	2	2.1244	6.0147	1.528 167
Mg(II)	2	2.1244	6.0147	1.528 167
Mg(III)	6	2.0039	4.8610	1.508 180
V(I)	6	1.6961	3.9891	2.071 742
O(I)	6	1.6961	1.5599	−1.021 643
O(II)	6	1.7362	1.6824	−1.132 506
O(III)	6	1.7288	2.1010	−1.103 164
O(IV)	6	1.7288	2.1010	−1.103 164
F(I)	2	2.1148	3.0200	−0.714 669

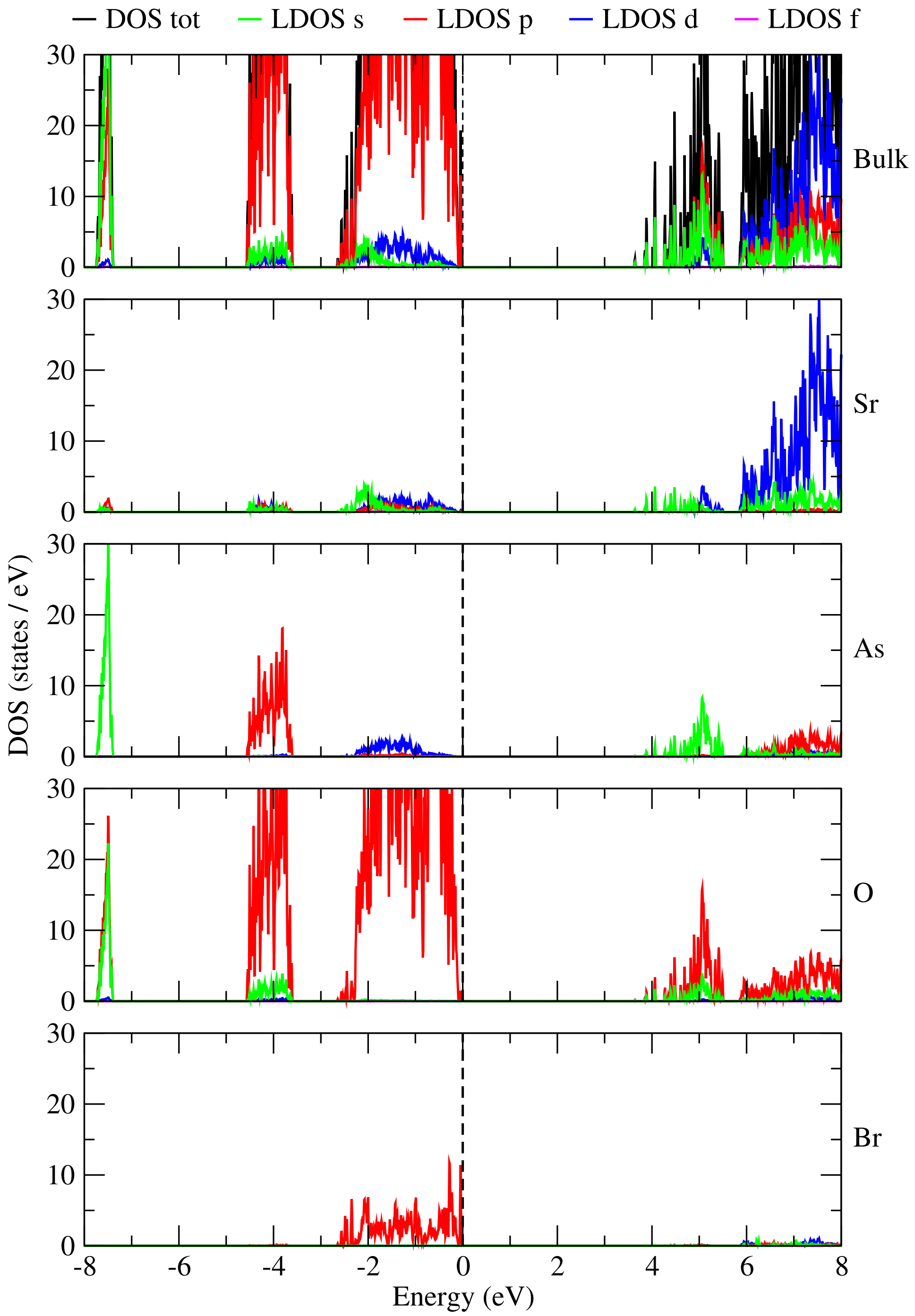

Figure S-121: Local density of states for the $Sr_{10}(AsO_4)_6Br_2$ bulk phase.

Table S-124: Calculated properties for the $Sr_{10}(AsO_4)_6Br_2$ bulk phase. Number of non-equivalent species, N; average distance for nearest neighbors, d_{NN}; effective coordination number, ECN; and net atomic charge, Q.

Non-equivalent species	N	d_{NN} (Å)	ECN (NNN)	Q (e^-)
Sr(I)	2	2.5799	7.9030	1.459 621
Sr(II)	2	2.5753	7.7581	1.460 583
Sr(III)	6	2.4529	5.0728	1.482 148
As(I)	6	1.7131	3.9976	1.671 376
O(I)	6	1.7131	1.0002	−0.939 284
O(II)	6	1.7332	1.0033	−1.002 816
O(III)	6	1.7192	1.0005	−0.967 266
O(IV)	6	1.7209	1.0005	−0.969 563
Br(I)	2	3.2633	13.6346	−0.743 985

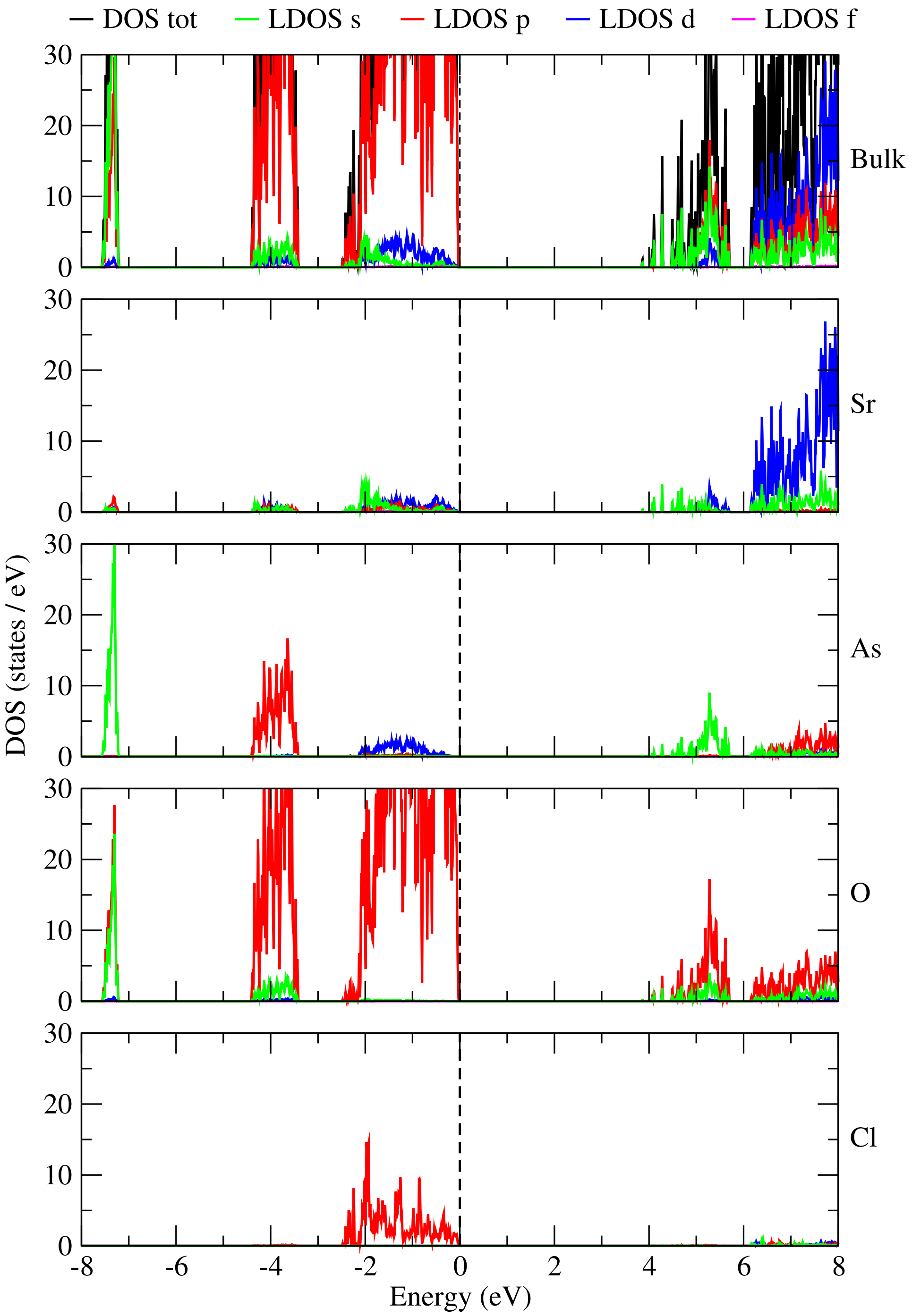

Figure S-122: Local density of states for the $Sr_{10}(AsO_4)_6Cl_2$ bulk phase.

Table S-125: Calculated properties for the $Sr_{10}(AsO_4)_6Cl_2$ bulk phase. Number of non-equivalent species, N; average distance for nearest neighbors, d_{NN}; effective coordination number, ECN; and net atomic charge, Q.

Non-equivalent species	N	d_{NN} (Å)	ECN (NNN)	Q (e^-)
Sr(I)	2	2.5942	8.4790	1.454 012
Sr(II)	2	2.5536	7.4047	1.458 282
Sr(III)	6	2.4726	5.4824	1.489 187
As(I)	6	1.7142	3.9986	1.665 212
O(I)	6	1.7142	1.0003	−0.938 887
O(II)	6	1.7297	1.0021	−0.997 795
O(III)	6	1.7200	1.0004	−0.967 882
O(IV)	6	1.7187	1.0007	−0.971 174
Cl(I)	2	3.0504	12.8734	−0.748 277

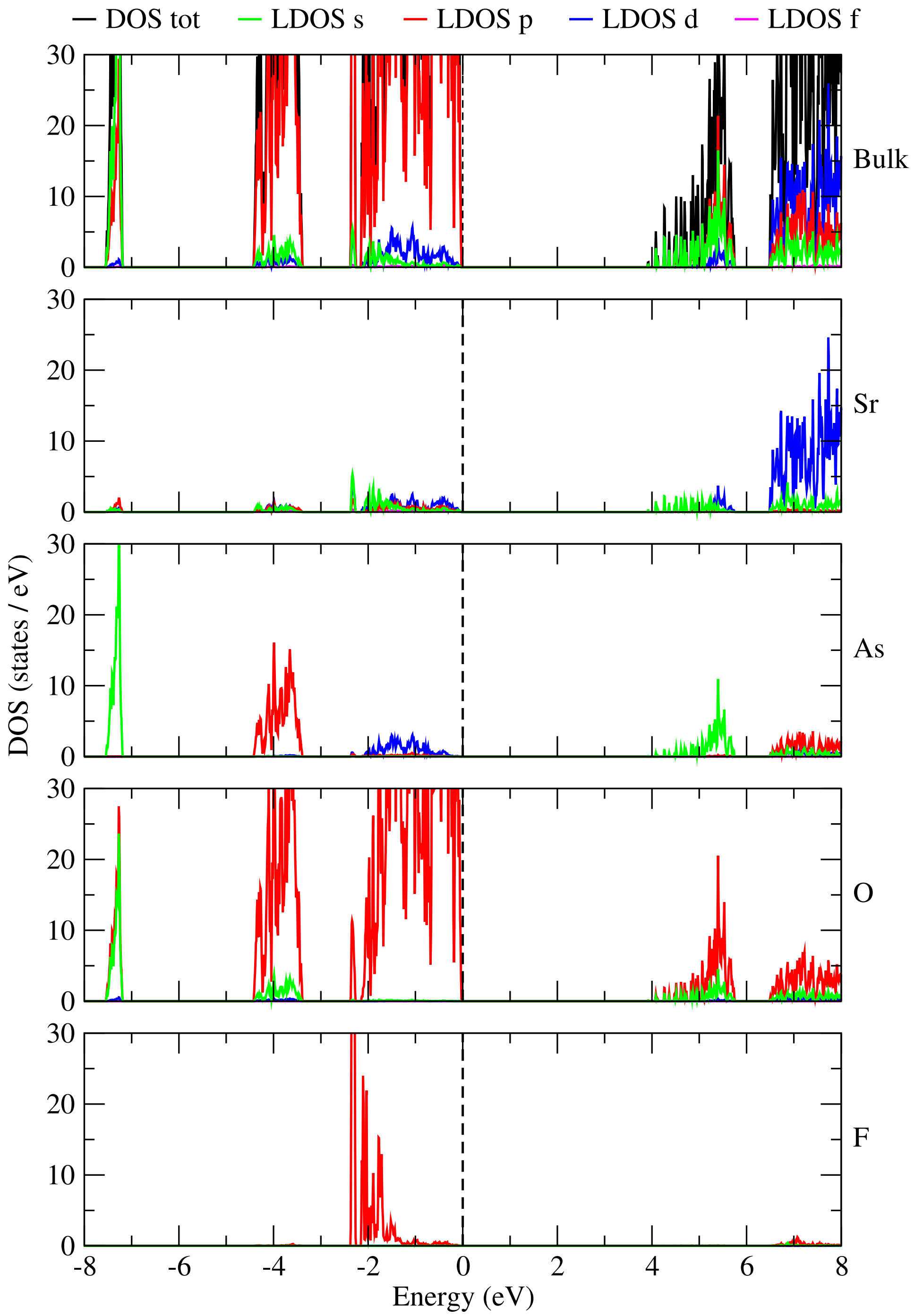

Figure S-123: Local density of states for the $Sr_{10}(AsO_4)_6F_2$ bulk phase.

Table S-126: Calculated properties for the $Sr_{10}(AsO_4)_6F_2$ bulk phase. Number of non-equivalent species, N; average distance for nearest neighbors, d_{NN}; effective coordination number, ECN; and net atomic charge, Q.

Non-equivalent species	N	d_{NN} (Å)	ECN (NNN)	Q (e^-)
Sr(I)	2	2.5828	8.1381	1.452 238
Sr(II)	2	2.5687	7.7217	1.454 692
Sr(III)	6	2.4792	6.6288	1.493 864
As(I)	6	1.7174	3.9996	1.646 088
O(I)	6	1.7174	1.0003	−0.944 351
O(II)	6	1.7248	1.0005	−0.990 090
O(III)	6	1.7188	1.0004	−0.963 144
O(IV)	6	1.7190	1.0004	−0.963 601
F(I)	2	2.4792	3.0719	−0.743 228

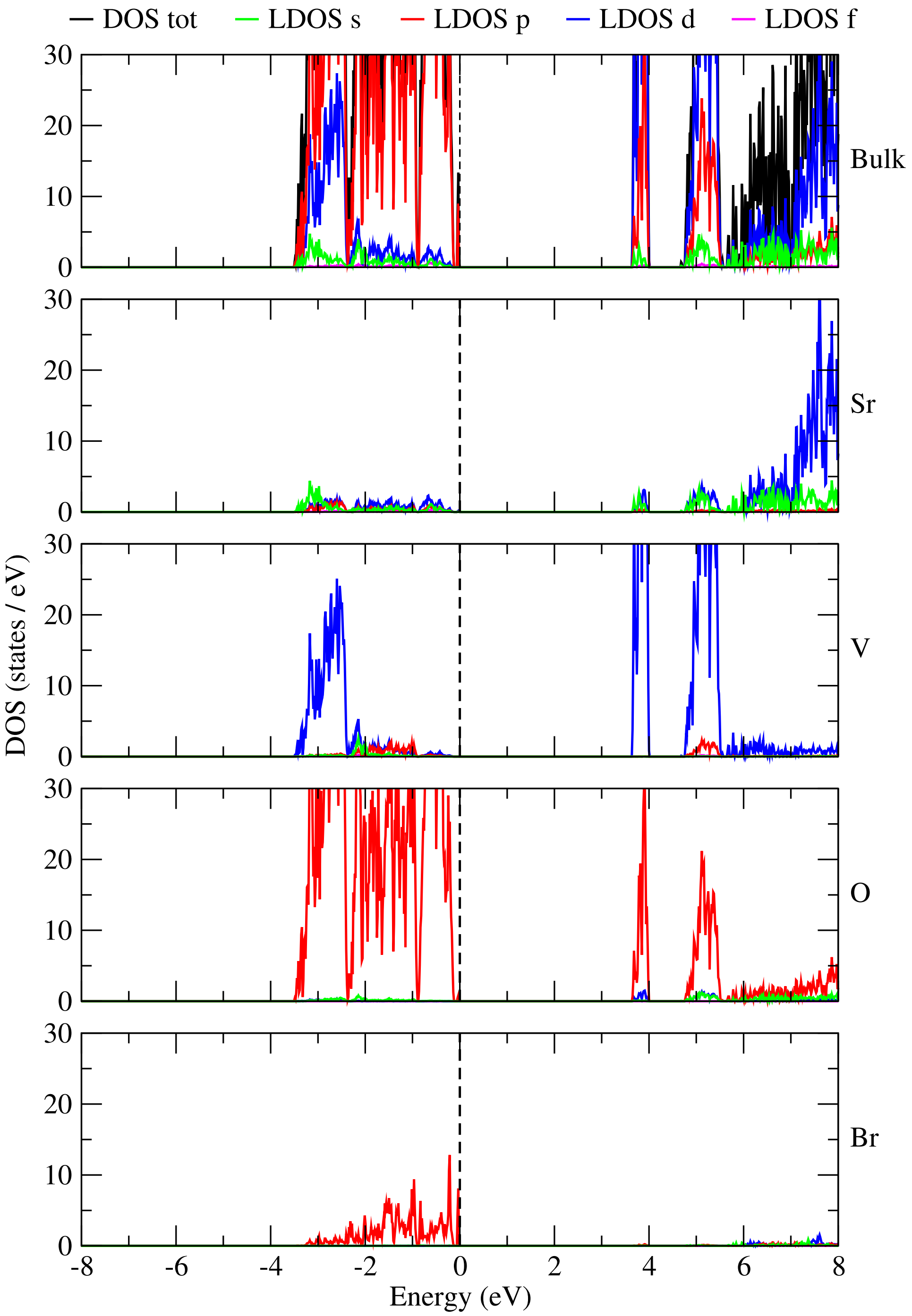

Figure S-124: Local density of states for the $Sr_{10}(VO_4)_6Br_2$ bulk phase.

Table S-127: Calculated properties for the $Sr_{10}(VO_4)_6Br_2$ bulk phase. Number of non-equivalent species, N; average distance for nearest neighbors, d_{NN}; effective coordination number, ECN; and net atomic charge, Q.

Non-equivalent species	N	d_{NN} (Å)	ECN (NNN)	Q (e^-)
Sr(I)	2	2.5580	7.4403	1.521 413
Sr(II)	2	2.5620	7.5303	1.520 810
Sr(III)	6	2.4422	5.0655	1.516 084
V(I)	6	1.7160	3.9956	1.984 203
O(I)	6	1.7160	1.0004	−1.043 922
O(II)	6	1.7407	1.0051	−1.125 265
O(III)	6	1.7181	1.0005	−1.052 092
O(IV)	6	1.7187	1.0005	−1.052 243
Br(I)	2	3.2818	13.6760	−0.722 517

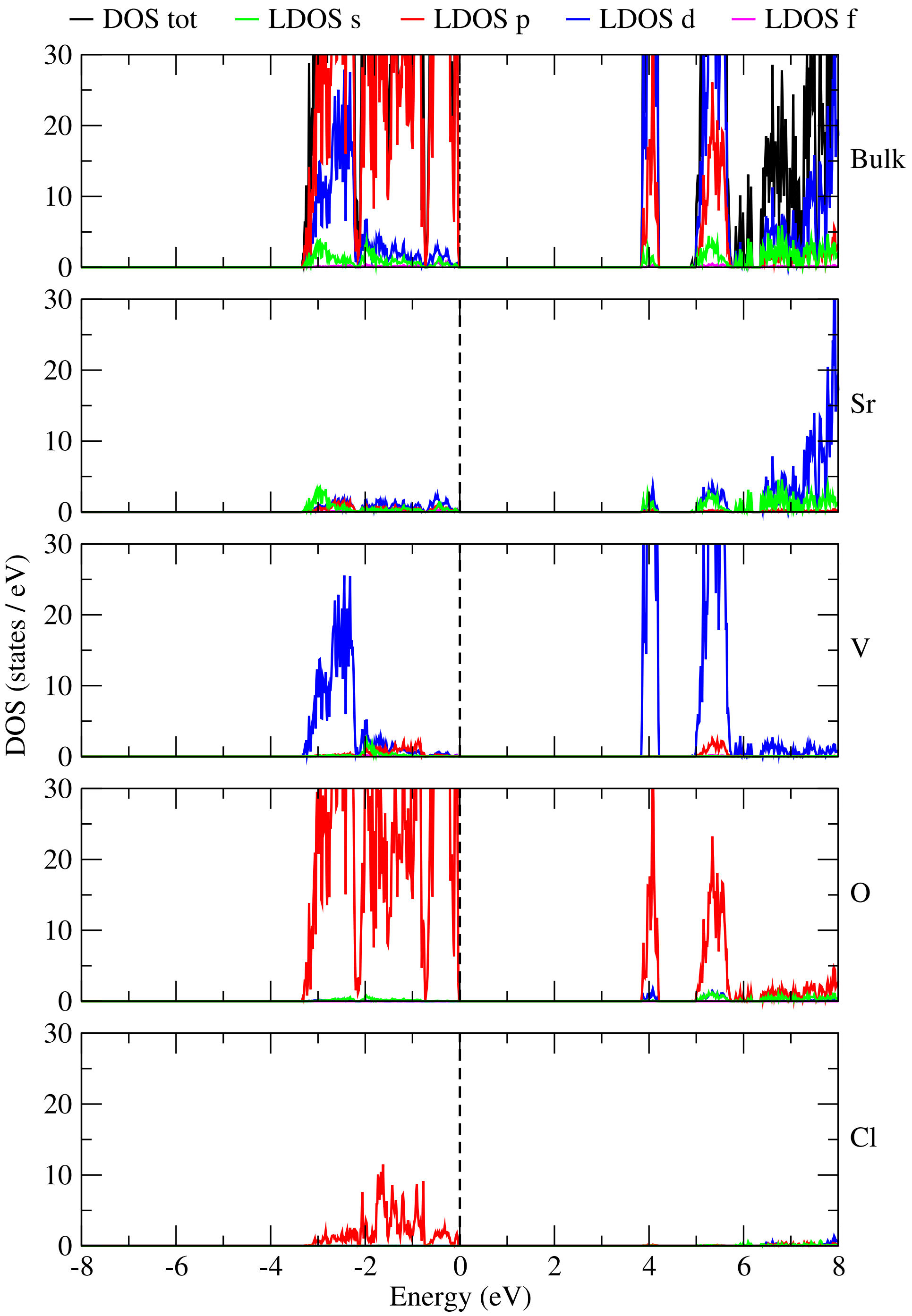

Figure S-125: Local density of states for the $Sr_{10}(VO_4)_6Cl_2$ bulk phase.

Table S-128: Calculated properties for the $Sr_{10}(VO_4)_6Cl_2$ bulk phase. Number of non-equivalent species, N; average distance for nearest neighbors, d_{NN}; effective coordination number, ECN; and net atomic charge, Q.

Non-equivalent species	N	d_{NN} (Å)	ECN (NNN)	Q (e^-)
Sr(I)	2	2.5590	7.8362	1.514 196
Sr(II)	2	2.5479	7.5280	1.514 269
Sr(III)	6	2.4657	5.3438	1.524 429
V(I)	6	1.7156	3.9963	1.977 053
O(I)	6	1.7175	1.0004	−1.044 467
O(II)	6	1.7377	1.0030	−1.119 065
O(III)	6	1.7168	1.0005	−1.051 222
O(IV)	6	1.7156	1.0006	−1.051 299
Cl(I)	2	3.1378	13.3093	−0.734 754

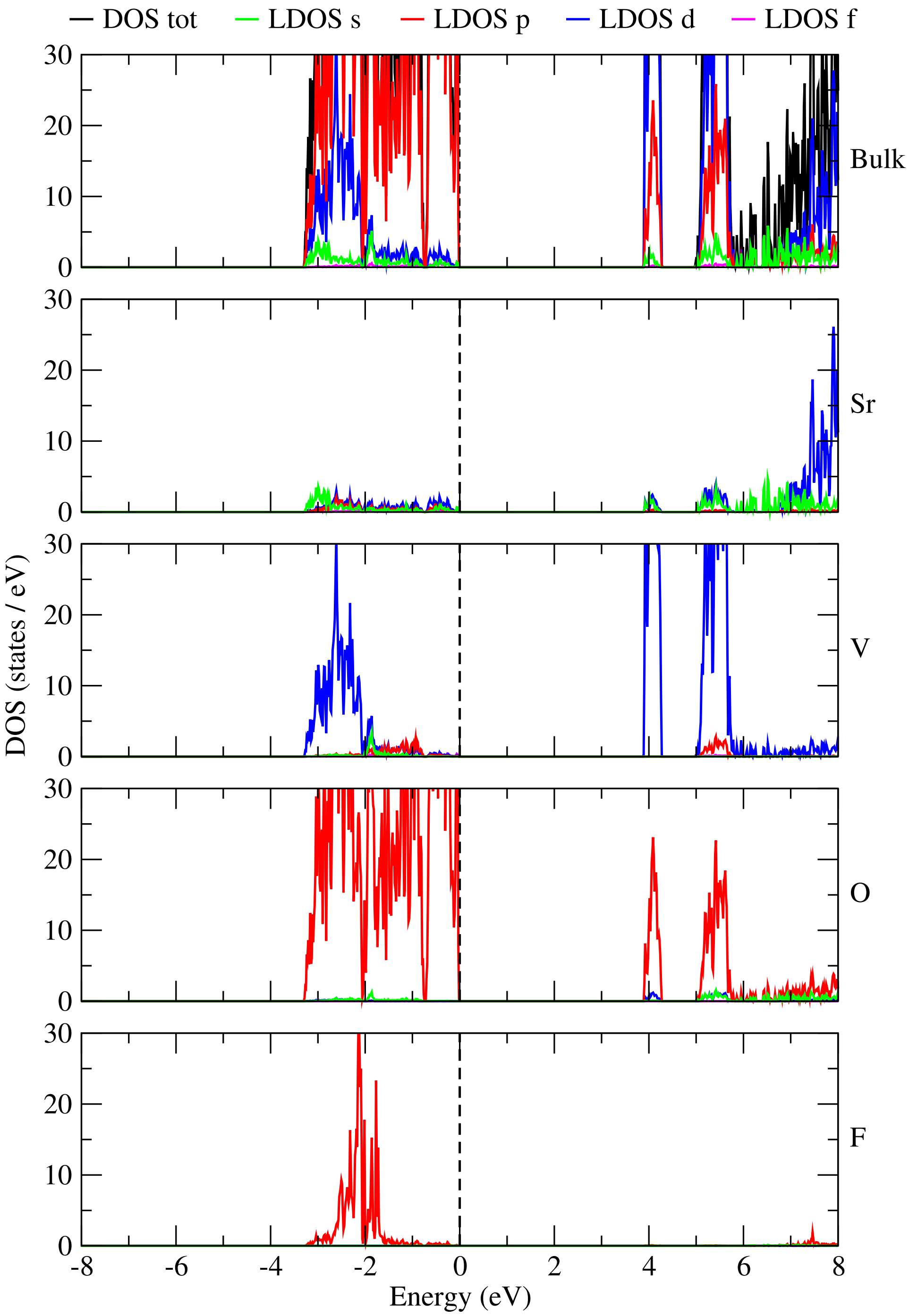

Figure S-126: Local density of states for the $Sr_{10}(VO_4)_6F_2$ bulk phase.

Table S-129: Calculated properties for the $Sr_{10}(VO_4)_6F_2$ bulk phase. Number of non-equivalent species, N; average distance for nearest neighbors, d_{NN}; effective coordination number, ECN; and net atomic charge, Q.

Non-equivalent species	N	d_{NN} (Å)	ECN (NNN)	Q (e^-)
Sr(I)	2	2.5781	7.9934	1.510 829
Sr(II)	2	2.5653	7.6362	1.513 099
Sr(III)	6	2.4740	6.6920	1.528 507
V(I)	6	1.7122	3.9969	1.961 849
O(I)	6	1.7257	1.0004	−1.065 435
O(II)	6	1.7317	1.0007	−1.115 240
O(III)	6	1.7130	1.0004	−1.036 133
O(IV)	6	1.7122	1.0003	−1.035 251
F(I)	2	2.4740	3.0924	−0.738 820

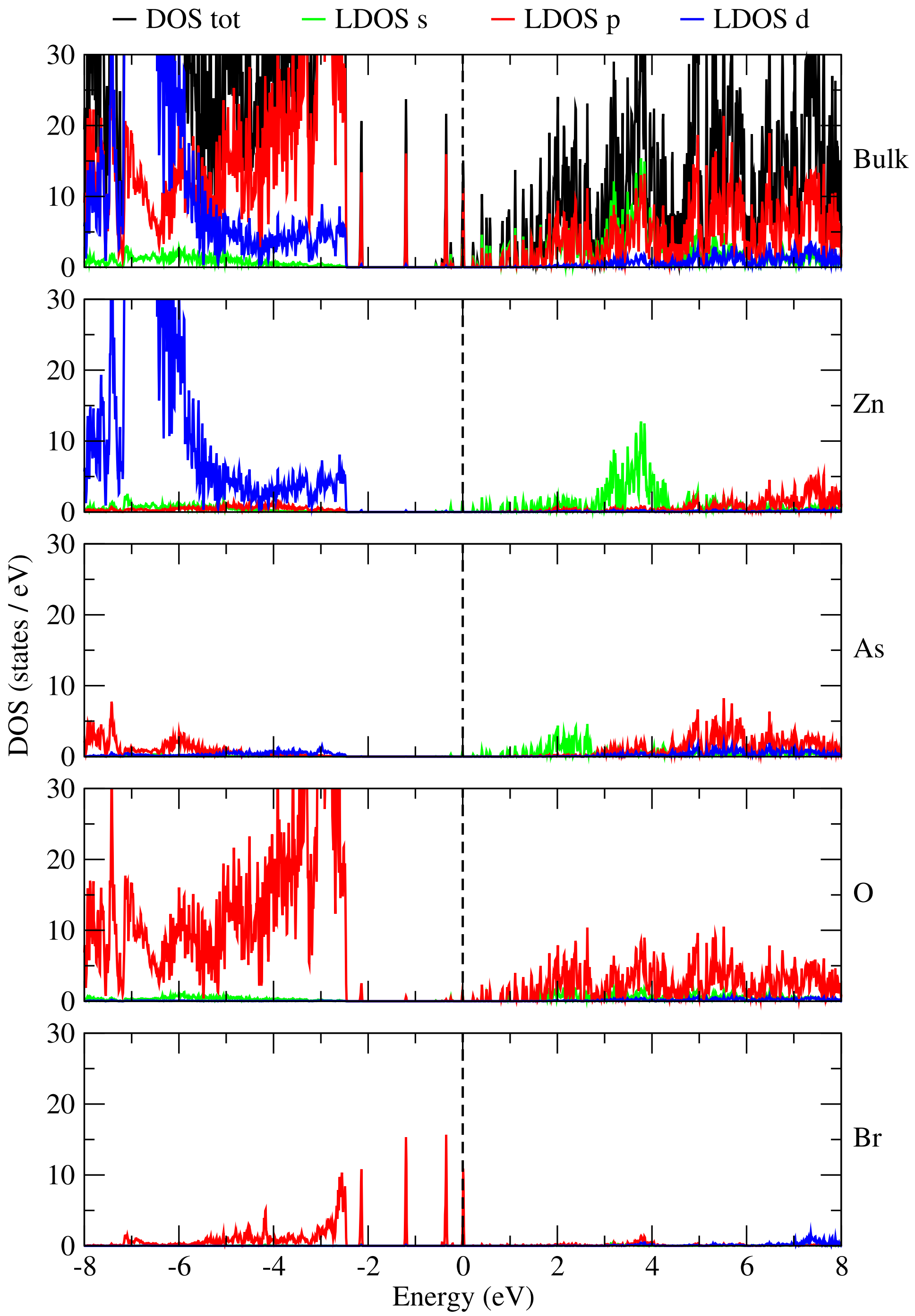

Figure S-127: Local density of states for the $Zn_{10}(AsO_4)_6Br_2$ bulk phase.

Table S-130: Calculated properties for the $Zn_{10}(AsO_4)_6Br_2$ bulk phase. Number of non-equivalent species, N; average distance for nearest neighbors, d_{NN}; effective coordination number, ECN; and net atomic charge, Q.

Non-equivalent species	N	d_{NN} (Å)	ECN (NNN)	Q (e^-)
Zn(I)	2	2.1308	5.9378	1.081 736
Zn(II)	2	2.1328	5.9466	1.081 438
Zn(III)	6	2.0437	4.7280	1.009 610
As(I)	6	1.6980	3.9687	1.672 894
O(I)	6	1.6980	1.5284	−0.794 272
O(II)	6	1.7718	2.1579	−0.867 780
O(III)	6	1.7230	1.5827	−0.810 676
O(IV)	6	1.7229	1.5806	−0.811 062
Br(I)	2	2.6166	9.5164	−0.359 321

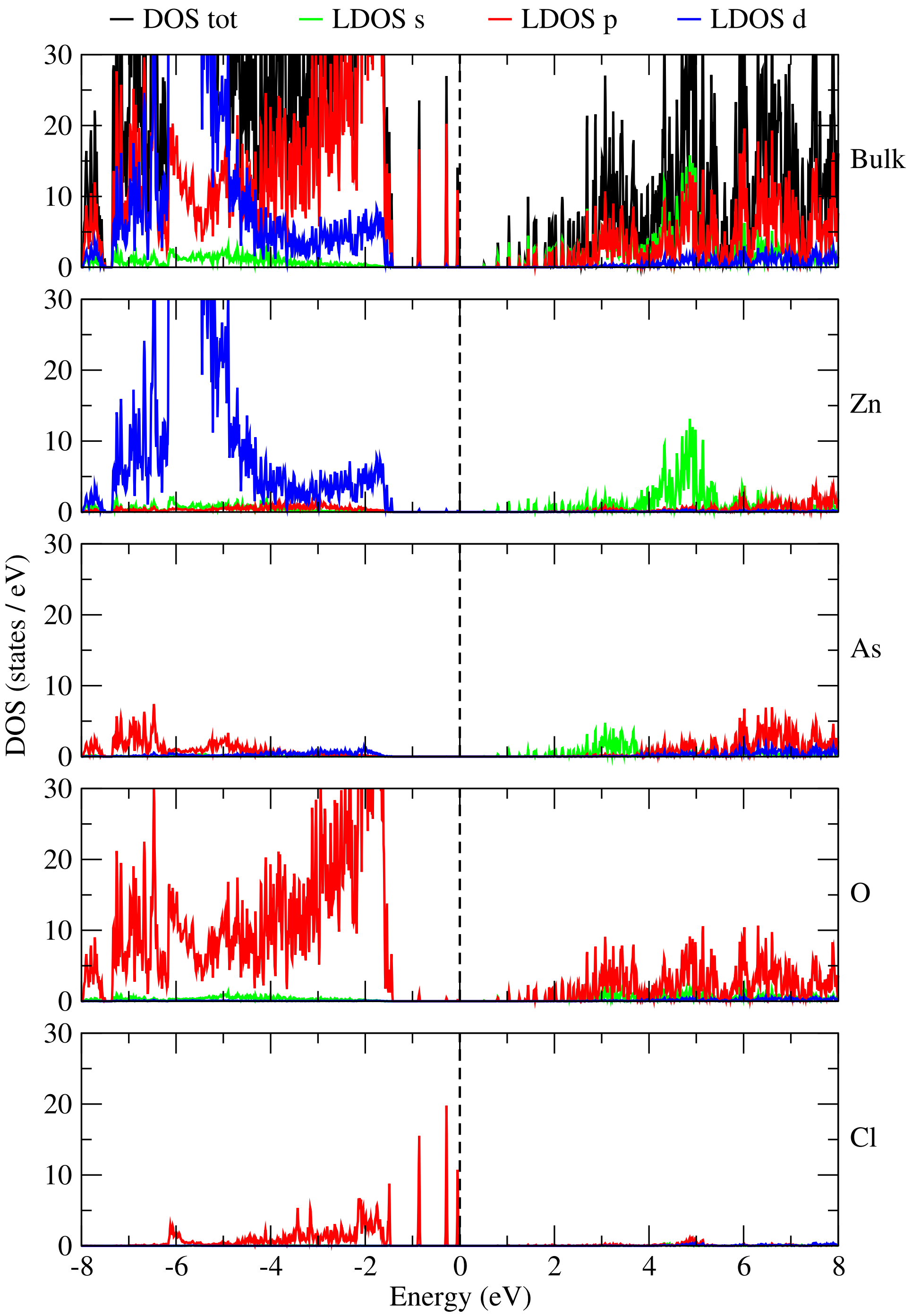

Figure S-128: Local density of states for the $Zn_{10}(AsO_4)_6Cl_2$ bulk phase.

Table S-131: Calculated properties for the $Zn_{10}(AsO_4)_6Cl_2$ bulk phase. Number of non-equivalent species, N; average distance for nearest neighbors, d_{NN}; effective coordination number, ECN; and net atomic charge, Q.

Non-equivalent species	N	d_{NN} (Å)	ECN (NNN)	Q (e^-)
Zn(I)	2	2.1277	5.9685	1.084 814
Zn(II)	2	2.1252	5.9602	1.084 519
Zn(III)	6	2.0342	4.9051	1.040 568
As(I)	6	1.6946	3.9780	1.685 906
O(I)	6	1.6946	1.5356	−0.795 457
O(II)	6	1.7576	1.6791	−0.868 803
O(III)	6	1.7263	1.7173	−0.817 006
O(IV)	6	1.7258	1.7108	−0.816 144
Cl(I)	2	2.4723	4.9757	−0.456 522

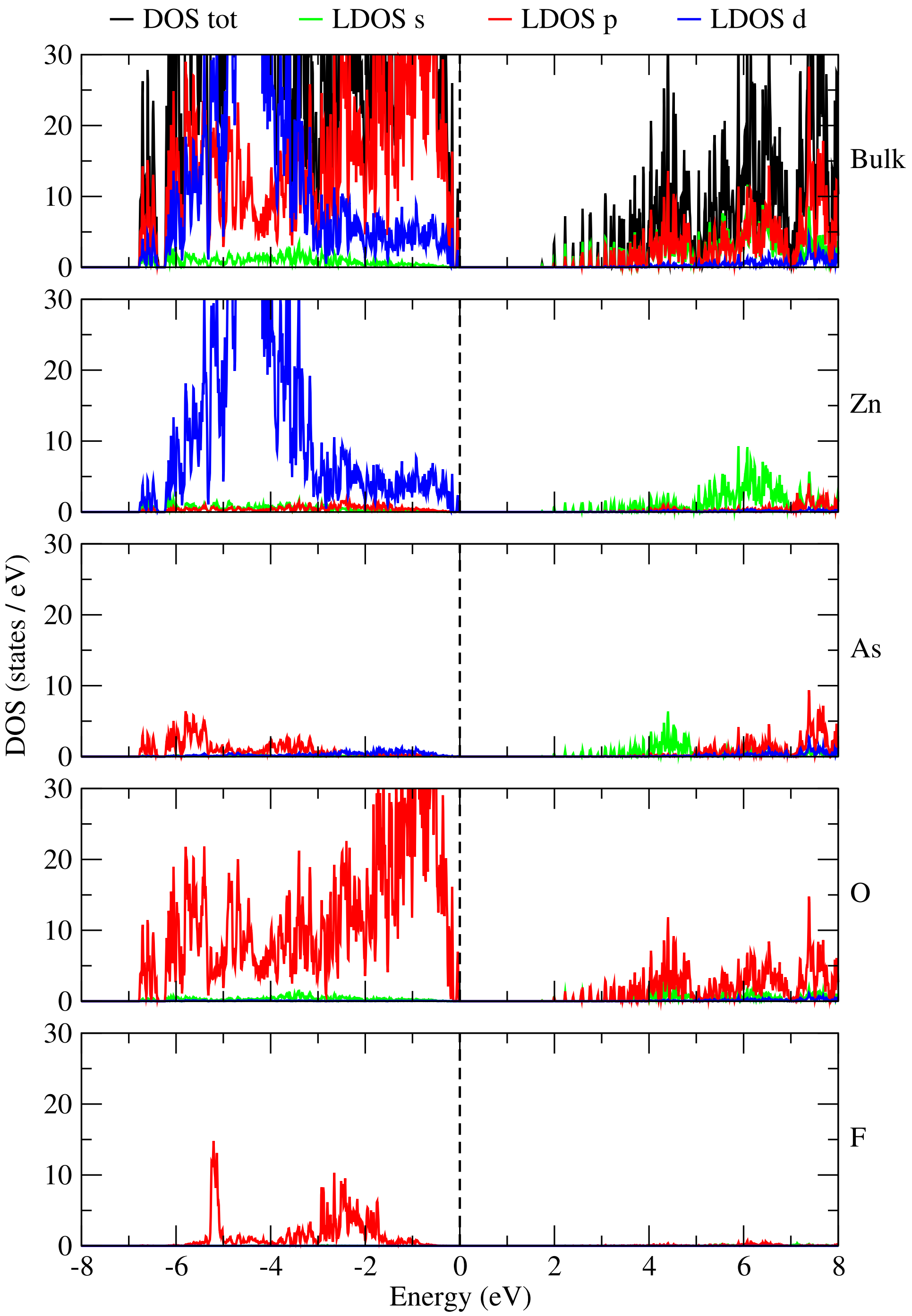

Figure S-129: Local density of states for the $Zn_{10}(AsO_4)_6F_2$ bulk phase.

Table S-132: Calculated properties for the $Zn_{10}(AsO_4)_6F_2$ bulk phase. Number of non-equivalent species, N; average distance for nearest neighbors, d_{NN}; effective coordination number, ECN; and net atomic charge, Q.

Non-equivalent species	N	d_{NN} (Å)	ECN (NNN)	Q (e^-)
Zn(I)	2	2.1275	6.0193	1.099 455
Zn(II)	2	2.1275	6.0193	1.099 455
Zn(III)	6	1.9980	4.7897	1.085 539
As(I)	6	1.6959	3.9864	1.716 527
O(I)	6	1.6959	1.5424	−0.804 995
O(II)	6	1.7341	1.6390	−0.861 067
O(III)	6	1.7367	2.1316	−0.841 965
O(IV)	6	1.7367	2.1316	−0.841 965
F(I)	2	2.1444	3.0380	−0.555 125

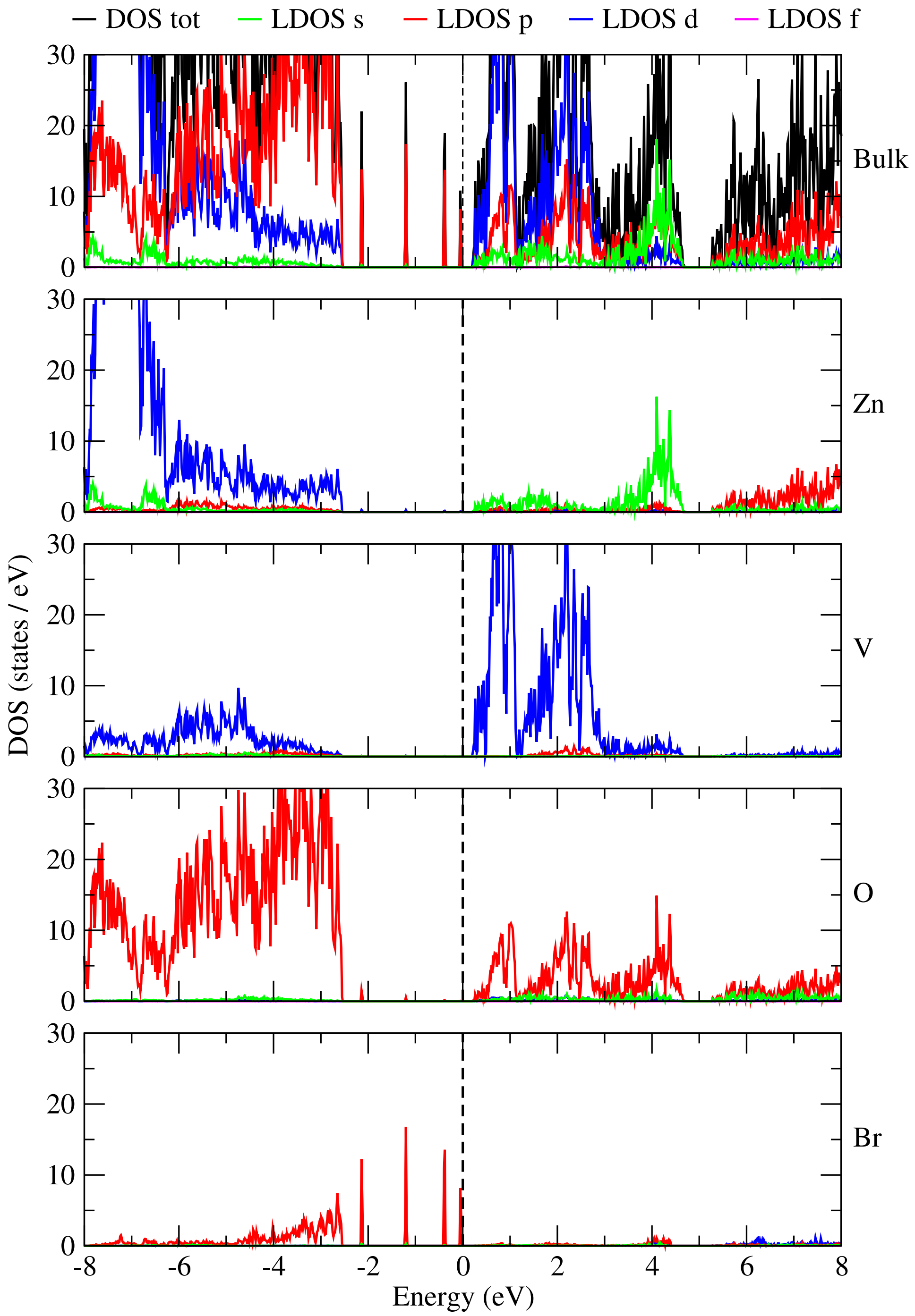

Figure S-130: Local density of states for the $Zn_{10}(VO_4)_6Br_2$ bulk phase.

Table S-133: Calculated properties for the $Zn_{10}(VO_4)_6Br_2$ bulk phase. Number of non-equivalent species, N; average distance for nearest neighbors, d_{NN}; effective coordination number, ECN; and net atomic charge, Q.

Non-equivalent species	N	d_{NN} (Å)	ECN (NNN)	Q (e^-)
Zn(I)	2	2.1509	5.9821	1.130 729
Zn(II)	2	2.1508	5.9818	1.130 698
Zn(III)	6	2.0108	4.3144	1.064 782
V(I)	6	1.6983	3.9301	1.973 232
O(I)	6	1.6983	1.4159	−0.863 947
O(II)	6	1.8000	2.7328	−1.022 084
O(III)	6	1.7114	1.5800	−0.891 454
O(IV)	6	1.7113	1.5814	−0.891 322
Br(I)	2	2.6034	9.1810	−0.369 048

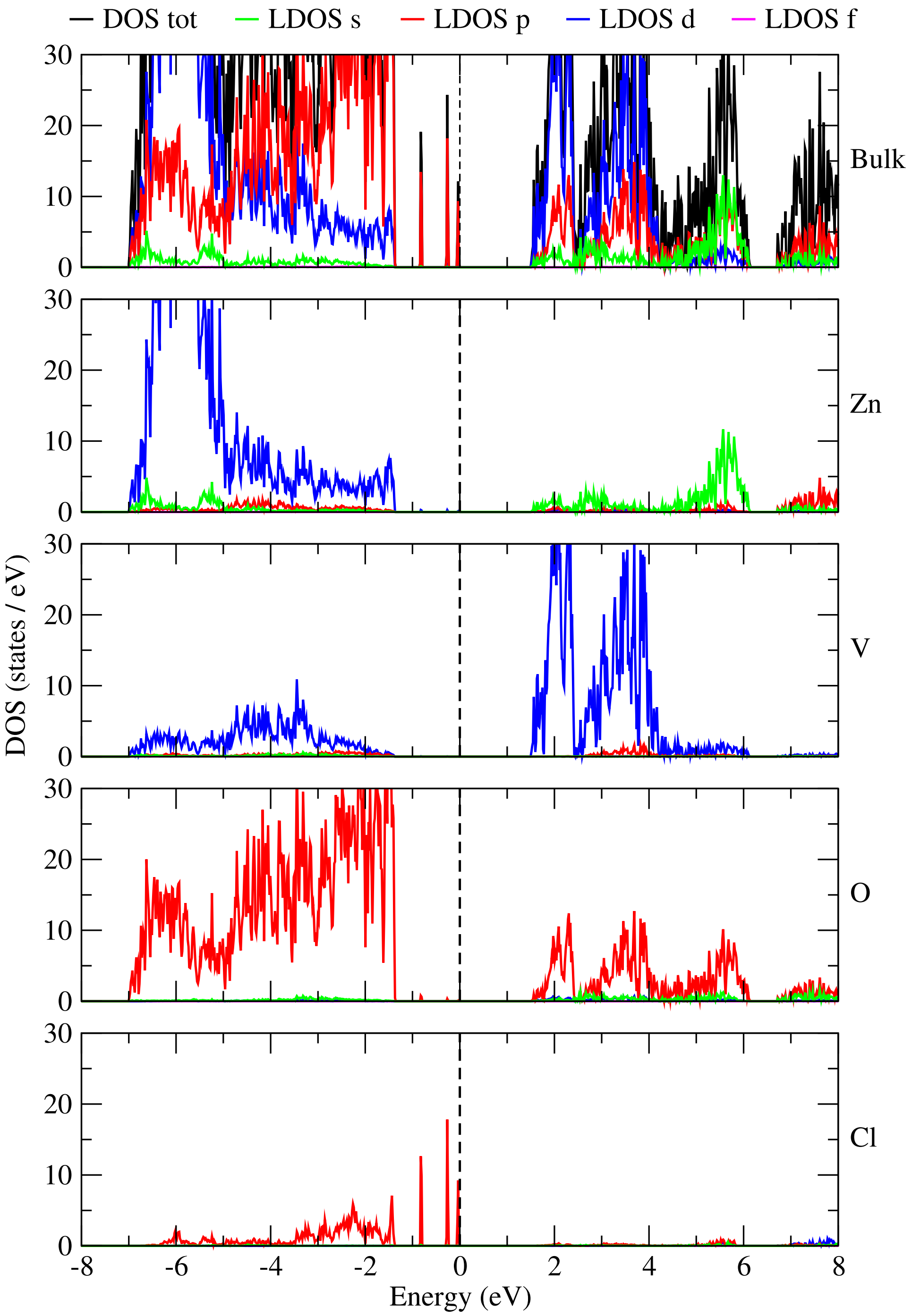

Figure S-131: Local density of states for the $Zn_{10}(VO_4)_6Cl_2$ bulk phase.

Table S-134: Calculated properties for the $Zn_{10}(VO_4)_6Cl_2$ bulk phase. Number of non-equivalent species, N; average distance for nearest neighbors, d_{NN}; effective coordination number, ECN; and net atomic charge, Q.

Non-equivalent species	N	d_{NN} (Å)	ECN (NNN)	Q (e^-)
Zn(I)	2	2.1442	5.9947	1.128 514
Zn(II)	2	2.1426	5.9904	1.128 928
Zn(III)	6	1.9964	4.6072	1.091 190
V(I)	6	1.6960	3.9508	1.980 149
O(I)	6	1.6960	1.4415	−0.861 240
O(II)	6	1.7854	2.3306	−1.020 182
O(III)	6	1.7165	1.6929	−0.899 129
O(IV)	6	1.7164	1.6896	−0.898 326
Cl(I)	2	2.4934	5.7607	−0.434 828

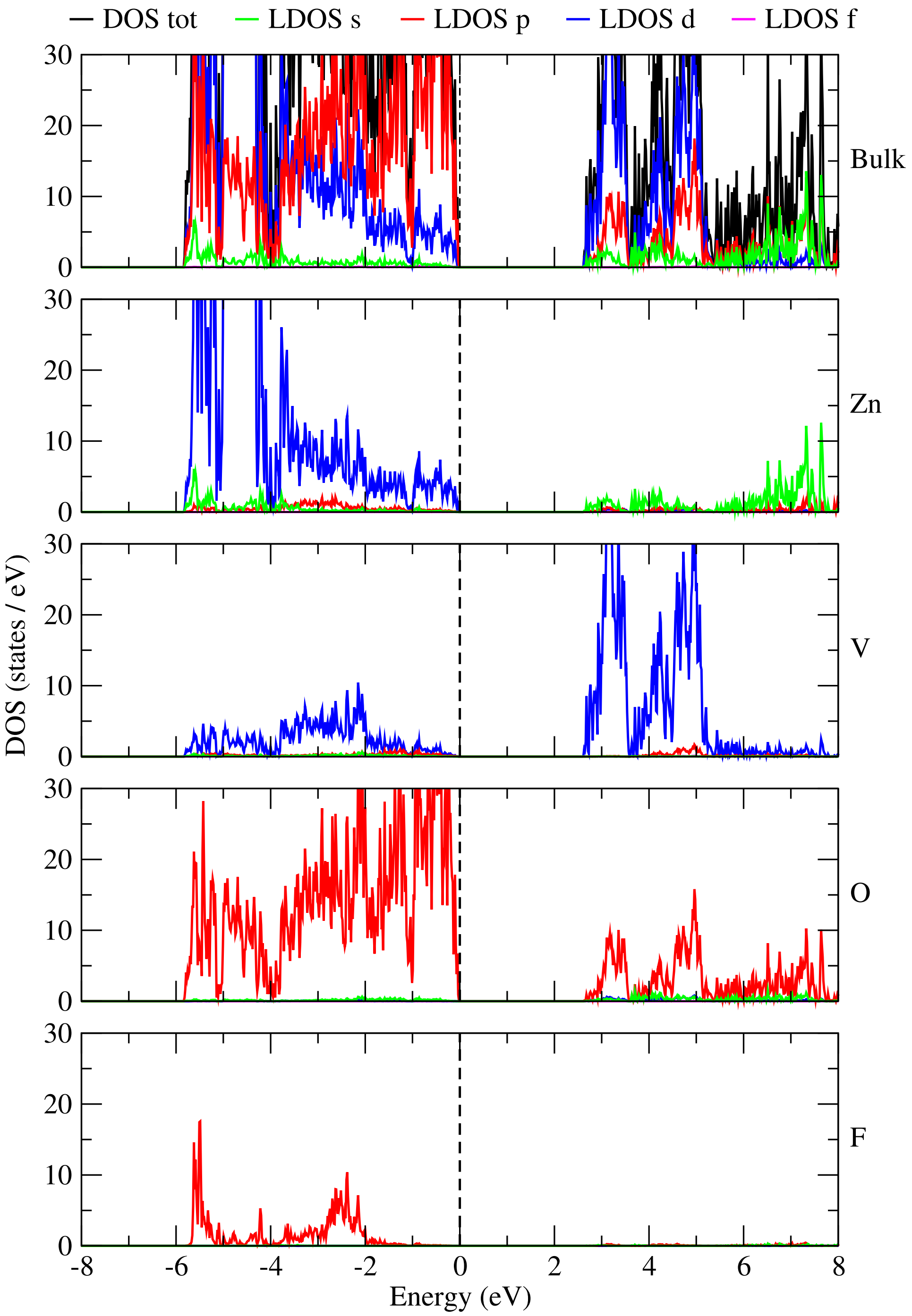

Figure S-132: Local density of states for the $Zn_{10}(VO_4)_6F_2$ bulk phase.

Table S-135: Calculated properties for the $Zn_{10}(VO_4)_6F_2$ bulk phase. Number of non-equivalent species, N; average distance for nearest neighbors, d_{NN}; effective coordination number, ECN; and net atomic charge, Q.

Non-equivalent species	N	d_{NN} (Å)	ECN (NNN)	Q (e^-)
Zn(I)	2	2.1429	6.0357	1.167 674
Zn(II)	2	2.1425	6.0355	1.167 835
Zn(III)	6	1.9571	4.5608	1.124 758
V(I)	6	1.7055	3.9927	2.012 685
O(I)	6	1.7055	1.5184	−0.891 136
O(II)	6	1.7376	1.7072	−0.976 730
O(III)	6	1.7336	2.0825	−0.934 099
O(IV)	6	1.7329	2.0711	−0.933 461
F(I)	2	2.1169	3.0248	−0.541 557

S-6.5 $Ca_4(PO_4)_2O$-based Materials

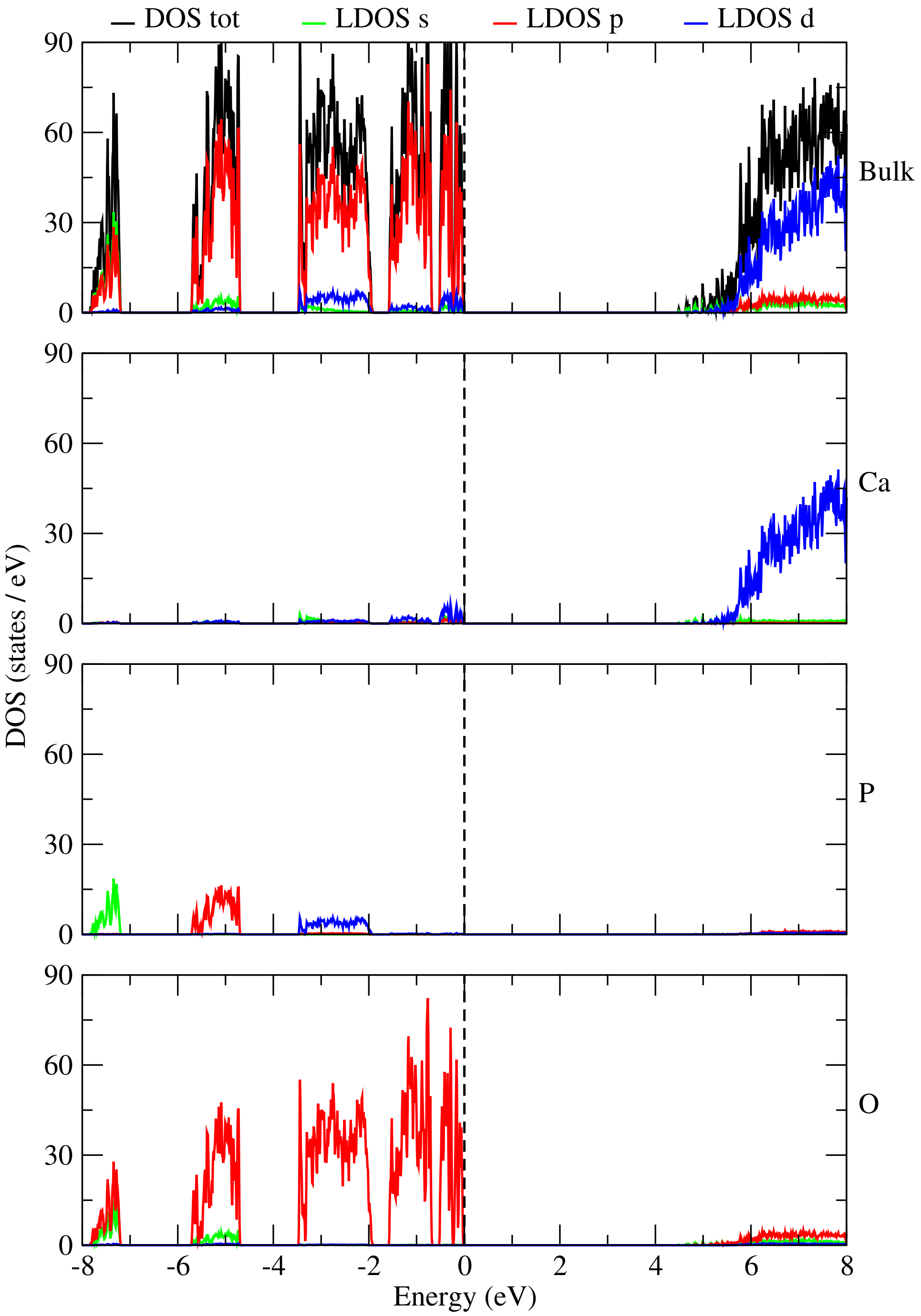

Figure S-133: Local density of states for the $Ca_4(PO_4)_2O$ bulk phase.

Table S-136: Calculated properties for the $Ca_4(PO_4)_2O$ bulk phase. Number of non-equivalent species, N; average distance for nearest neighbors, d_{NN}; effective coordination number, ECN; and net atomic charge, Q.

Non-equivalent species	N	d_{NN} (Å)	ECN (NNN)	Q (e^-)
Ca(I)	2	2.2161	6.1292	1.425 753
Ca(II)	2	2.2507	6.5808	1.419 709
Ca(III)	2	2.1442	5.3500	1.421 624
Ca(IV)	2	2.2939	6.6876	1.460 836
Ca(V)	2	2.2315	5.0880	1.483 982
Ca(VI)	2	2.2061	6.1192	1.404 750
Ca(VII)	2	2.1781	6.3086	1.417 435
Ca(VIII)	2	2.2164	6.1990	1.437 939
P(I)	2	1.5537	3.9957	1.470 343
P(II)	2	1.5413	3.9876	1.515 304
P(III)	2	1.5456	3.9946	1.460 363
P(IV)	2	1.5530	3.9980	1.496 459
O(I)	2	1.5764	1.0001	−0.936 635
O(II)	2	1.5537	1.0000	−0.903 225
O(III)	2	1.5560	1.0000	−0.907 056
O(IV)	2	1.5580	1.0000	−0.924 942
O(V)	2	1.5768	1.0001	−0.949 847
O(VI)	2	1.5631	1.0000	−0.909 142
O(VII)	2	1.5793	1.0001	−0.938 743
O(VIII)	2	1.5413	1.0000	−0.890 224
O(IX)	2	1.5646	1.0001	−0.936 855
O(X)	2	1.5456	1.0000	−0.907 550
O(XI)	2	1.5492	1.0002	−0.915 819
O(XII)	2	1.5692	1.0000	−0.929 546
O(XIII)	2	1.5627	1.0000	−0.917 072
O(XIV)	2	1.5701	1.0000	−0.942 473
O(XV)	2	1.5595	1.0000	−0.923 501
O(XVI)	2	1.5530	1.0000	−0.915 287
O(XVII)	2	2.2061	4.0196	−1.328 152
O(XVIII)	2	2.1442	4.0062	−1.338 428

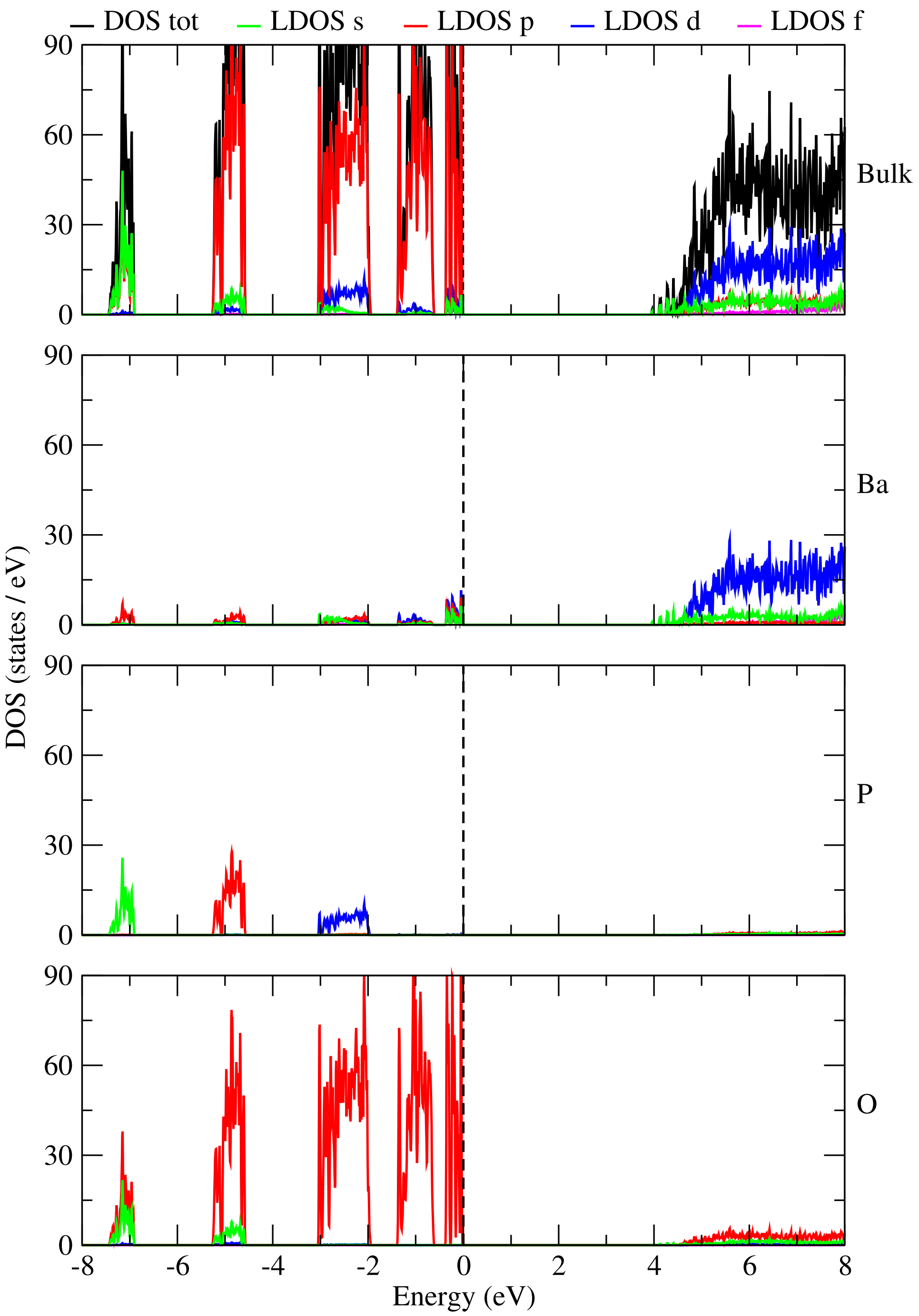

Figure S-134: Local density of states for the $Ba_4(PO_4)_2O$ bulk phase.

Table S-137: Calculated properties for the $Ba_4(PO_4)_2O$ bulk phase. Number of non-equivalent species, N; average distance for nearest neighbors, d_{NN}; effective coordination number, ECN; and net atomic charge, Q.

Non-equivalent species	N	d_{NN} (Å)	ECN (NNN)	Q (e^-)
Ba(I)	2	2.4799	6.3971	1.444 863
Ba(II)	2	2.5097	6.7742	1.440 477
Ba(III)	2	2.4252	6.3053	1.430 418
Ba(IV)	2	2.5939	6.8399	1.503 943
Ba(V)	2	2.5284	5.9733	1.509 972
Ba(VI)	2	2.4770	7.2352	1.428 560
Ba(VII)	2	2.4836	6.5499	1.452 386
Ba(VIII)	2	2.4926	6.2782	1.469 317
P(I)	2	1.5630	3.9991	1.479 403
P(II)	2	1.5538	3.9929	1.516 498
P(III)	2	1.5568	3.9975	1.475 077
P(IV)	2	1.5644	3.9973	1.496 736
O(I)	2	1.5735	1.0000	−0.954 304
O(II)	2	1.5630	1.0000	−0.925 091
O(III)	2	1.5678	1.0000	−0.929 025
O(IV)	2	1.5638	1.0000	−0.946 932
O(V)	2	1.5806	1.0000	−0.947 712
O(VI)	2	1.5739	1.0000	−0.934 620
O(VII)	2	1.5828	1.0000	−0.951 652
O(VIII)	2	1.5538	1.0000	−0.920 785
O(IX)	2	1.5630	1.0000	−0.946 993
O(X)	2	1.5568	1.0000	−0.926 333
O(XI)	2	1.5604	1.0000	−0.929 432
O(XII)	2	1.5751	1.0000	−0.974 553
O(XIII)	2	1.5689	1.0000	−0.933 919
O(XIV)	2	1.5825	1.0000	−0.961 822
O(XV)	2	1.5665	1.0000	−0.934 210
O(XVI)	2	1.5644	1.0000	−0.932 946
O(XVII)	2	2.4770	4.0021	−1.296 748
O(XVIII)	2	2.4252	3.9991	−1.300 575

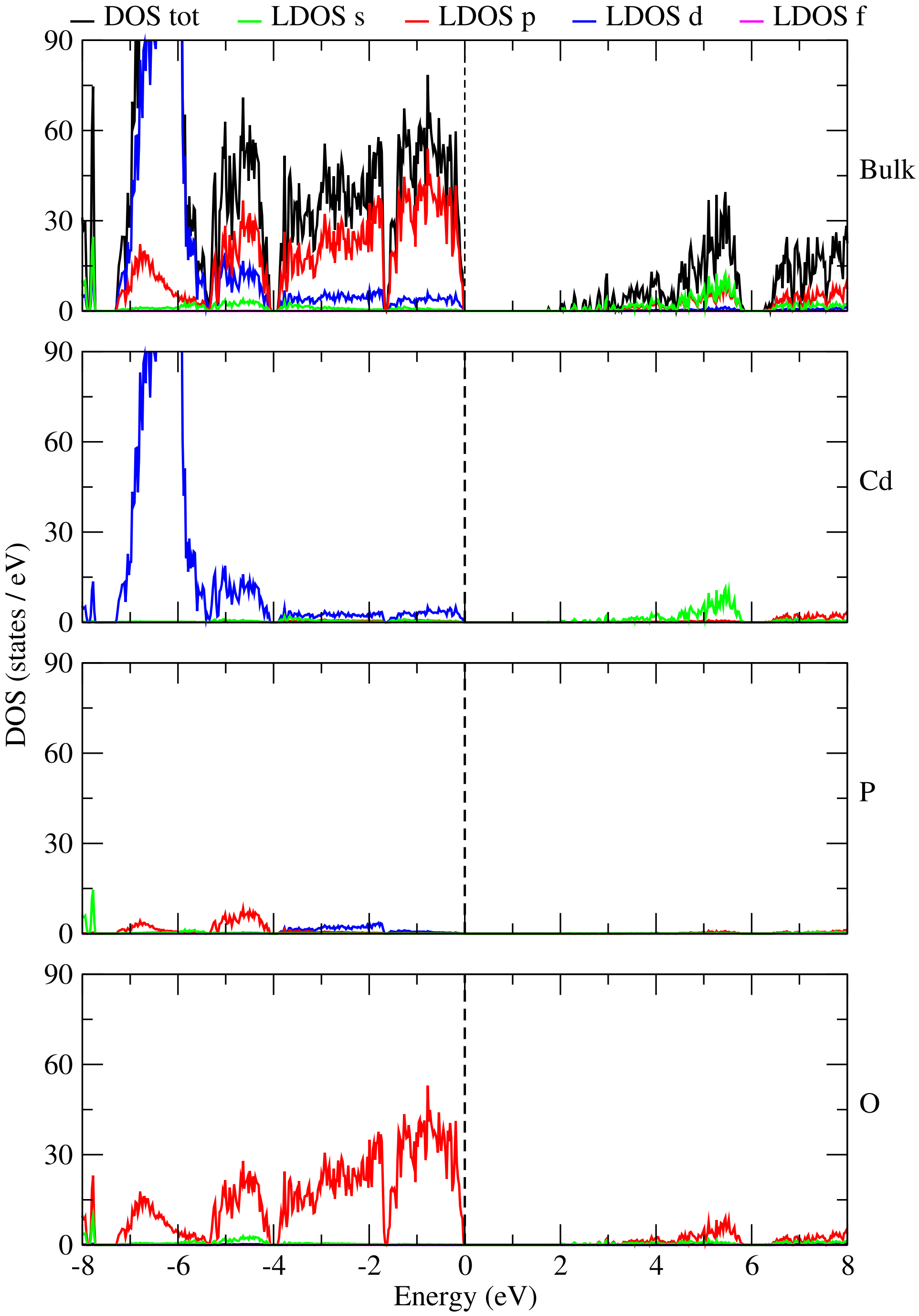

Figure S-135: Local density of states for the $Cd_4(PO_4)_2O$ bulk phase.

Table S-138: Calculated properties for the $Cd_4(PO_4)_2O$ bulk phase. Number of non-equivalent species, N; average distance for nearest neighbors, d_{NN}; effective coordination number, ECN; and net atomic charge, Q.

Non-equivalent species	N	d_{NN} (Å)	ECN (NNN)	Q (e^-)
Cd(I)	2	2.2066	5.8165	1.152 692
Cd(II)	2	2.2118	5.7020	1.121 496
Cd(III)	2	2.1284	4.5167	1.138 581
Cd(IV)	2	2.2839	6.1398	1.205 716
Cd(V)	2	2.2358	4.9895	1.209 069
Cd(VI)	2	2.1936	5.2005	1.106 252
Cd(VII)	2	2.1298	5.8229	1.120 144
Cd(VIII)	2	2.1468	5.1394	1.132 633
P(I)	2	1.5442	3.9719	1.456 449
P(II)	2	1.5456	3.9785	1.487 021
P(III)	2	1.5290	3.9677	1.456 331
P(IV)	2	1.5548	3.9961	1.491 776
O(I)	2	1.6020	1.0031	−0.811 422
O(II)	2	1.5512	1.0001	−0.811 915
O(III)	2	1.5523	1.0002	−0.809 054
O(IV)	2	1.5442	1.0001	−0.802 151
O(V)	2	1.5942	1.0014	−0.849 730
O(VI)	2	1.5550	1.0000	−0.823 136
O(VII)	2	1.5840	1.0001	−0.830 593
O(VIII)	2	1.5456	1.0000	−0.815 138
O(IX)	2	1.5965	1.0036	−0.817 196
O(X)	2	1.5290	1.0005	−0.802 723
O(XI)	2	1.5580	1.0005	−0.825 332
O(XII)	2	1.5718	1.0000	−0.810 291
O(XIII)	2	1.5786	1.0004	−0.824 234
O(XIV)	2	1.5631	1.0001	−0.821 169
O(XV)	2	1.5672	1.0001	−0.833 231
O(XVI)	2	1.5548	1.0008	−0.814 251
O(XVII)	2	2.1936	3.9756	−1.008 489
O(XVIII)	2	2.1284	4.0131	−0.968 105

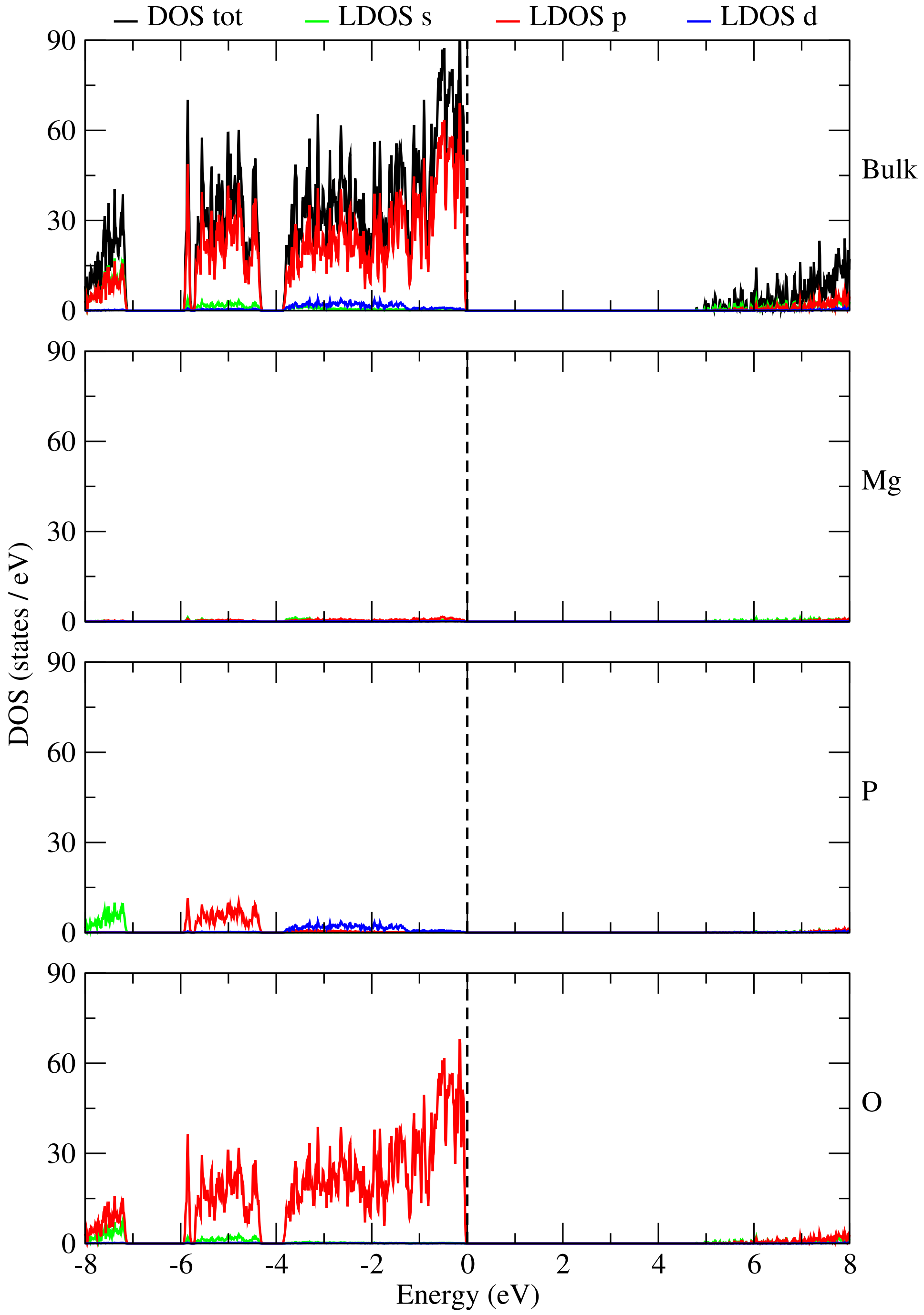

Figure S-136: Local density of states for the $Mg_4(PO_4)_2O$ bulk phase.

Table S-139: Calculated properties for the $Mg_4(PO_4)_2O$ bulk phase. Number of non-equivalent species, N; average distance for nearest neighbors, d_{NN}; effective coordination number, ECN; and net atomic charge, Q.

Non-equivalent species	N	d_{NN} (Å)	ECN (NNN)	Q (e^-)
Mg(I)	2	1.9944	5.3719	1.466 657
Mg(II)	2	1.9493	4.3947	1.472 469
Mg(III)	2	2.0000	5.7446	1.459 668
Mg(IV)	2	2.0125	5.7367	1.485 126
Mg(V)	2	2.0116	5.7348	1.485 008
Mg(VI)	2	2.0000	5.7428	1.459 368
Mg(VII)	2	1.9947	5.3627	1.466 435
Mg(VIII)	2	1.9485	4.3852	1.472 453
P(I)	2	1.5356	3.9832	1.616 072
P(II)	2	1.5376	3.9797	1.573 290
P(III)	2	1.5356	3.9831	1.615 849
P(IV)	2	1.5377	3.9797	1.573 459
O(I)	2	1.5537	1.0931	−0.938 452
O(II)	2	1.5741	1.0168	−0.947 862
O(III)	2	1.5356	1.0642	−0.928 872
O(IV)	2	1.5800	1.1981	−1.012 930
O(V)	2	1.5620	1.1064	−0.954 085
O(VI)	2	1.5376	1.0235	−0.916 277
O(VII)	2	1.5910	1.0262	−0.997 050
O(VIII)	2	1.5528	1.0190	−0.935 134
O(IX)	2	1.5801	1.1963	−1.013 022
O(X)	2	1.5356	1.0652	−0.928 905
O(XI)	2	1.5744	1.0172	−0.947 995
O(XII)	2	1.5540	1.0926	−0.938 191
O(XIII)	2	1.5522	1.0189	−0.934 910
O(XIV)	2	1.5909	1.0267	−0.996 531
O(XV)	2	1.5620	1.1083	−0.954 380
O(XVI)	2	1.5377	1.0227	−0.916 322
O(XVII)	2	1.9493	3.8796	−1.442 468
O(XVIII)	2	1.9485	3.8793	−1.442 469

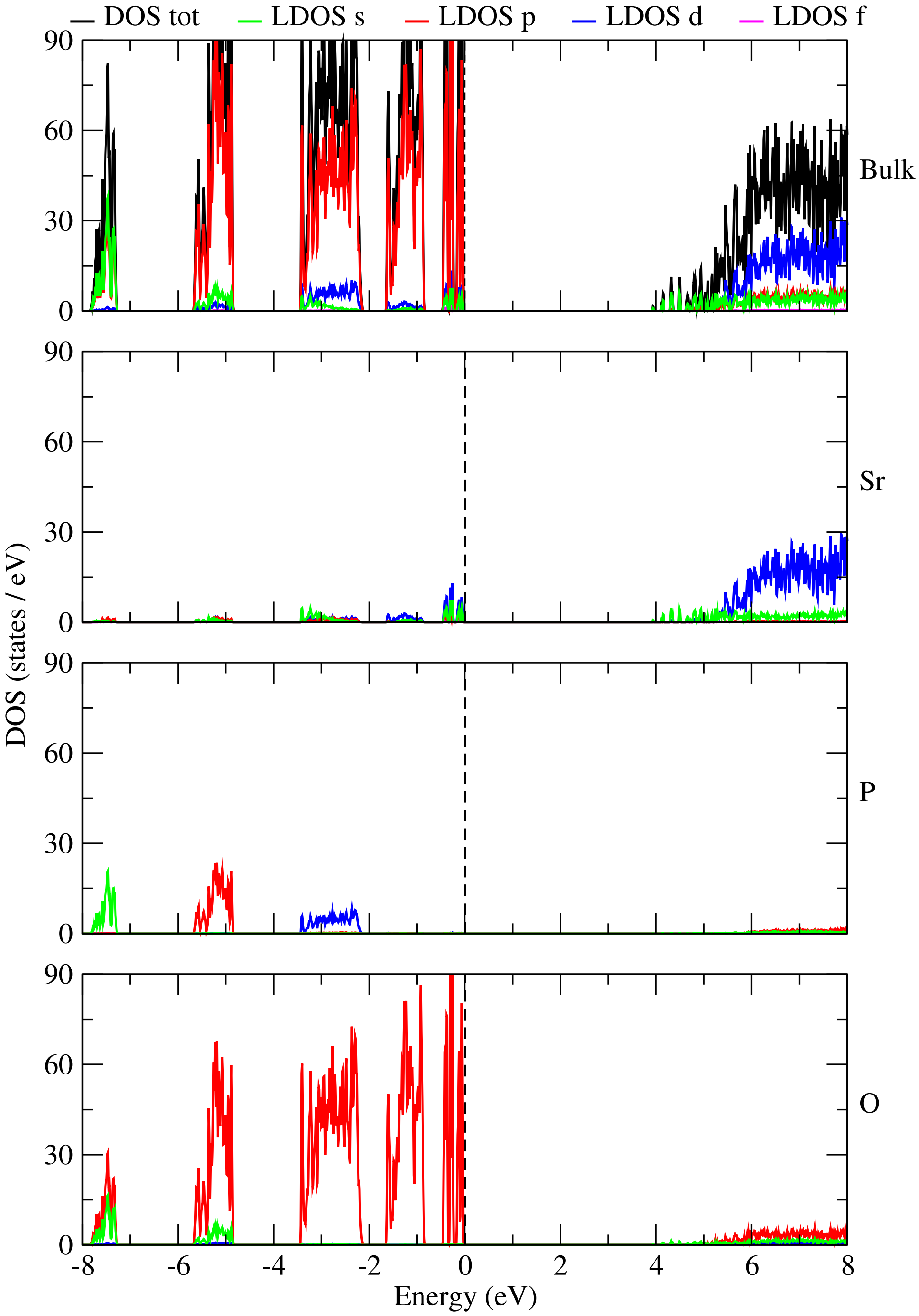

Figure S-137: Local density of states for the $Sr_4(PO_4)_2O$ bulk phase.

Table S-140: Calculated properties for the $Sr_4(PO_4)_2O$ bulk phase. Number of non-equivalent species, N; average distance for nearest neighbors, d_{NN}; effective coordination number, ECN; and net atomic charge, Q.

Non-equivalent species	N	d_{NN} (Å)	ECN (NNN)	Q (e^-)
Sr(I)	2	2.3490	6.3848	1.461 872
Sr(II)	2	2.3827	6.7684	1.461 607
Sr(III)	2	2.2914	6.0700	1.453 690
Sr(IV)	2	2.4273	6.6969	1.508 283
Sr(V)	2	2.3787	5.5588	1.525 011
Sr(VI)	2	2.3436	6.9144	1.445 712
Sr(VII)	2	2.3341	6.4979	1.464 959
Sr(VIII)	2	2.3575	6.2874	1.482 569
P(I)	2	1.5579	3.9980	1.473 069
P(II)	2	1.5473	3.9905	1.515 473
P(III)	2	1.5512	3.9951	1.466 479
P(IV)	2	1.5603	3.9976	1.493 632
O(I)	2	1.5746	1.0000	−0.954 087
O(II)	2	1.5579	1.0000	−0.920 630
O(III)	2	1.5632	1.0000	−0.925 851
O(IV)	2	1.5630	1.0000	−0.943 388
O(V)	2	1.5783	1.0000	−0.959 015
O(VI)	2	1.5703	1.0000	−0.929 987
O(VII)	2	1.5808	1.0000	−0.953 218
O(VIII)	2	1.5473	1.0000	−0.913 046
O(IX)	2	1.5628	1.0000	−0.949 423
O(X)	2	1.5512	1.0000	−0.928 667
O(XI)	2	1.5533	1.0000	−0.927 897
O(XII)	2	1.5753	1.0000	−0.960 578
O(XIII)	2	1.5651	1.0000	−0.934 667
O(XIV)	2	1.5777	1.0000	−0.960 119
O(XV)	2	1.5627	1.0000	−0.934 865
O(XVI)	2	1.5603	1.0000	−0.930 617
O(XVII)	2	2.3436	4.0248	−1.357 679
O(XVIII)	2	2.2914	4.0085	−1.368 621

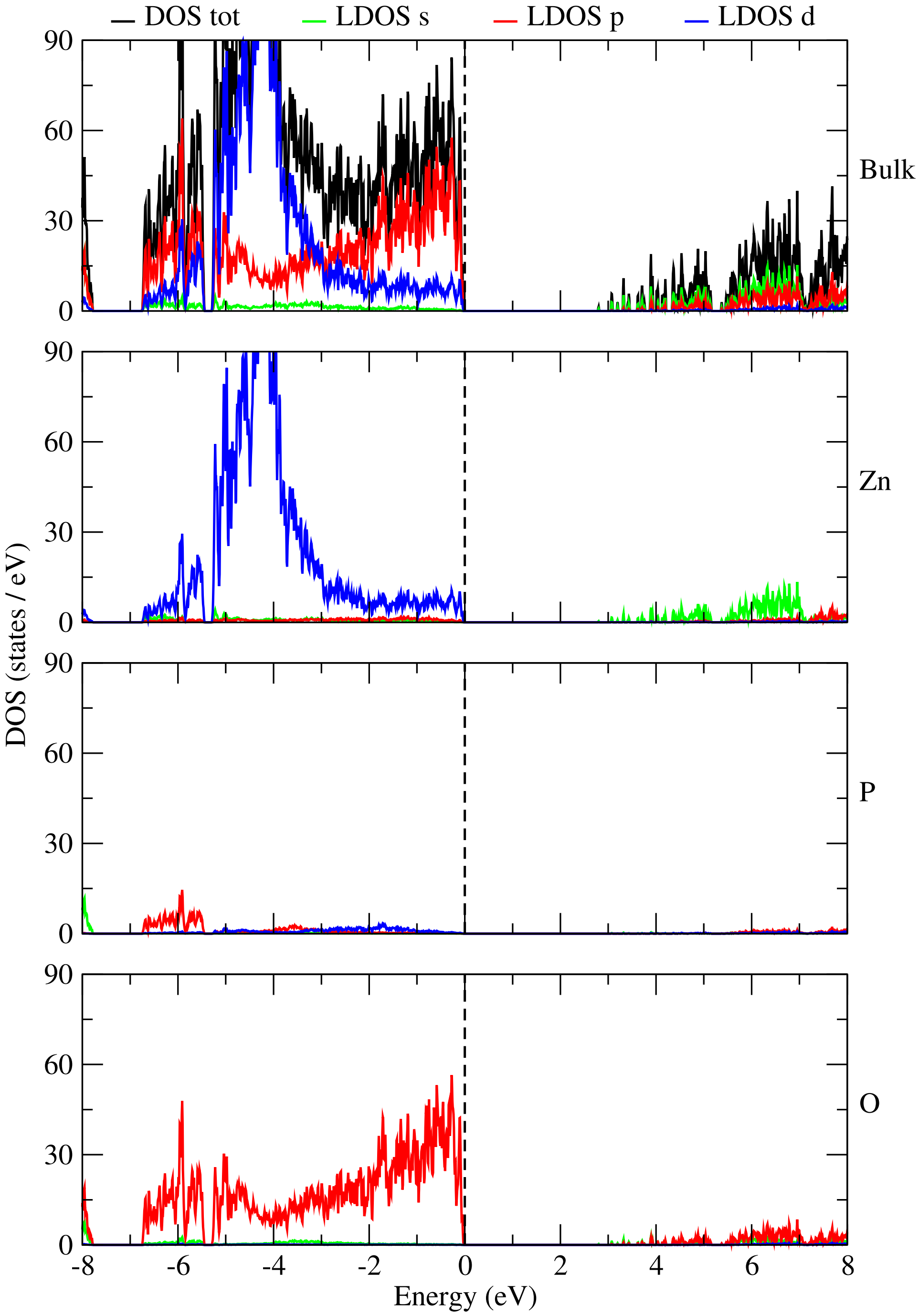

Figure S-138: Local density of states for the $Zn_4(PO_4)_2O$ bulk phase.

Table S-141: Calculated properties for the $Zn_4(PO_4)_2O$ bulk phase. Number of non-equivalent species, N; average distance for nearest neighbors, d_{NN}; effective coordination number, ECN; and net atomic charge, Q.

Non-equivalent species	N	d_{NN} (Å)	ECN (NNN)	Q (e^-)
Zn(I)	2	1.9283	3.9515	1.053 696
Zn(II)	2	1.9442	3.9444	1.062 282
Zn(III)	2	2.0249	5.0629	1.088 575
Zn(IV)	2	2.0629	4.9733	1.108 075
Zn(V)	2	2.0637	4.9753	1.107 970
Zn(VI)	2	2.0258	5.0685	1.088 670
Zn(VII)	2	1.9278	3.9527	1.053 939
Zn(VIII)	2	1.9437	3.9444	1.062 194
P(I)	2	1.5435	3.9916	1.562 027
P(II)	2	1.5206	3.9630	1.538 352
P(III)	2	1.5434	3.9918	1.561 390
P(IV)	2	1.5204	3.9626	1.538 097
O(I)	2	1.5435	1.2034	−0.796 471
O(II)	2	1.5658	1.0548	−0.793 749
O(III)	2	1.5612	1.0821	−0.785 208
O(IV)	2	1.5782	1.1696	−0.824 617
O(V)	2	1.5863	1.2218	−0.798 685
O(VI)	2	1.5206	1.0286	−0.801 402
O(VII)	2	1.5813	1.1322	−0.815 954
O(VIII)	2	1.5602	1.0452	−0.799 656
O(IX)	2	1.5775	1.1664	−0.823 921
O(X)	2	1.5617	1.0828	−0.785 024
O(XI)	2	1.5663	1.0548	−0.794 027
O(XII)	2	1.5434	1.2020	−0.796 693
O(XIII)	2	1.5604	1.0443	−0.799 840
O(XIV)	2	1.5810	1.1312	−0.815 656
O(XV)	2	1.5867	1.2246	−0.798 590
O(XVI)	2	1.5204	1.0285	−0.801 211
O(XVII)	2	1.9442	3.8981	−0.997 294
O(XVIII)	2	1.9437	3.8984	−0.997 269

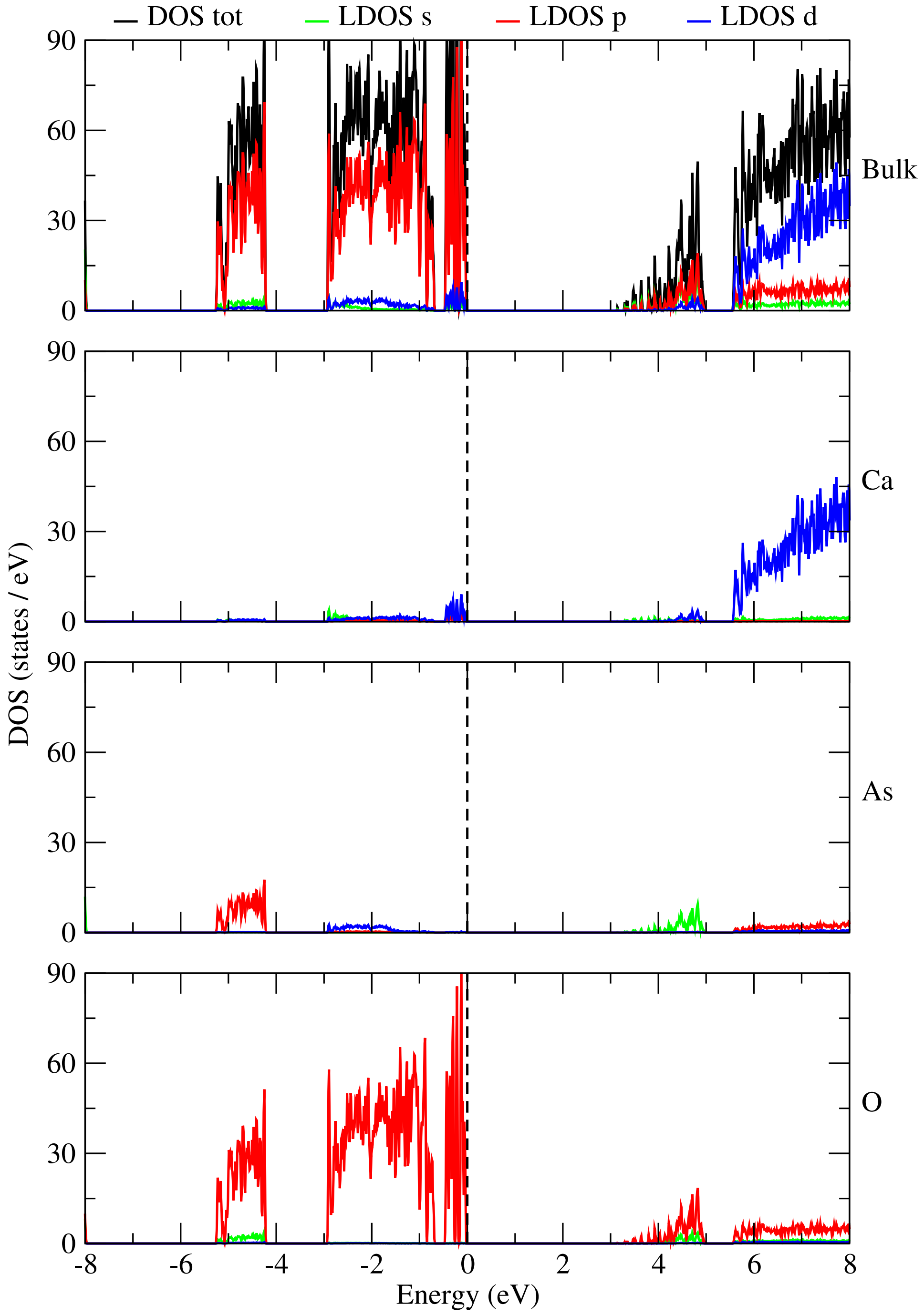

Figure S-139: Local density of states for the $Ca_4(AsO_4)_2O$ bulk phase.

Table S-142: Calculated properties for the $Ca_4(AsO_4)_2O$ bulk phase. Number of non-equivalent species, N; average distance for nearest neighbors, d_{NN}; effective coordination number, ECN; and net atomic charge, Q.

Non-equivalent species	N	d_{NN} (Å)	ECN (NNN)	Q (e^-)
Ca(I)	2	2.2500	5.8570	1.416 533
Ca(II)	2	2.2715	6.0865	1.398 400
Ca(III)	2	2.1624	4.7653	1.403 228
Ca(IV)	2	2.3348	6.5328	1.436 212
Ca(V)	2	2.2334	5.2397	1.441 866
Ca(VI)	2	2.2280	5.2773	1.376 545
Ca(VII)	2	2.1777	5.9506	1.379 684
Ca(VIII)	2	2.2361	6.2927	1.406 815
As(I)	2	1.7041	3.9928	1.636 273
As(II)	2	1.7006	3.9880	1.649 743
As(III)	2	1.7053	3.9971	1.594 369
As(IV)	2	1.7050	3.9967	1.647 972
O(I)	2	1.7389	1.0092	−0.962 231
O(II)	2	1.7152	1.0008	−0.918 917
O(III)	2	1.7041	1.0128	−0.930 737
O(IV)	2	1.7154	1.0209	−0.963 368
O(V)	2	1.7365	1.0118	−0.981 398
O(VI)	2	1.7154	1.0034	−0.928 953
O(VII)	2	1.7412	1.0164	−0.974 239
O(VIII)	2	1.7006	1.0019	−0.905 547
O(IX)	2	1.7259	1.0150	−0.973 649
O(X)	2	1.7053	1.0048	−0.916 779
O(XI)	2	1.7119	1.0161	−0.946 141
O(XII)	2	1.7212	1.0006	−0.940 665
O(XIII)	2	1.7219	1.0048	−0.940 608
O(XIV)	2	1.7284	1.0061	−0.955 477
O(XV)	2	1.7160	1.0107	−0.949 515
O(XVI)	2	1.7050	1.0161	−0.955 294
O(XVII)	2	2.2280	4.0264	−1.326 641
O(XVIII)	2	2.1624	4.0354	−1.317 483

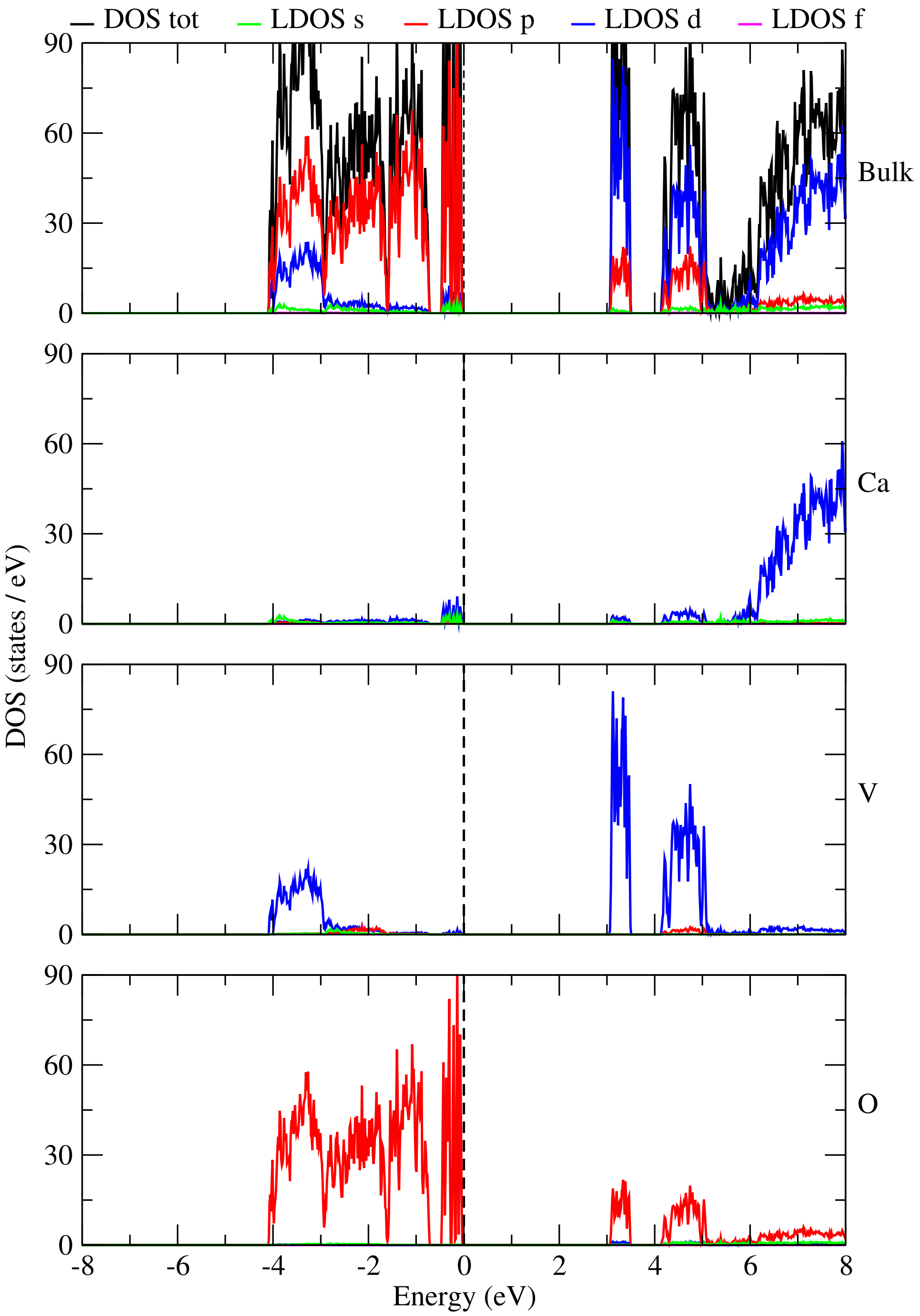

Figure S-140: Local density of states for the $Ca_4(VO_4)_2O$ bulk phase.

Table S-143: Calculated properties for the $Ca_4(VO_4)_2O$ bulk phase. Number of non-equivalent species, N; average distance for nearest neighbors, d_{NN}; effective coordination number, ECN; and net atomic charge, Q.

Non-equivalent species	N	d_{NN} (Å)	ECN (NNN)	Q (e^-)
Ca(I)	2	2.2534	5.8220	1.458 364
Ca(II)	2	2.2802	6.1188	1.420 620
Ca(III)	2	2.1765	5.0651	1.437 434
Ca(IV)	2	2.3252	6.2838	1.479 449
Ca(V)	2	2.2387	5.1566	1.486 431
Ca(VI)	2	2.2265	4.9115	1.402 101
Ca(VII)	2	2.1818	5.7954	1.414 128
Ca(VIII)	2	2.2422	5.9870	1.450 224
V(I)	2	1.6903	3.9839	1.959 470
V(II)	2	1.6993	3.9858	1.960 681
V(III)	2	1.6949	3.9836	1.918 515
V(IV)	2	1.7068	3.9960	1.970 268
O(I)	2	1.7432	1.0187	−1.068 621
O(II)	2	1.7238	1.0021	−1.034 079
O(III)	2	1.6903	1.0108	−0.982 271
O(IV)	2	1.7180	1.0286	−1.063 199
O(V)	2	1.7360	1.0099	−1.079 137
O(VI)	2	1.7193	1.0034	−1.025 095
O(VII)	2	1.7464	1.0260	−1.088 713
O(VIII)	2	1.6993	1.0016	−0.995 902
O(IX)	2	1.7435	1.0224	−1.126 422
O(X)	2	1.6949	1.0098	−0.994 100
O(XI)	2	1.7025	1.0201	−1.029 968
O(XII)	2	1.7254	1.0007	−1.041 077
O(XIII)	2	1.7335	1.0095	−1.054 956
O(XIV)	2	1.7192	1.0061	−1.024 003
O(XV)	2	1.7189	1.0209	−1.060 880
O(XVI)	2	1.7068	1.0213	−1.056 021
O(XVII)	2	2.2265	4.0402	−1.324 143
O(XVIII)	2	2.1765	4.0383	−1.309 100

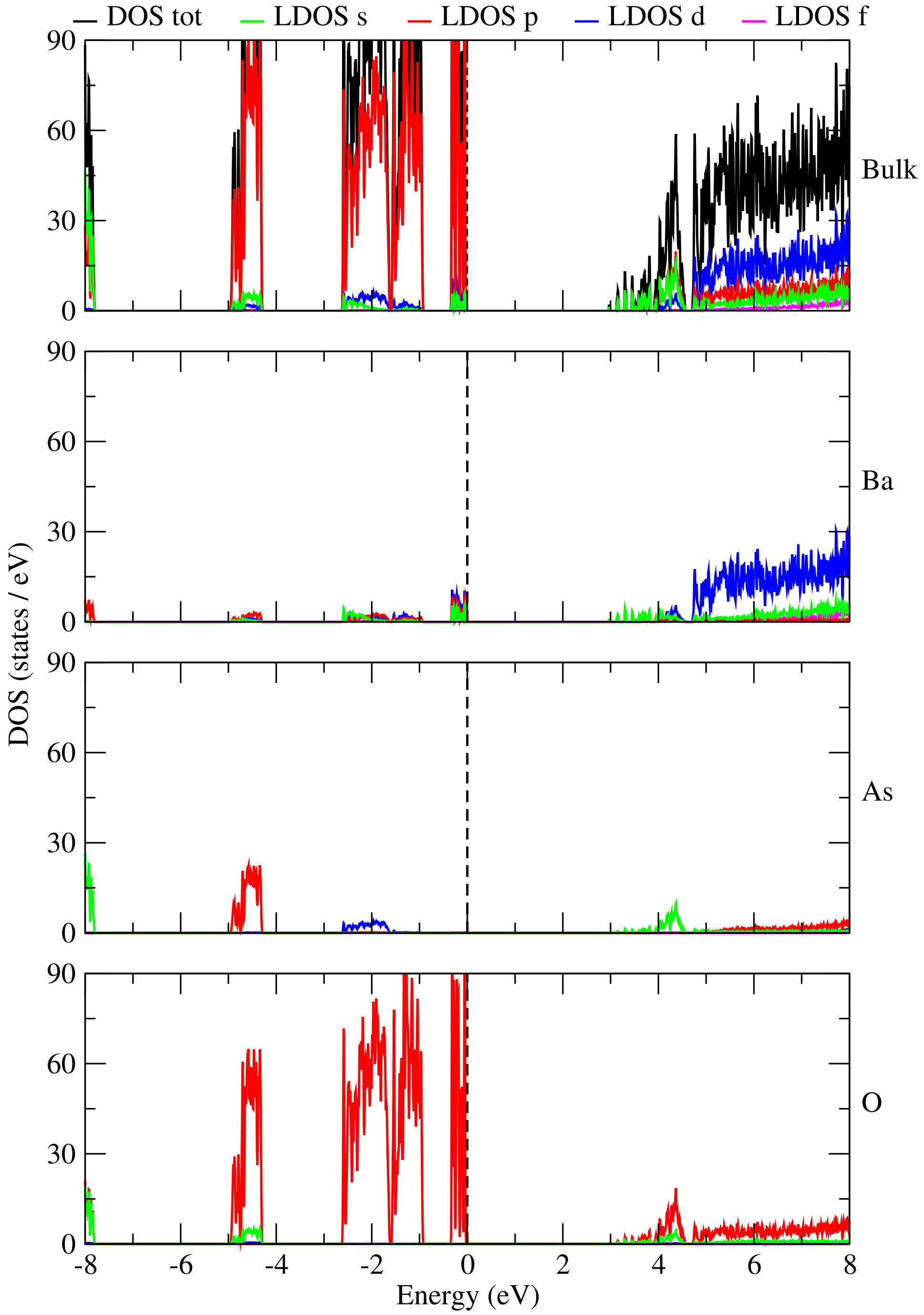

Figure S-141: Local density of states for the $Ba_4(AsO_4)_2O$ bulk phase.

Table S-144: Calculated properties for the $Ba_4(AsO_4)_2O$ bulk phase. Number of non-equivalent species, N; average distance for nearest neighbors, d_{NN}; effective coordination number, ECN; and net atomic charge, Q.

Non-equivalent species	N	d_{NN} (Å)	ECN (NNN)	Q (e^-)
Ba(I)	2	2.5050	6.2924	1.435 874
Ba(II)	2	2.5324	6.5999	1.421 614
Ba(III)	2	2.4324	5.8203	1.405 079
Ba(IV)	2	2.6247	6.7758	1.480 446
Ba(V)	2	2.5369	5.6073	1.476 260
Ba(VI)	2	2.4940	6.4309	1.395 048
Ba(VII)	2	2.4888	6.4317	1.412 323
Ba(VIII)	2	2.5213	6.4544	1.439 386
As(I)	2	1.7198	3.9987	1.616 863
As(II)	2	1.7101	3.9930	1.646 767
As(III)	2	1.7158	3.9986	1.613 594
As(IV)	2	1.7214	3.9983	1.631 687
O(I)	2	1.7341	1.0000	−0.966 660
O(II)	2	1.7198	1.0000	−0.939 526
O(III)	2	1.7252	1.0000	−0.949 132
O(IV)	2	1.7229	1.0000	−0.974 056
O(V)	2	1.7399	1.0000	−0.978 658
O(VI)	2	1.7318	1.0000	−0.956 688
O(VII)	2	1.7416	1.0000	−0.973 569
O(VIII)	2	1.7101	1.0000	−0.936 103
O(IX)	2	1.7230	1.0000	−0.978 504
O(X)	2	1.7158	1.0000	−0.949 719
O(XI)	2	1.7170	1.0000	−0.952 659
O(XII)	2	1.7300	1.0000	−0.982 625
O(XIII)	2	1.7264	1.0000	−0.958 033
O(XIV)	2	1.7382	1.0000	−0.974 025
O(XV)	2	1.7255	1.0000	−0.960 201
O(XVI)	2	1.7214	1.0000	−0.958 472
O(XVII)	2	2.4940	4.0279	−1.300 840
O(XVIII)	2	2.4324	4.0199	−1.285 473

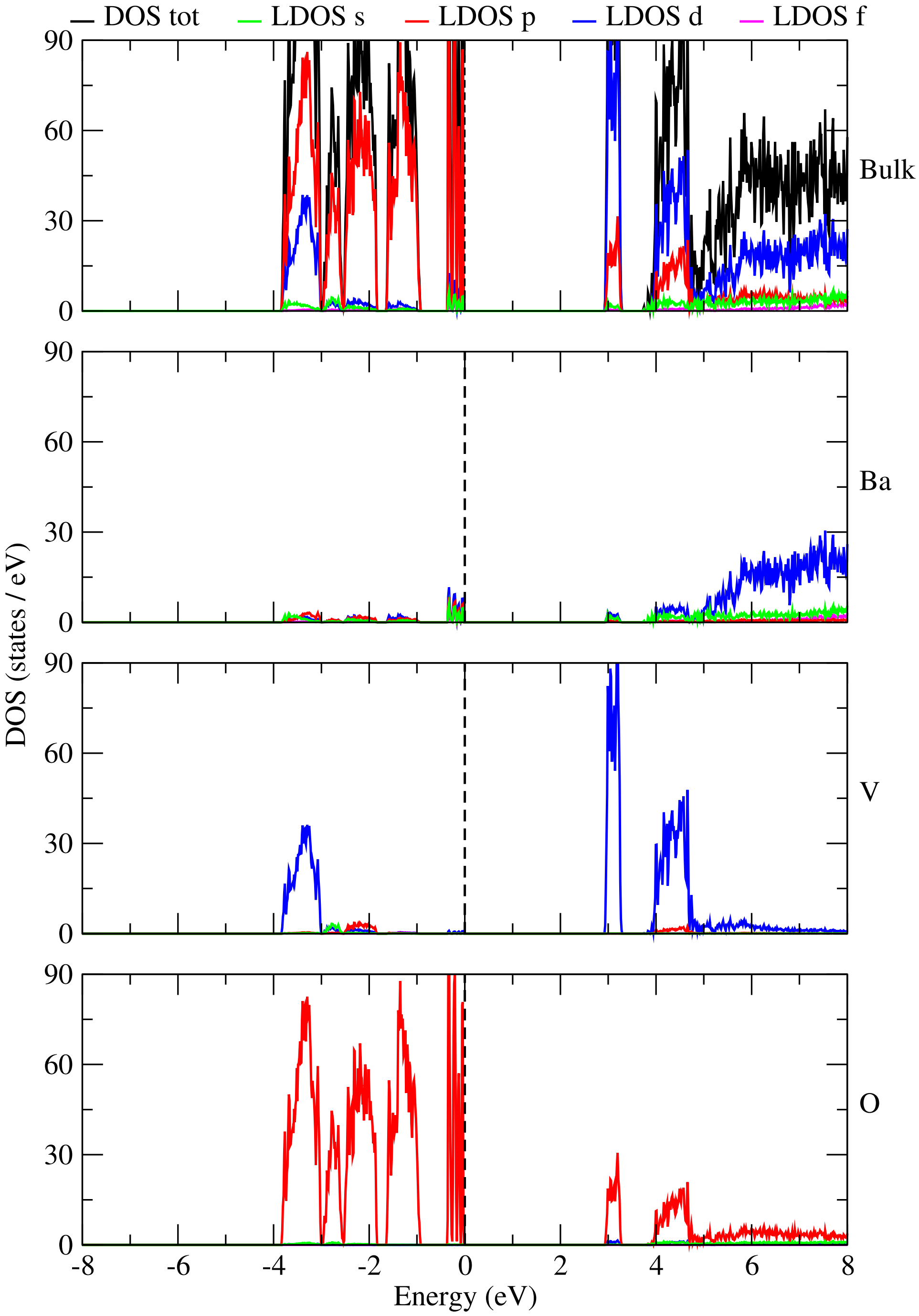

Figure S-142: Local density of states for the $Ba_4(VO_4)_2O$ bulk phase.

Table S-145: Calculated properties for the $Ba_4(VO_4)_2O$ bulk phase. Number of non-equivalent species, N; average distance for nearest neighbors, d_{NN}; effective coordination number, ECN; and net atomic charge, Q.

Non-equivalent species	N	d_{NN} (Å)	ECN (NNN)	Q (e^-)
Ba(I)	2	2.5031	6.3187	1.472 367
Ba(II)	2	2.5546	6.7567	1.453 323
Ba(III)	2	2.4382	5.9280	1.439 464
Ba(IV)	2	2.6050	6.8242	1.515 305
Ba(V)	2	2.5190	5.3302	1.505 470
Ba(VI)	2	2.4886	6.3086	1.416 043
Ba(VII)	2	2.4885	6.4669	1.451 449
Ba(VIII)	2	2.5374	6.5894	1.476 805
V(I)	2	1.7160	3.9973	1.939 315
V(II)	2	1.7050	3.9890	1.964 289
V(III)	2	1.7085	3.9943	1.931 673
V(IV)	2	1.7262	3.9999	1.956 514
O(I)	2	1.7353	1.0000	−1.065 080
O(II)	2	1.7160	1.0000	−1.028 051
O(III)	2	1.7190	1.0000	−1.027 001
O(IV)	2	1.7294	1.0000	−1.087 010
O(V)	2	1.7405	1.0000	−1.080 027
O(VI)	2	1.7310	1.0000	−1.052 218
O(VII)	2	1.7458	1.0000	−1.081 883
O(VIII)	2	1.7050	1.0000	−1.022 563
O(IX)	2	1.7310	1.0001	−1.109 873
O(X)	2	1.7113	1.0000	−1.035 232
O(XI)	2	1.7085	1.0000	−1.025 393
O(XII)	2	1.7336	1.0000	−1.090 808
O(XIII)	2	1.7281	1.0000	−1.054 412
O(XIV)	2	1.7294	1.0000	−1.052 563
O(XV)	2	1.7272	1.0000	−1.069 190
O(XVI)	2	1.7262	1.0000	−1.068 646
O(XVII)	2	2.4886	4.0482	−1.293 129
O(XVIII)	2	2.4382	4.0562	−1.278 940

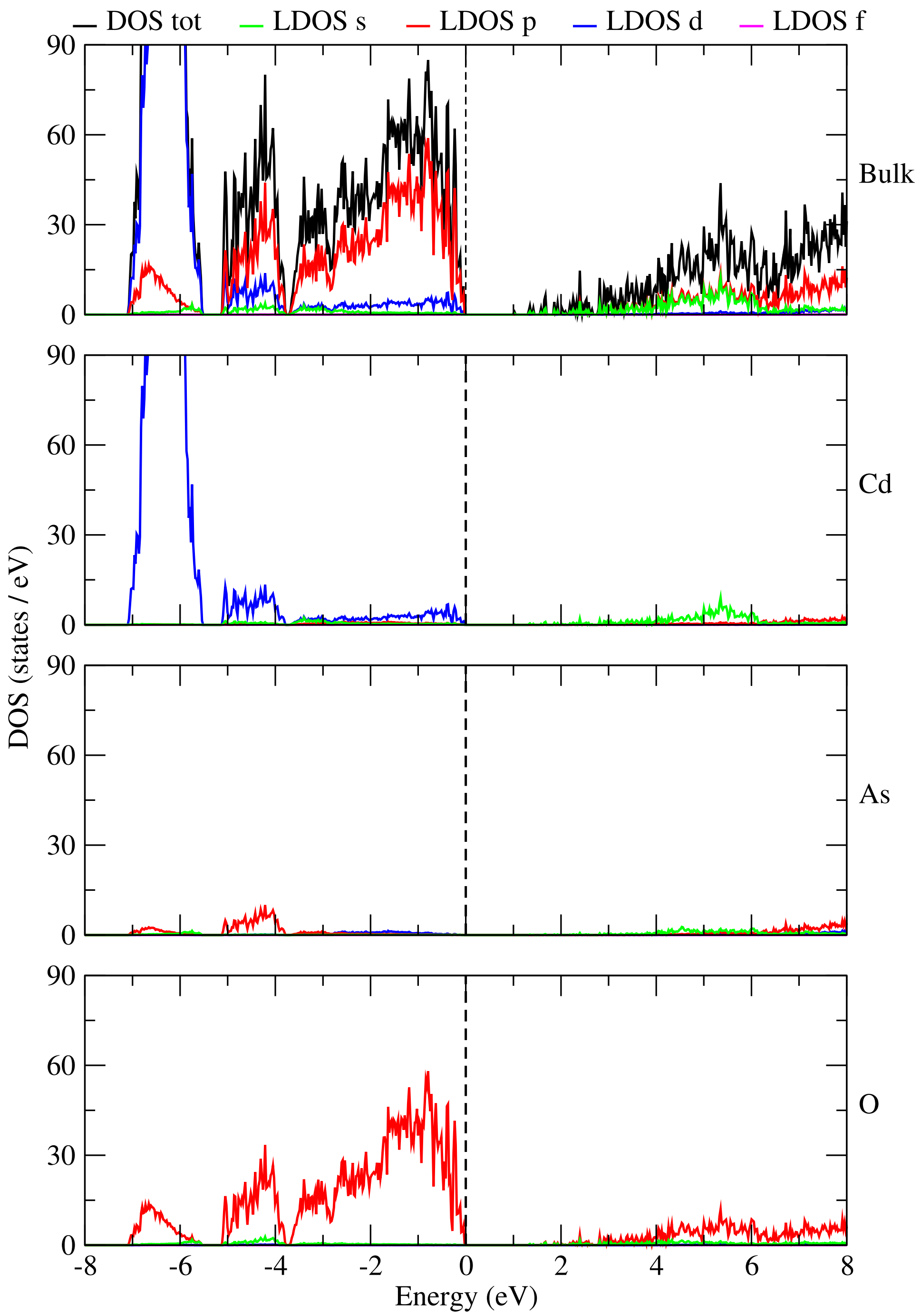

Figure S-143: Local density of states for the $Cd_4(AsO_4)_2O$ bulk phase.

Table S-146: Calculated properties for the $Cd_4(AsO_4)_2O$ bulk phase. Number of non-equivalent species, N; average distance for nearest neighbors, d_{NN}; effective coordination number, ECN; and net atomic charge, Q.

Non-equivalent species	N	d_{NN} (Å)	ECN (NNN)	Q (e^-)
Cd(I)	2	2.2080	5.5589	1.097 731
Cd(II)	2	2.2623	5.7475	1.086 180
Cd(III)	2	2.1714	4.7997	1.087 794
Cd(IV)	2	2.2749	5.8868	1.155 140
Cd(V)	2	2.2094	5.1699	1.136 951
Cd(VI)	2	2.1872	4.6697	1.032 060
Cd(VII)	2	2.1417	5.3954	1.055 859
Cd(VIII)	2	2.1656	5.2994	1.075 692
As(I)	2	1.7047	3.9798	1.620 336
As(II)	2	1.7047	3.9806	1.615 945
As(III)	2	1.6842	3.9635	1.613 283
As(IV)	2	1.7141	3.9966	1.648 310
O(I)	2	1.7596	1.0862	−0.824 951
O(II)	2	1.7165	1.0072	−0.807 870
O(III)	2	1.7047	1.0404	−0.817 753
O(IV)	2	1.7114	1.0308	−0.814 942
O(V)	2	1.7538	1.0498	−0.870 890
O(VI)	2	1.7126	1.0123	−0.830 266
O(VII)	2	1.7458	1.0344	−0.836 410
O(VIII)	2	1.7047	1.0004	−0.819 107
O(IX)	2	1.7651	1.0710	−0.854 877
O(X)	2	1.6842	1.0549	−0.808 655
O(XI)	2	1.7197	1.0818	−0.832 444
O(XII)	2	1.7241	1.0359	−0.813 209
O(XIII)	2	1.7355	1.0435	−0.834 280
O(XIV)	2	1.7162	1.0198	−0.826 439
O(XV)	2	1.7280	1.0314	−0.857 408
O(XVI)	2	1.7141	1.0526	−0.841 261
O(XVII)	2	2.1872	3.9387	−0.992 138
O(XVIII)	2	2.1417	4.0576	−0.942 383

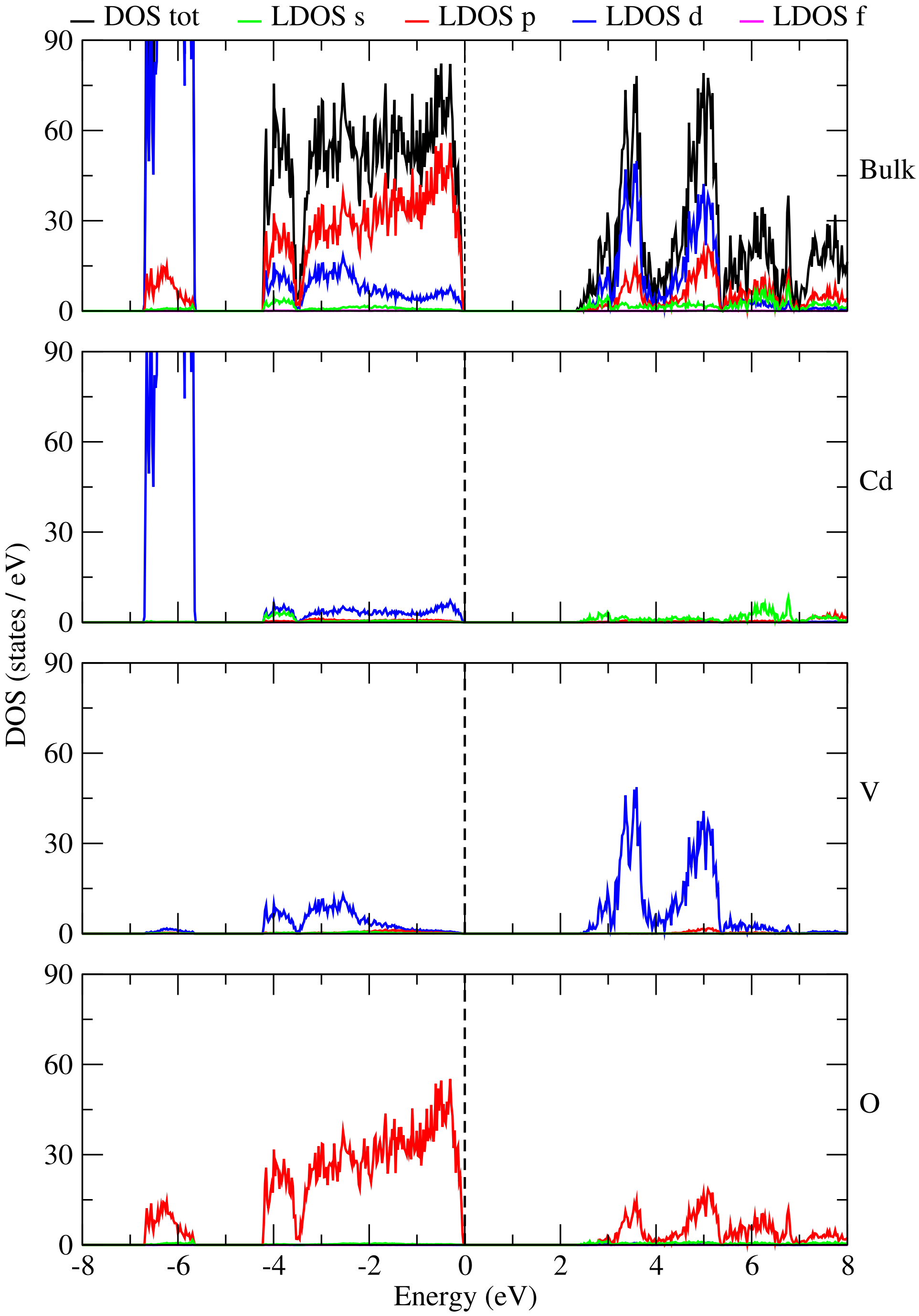

Figure S-144: Local density of states for the $Cd_4(VO_4)_2O$ bulk phase.

Table S-147: Calculated properties for the $Cd_4(VO_4)_2O$ bulk phase. Number of non-equivalent species, N; average distance for nearest neighbors, d_{NN}; effective coordination number, ECN; and net atomic charge, Q.

Non-equivalent species	N	d_{NN} (Å)	ECN (NNN)	Q (e^-)
Cd(I)	2	2.1694	5.0945	1.115 142
Cd(II)	2	2.1811	4.0135	1.159 232
Cd(III)	2	2.1694	4.7951	1.109 566
Cd(IV)	2	2.2439	5.9112	1.193 402
Cd(V)	2	2.2323	5.9034	1.198 755
Cd(VI)	2	2.1649	4.7567	1.110 605
Cd(VII)	2	2.1678	5.0758	1.115 610
Cd(VIII)	2	2.1718	4.2083	1.158 247
V(I)	2	1.6779	3.9364	2.003 037
V(II)	2	1.6986	3.9741	1.981 206
V(III)	2	1.6809	3.9305	1.998 548
V(IV)	2	1.7055	3.9816	1.987 819
O(I)	2	1.6779	1.0861	−0.876 984
O(II)	2	1.7725	1.1244	−0.983 734
O(III)	2	1.7023	1.0516	−0.909 341
O(IV)	2	1.7518	1.3882	−0.958 605
O(V)	2	1.7558	1.2778	−0.985 799
O(VI)	2	1.6986	1.0161	−0.945 847
O(VII)	2	1.7169	1.0217	−0.944 468
O(VIII)	2	1.7528	1.0409	−0.967 201
O(IX)	2	1.7617	1.4306	−0.964 844
O(X)	2	1.6930	1.0483	−0.899 271
O(XI)	2	1.7688	1.1117	−0.980 642
O(XII)	2	1.6809	1.0887	−0.874 837
O(XIII)	2	1.7411	1.0456	−0.958 275
O(XIV)	2	1.7150	1.0296	−0.940 562
O(XV)	2	1.7569	1.2974	−0.983 768
O(XVI)	2	1.7055	1.0175	−0.952 840
O(XVII)	2	2.1649	3.7891	−1.002 548
O(XVIII)	2	2.1694	3.8137	−1.001 601

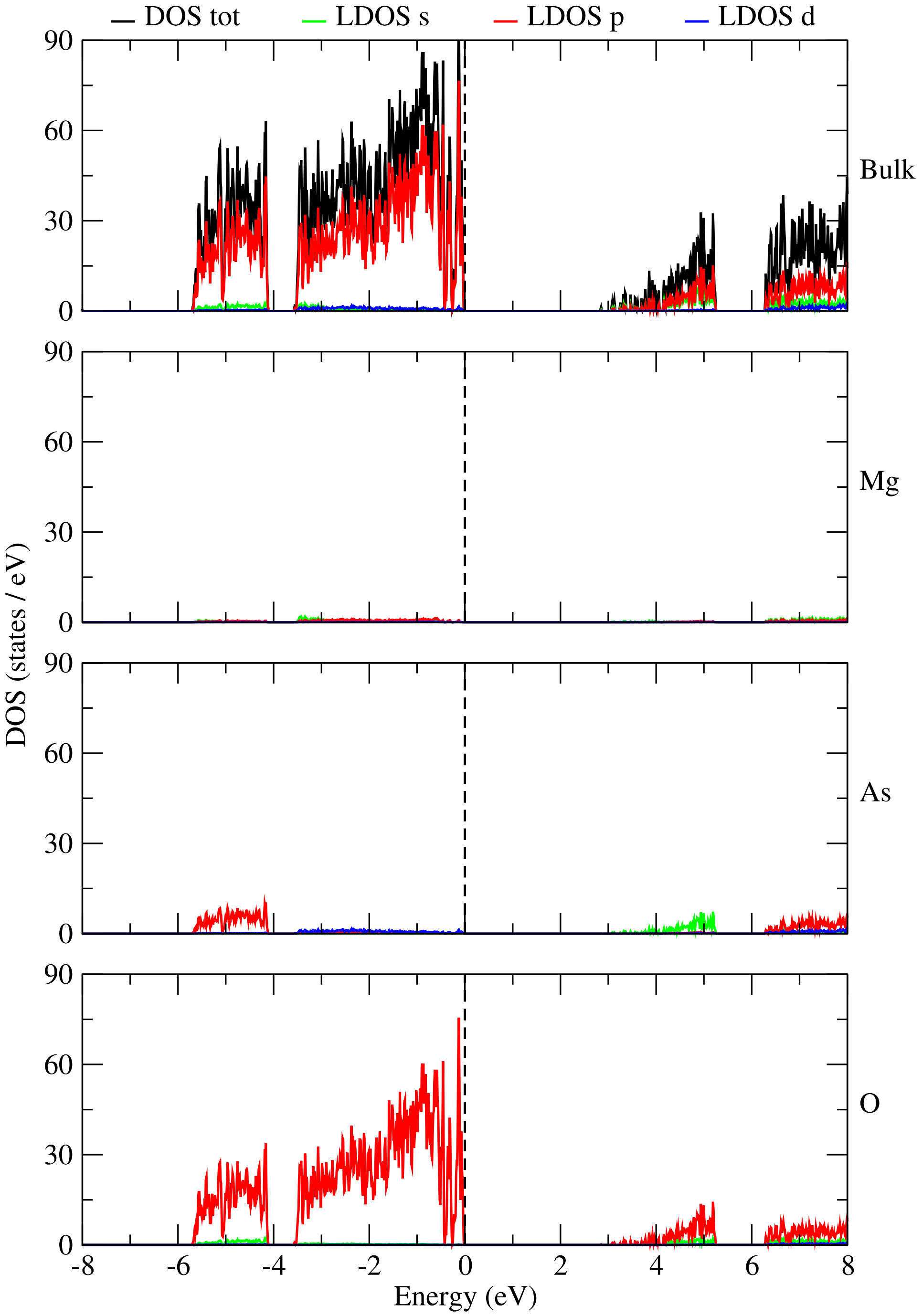

Figure S-145: Local density of states for the $Mg_4(AsO_4)_2O$ bulk phase.

Table S-148: Calculated properties for the $Mg_4(AsO_4)_2O$ bulk phase. Number of non-equivalent species, N; average distance for nearest neighbors, d_{NN}; effective coordination number, ECN; and net atomic charge, Q.

Non-equivalent species	N	d_{NN} (Å)	ECN (NNN)	Q (e^-)
Mg(I)	2	1.9988	4.9426	1.481 394
Mg(II)	2	1.9512	4.1761	1.435 496
Mg(III)	2	2.0524	5.0202	1.461 511
Mg(IV)	2	1.9874	5.3535	1.498 032
Mg(V)	2	1.9557	4.9042	1.486 382
Mg(VI)	2	2.0147	4.8388	1.451 176
Mg(VII)	2	2.0103	4.0800	1.464 109
Mg(VIII)	2	1.9573	3.9876	1.475 074
As(I)	2	1.6657	3.9581	1.763 219
As(II)	2	1.7122	3.9983	1.719 972
As(III)	2	1.6621	3.9429	1.757 129
As(IV)	2	1.7134	3.9945	1.771 695
O(I)	2	1.7323	2.1492	−1.006 841
O(II)	2	1.7395	1.7217	−0.972 316
O(III)	2	1.6657	1.4562	−0.953 414
O(IV)	2	1.7329	2.2843	−1.015 754
O(V)	2	1.7189	1.5148	−0.988 673
O(VI)	2	1.7122	1.9310	−0.974 890
O(VII)	2	1.7293	2.3020	−1.033 349
O(VIII)	2	1.7193	1.2466	−0.949 620
O(IX)	2	1.7411	2.1329	−1.047 818
O(X)	2	1.6621	1.5375	−0.937 301
O(XI)	2	1.7543	2.2460	−1.021 228
O(XII)	2	1.7213	1.9902	−0.976 718
O(XIII)	2	1.7399	1.1449	−0.967 693
O(XIV)	2	1.7154	1.4351	−0.991 748
O(XV)	2	1.7140	2.2868	−1.013 747
O(XVI)	2	1.7134	1.7279	−1.005 092
O(XVII)	2	1.9512	3.9528	−1.456 483
O(XVIII)	2	1.9573	3.9511	−1.452 504

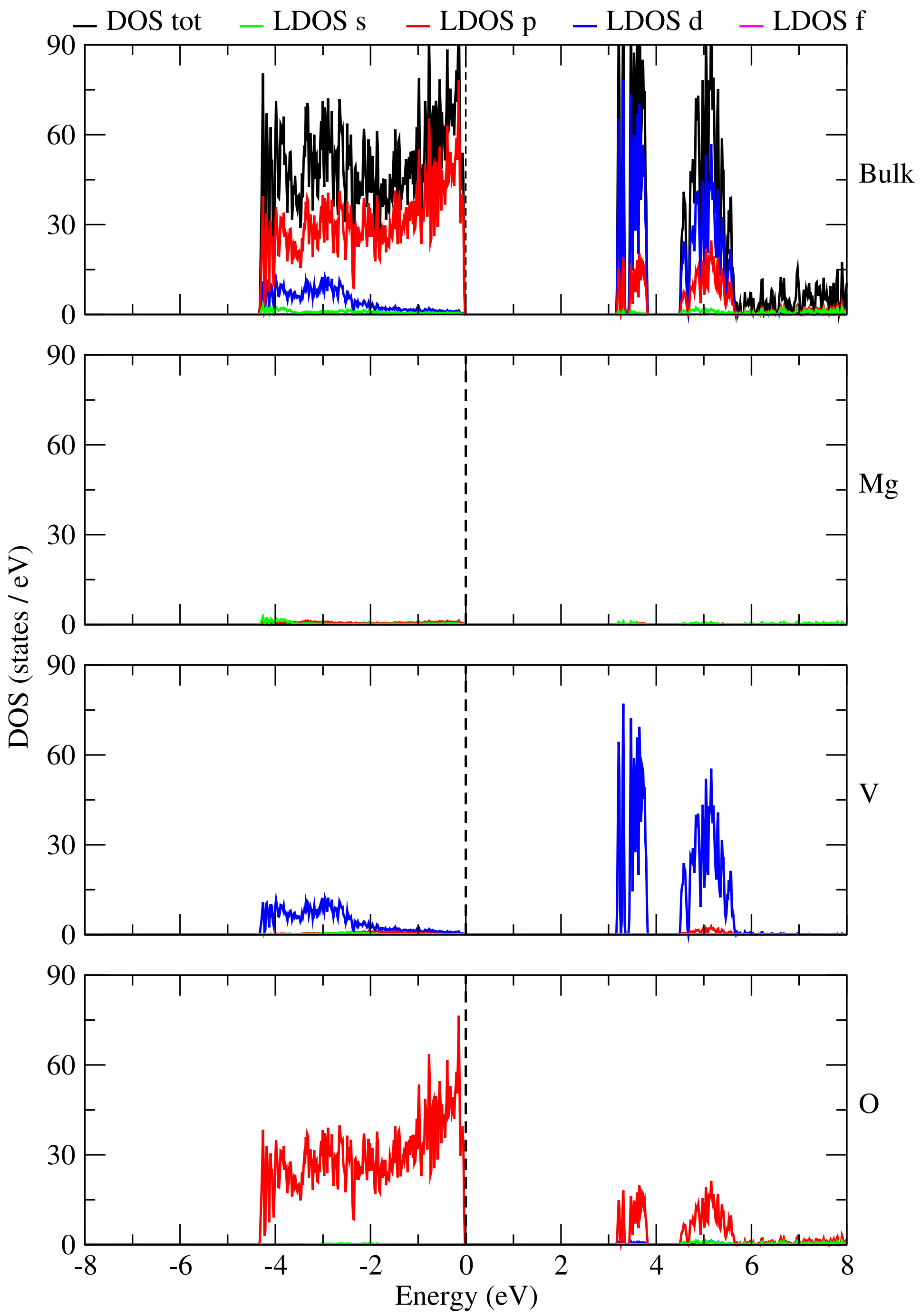

Figure S-146: Local density of states for the $Mg_4(VO_4)_2O$ bulk phase.

Table S-149: Calculated properties for the $Mg_4(VO_4)_2O$ bulk phase. Number of non-equivalent species, N; average distance for nearest neighbors, d_{NN}; effective coordination number, ECN; and net atomic charge, Q.

Non-equivalent species	N	d_{NN} (Å)	ECN (NNN)	Q (e^-)
Mg(I)	2	2.0175	5.0003	1.492 778
Mg(II)	2	1.9991	4.7123	1.485 304
Mg(III)	2	2.0430	5.7172	1.488 226
Mg(IV)	2	2.0611	5.8132	1.507 943
Mg(V)	2	2.0125	5.8634	1.522 942
Mg(VI)	2	2.0098	4.9450	1.490 250
Mg(VII)	2	1.9958	4.7595	1.482 293
Mg(VIII)	2	1.9437	4.1657	1.528 857
V(I)	2	1.6955	3.9547	2.078 479
V(II)	2	1.7077	3.9921	1.999 881
V(III)	2	1.6752	3.9304	2.080 888
V(IV)	2	1.6968	3.9897	2.084 977
O(I)	2	1.6955	1.4940	−1.009 636
O(II)	2	1.7759	2.7258	−1.140 328
O(III)	2	1.7001	1.6229	−1.050 538
O(IV)	2	1.7340	2.2726	−1.106 302
O(V)	2	1.7077	1.8417	−1.064 778
O(VI)	2	1.7168	1.8327	−1.089 801
O(VII)	2	1.7099	1.4748	−1.066 922
O(VIII)	2	1.7414	1.4249	−1.103 339
O(IX)	2	1.7394	2.2822	−1.153 565
O(X)	2	1.6752	1.5827	−1.011 485
O(XI)	2	1.7846	3.0788	−1.173 109
O(XII)	2	1.7146	1.5659	−1.025 424
O(XIII)	2	1.7087	1.7480	−1.061 758
O(XIV)	2	1.6968	1.6980	−1.048 317
O(XV)	2	1.7315	2.3513	−1.124 684
O(XVI)	2	1.7322	1.7113	−1.148 245
O(XVII)	2	1.9991	3.8498	−1.422 325
O(XVIII)	2	1.9437	3.9358	−1.442 262

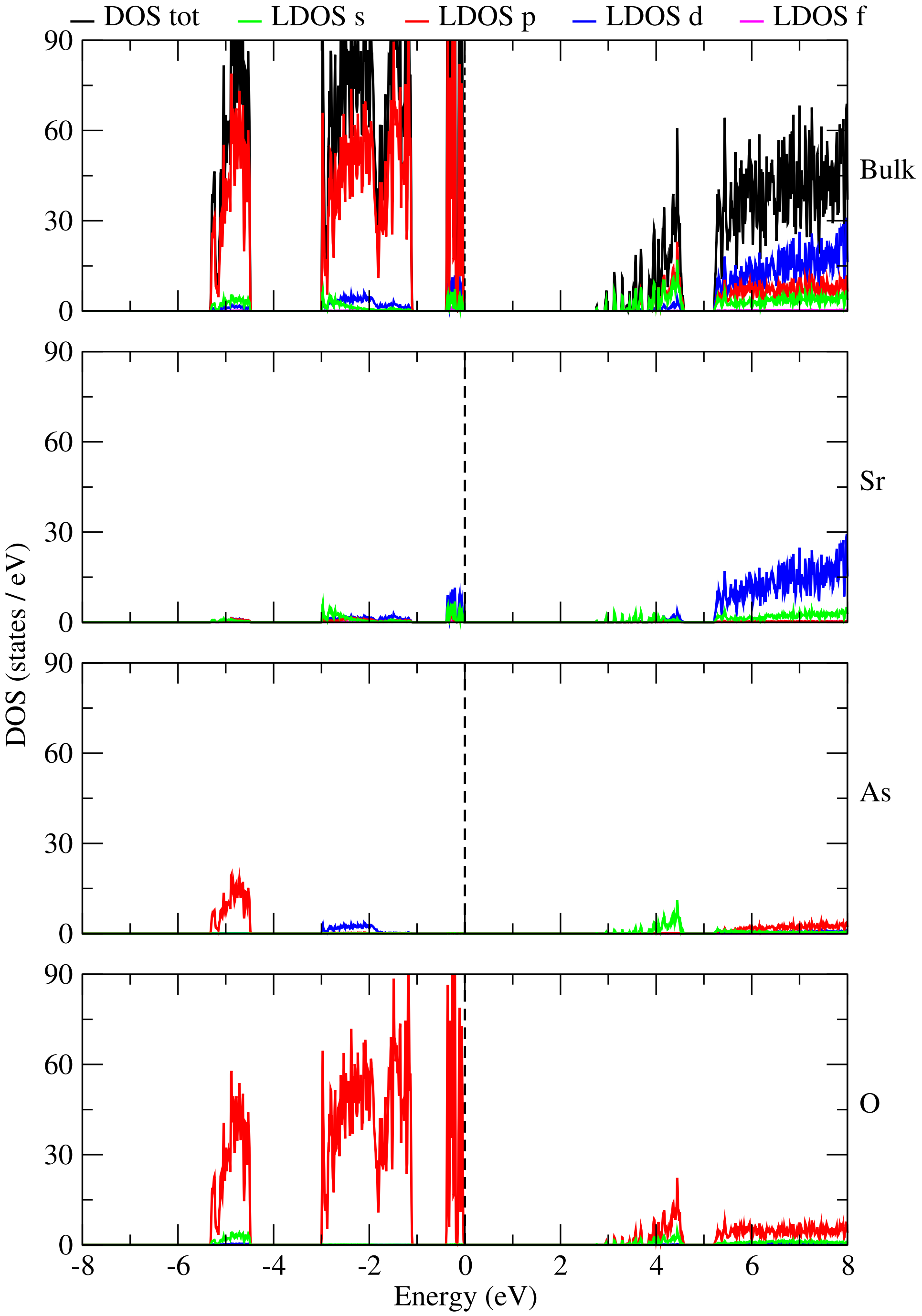

Figure S-147: Local density of states for the $Sr_4(AsO_4)_2O$ bulk phase.

Table S-150: Calculated properties for the $Sr_4(AsO_4)_2O$ bulk phase. Number of non-equivalent species, N; average distance for nearest neighbors, d_{NN}; effective coordination number, ECN; and net atomic charge, Q.

Non-equivalent species	N	d_{NN} (Å)	ECN (NNN)	Q (e^-)
Sr(I)	2	2.3745	6.2241	1.449 075
Sr(II)	2	2.4091	6.5807	1.437 783
Sr(III)	2	2.3036	5.4365	1.428 571
Sr(IV)	2	2.4642	6.6798	1.482 110
Sr(V)	2	2.3874	5.3964	1.486 996
Sr(VI)	2	2.3695	6.1743	1.413 870
Sr(VII)	2	2.3339	6.3402	1.421 384
Sr(VIII)	2	2.3845	6.5048	1.448 509
As(I)	2	1.7160	3.9974	1.614 500
As(II)	2	1.7035	3.9910	1.647 096
As(III)	2	1.7108	3.9980	1.602 347
As(IV)	2	1.7163	3.9983	1.631 935
O(I)	2	1.7354	1.0004	−0.969 536
O(II)	2	1.7160	1.0000	−0.933 096
O(III)	2	1.7187	1.0002	−0.946 154
O(IV)	2	1.7195	1.0005	−0.975 252
O(V)	2	1.7362	1.0006	−0.991 847
O(VI)	2	1.7269	1.0000	−0.952 073
O(VII)	2	1.7401	1.0004	−0.978 214
O(VIII)	2	1.7035	1.0001	−0.926 150
O(IX)	2	1.7230	1.0010	−0.985 722
O(X)	2	1.7124	1.0001	−0.948 434
O(XI)	2	1.7108	1.0007	−0.947 938
O(XII)	2	1.7263	1.0000	−0.966 541
O(XIII)	2	1.7226	1.0002	−0.957 706
O(XIV)	2	1.7329	1.0002	−0.971 688
O(XV)	2	1.7199	1.0002	−0.958 844
O(XVI)	2	1.7163	1.0002	−0.962 538
O(XVII)	2	2.3695	4.0641	−1.350 503
O(XVIII)	2	2.3036	4.0404	−1.341 940

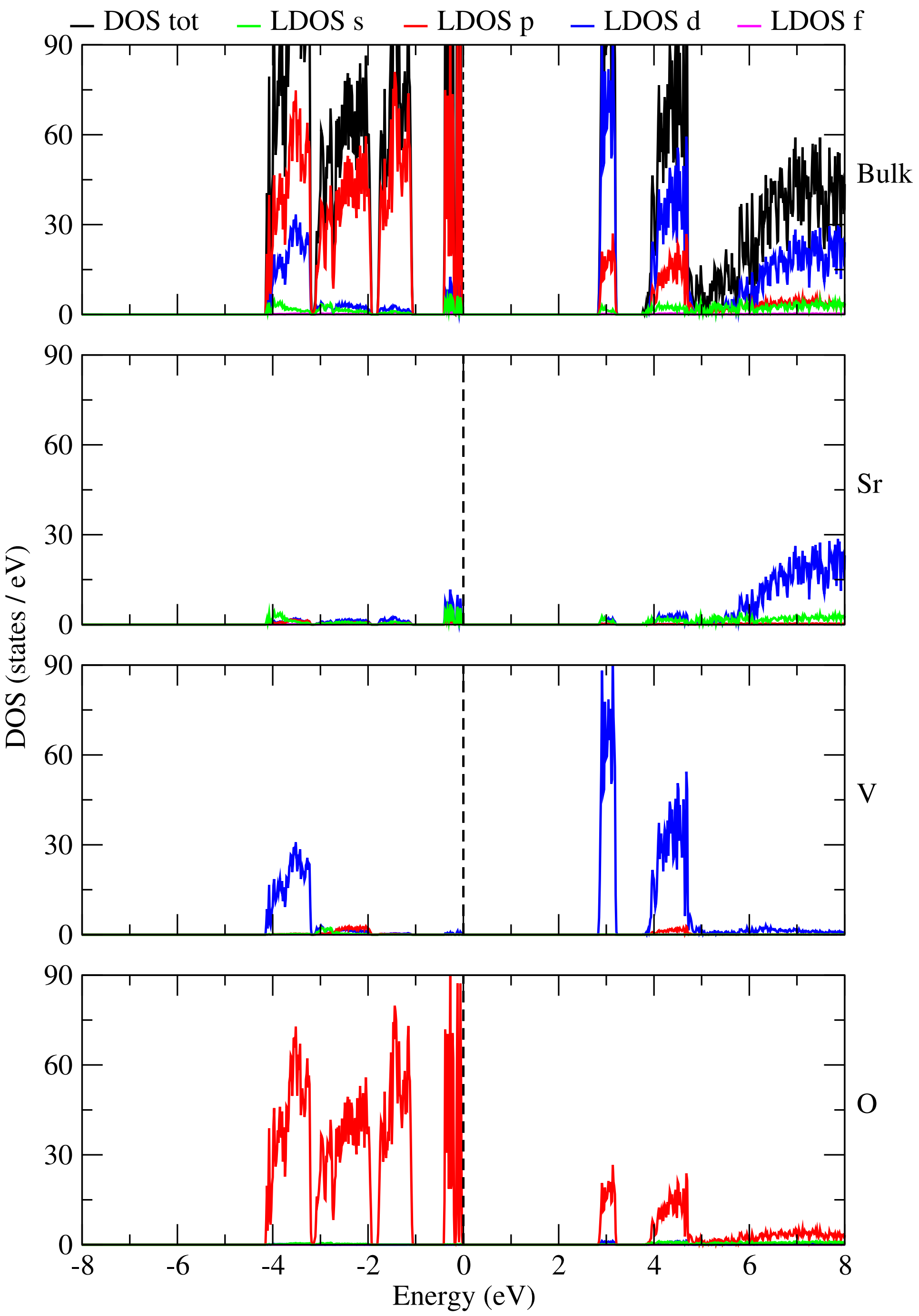

Figure S-148: Local density of states for the $Sr_4(VO_4)_2O$ bulk phase.

Table S-151: Calculated properties for the $Sr_4(VO_4)_2O$ bulk phase. Number of non-equivalent species, N; average distance for nearest neighbors, d_{NN}; effective coordination number, ECN; and net atomic charge, Q.

Non-equivalent species	N	d_{NN} (Å)	ECN (NNN)	Q (e^-)
Sr(I)	2	2.3795	6.2166	1.492 595
Sr(II)	2	2.4244	6.6881	1.471 506
Sr(III)	2	2.3123	5.5609	1.468 347
Sr(IV)	2	2.4537	6.7613	1.527 040
Sr(V)	2	2.3881	5.0465	1.528 166
Sr(VI)	2	2.3643	5.9128	1.436 688
Sr(VII)	2	2.3430	6.3216	1.466 012
Sr(VIII)	2	2.3982	6.3846	1.493 639
V(I)	2	1.7123	3.9950	1.933 093
V(II)	2	1.6956	3.9830	1.962 149
V(III)	2	1.7005	3.9897	1.922 286
V(IV)	2	1.7210	3.9998	1.955 998
O(I)	2	1.7379	1.0012	−1.074 526
O(II)	2	1.7123	1.0001	−1.025 239
O(III)	2	1.7123	1.0001	−1.020 566
O(IV)	2	1.7249	1.0007	−1.082 776
O(V)	2	1.7379	1.0013	−1.094 822
O(VI)	2	1.7289	1.0001	−1.053 828
O(VII)	2	1.7473	1.0005	−1.093 513
O(VIII)	2	1.6956	1.0000	−1.006 894
O(IX)	2	1.7354	1.0017	−1.130 609
O(X)	2	1.7073	1.0002	−1.037 396
O(XI)	2	1.7005	1.0006	−1.019 816
O(XII)	2	1.7319	1.0000	−1.074 327
O(XIII)	2	1.7254	1.0003	−1.058 829
O(XIV)	2	1.7210	1.0003	−1.043 130
O(XV)	2	1.7244	1.0003	−1.077 641
O(XVI)	2	1.7214	1.0004	−1.073 610
O(XVII)	2	2.3643	4.0622	−1.350 116
O(XVIII)	2	2.3123	4.0630	−1.339 881

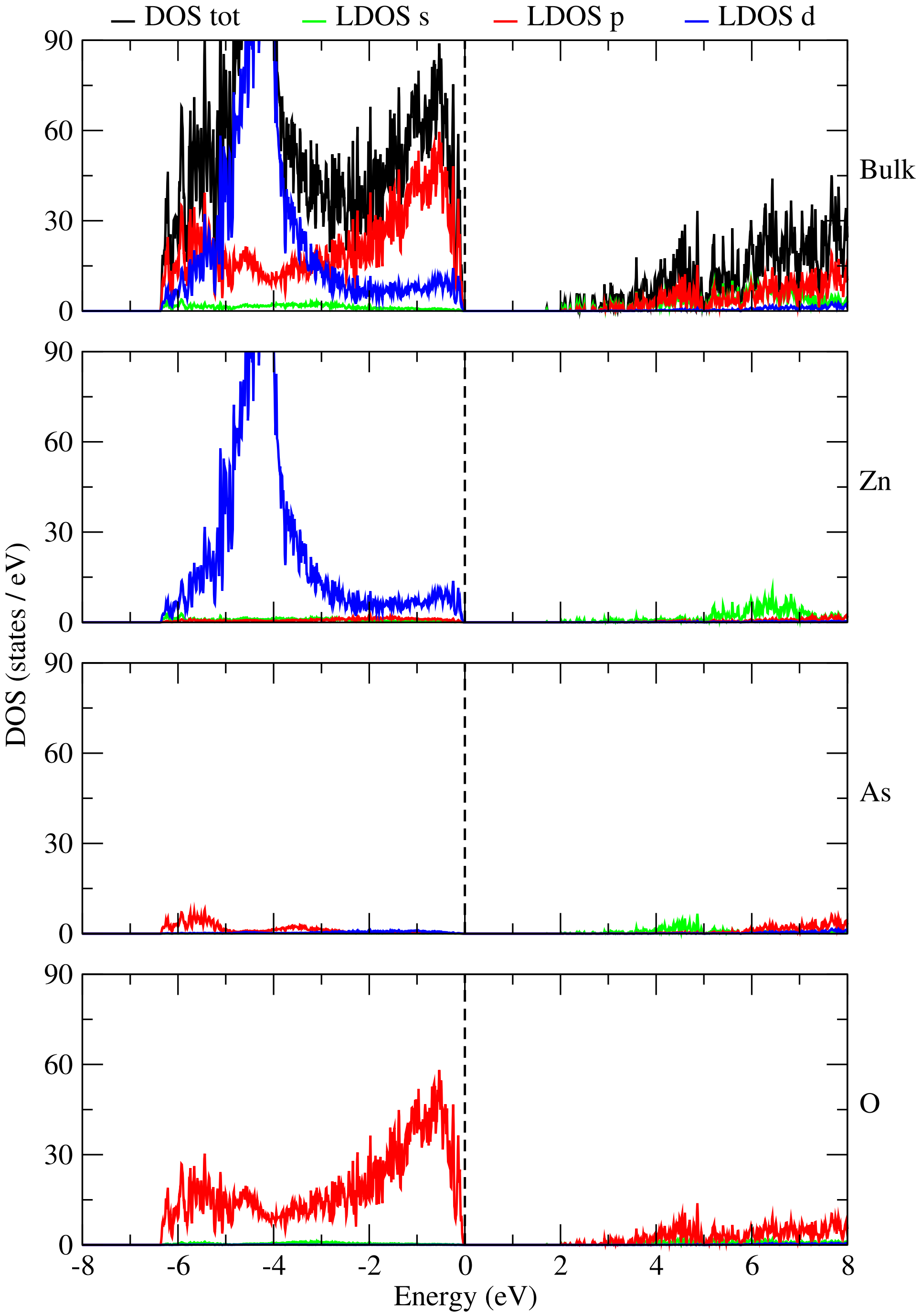

Figure S-149: Local density of states for the $Zn_4(AsO_4)_2O$ bulk phase.

Table S-152: Calculated properties for the $Zn_4(AsO_4)_2O$ bulk phase. Number of non-equivalent species, N; average distance for nearest neighbors, d_{NN}; effective coordination number, ECN; and net atomic charge, Q.

Non-equivalent species	N	d_{NN} (Å)	ECN (NNN)	Q (e^-)
Zn(I)	2	2.0116	4.7248	1.018 155
Zn(II)	2	2.0210	4.7825	1.024 450
Zn(III)	2	2.0265	5.7417	1.036 931
Zn(IV)	2	2.0738	5.4194	1.084 057
Zn(V)	2	2.0358	5.0675	1.085 840
Zn(VI)	2	1.9992	4.7137	1.030 129
Zn(VII)	2	1.9355	3.9204	1.010 406
Zn(VIII)	2	1.9273	3.9620	1.029 674
As(I)	2	1.7146	3.9928	1.718 862
As(II)	2	1.7068	3.9968	1.649 355
As(III)	2	1.7086	3.9908	1.705 164
As(IV)	2	1.7128	3.9936	1.708 642
O(I)	2	1.7146	1.7477	−0.828 253
O(II)	2	1.7360	1.4732	−0.798 698
O(III)	2	1.7162	1.7727	−0.835 570
O(IV)	2	1.7445	2.3861	−0.820 415
O(V)	2	1.7228	1.9210	−0.822 892
O(VI)	2	1.7068	1.4830	−0.821 880
O(VII)	2	1.7300	1.8999	−0.838 315
O(VIII)	2	1.7192	1.5893	−0.818 100
O(IX)	2	1.7435	2.2434	−0.863 784
O(X)	2	1.7086	2.0349	−0.827 072
O(XI)	2	1.7404	1.6831	−0.802 355
O(XII)	2	1.7202	1.6839	−0.781 081
O(XIII)	2	1.7206	1.9173	−0.825 801
O(XIV)	2	1.7152	1.6853	−0.814 066
O(XV)	2	1.7433	2.3316	−0.824 994
O(XVI)	2	1.7128	1.0929	−0.844 474
O(XVII)	2	1.9992	3.9466	−0.965 077
O(XVIII)	2	1.9273	3.9547	−0.968 835

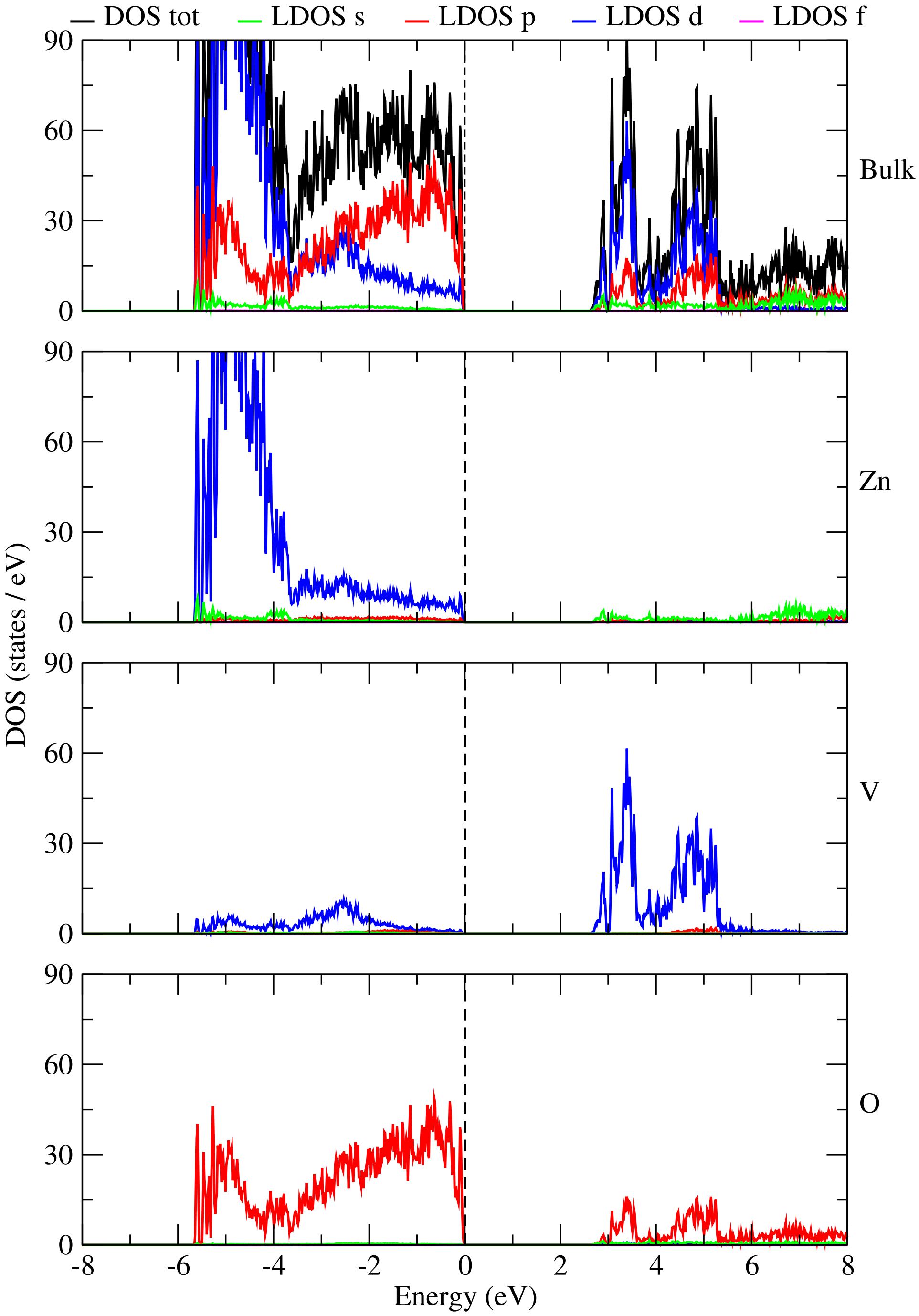

Figure S-150: Local density of states for the $Zn_4(VO_4)_2O$ bulk phase.

Table S-153: Calculated properties for the $Zn_4(VO_4)_2O$ bulk phase. Number of non-equivalent species, N; average distance for nearest neighbors, d_{NN}; effective coordination number, ECN; and net atomic charge, Q.

Non-equivalent species	N	d_{NN} (Å)	ECN (NNN)	Q (e^-)
Zn(I)	2	1.9266	3.8738	1.085 806
Zn(II)	2	1.9367	3.8646	1.065 772
Zn(III)	2	1.9768	3.9733	1.114 181
Zn(IV)	2	1.9170	3.7791	1.063 492
Zn(V)	2	1.9303	3.9986	1.063 962
Zn(VI)	2	1.9508	3.9594	1.089 157
Zn(VII)	2	1.9287	3.9173	1.033 891
Zn(VIII)	2	1.9435	4.3003	1.128 534
V(I)	2	1.6728	3.8801	2.010 144
V(II)	2	1.6506	3.8579	2.002 511
V(III)	2	1.6670	3.8915	1.995 970
V(IV)	2	1.6920	3.9518	2.014 619
O(I)	2	1.6906	1.6996	−0.874 238
O(II)	2	1.8026	2.7964	−0.953 930
O(III)	2	1.6728	1.6780	−0.830 097
O(IV)	2	1.7586	2.4810	−0.947 679
O(V)	2	1.7986	2.7878	−0.980 208
O(VI)	2	1.7679	2.4553	−0.960 885
O(VII)	2	1.7172	1.5764	−0.915 643
O(VIII)	2	1.6506	1.1032	−0.855 436
O(IX)	2	1.7827	2.7241	−0.987 939
O(X)	2	1.6670	1.6251	−0.829 445
O(XI)	2	1.7672	2.6710	−0.930 408
O(XII)	2	1.6902	1.7037	−0.885 744
O(XIII)	2	1.7211	1.6583	−0.918 569
O(XIV)	2	1.7174	1.9540	−0.920 097
O(XV)	2	1.7828	2.6227	−0.992 657
O(XVI)	2	1.6920	1.6480	−0.918 438
O(XVII)	2	1.9745	3.8618	−0.984 201
O(XVIII)	2	1.9399	4.0047	−0.982 424

References

1 Kresse, G.; Hafner, J. *Ab initio* Molecular Dynamics for Open-Shell Transition Metals. *Phys. Rev. B* **1993**, *48*, 13115–13118, DOI: 10.1103/physrevb.48.13115.

2 Kresse, G.; Furthmüller, J. Efficient Iterative Schemes For *Ab Initio* Total-Energy

Calculations Using a Plane-Wave Basis Set. *Phys. Rev. B* **1996**, *54*, 11169–11186, DOI: 10.1103/physrevb.54.11169.

3 Blöchl, P. E. Projector Augmented-Wave Method. *Phys. Rev. B* **1994**, *50*, 17953–17979, DOI: 10.1103/PhysRevB.50.17953.

4 Kresse, G.; Joubert, D. From Ultrasoft Pseudopotentials to the Projector Augmented-Wave Method. *Phys. Rev. B* **1999**, *59*, 1758–1775, DOI: 10.1103/physrevb.59.1758.

5 Nespolo, M. Charge Distribution as a Tool to Investigate Structural Details. IV. A New Route to Heteroligand Polyhedra. *Acta Crystallogr., Sect. B: Struct. Sci., Cryst. Eng. Mater.* **2016**, *72*, 51–66, DOI: 10.1107/S2052520615019472.

6 Otero-de-la Roza, A.; Johnson, E. R.; Luaña, V. Critic2: A Program for Real-Space Analysis of Quantum Chemical Interactions in Solids. *Comput. Phys. Commun.* **2014**, *185*, 1007–1018, DOI: 10.1016/j.cpc.2013.10.026.
